# Metal Free Synthesis of Seven‐Membered Biaryl Sultams

**DOI:** 10.1002/asia.202500969

**Published:** 2025-11-24

**Authors:** Hasil Aman, Bo‐sen Huang, Jian‐Yu Liao, Vijaykumar H. Thorat, Zhe‐yi Liao, Yu‐Hao Liu, Luo‐Ting Yen, Jen‐Chieh Hsieh, Gary Jing Chuang

**Affiliations:** ^1^ Department of Chemistry Chung Yuan Christian University Taoyuan Taiwan (ROC); ^2^ Department of Chemistry Tamkang University New Taipei Taiwan (ROC)

**Keywords:** benzothiatriazines, biaryl sultams, denitrogenative cyclization, diradical mechanism, metal‐free synthesis

## Abstract

A concise, metal‐free denitrogenative cyclization of benzothiatriazines was developed for the efficient synthesis of seven‐membered biaryl sultams. This method proceeds under simple thermal conditions and tolerates a broad substrate scope. The mechanistic study indicates that the reaction involves the formation of a diradical intermediate.

## Graphical abstract

A simple, metal‐free denitrogenative cyclization of benzothiatriazines enables efficient access to seven‐membered biaryl sultams under thermal conditions. The reaction tolerates diverse functional groups, and mechanistic studies, including radical trapping and EPR analysis, support a diradical pathway, providing new insights into sultam formation.

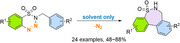

## 1 Introduction

**FIGURE 1:**
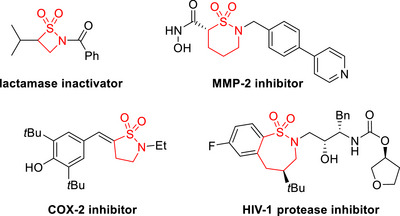
Sultam containing protein inhibitors.

Owing to their broad applications as pharmacophores, sulfonamide‐containing compounds have always captured the attention of medicinal and synthetic chemists [1, 2, 3, 4]. Often considered to be mimics of lactams, cyclic sulfonamides, also known as sultams are especially privileged moieties that can be found in a large number of bioactive compounds or pharmaceuticals [5, 6, 7, 8, 9, 10, 11]. For example, Figure 1 shows sultams ranging from four to seven‐membered rings, which can all be found in the core structures in reported inhibitors of biologically relevant proteins [12]. Moreover, sultams have also played important roles in organic synthesis, such as reagents for fluorination, trifluoromethylation, trifluoromethylthiolation, and also as a chiral auxiliary [13, 14, 15, 16, 17].

**SCHEME 1:**
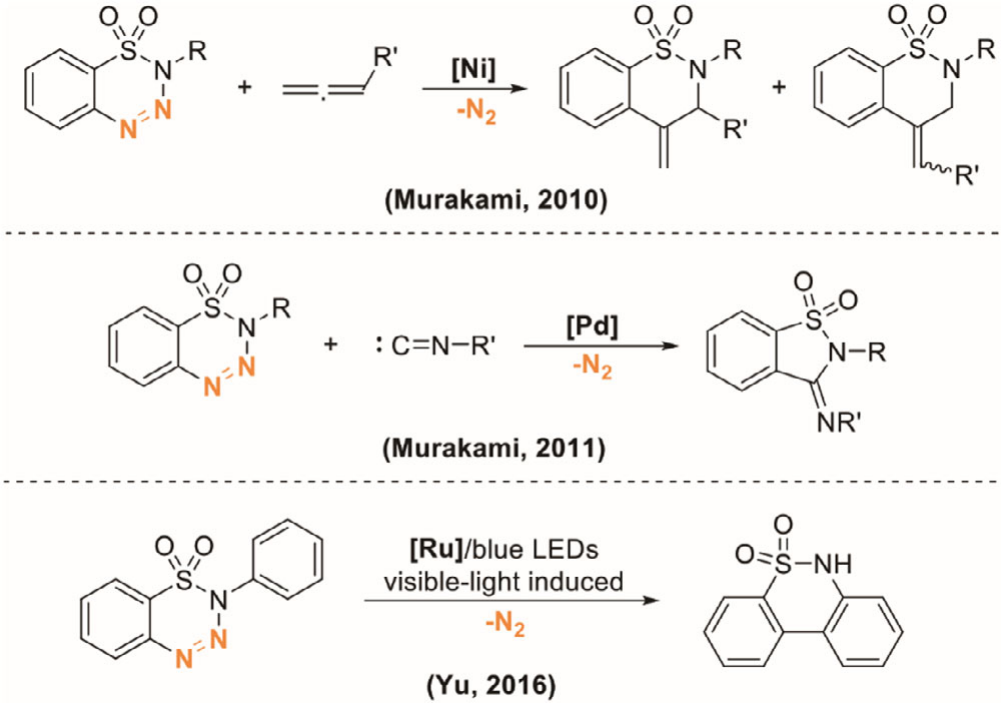
Transition‐metal‐catalyzed denitrogenative cyclization for the synthesis of benzosultams.

Because of the aforementioned importance of the sultam framework in both pharmaceutical applications and synthetic chemistry, various strategies have been developed to meet the demand for sultam synthesis [18, 19]. In addition to C–H activation approaches employing Pd, Rh, Cu, or Ir catalysts that enable access to structurally diverse benzosultams, the construction of biaryl and medium‐ to large‐ring sultams has also attracted significant attention. Palladium‐catalyzed intramolecular arylations have enabled access to biaryl and polycyclic sultams, [20, 21, 22] complemented by reductive, [23] electrochemical, [24] and radical‐mediated strategies for benzosultam formation [25]. Other approaches, including Sonogashira coupling‐cyclization, [26] Pd‐catalyzed hydrocarbonation, [27] and RCM, [28] further broaden the scope toward medium‐ to large‐ring sultams. A relevant method via transition‐metal‐catalyzed denitrogenative cyclization of benzothiatriazines provides another attractive pathway toward benzosultams. The pioneering work was first carried out by Murakami, combining denitrogenation with the insertion of unsaturated coupling partners (Scheme 1) [29, 30]. Later, Yu developed a visible‐light‐promoted denitrogenative cyclization to form biaryl sultams using a ruthenium photocatalyst [31]. Despite these advances, a concise, purely thermal, and metal‐free denitrogenative cyclization to access seven‐membered biaryl sultams has not yet been realized. Herein, we report such a strategy.

**SCHEME 2:**
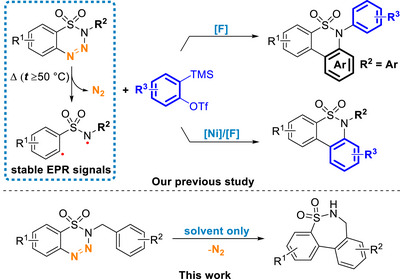
Our previous studies and the present work involving the denitrogenative cyclization.

Inspired by their pioneering works, we contributed two different denitrogenative cyclization reactions of benzothiatriazines with benzyne precursors (Scheme 2). In our studies, we found that a reactive diradical intermediate is formed through the thermal extrusion of a nitrogen molecule. This reactive diradical species can further undergo homolytic aromatic substitution or react with a nickel complex to form an aza‐nickelacycle intermediate [32, 33, 34]. With the subsequent steps, these two intermediates can lead to similar six‐membered biaryl sultams with different substitution types. In addition, our continuous effort have extended to the formation of seven‐membered biaryl sultams under very concise conditions. Herein, we would like to report a facile and metal‐free synthesis of seven‐membered biaryl sultams through a denitrogenative cyclization of benzothiatriazines.

## 2 Results and Discussion

We started our pilot experiments by using benzothiatriazine **1a** as the model substrate (Table 1). Starting with benzene as solvent, we were pleased to find that the desired product **2a** can be isolated from the reaction mixture in 37% yield. (Table 1, entry 1). The yield was improved to 63% by conducting the reaction in toluene (Table 1, entry 2), and the denitrogenative cyclization was also found to be reasonably compatible in polar solvents, such as DMF (29%), DMSO (42%), and ethanol (53%) (Table 1, entry 3–5). It is worth noting that the formation of side product **3a**, likely arising from hydrogen abstraction, was detected in most cases, particularly when the reaction was conducted in EtOH. And by using THF, **3a** became the major product in the reaction (Table 1, entry 6). The reaction performed in CH_3_CN (Table 1, entry 7) provided **2a** in 68% yield with trace amounts of the starting material remaining after the standard reaction time (∼14 h). As the reaction in CH_3_CN proceeded slightly slower than in other solvents, extending the reaction time to 18 h resulted in complete consumption of 1a, and afforded **2a** in 73% isolated yield.

**TABLE 1:** Optimization for seven‐membered sultam cyclization.

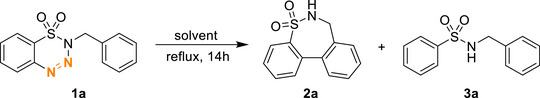			
Entry	Solvent^a^	Yield^b^ 2a (%)	Yield^b^ 3a (%)
1	Benzene	37	trace
2	Toluene	63	trace
3	DMF	29	trace
4	DMSO	42	0
5	Ethanol	53	21
6	THF	Trace	56
7	MeCN	68/73^c^	0

^a^ 2.2 mmol of **1a** was refluxed in 0.1 M solvent.

^b^ Isolated yield.

^c^ 18‐h reaction.

After the optimization of the reaction conditions, we then explored the functional group compatibility of this cyclization reaction. As indicated in Table 2, a wide range of 1,2,3,4‐benzothiatriazines with different substitution patterns have been surveyed. Starting with substituents at the *para*‐position of the *N*‐benzyl moiety on benzothiatriazines **1**, alkyls and methoxy groups were found to give 71–83% yield (Table 2, **2b**‐**2d**). Substrates with electron‐withdrawing groups were also found compatible as sultam products with ‐CF_3_ and cyano groups were isolated in 70–88% (Table 2, **2e** and **2** **h**). Though halogen (Cl and Br) and ester substituents in **2f**, **2** **g**, and **2i** gave slightly lower yields, around 50%. When substituents with potential pharmaceutical applications (CF_3_, F, and OCF_3_) were introduced at the meta‐position of the *N*‐benzyl moiety of **1**, **2j**, **2k**, and **2l** were obtained exclusively as the sole products. This selectivity is likely due to ring closure taking place at the less sterically hindered position.

**TABLE 2:** Substrates scope for seven membered‐sultams.

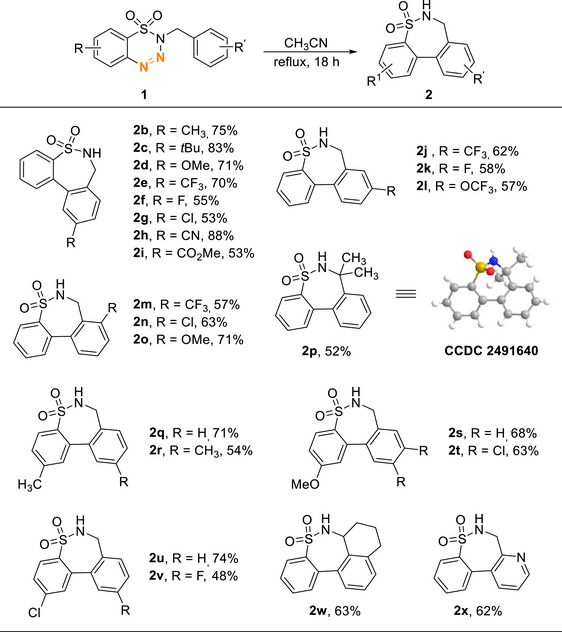

Isolated yield.

As for substrates **1m–o**, in which the ortho‐position is substituted with CF_3_, Cl, and OMe, the corresponding products **2m–o** were obtained in yields ranging from 57% to 71%. Substrates with quaternary carbon at the benzylic position (**1p**) was shown to be less reactive when compared to **1a**, resulting in the cyclized product **2p** in 52%. For substitutions on the aryl ring of benzothiatriazine, methyl (**2q** and **2r**), methoxy (**2s** and **2t**), and chloro (**2u** and **2v**) have shown practical compatibility, resulting in the desired products in yields of 48–74%. In the examples of **2w**, an extra ring at the benzylic position was accomplished in 63%. Pyridine was also found compatible in the cyclization as **2x** was isolated in 62%.

**SCHEME 3:**
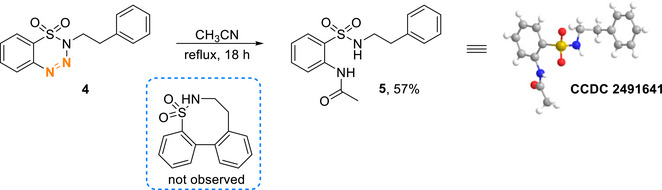
Unexpected formation of **5**.

Having successfully achieved the formation of the seven‐membered sultam, we next sought to examine whether this strategy could be extended to the synthesis of larger ring systems. As illustrated in Scheme 3, the attempted cyclization of substrate **4** did not afford the expected eight‐membered sultam. Instead, acetamide compound **5** was isolated and identified by X‐ray crystallographic analysis. The formation of **5** likely occurs after the extrusion of *N*_2_, with its side chain plausibly derived from the solvent, acetonitrile.

**SCHEME 4:**
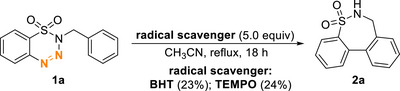
Radical scavenger experiments.

As mentioned in the introduction, in our previous experience, the denitrogenation of benzotriazene generated a diradical intermediate, which subsequently undergoes a cyclization reaction. Therefore, to understand whether the current reaction proceeds through a similar pathway, we attempted to add a radical scavenger to the reaction. As shown in Scheme 4, although the formation of **2a** still proceeded in the presence of BHT or TEMPO, the yield was significantly lower than the optimized condition (73%), suggesting the possible involvement of a radical intermediate in this transformation.

**SCHEME 5:**
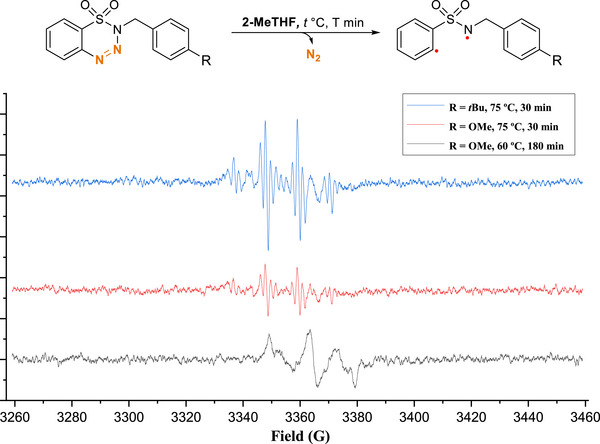
EPR spectra of **1c** and **1d** in 2‐MeTHF.

We further investigated the performance of benzothiatriazine in the EPR spectrum by employing the substrates **1c** and **1d** in 2‐MeTHF at various temperatures. As shown in Scheme 5, the EPR signals at a lower temperature clearly show a triplet pattern, and the *g* value is around 2.0054, which represents a typical nitrogen‐centered radical,[35, 36] caused by the release of a nitrogen molecule (black line). The carbon‐centered radical cannot have any EPR signal response since the nuclear spin of ^12^C is zero. When the substrates were measured at a higher temperature, the EPR signals appeared more quickly than at a lower temperature, and the profiles were different and more complex (red and blue lines). This result clearly indicates that the radical transfers from the nitrogen center to others. In addition, the complicated hyperfine coupling with a comparatively smaller coupling constant is probably due to the coupling with the protons.

**SCHEME 6:**
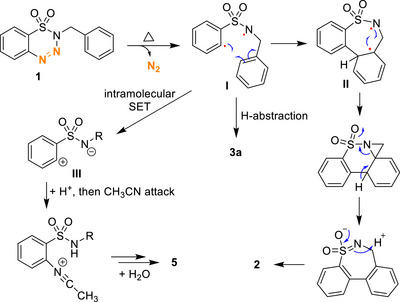
Proposed mechanism for the formation of Product **2**.

Based on the previous references and the results of mechanistic studies, the proposed mechanism is built as follows (Scheme 6). The reaction is initiated by benzothiatriazene **1** undergoing thermal decomposition to release a molecule of *N*_2_, which generates a diradical species **I**. The aryl radical then attacks the *ortho* position of the *N*‐benzyl group, leading to the formation of the seven‐membered ring intermediate **II**. Subsequently, the 1,3‐diradical of **II** cyclizes to form an aziridine ring. Deprotonation followed by ring‐opening of the aziridine and a final protonation step affords the desired sultam product **2**. The side product **3a** is likely formed through hydrogen abstraction by diradical **I**, which explains the higher yield of **3a** observed in THF. In the reaction of substrate **4**, the slower formation of the eight‐membered ring allows for an alternative pathway involving an intramolecular single‐electron transfer, generating zwitterion **III**. Subsequent protonation and nucleophilic attack by acetonitrile form the aryl‐N bond, and finally, the reaction of the cationic intermediate with water produces compound **5**.

## 3 Conclusion

In summary, we have developed a practical and metal‐free synthetic strategy for the efficient construction of seven‐membered biaryl sultams via the denitrogenative cyclization of benzothiatriazines. The transformation proceeds smoothly under simple thermal conditions without the need for any catalysts or additives, and demonstrates broad functional group tolerance. Mechanistic investigations, including control experiments with radical scavengers and EPR spectroscopic studies support the involvement of a diradical intermediate. These findings not only shed light on the underlying reaction pathway, but also expand the synthetic versatility and potential applications of biaryl sultam scaffolds in organic and medicinal chemistry (Supporting Information).

## Conflicts of Interests

The authors declare no conflict of interests.

## Supporting information




**Supplementary File 1**: asia70459‐sup‐0001‐Data.pdf

**Supplementary File 2**: asia70459‐sup‐0002‐SuppMat.zip

## Data Availability

The data that support the findings of this study are available in the supplementary material of this article.
