# Supplementary material for: Metal Free Synthesis of Seven‐Membered Biaryl Sultams
Source: Chem Asian J. 2025 Nov 24;20(24):e00969. doi: 10.1002/asia.202500969 (PMC12711382; doi:10.1002/asia.202500969)

Supporting Information

Metal free synthesis of Seven-membered Biaryl sultams.

Hasil Aman,[a] Bo-sen Huang,[a] Jian-Yu Liao,[b] Vijaykumar H. Thorat,[b] Zhe-yi Liao,[a] Yu-Hao Liu,[a] Luo Ting Yen,[b] Jen-Chieh Hsieh,[*b] Gary Jing Chuang[*a]

Table of Contents

Experimental 5
Genral procedure I: Preparation of substituted 2-benzyl-2*H*-benzo[*e*][1,2,3,4]thiatriazine 1,1-dioxide (1a-1x) 5
General procedure II: Synthesis of seven-membered sultams (2a-2x).......................5
2-benzyl-2l4-benzo[e][1,2,3,4]thiatriazine 1,1-dioxide, 1a 5
2-(4-methylbenzyl)-2H-benzo[e][1,2,3,4]thiatriazine 1,1-dioxide, 1b........................6
2-(4-(tert-butyl)benzyl)-2H-benzo[e][1,2,3,4]thiatriazine 1,1-dioxide, 1c..................6
2-(4-methoxybenzyl)-2H-benzo[e][1,2,3,4]thiatriazine 1,1-dioxide, 1d.....................6
2-(4-(trifluoromethyl)benzyl)-2H-benzo[e][1,2,3,4]thiatriazine 1,1-dioxide, 1e 6
2-(4-fluorobenzyl)-2H-benzo[e][1,2,3,4]thiatriazine 1,1-dioxide, 1f 7
2-(4-chlorobenzyl)-2H-benzo[e][1,2,3,4]thiatriazine 1,1-dioxide, 1g 7
4-((1,1-dioxido-2H-benzo[e][1,2,3,4]thiatriazin-2-yl)methyl)benzonitrile, 1h 7
methyl4-((1,1-dioxido-2H-benzo[e][1,2,3,4]thiatriazin-2-yl)methyl)benzoate, 1i.......7
2-(3-(trifluoromethyl)benzyl)-2H-benzo[e][1,2,3,4]thiatriazine 1,1-dioxide, 1j 8
2-(3-fluorobenzyl)-2H-benzo[e][1,2,3,4]thiatriazine 1,1-dioxide, 1k..........................8
2-(3-(trifluoromethoxy)benzyl)-2H-benzo[e][1,2,3,4]thiatriazine 1,1-dioxide，1l 8
2-(2-(trifluoromethyl)benzyl)-2H-benzo[e][1,2,3,4]thiatriazine 1,1-dioxide 1m 9
2-(2-chlorobenzyl)-2H-benzo[e][1,2,3,4]thiatriazine 1,1-dioxide, 1n..........................9
2-(2-methoxybenzyl)-2H-benzo[e][1,2,3,4]thiatriazine 1,1-dioxide，1o...................9
2-(2-phenylpropan-2-yl)-2H-benzo[e][1,2,3,4]thiatriazine 1,1-dioxide, 1p................9
2-benzyl-6-methyl-2H-benzo[e][1,2,3,4]thiatriazine 1,1-dioxide, 1q.......................10
6-methyl-2-(4-methylbenzyl)-2H-benzo[e][1,2,3,4]thiatriazine 1,1-dioxide, 1r 10
2-benzyl-6-methoxy-2H-benzo[e][1,2,3,4]thiatriazine 1,1-dioxide, 1s 10
2-(3,4-dichlorobenzyl)-6-methoxy-2H-benzo[e][1,2,3,4]thiatriazine 1,1-dioxide, 1t10
2-benzyl-6-chloro-2H-benzo[e][1,2,3,4]thiatriazine 1,1-dioxide, 1u........................11
6-chloro-2-(4-fluorobenzyl)-2H-benzo[e][1,2,3,4]thiatriazine 1,1-dioxide, 1v..........11
2-(1,2,3,4-tetrahydronaphthalen-2-yl)-2H-benzo[e][1,2,3,4]thiatriazine 1,1-dioxide, 1w...11
2-(pyridin-2-ylmethyl)-2H-benzo[e][1,2,3,4]thiatriazine 1,1-dioxide, 1x 11
6,7-dihydrodibenzo[d,f][1,2]thiazepine 5,5-dioxide, 2a 12
10-methyl-6,7-dihydrodibenzo[d,f][1,2]thiazepine 5,5-dioxide, 2b 12
10-(tert-butyl)-6,7-dihydrodibenzo[d,f][1,2]thiazepine 5,5-dioxide, 2c 12
10-methoxy-6,7-dihydrodibenzo[d,f][1,2]thiazepine 5,5-dioxide, 2d 13
10-(trifluoromethyl)-6,7-dihydrodibenzo[d,f][1,2]thiazepine 5,5-dioxide, 2e...........13
10-fluoro-6,7-dihydrodibenzo[d,f][1,2]thiazepine 5,5-dioxide, 2f............................13
10-chloro-6,7-dihydrodibenzo[d,f][1,2]thiazepine 5,5-dioxide, 2g 14
6,7-dihydrodibenzo[d,f][1,2]thiazepine-10-carbonitrile 5,5-dioxide, 2h...................14
methyl 6,7-dihydrodibenzo[d,f][1,2]thiazepine-10-carboxylate 5,5-dioxide, 2i 14
9-(trifluoromethyl)-6,7-dihydrodibenzo[d,f][1,2]thiazepine 5,5-dioxide. 2j.............14
9-fluoro-6,7-dihydrodibenzo[d,f][1,2]thiazepine 5,5-dioxide, 2k 15

9-(trifluoromethoxy)-6,7-dihydrodibenzo[d,f][1,2]thiazepine 5,5-dioxide, 2l 15
8-(trifluoromethyl)-6,7-dihydrodibenzo[d,f][1,2]thiazepine 5,5-dioxide, 2m 15
8-chloro-6,7-dihydrodibenzo[d,f][1,2]thiazepine 5,5-dioxide, 2n 16
8-methoxy-6,7-dihydrodibenzo[d,f][1,2]thiazepine 5,5-dioxide, 2o 16
7,7-dimethyl-6,7-dihydrodibenzo[d,f][1,2]thiazepine 5,5-dioxide, 2p 16
2-methyl-6,7-dihydrodibenzo[d,f][1,2]thiazepine 5,5-dioxide, 2q 16
2,10-dimethyl-6,7-dihydrodibenzo[d,f][1,2]thiazepine 5,5-dioxide, 2r 17
2-methoxy-6,7-dihydrodibenzo[d,f][1,2]thiazepine 5,5-dioxide, 2s 17
9,10-dichloro-2-methoxy-6,7-dihydrodibenzo[d,f][1,2]thiazepine 5,5-dioxide, 2t ...17
2-chloro-6,7-dihydrodibenzo[d,f][1,2]thiazepine 5,5-dioxide, 2u 18
2-chloro-10-fluoro-6,7-dihydrodibenzo[d,f][1,2]thiazepine 5,5-dioxide, 2v 18
2,3,3a,4-tetrahydro-1H-benzo[f]naphtho[1,8-cd][1,2]thiazepine 5,5-dioxide, 2w...18
5,6-dihydrobenzo[f]pyrido[2,3-d][1,2]thiazepine 7,7-dioxide, 2x 19
ORTEP and x-ray data of 2p (ccdc-2491640) 20
ORTEP and x-ray data of 5 (ccdc-2491641) 33
^{1}H NMR of 1a 8
^{13}C NMR of 1a 9
^{1}H NMR of 1b 10
^{13}C NMR of 1b 11
^{1}H NMR of 1c 12
^{13}C NMR of 1c 13
^{1}H NMR of 1d 14
^{13}C NMR of 1d 15
^{1}H NMR of 1e 17
^{13}C NMR of 1e 18
^{1}H NMR of 1f 19
^{13}C NMR of 1f 20
^{1}H NMR of 1g 21
^{13}C NMR of 1g 21
^{1}H NMR of 1h 23
^{13}C NMR of 1h 24
^{1}H NMR of 1i 25
^{13}C NMR of 1i 26
^{1}H NMR of 1j 27
^{13}C NMR of 1j 28
^{1}H NMR of 1k 29
^{1}H NMR of 1l 32
^{13}C NMR of 1n 37
^{1}H NMR of 1o 38
^{13}C NMR of 1o 39
^{1}H NMR of 1p 41
^{13}C NMR of 1p 42
^{1}H NMR of 1q 43
^{13}C NMR of 1q 45

^{1}H NMR of 1r 46
^{13}C NMR of 1r 47
^{1}H NMR of 1s 48
^{13}C NMR of 1s 49
^{1}H NMR of 1t 51
^{13}C NMR of 1t 53
^{1}H NMR of 1u 54
^{13}C NMR of 1u 55
^{1}H NMR of 1v 56
^{13}C NMR of 1v 58
^{1}H NMR of 1w 59
^{13}C NMR of 1w 60
^{1}H NMR of 1x 61
^{13}C NMR of 1x 62
^{1}H NMR of 2a 63
^{1}H NMR of 2b 65
^{13}C NMR of 2b 67
^{1}H NMR of 2c 69
^{13}C NMR of 2c 71
^{1}H NMR of 2d 73
^{13}C NMR of 2d 74
^{1}H NMR of 2e 75
^{13}C NMR of 2e 77
^{1}H NMR of 2f 79
^{13}C NMR of 2f 81
^{1}H NMR of 2g 83
^{13}C NMR of 2g 85
^{1}H NMR of 2h 86
^{13}C NMR of 2h 88
^{1}H NMR of 2i 90
^{13}C NMR of 2i 92
^{1}H NMR of 2j 94
^{13}C NMR of 2j 96
^{1}H NMR of 2k 98
^{13}C NMR of 2k 100
^{1}H NMR of 2l 102
^{13}C NMR of 2l 104
^{1}H NMR of 2m 106
^{13}C NMR of 2m 107
^{1}H NMR of 2n 108
^{13}C NMR of 2n 109
^{1}H NMR of 2o 111
^{13}C NMR of 2o 113
^{1}H NMR of 2p 115

^{13}C NMR of 2p....116
^{1}H NMR of 2q....118
^{13}C NMR of 2q....119
^{1}H NMR of 2r....120
^{13}C NMR of 2r....121
^{1}H NMR of 2s....122
^{13}C NMR of 2s....123
^{1}H NMR of 2t....124
^{13}C NMR of 2t....125
^{1}H NMR of 2u....126
^{13}C NMR of 2u....127
^{1}H NMR of 2v....128
^{13}C NMR of 2v....129
^{1}H NMR of 2w....130
^{13}C NMR of 2w....132
^{1}H NMR of 2x....134
^{13}C NMR of 2x....135

Experimental

Unless otherwise noted, all the reagents were obtained from commercial sources and used without further purification. All reactions were performed under a nitrogen atmosphere in anhydrous solvents which were dried prior to use following standard procedures. Reactions were monitored with thin-layer chromatography carried out on 0.25 mm E. Merck silica gel plates (60F-254) using 7% ethanolic phosphomolybdic acid as developing agent. Standard column chromatography was performed using 230–400 mesh silica gel obtained from E. Merck. All NMR spectra were run at 400 MHz (^{1}H NMR), 100 MHz (^{13}C NMR) in $CDCl_3$ solution and chemical shifts are reported in δ (ppm) using solvent resonance as the internal reference.

Genral procedure I: Preparation of substituted 2-benzyl-2*H*-benzo[*e*][1,2,3,4]thiatriazine 1,1-dioxide (1a-1x)

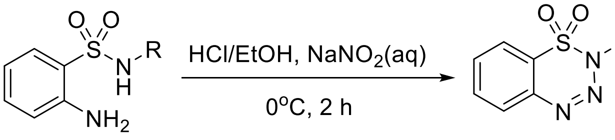

S1

To a solution of above amine in EtOH (20 mL) and 2N HCl(aq) (12 mL) was slowly added a solution of $NaNO_2$ (467 mg, 6.8 mmol) in H_2O (4 mL) at 0 °C. After stirring at 0 °C for 2 h, the reaction mixture was diluted with H_2O (20 mL) and extracted with CH_2Cl_2 (3 x 20 mL). The combined organic layers were dried over $MgSO_4$. The solvents were removed under reduced pressure and the residue was purified through flash chromatography by using hexane and ethyl acetate as the eluents to give the desired substrate **(1a-1x**).

General procedure II: Synthesis of seven-membered sultams (2a-2x)

100 mg of benzothiatriazines in 25 mL two neck flask fitted with condenser. anhydrous acetonitrile (0.1 M) was added. Reflux the reaction mixture for 18 hours. The mixture was concentrated and the residue was purified through a column chromatography by using hexane and ethyl acetate as eluent to afford the desired products (**2a-2x**).

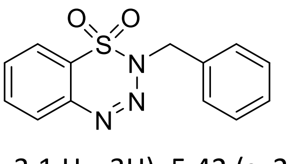

2-benzyl-2l4-benzo[e][1,2,3,4]thiatriazine 1,1-dioxide, 1a

^{1}H NMR (400 MHz, $CDCl_3$) δ 8.03 (m, *J* = 1.9 Hz, 2H), 7.88 (m, *J* = 3.4 Hz, 1H), 7.78 (m, *J* = 3.3 Hz, 1H), 7.48 (m, *J* = 2.4 Hz, 2H), 7.35 (m, *J* = 3.1 Hz, 3H), 5.42 (s, 2H)； ^{13}C NMR (101 MHz, $CDCl_3$) δ 134.1 , 132.6, 129.4, 128.9, 128.8, 128.5, 126.0, 120.5, 51.2； HRMS (EI) calcd. for $C_{13}H_{11}N_3O_2S$ 273.0572, found 273.0568.

2-(4-methylbenzyl)-2H-benzo[e][1,2,3,4]thiatriazine 1,1-dioxide, 1b

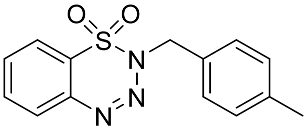

^{1}H NMR (400 MHz, $CDCl_3$) δ 8.02 (m, *J* = 1.4 Hz, 2H), 7.87 (m, *J* = 2.4 Hz, 1H), 7.76 (m, *J* = 2.8 Hz, 1H), 7.38 (d, *J* = 8.0 Hz, 2H), 7.16 (d, *J* = 7.9 Hz, 2H), 5.38 (s, 2H), 2.33 (s, 3H)； ^{13}C NMR (101 MHz, $CDCl_3$) δ 138.4, 134.0, 132.6, 132.3, 129.5, 129.4, 128.9, 120.5, 51.1, 21.2； **HRMS** (EI) calcd. for $C_{14}H_{13}N_3O_2S$ 287.0728, found 287.0722.

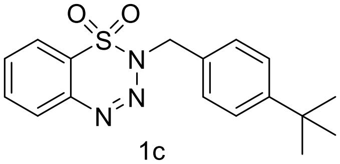

1c

2-(4-(tert-butyl)benzyl)-2H-benzo[e][1,2,3,4]thiatriazine 1,1-dioxide, 1c

^{1}H NMR (400 MHz, $CDCl_3$) δ 8.02 (m, *J* = 2.7 Hz, 2H), 7.87 (m, *J* = 3.4 Hz, 1H), 7.77 (m, *J* = 3.3 Hz, 1H), 7.39 (m, *J* = 4.4 Hz, 4H), 5.39 (s, 2H), 1.29 (s, 9H)； ^{13}C NMR (101 MHz, $CDCl_3$) δ 151.5, 141.7, 134.0, 132.6, 132.2, 129.4, 128.6, 127.8, 125.7, 120.5, 50.8, 34.6, 31.3； **HRMS** (EI) calcd. for $C_{17}H_{19}N_3O_2S$ 329.1198, found 329.1193.

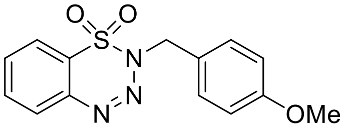

2-(4-methoxybenzyl)-2H-benzo[e][1,2,3,4]thiatriazine 1,1-dioxide, 1d

^{1}H NMR (600 MHz, $CDCl_3$): δ 8.00 (t, *J* = 7.8 Hz, 2H), 7.85 (t, *J* = 7.8 Hz, 1H), 7.75 (t, *J* = 7.8 Hz, 1H), 7.42 (d, *J* = 8.4 Hz, 2H), 6.87 (d, *J* = 9.0 Hz, 2H), 5.35 (s, 2H), 3.78 (s, 3H)； ^{13}C NMR (150 MHz, $CDCl_3$): δ 159.7, 141.6, 133.9, 132.5, 130.3, 129.2, 127.3, 125.8, 120.3, 114.1, 55.2, 50.8； **HRMS** (ESI) calcd. for $C_{14}H_{13}N_3O_3S$ 303.0678, found 303.0674.

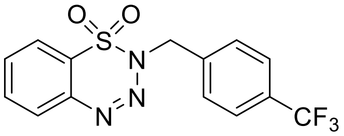

2-(4-(trifluoromethyl)benzyl)-2H-benzo[e][1,2,3,4]thiatriazine 1,1-dioxide, 1e

^{1}H NMR (400 MHz, $CDCl_3$) δ 8.05 (d, *J* = 8.2 Hz, 2H), 7.90 (m, *J* = 3.4 Hz, 1H), 7.80 (m, *J* = 3.3 Hz, 1H), 7.62 (t, *J* = 6.5 Hz, 4H), 5.46 (s, 2H)； ^{13}C NMR (101 MHz, $CDCl_3$) δ 141.6, 139.1, 134.3, 132.9, 129.6, 129.1, 126.0, 125.8 (q, *J* = 3.8 Hz, 1C), 122.6, 120.5, 50.5； **HRMS** (ESI) calcd. for $C_{14}H_{10}N_3O_2S$ 341.0446, found 341.0434.

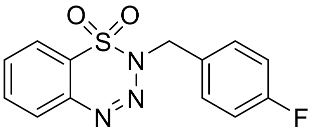

2-(4-fluorobenzyl)-2H-benzo[e][1,2,3,4]thiatriazine 1,1-dioxide, 1f

^{1}H NMR (400 MHz, $CDCl_3$) δ 8.03 (d, *J* = 8.1 Hz, 2H), 7.88 (m, *J* = 3.4 Hz, 1H), 7.78 (m, *J* = 3.3 Hz, 1H), 7.47 (m, *J* = 2.8 Hz, 2H), 7.04 (q, *J* = 5.8 Hz, 2H), 5.38 (s, 2H) ; ^{13}C NMR (101 MHz, $CDCl_3$) δ 164.1, 161.6, 141.6, 134.2, 132.7, 130.9, 130.8, 129.5, 125.9, 120.5, 115.9, 115.7, 50.5. ; **HRMS** (ESI) calcd. for $C_{13}H_{10}FN_3O_2S$ 291.0478, found 291.0479.

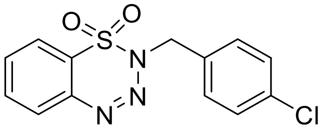

2-(4-chlorobenzyl)-2H-benzo[e][1,2,3,4]thiatriazine 1,1-dioxide, 1g

^{1}H NMR (400 MHz, $CDCl_3$) δ 8.03 (m, *J* = 3.0 Hz, 2H), 7.88 (t, *J* = 3.4 Hz, 1H), 7.78 (t, *J* = 5.4 Hz, 1H), 7.37 (q, *J* = 15.7 Hz, 4H), 5.36 (s, 2H). ; ^{13}C NMR (101 MHz, $CDCl_3$) δ 146.6, 141.3, 134.3, 133.0, 132.3, 129.6, 129.0, 128.1, 126.5, 121.0, 29.7, 28.7, 15.4 ; **HRMS** (EI) calcd. for $C_{13}H_{10}ClN_3O_2S$ 307.0182, found 307.0182.

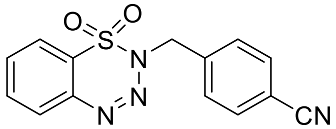

4-((1,1-dioxido-2H-benzo[e][1,2,3,4]thiatriazin-2-yl)methyl)benzonitrile, 1h

^{1}H NMR (400 MHz, $CDCl_3$) δ 8.05 (m, *J* = 1.9 Hz, 2H), 7.92 (td, *J* = 3.4 Hz, 1H), 7.81 (td, *J* = 3.3 Hz, 1H), 7.66 (d, *J* = 2.8 Hz, 2H), 7.58 (d, *J* = 8.4 Hz, 2H), 5.44 (s, 2H). ; ^{13}C NMR (101 MHz, $CDCl_3$) δ 141.5, 140.4, 134.4, 133.1, 132.6, 129.7, 129.4, 126.0, 120.6, 118.4, 112.5, 50.4, 29.7, 14.2.; **HRMS** (EI) calcd. for $C_{14}H_{10}N_4O_2S$ 298.0524, found 298.0514.

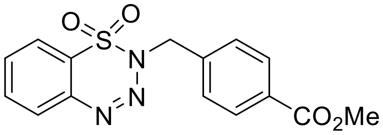

methyl4-((1,1-dioxido-2H-benzo[e][1,2,3,4]thiatriazin-2-yl)methyl)benzoate, 1i

^{1}H NMR (400 MHz, $CDCl_3$) δ 8.03 (m, *J* = 3.9 Hz, 4H), 7.90 (td, *J* = 3.4 Hz, 1H), 7.79 (td, *J* = 3.3 Hz, 1H), 7.54 (d, *J* = 8.3 Hz, 2H), 5.45 (s, 2H), 3.90 (s, 3H).; ^{13}C NMR (101 MHz, $CDCl_3$) δ 166.6, 141.6, 140.1, 134.2, 132.9, 130.3, 130.1,

129.6, 128.7, 126.0, 120.5, 52.2, 50.7, 29.7.; **HRMS** (EI) calcd. for $C_{15}H_{13}N_3O_4S$ 331.0627, found 331.0614.

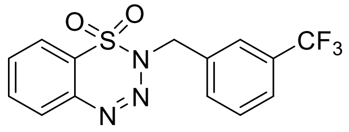

2-(3-(trifluoromethyl)benzyl)-2H-benzo[e][1,2,3,4]thiatriazine 1,1-dioxide, 1j

^{1}H NMR (400 MHz, CDCl$_3$) δ 8.07 (m, *J* = 4.0 Hz, 2H), 7.92 (m, *J* = 2.8 Hz, 1H), 7.82 (td, *J* = 3.3 Hz, 1H), 7.76 (s, 1H), 7.70 (d, *J* = 7.7 Hz, 1H), 7.62 (d, *J* = 7.8 Hz, 1H), 7.52 (t, *J* = 15.5 Hz, 1H), 5.48 (s, 2H).; ^{13}C NMR (101 MHz, CDCl$_3$) δ 141.5, 136.2, 134.3, 132.9, 132.2, 131.4, 131.1, 129.6, 129.4, 126.0, 125.7 (q, *J* = 3.8 Hz, 1C), 125.4 (q, *J* = 3.8 Hz, 1C), 120.6, 50.5. **HRMS** (EI) calcd. for $C_{14}H_{10}F_3N_3O_2S$ 341.0446, found 341.043.

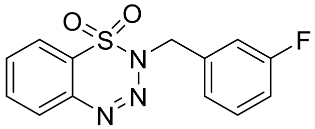

2-(3-fluorobenzyl)-2H-benzo[e][1,2,3,4]thiatriazine 1,1-dioxide, 1k

^{1}H NMR (400 MHz, CDCl$_3$) δ 8.07 (m, *J* = 4.0 Hz, 2H), 7.92 (td, *J* = 4.2 Hz, 1H), 7.82 (td, *J* = 4.1 Hz, 1H), 7.35 (m, *J* = 4.3 Hz, 1H), 7.28 (m, *J* = 3.8 Hz, 1H), 7.20 (d, *J* = 9.5 Hz, 1H), 7.04 (td, *J* = 3.7 Hz, 1H), 5.42 (s, 2H)..; ^{13}C NMR (101 MHz, CDCl$_3$) δ 134.2, 132.8, 130.4, 130.3, 129.5, 124.4, 124.4, 120.5, 115.9, 115.6, 115.4, 50.5, 50.4.; **HRMS** (EI) calcd. for $C_{13}H_{10}FN_3O_2S$ 291.0478, found 291.0479.

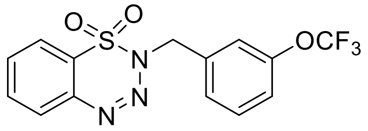

2-(3-(trifluoromethoxy)benzyl)-2H-benzo[e][1,2,3,4]thiatriazine 1,1-dioxide，1l

^{1}H NMR (400 MHz, CDCl$_3$) δ 8.05 (m, *J* = 4.5 Hz, 2H), 7.90 (td, *J* = 3.4 Hz, 1H), 7.80 (m, *J* = 3.9 Hz, 1H), 7.39 (m, *J* = 7.6 Hz, 2H), 7.33 (s, 1H), 7.19 (d, *J* = 7.4 Hz, 1H), 5.41 (s, 2H).; ^{13}C NMR (101 MHz, CDCl$_3$) δ 149.4, 141.6, 137.5, 134.3, 132.9, 130.2, 129.6, 127.1, 126.0, 121.4, 120.9, 120.5, 119.1, 50.4. **HRMS** (EI) calcd. for $C_{14}H_{10}F_3N_3O_3S$ 357.0395, found 357.0403

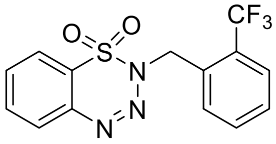

2-(2-(trifluoromethyl)benzyl)-2H-benzo[e][1,2,3,4]thiatriazine 1,1-dioxide 1m

^{1}H NMR (400 MHz, $CDCl_3$) δ 8.05 (m, *J* = 8.3 Hz, 2H), 7.90 (td, *J* = 3.4 Hz, 1H), 7.80 (td, *J* = 3.3 Hz, 1H), 7.61 (m, *J* = 10.8 Hz, 4H), 5.46 (s, 2H).; ^{13}C NMR (101 MHz, $CDCl_3$) δ 141.6, 139.1, 134.3, 132.9, 129.6, 129.1, 126.0, 125.8, 120.5, 50.5; **HRMS** (EI) calcd. for $C_{14}H_{10}F_3N_3O_2S$ 341.0446, found 341.043

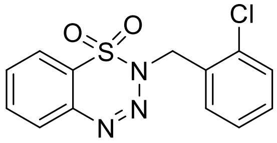

2-(2-chlorobenzyl)-2H-benzo[e][1,2,3,4]thiatriazine 1,1-dioxide, 1n

^{1}H NMR (400 MHz, $CDCl_3$) δ 8.03 (d, *J* = 3.1 Hz, 2H), 7.89 (td, *J* = 2.8 Hz, 1H), 7.79 (td, *J* = 4.1 Hz, 1H), 7.42 (d, *J* = 8.6 Hz, 2H), 7.33 (d, *J* = 8.5 Hz, 2H), 5.37 (s, 2H).; **^{13}C NMR** (101 MHz, $CDCl_3$) δ 135.2, 134.2, 134.1, 132.6, 130.3, 129.4, 128.9, 128.8, 128.5, 127.1, 126.0, 120.5, 120.5.; **HRMS** (EI) calcd. for $C_{13}H_{10}ClN_3O_2S$ 307.0182, found 307.0174.

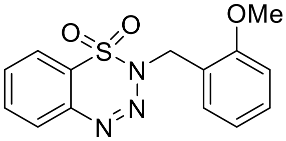

2-(2-methoxybenzyl)-2H-benzo[e][1,2,3,4]thiatriazine 1,1-dioxide，1o

^{1}H NMR (400 MHz, $CDCl_3$) δ 8.02 (m, *J* = 2.3 Hz, 2H), 7.87 (td, *J* = 5.6 Hz, 1H), 7.76 (td, *J* = 3.3 Hz, 1H), 7.32 (m, *J* = 8.2 Hz, 1H), 7.28 (d, *J* = 1.7 Hz, 1H), 6.92 (q, *J* = 5.7 Hz, 2H), 5.50 (s, 2H), 3.87 (s, 3H)； ^{13}C NMR (101 MHz, $CDCl_3$) δ 157.5 , 141.6, 134.0, 132.5, 130.0, 129.7, 129.3, 126.0, 123.5, 120.5, 120.4, 110.6, 55.5, 46.2； **HRMS** (EI) calcd. for $C_{14}H_{13}N_3O_3S$ 303.0678, found 303.0669.

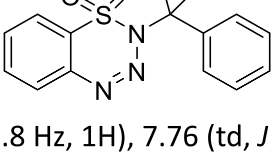

2-(2-phenylpropan-2-yl)-2H-benzo[e][1,2,3,4]thiatriazine 1,1-dioxide, 1p

^{1}H NMR (400 MHz, $CDCl_3$) δ 8.00 (m, *J* = 3.2 Hz, 2H), 7.88 (td, *J* = 2.8 Hz, 1H), 7.76 (td, *J* = 3.3 Hz, 1H), 7.45 (m, *J* = 8.1 Hz, 2H), 7.36 (m, *J* = 5.0 Hz, 2H), 7.29 (m, *J* = 2.6 Hz, 1H), 2.23 (s, 6H).; ^{13}C NMR (101 MHz, $CDCl_3$) δ 145.4, 141.0, 133.9, 132.3, 128.7, 128.4, 128.0, 127.5, 126.5, 125.5, 120.3, 70.8, 30.2, 29.8, 29.7. ; **HRMS** (EI) calcd.

For $C_{15}H_{15}N_3O_2S$ 301.0885, found 301.0885.

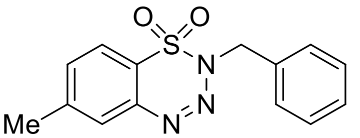

2-benzyl-6-methyl-2H-benzo[e][1,2,3,4]thiatriazine 1,1-dioxide, 1q

^{1}H NMR (600 MHz, CDCl$_3$): δ 7.91 (d, *J* = 8.1 Hz, 1H), 7.80 (s, 1H), 7.56 (d, *J* = 8.1 Hz, 1H), 7.47 (d, *J* = 6.9 Hz, 2H), 7.36-7.30 (m, 3H), 5.39 (s, 2H), 2.55 (s, 3H).; ^{13}C NMR (150 MHz, CDCl$_3$): δ 145.4, 141.7, 135.3, 133.4, 129.3, 128.8, 128.7, 128.3, 123.3, 120.3, 50.9, 21.6; **HRMS** (ESI) calcd. for $C_{14}H_{13}N_3O_2S$ 287.0728, found 287.0727.

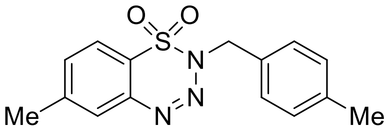

6-methyl-2-(4-methylbenzyl)-2H-benzo[e][1,2,3,4]thiatriazine 1,1-dioxide, 1r

^{1}H NMR (600 MHz, CDCl$_3$): δ 7.90 (d, *J* = 8.4 Hz, 1H), 7.79 (s, 1H), 7.65 (d, *J* = 8.4 Hz, 1H), 7.36 (d, *J* = 7.8 Hz, 2H), 7.15 (d, *J* = 7.8 Hz, 2H), 5.35 (s, 2H), 2.54 (s, 3H), 2.32 (s, 3H); ^{13}C NMR (150 MHz, CDCl$_3$): δ 145.3, 141.7, 138.2, 133.4, 132.3, 129.4, 129.2, 128.8, 123.2, 120.3, 50.8, 21.6, 21.1; **HRMS** (EI) calcd. for $C_{15}H_{15}N_3O_2S$ 301.0885, found 301.0885.

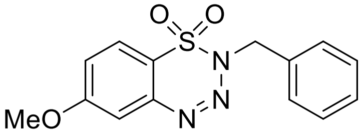

2-benzyl-6-methoxy-2H-benzo[e][1,2,3,4]thiatriazine 1,1-dioxide, 1s

^{1}H NMR (600 MHz, CDCl$_3$): δ 7.92 (d, *J* = 8.7 Hz, 1H), 7.47 (d, *J* = 7.8 Hz, 2H), 7.41 (s, 1H), 7.34 (t, *J* = 7.2 Hz, 2H), 7.32 (d, *J* = 7.2 Hz, 1H), 7.27-7.25 (m, 1H), 5.39 (s, 2H), 3.94 (s, 3H); ^{13}C NMR (150 MHz, CDCl$_3$): δ 163.7, 143.6, 135.2, 128.7(2C), 128.3, 122.2, 120.4, 118.2, 111.7, 56.1, 50.9; **HRMS** (EI) calcd. for $C_{14}H_{13}N_3O_3S$ 303.0678, found 303.0681.

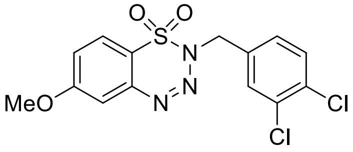

2-(3,4-dichlorobenzyl)-6-methoxy-2H-benzo[e][1,2,3,4]thiatriazine 1,1-dioxide, 1t

^{1}H NMR (600 MHz, CDCl$_3$): δ 7.92 (d, *J* = 9.0 Hz, 1H), 7.56 (sd, *J* = 1.8 Hz, 1H), 7.42 (d, *J* = 10.2 Hz, 2H), 7.31 (dd, *J* = 8.4, 2.4 Hz, 1H), 7.28 (dd, *J* = 8.4, 2.4 Hz, 1H), 5.31 (s, 2H), 3.96 (s, 3H); ^{13}C NMR (150 MHz, CDCl$_3$): δ 163.9, 143.5, 135.4, 132.9, 132.8, 130.7, 128.1, 122.3, 120.6, 118.2, 112.0, 56.1, 49.7; **HRMS** (ESI) calcd. for $C_{14}H_{11}Cl_2N_3O_3S$ 370.9898, found 370.9894.

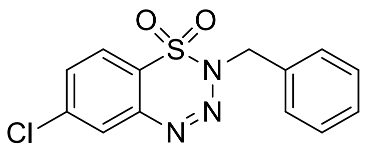

2-benzyl-6-chloro-2H-benzo[e][1,2,3,4]thiatriazine 1,1-dioxide, 1u

^{1}H NMR (600 MHz, $CDCl_3$): δ 8.00 (s, 1H), 7.95 (d, *J* = 8.4 Hz, 1H) , 7.71 (d, *J* = 9.0 Hz, 1H), 7.47 (d, *J* = 7.2 Hz, 2H), 7.37-7.32 (m, 3H), 5.41 (s, 2H); ^{13}C NMR (150 MHz, $CDCl_3$): δ 142.4, 140.4, 134.9, 132.7, 129.0, 128.8, 128.5, 124.1, 122.0, 51.3; **HRMS** (ESI) calcd. for $C_{13}H_{10}ClN_3O_2S$ 307.0182, found 307.0179.

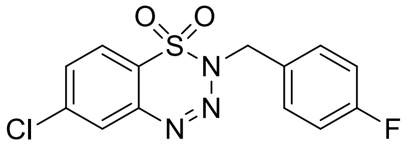

6-chloro-2-(4-fluorobenzyl)-2H-benzo[e][1,2,3,4]thiatriazine 1,1-dioxide, 1v

^{1}H NMR (600 MHz, $CDCl_3$): δ 8.00 (sd, *J* = 1.2 Hz, 1H), 7.95 (d, *J* = 8.4 Hz, 1H), 7.73 (dd, *J* = 8.4, 1.8 Hz, 1H), 7.47–7.44 (m, 2H), 7.04 (t, *J* = 8.4 Hz, 2H), 5.37 (s, 2H); ^{13}C NMR (150 MHz, $CDCl_3$): δ 163.7, 162.0, 142.4, 140.6, 132.8, 130.8(2C), 129.1, 124.1, 122.0, 115.8, 115.7, 50.7; **HRMS** (ESI) calcd. for $C_{13}H_9ClFN_3O_2S$ 325.0087, found 325.0088.

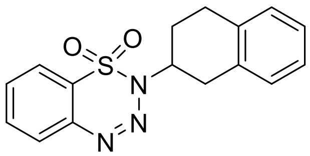

2-(1,2,3,4-tetrahydronaphthalen-2-yl)-2H-benzo[e][1,2,3,4]thiatriazine 1,1-dioxide, 1w

^{1}H NMR (400 MHz, $CDCl_3$) δ 8.09 (m, *J* = 3.0 Hz, 1H), 8.01 (m, *J* = 3.0 Hz, 1H), 7.88 (td, *J* = 3.4 Hz, 1H), 7.80 (td, *J* = 3.3 Hz, 1H), 7.15 (m, *J* = 3.9 Hz, 4H), 5.94 (t, *J* = 7.3 Hz, 1H), 3.00 (m, *J* = 5.2 Hz, 1H), 2.85 (m, *J* = 5.4 Hz, 1H), 2.52 (m, *J* = 2.6 Hz, 1H), 2.18 (m, *J* = 2.6 Hz, 1H), 1.95 (m, *J* = 4.2 Hz, 1H).; ^{13}C NMR (101 MHz, $CDCl_3$) δ 141.4, 138.9, 134.1, 133.8, 132.5, 129.4, 129.3, 128.3, 127.6, 126.4, 125.3, 120.6, 56.5, 32.1, 29.2, 21.3. ; **HRMS** (EI) calcd. for $C_{16}H_{15}N_3O_2S$ 313.0885, found 313.0885.

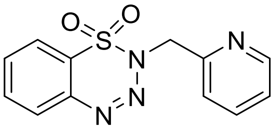

2-(pyridin-2-ylmethyl)-2H-benzo[e][1,2,3,4]thiatriazine 1,1-dioxide, 1x

^{1}H NMR (400 MHz, $CDCl_3$) δ 8.61 (d, *J* = 4.9 Hz, 1H), 8.06 (m, *J* = 1.8 Hz, 2H), 7.90 (td, *J* = 3.4 Hz, 1H), 7.80 (td, *J* = 3.3 Hz, 1H), 7.69 (td, *J* = 3.4 Hz, 1H), 7.40 (d, *J* = 7.9 Hz, 1H), 7.24 (dd, *J* = 4.1 Hz, 1H), 5.57 (s, 2H).; ^{13}C NMR (101 MHz, $CDCl_3$) δ 149.7, 137.0, 134.2, 132.8, 129.6, 129.1, 126.1, 123.1, 122.4, 120.6, 52.5.; **HRMS** (EI) calcd. for $C_{12}H_{10}N_4O_2S$ 274.0524, found 274.0514.

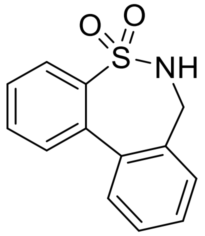

6,7-dihydrodibenzo[d,f][1,2]thiazepine 5,5-dioxide, 2a

^{1}H NMR (400 MHz, $CDCl_3$) δ 8.05 (d, *J* = 6.6 Hz, 1H), 7.75 (d, *J* = 1.4 Hz, 1H), 7.73 (d, *J* = 1.3 Hz, 2H), 7.71 (d, *J* = 1.3 Hz, 1H), 7.61 -7.45 (m, 6H), 5.14 (s, 1H), 4.08 (d, *J* = 1.6 Hz, 2H); **^{13}C NMR** (101 MHz, $CDCl_3$) δ 140.9, 139.0, 137.2, 133.3, 132.5, 130.3, 130.2, 129.7, 129.22, 129.0, 128.5, 126.3, 47.6；**HRMS** [(ESI), $(M+Na)^+$]: 268.0409. (calcd. $C_{13}H_{11}NNaO_2S$ 268.0408).

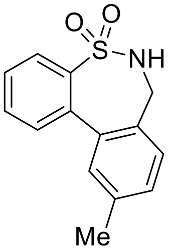

10-methyl-6,7-dihydrodibenzo[d,f][1,2]thiazepine 5,5-dioxide, 2b

^{1}H NMR (400 MHz, $CDCl_3$) δ 8.05 (q, *J* = 3.0 Hz, 1H), 7.72 (q, *J* = 2.9 Hz, 1H), 7.60 (m, *J* = 4.4 Hz, 2H), 7.31 (m, *J* = 8.6 Hz, 3H), 5.25 (N-H) (s, 1H), 4.06 benzylic C-H (d, *J* = 2.0 Hz, 2H), 2.45 (s, 3H); **^{13}C NMR** (101 MHz, $CDCl_3$) δ 140.7, 139.5, 139.2, 137.3, 133.2, 130.3, 130.1, 129.9, 129.8, 129.6, 128.4, 126.2, 47.2, 21.3；**HRMS** [(ESI), $(M+Na)^+$]: 282.0566. (calculated $C_{13}H_{11}NNaO_2S$ 282.0565).

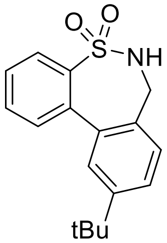

10-(tert-butyl)-6,7-dihydrodibenzo[d,f][1,2]thiazepine 5,5-dioxide, 2c

^{1}H NMR (400 MHz, $CDCl_3$) δ 8.05 (d, *J* = 7.7 Hz, 1H), 7.74 (t, *J* = 7.4 Hz, 1H), 7.63 (d, *J* = 7.5 Hz, 1H), 7.57 (t, *J* = 7.5 Hz, 1H), 7.48 (d, *J* = 7.2 Hz, 2H), 7.38 (d, *J* = 8.0 Hz, 1H), 5.37 (s, 2H), 4.06 (s, 1H), 1.39 (s, 9H); **^{13}C NMR** (101 MHz, $CDCl_3$) δ 152.7, 140.5, 139.5, 137.4, 133.3, 130.3, 130.1, 129.7, 128.3, 126.2, 126.1, 47. 1, 34.8, 31.3；**HRMS** [(ESI), $(M+Na)^+$]: Obs=324.1032. (calculated $C_{17}H_{19}NNaO_2S$ Obs=324.1034).

10-methoxy-6,7-dihydrodibenzo[d,f][1,2]thiazepine 5,5-dioxide, 2d

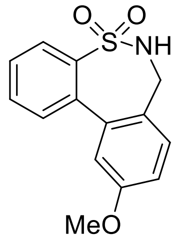

^{1}H NMR (600 MHz, $CDCl_3$): δ 8.05 (dd, *J* = 7.8, 0.9 Hz, 1H), 7.71 (td, *J* = 7.5, 1.2 Hz, 1H), 7.60-7.55 (m, 2H), 7.35 (d, *J* = 8.1 Hz, 1H), 6.99 (sd, *J* = 2.4 Hz, 1H), 6.94 (dd, *J* = 8.1, 2.4 Hz, 1H), 5.01 (s, 1H), 4.03 (sd, *J* = 2.1 Hz, 2H), 3.86 (s, 3H); ^{13}C NMR (150 MHz, $CDCl_3$): δ 160.4, 142.1, 138.9, 137.3, 133.2, 131.5, 130.0, 128.5, 126.2, 124.5, 114.9, 114.0, 55.4, 46.9；HRMS [(ESI), $(M+Na)^+$]: 275.0615 (calculated $C_{14}H_{13}NO_3S$ 275.0616)

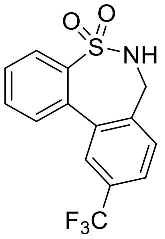

10-(trifluoromethyl)-6,7-dihydrodibenzo[d,f][1,2]thiazepine 5,5-dioxide, 2e

^{1}H NMR (400 MHz, $CDCl_3$) δ 8.10 (dd, *J* = 3.0 Hz, 1H), 7.76 (m, *J* = 3.2 Hz, 2H), 7.73 (s, 1H), 7.68-7.59 (m, *J* = 4.7 Hz, 3H), 5.25 (s, 1H), 4.14 (d, *J* = 1.7 Hz, 2H); **^{13}C NMR** (101 MHz, $CDCl_3$) δ 144.6, 137.5, 133.5, 130.2, 129.4, 127.2, 126.6, 126.4, 47.2；**HRMS** [(ESI), $(M+Na)^+$]: 336.0283. (calculated $C_{14}H_{10}F_3NNaO_2S$ 336.0282).

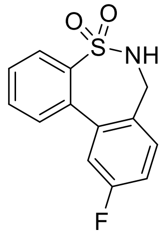

10-fluoro-6,7-dihydrodibenzo[d,f][1,2]thiazepine 5,5-dioxide, 2f

^{1}H NMR (400 MHz, $CDCl_3$) δ 8.08 (d, *J* = 7.8 Hz, 1H), 7.76 (t, *J* = 7.6 Hz, 1H), 7.62 (q, *J* = 7.2 Hz, 2H), 7.44 (q, *J* = 4.6 Hz, 1H), 7.17 (m, *J* = 4.4 Hz, 2H), 5.11(N-H) (s, 1H), 4.07 (s, 2H).; **^{13}C NMR** (101 MHz, $CDCl_3$) δ 164.5, 162.0, 143.0 (d, *J* = 8.3 Hz, 1C), 137.9, 137.3, 133.4, 132.1 (d, *J* = 8.5 Hz, 1C), 130.0 , 129.1, 128.4 (d, *J* = 3.3 Hz, 1C), 126.4, 116.0 (q, *J* = 16.8 Hz, 1C), 46.8；**HRMS** [(ESI), $(M+Na)^+$]: 286.0318. (calculated $C_{13}H_{10}FNNaO_2S$ 286.0314).

10-chloro-6,7-dihydrodibenzo[d,f][1,2]thiazepine 5,5-dioxide, 2g

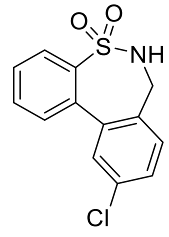

^{1}H NMR (400 MHz, Acetone) δ 7.99 (m, *J* = 1.6 Hz, 1H), 7.83 (m, *J* = 2.5 Hz, 1H), 7.71 (m, *J* = 4.0 Hz, 1H), 7.49 (m, *J* = 4.7 Hz, 1H), 4.07 (d, *J* = 2.7 Hz, 1H)； **HRMS** [(ESI), $(M+Na)^+$]: 302.0015. (calculated $C_{13}H_{10}ClNNaO_2S$=302.0018).

6,7-dihydrodibenzo[d,f][1,2]thiazepine-10-carbonitrile 5,5-dioxide, 2h

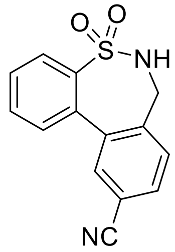

^{1}H NMR (400 MHz, $CDCl_3$) δ 8.10 (q, *J* = 3.0 Hz, 1H), 7.78 (m, *J* = 4.4 Hz, 3H), 7.68 (m, *J* = 3.3 Hz, 1H), 7.61 (m, *J* = 4.4 Hz, 2H), 5.20 (s, 1H), 4.13 (s, 2H); **^{13}C NMR** (101 MHz, $CDCl_3$) δ 142.4, 137.5, 137.4, 136.7, 133.7, 132.6, 132.1, 131.2, 130.0, 129.7, 126.5, 118.0, 113.9, 47.2 (benzylic C)； **HRMS** [(ESI), $(M+Na)^+$]=293.0347, (calculated $C_{14}H_{10}N_2NaO_2S$ 293.0361).

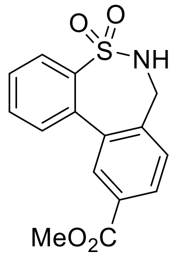

methyl 6,7-dihydrodibenzo[d,f][1,2]thiazepine-10-carboxylate 5,5-dioxide, 2i

^{1}H NMR (400 MHz, $CDCl_3$) δ 8.14 (d, *J* = 1.6 Hz, 1H), 8.08 (m, *J* = 3.7 Hz, 2H), 7.75 (q, *J* = 2.9 Hz, 1H), 7.63 (m, *J* = 4.0 Hz, 2H), 7.53 (d, *J* = 7.8 Hz, 1H), 5.22 (s, 1H), 4.11 (s, 2H), 3.94 (s, 3H); **^{13}C NMR** (101 MHz, $CDCl_3$) δ 166.3, 141.3, 138.0, 137.3, 133.5, 131.5, 130.6, 130.3 (d, *J* = 3.9 Hz), 130.0, 129.0, 126.5, 126.3, 52.4, 47.3; **HRMS** (EI), [M]+: Obs=303.0540. (calculated $C_{15}H_{13}NO_4S$ calc=303.0565).

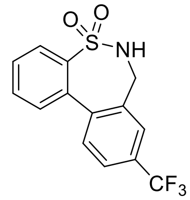

9-(trifluoromethyl)-6,7-dihydrodibenzo[d,f][1,2]thiazepine 5,5-dioxide. 2j

^{1}H NMR (400 MHz, $CDCl_3$) δ 8.10 (dd, *J* = 3.0 Hz, 1H), 7.76 (m, *J* = 3.2 Hz, 2H), 7.73 (s, 1H), 7.68-7.59 (m, *J* = 4.7 Hz, 3H), 5.25 (s, 1H), 4.14 (d, *J* = 1.7 Hz, 2H). **^{13}C NMR** (101 MHz, $CDCl_3$) δ 144.6, 137.5, 133.5, 130.2, 129.4, 127.2, 126.6, 126.4, 47.2 (Benzylic C); **HRMS** [(ESI), $(M+Na)^+$]: 336.0283. (calculated

$C_{14}H_{10}F_3NNaO_2S$ 336.0282).

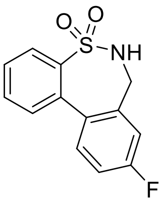

9-fluoro-6,7-dihydrodibenzo[d,f][1,2]thiazepine 5,5-dioxide, 2k

^{1}H NMR (400 MHz, $CDCl_3$) δ 8.07 (q, *J* = 2.9 Hz, 1H), 7.74 (m, *J* = 3.3 Hz, 1H), 7.60 (m, *J* = 3.0 Hz, 1H), 7.46 (q, *J* = 4.6 Hz, 1H), 7.22 (m, *J* = 3.9 Hz, 1H), 5.16 (s, 1H), 4.07 (d, *J* = 2.5 Hz, 1H); **^{13}C NMR** (101 MHz, $CDCl_3$) δ 164.1, 137.0 (d, *J* = 13.9 Hz), 134.6, 133.4, 130.7 (d, *J* = 8.4 Hz), 130.1, 128.6, 126.4, 117.7, 117.5, 116.7, 116.5, 47.2; **HRMS** [(ESI), $(M+Na)^+$]: 286.0311. (calculated $C_{13}H_{10}FNNaO_2S$ 286.0314).

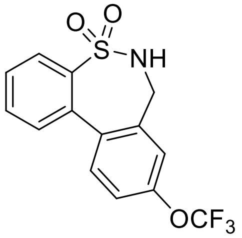

9-(trifluoromethoxy)-6,7-dihydrodibenzo[d,f][1,2]thiazepine 5,5-dioxide, 2l

^{1}H NMR (400 MHz, $CDCl_3$) δ 7.87 (q, *J* = 3.2 Hz, 1H), 7.72 (m, *J* = 4.0 Hz, 1H), 7.62 (m, *J* = 3.9 Hz, 2H), 7.47 (m, *J* = 5.3 Hz, 1H), 5.06 (s, 1H), 4.14 (t, *J* = 7.9 Hz, 2H). ^{13}C NMR (101 MHz, $CDCl_3$) δ 145.7, 137.2, 136.1, 135.2, 134.4, 133.7, 131.6, 130.3 (d, *J* = 2.5 Hz, 1C), 128.8, 124.0, 47.6 (benzylic C); **HRMS** [(ESI), $(M+Na)^+$]: 352.0236 (calculated $C_{14}H_{10}F_3NNaO_3S$ 352.0231). Obtained as a mixture with **S2** (2l : S2 = 1 : 1.11).

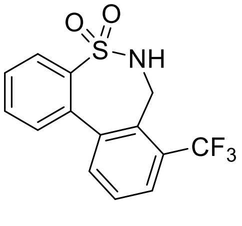

8-(trifluoromethyl)-6,7-dihydrodibenzo[d,f][1,2]thiazepine 5,5-dioxide, 2m

^{1}H NMR (400 MHz, $CDCl_3$) δ 8.09 (d, *J* = 7.8 Hz, 1H), 7.80 (q, *J* = 7.6 Hz, 2H), 7.65 (m, *J* = 4.3 Hz, 4H), 5.15 (s, 1H), 4.24 (s, 1H). **^{13}C NMR** (101 MHz, $CDCl_3$) δ 137.9, 136.5, 133.5, 132.5, 131.0, 130.0, 129.4 (d, *J* = 2.9 Hz, 1C), 126.4 (t, *J* = 11.7 Hz, 1C), 43.0 (d, *J* = 2.3 Hz, 1C) ; **HRMS** [(ESI), $(M+Na)^+$]: 336.0288.

(calculated $C_{14}H_{10}F_3NNaO_2S$ 336.0282).

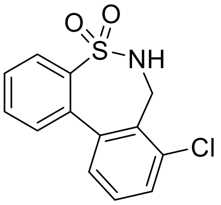

8-chloro-6,7-dihydrodibenzo[d,f][1,2]thiazepine 5,5-dioxide, 2n

^{1}H NMR (400 MHz, DMSO) δ 8.16 (s, 1H), 7.94 (d, *J* = 7.6 Hz, 1H), 7.85 (t, *J* = 7.4 Hz, 1H), 7.73 (t, *J* = 7.1 Hz, 1H), 7.59 (d, *J* = 7.8 Hz, 1H), 7.52 (t, *J* = 7.8 Hz, 1H), 7.46 (d, *J* = 7.3 Hz, 1H), 3.99 (s, 1H). **^{13}C NMR** (101 MHz, DMSO) δ 143.7, 138.1, 137.4 (s, 1C), 133.9, 131.6, 130.8, 130.6, 129.9, 128.2, 125.7, 43.3； **HRMS** [(ESI), $(M+Na)^+$]: 302.0011. (calculated $C_{13}H_{10}ClNNaO_2S$=302.0018).

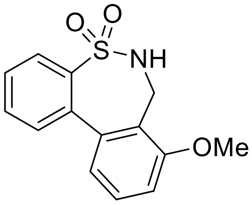

8-methoxy-6,7-dihydrodibenzo[d,f][1,2]thiazepine 5,5-dioxide, 2o

^{1}H NMR (400 MHz, $CDCl_3$) δ 8.08 (d, *J* = 7.8 Hz, 1H), 7.73 (q, *J* = 5.4 Hz, 1H), 7.61 (m, *J* = 6.7 Hz, 1H), 7.48 (t, *J* = 8.0 Hz, 1H), 7.09 (d, *J* = 7.7 Hz, 1H), 7.03 (d, *J* = 8.4 Hz, 1H), 5.07 (s, 1H), 4.21 (s, 1H), 3.93 (s, 1H). **^{13}C NMR** (101 MHz, $CDCl_3$) δ 157.0 (s, 1C), 142.6, 139.1, 136.9, 133.2, 130.1 (d, *J* = 38.2 Hz, 1C), 128.5, 126.3, 120.9, 111.2, 56.0, 39.4 (s, 1C).**;** **HRMS** [(ESI), $(M+Na)^+$]: 298.0511 (calculated $C_{14}H_{13}NNaO_3S$ 298.0514).

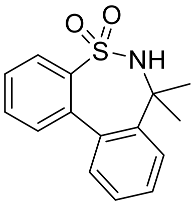

7,7-dimethyl-6,7-dihydrodibenzo[d,f][1,2]thiazepine 5,5-dioxide, 2p

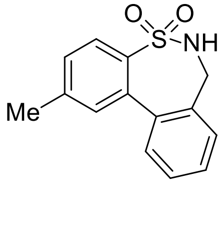

2-methyl-6,7-dihydrodibenzo[d,f][1,2]thiazepine 5,5-dioxide, 2q

^{1}H NMR (600 MHz, $CDCl_3$): δ 7.92 (d, *J* = 7.8 Hz, 1H), 7.51-7.49 (m, 1H), 7.46 (d, *J* = 7.2 Hz, 1H), 7.43-7.42 (m, 2H), 7.39 (s, 1H), 7.36 (d, *J* = 8.1Hz, 1H), 5.02 (s, 1H), 4.06 (s, 2H), 2.51 (s, 3H)； **^{13}C NMR** (150 MHz, $CDCl_3$): δ 144.0, 140.9, 138.8, 134.5, 132.5, 130.7, 130.2, 129.5, 129.1, 129.0, 128.8, 126.3,

47.6, 29.6, 21.5；**HRMS** (EI): 259.0668 (calculated $C_{14}H_{13}NO_2S$ 259.0667).

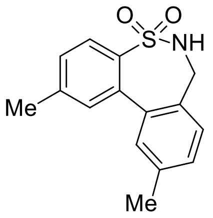

2,10-dimethyl-6,7-dihydrodibenzo[d,f][1,2]thiazepine 5,5-dioxide, 2r

^{1}H NMR (600 MHz, $CDCl_3$): δ 7.92 (d, *J* = 8.4 Hz, 1H), 7.38 (s, 1H), 7.34 (d, *J* = 7.8 Hz, 1H), 7.32 (d, *J* = 11.4 Hz, 1H), 7.27 (s, 1H), 7.22 (d, *J* = 7.8 Hz, 1H), 4.98 (s, 1H), 4.03 (s, 2H), 2.50 (s, 3H), 2.42 (s, 3H)；**^{13}C NMR** (150 MHz, $CDCl_3$): δ 143.9, 140.7, 139.4, 139.0, 134.5, 130.7, 130.1, 129.7, 129.6(2C), 128.8, 126.3, 47.2, 29.7, 21.5, 21.3；**HRMS** (EI): 273.0825 (calculated $C_{15}H_{15}NO_2S$ 273.0823).

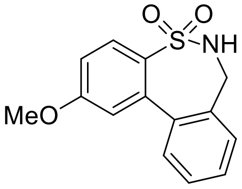

2-methoxy-6,7-dihydrodibenzo[d,f][1,2]thiazepine 5,5-dioxide, 2s

^{1}H NMR (600 MHz, $CDCl_3$): δ 7.98 (d, *J* = 8.7 Hz, 1H), 7.52–7.49 (m, 1H), 7.47 (d, *J* = 7.5 Hz, 1H), 7.43 (d, *J* = 4.2 Hz, 2H), 7.07 (sd, *J* = 2.1 Hz, 1H), 7.02 (dd, *J* = 8.7, 2.4 Hz, 1H), 5.01 (s, 1H), 4.07 (s, 2H), 3.93 (s, 3H)；**^{13}C NMR** (150 MHz, $CDCl_3$): δ 163.1, 140.9, 140.7, 132.6, 130.3, 129.6, 129.4, 129.3, 128.7, 128.5, 115.8, 112.8, 55.7, 47.5；**HRMS** (EI): 275.0614 (calculated $C_{14}H_{13}NO_3S$ 275.0616).

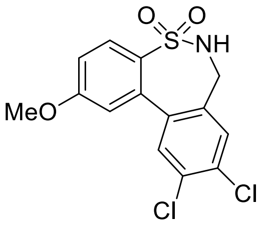

9,10-dichloro-2-methoxy-6,7-dihydrodibenzo[d,f][1,2]thiazepine 5,5-dioxide, 2t

^{1}H NMR (600 MHz, $CDCl_3$): δ 7.98 (d, *J* = 9.0 Hz, 1H), 7.53 (d, *J* = 10.2 Hz, 2H), 7.05 (dd, *J* = 9.0, 2.4 Hz, 1H), 7.02 (sd, *J* = 2.4 Hz, 1H), 5.00 (s, 5H), 4.00 (s, 2H), 3.94 (s, 3H)；**^{13}C NMR** (150 MHz, $CDCl_3$): δ 163.3, 140.6, 138.6, 133.7, 133.2, 132.4, 132.1, 130.3, 129.3, 128.8, 115.8, 113.5, 55.9, 46.5, 29.7；**HRMS** (EI): 342.9833 (calculated $C_{14}H_{11}Cl_2NO_3S$ 342.9837).

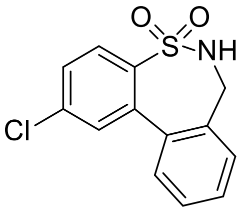

2-chloro-6,7-dihydrodibenzo[d,f][1,2]thiazepine 5,5-dioxide, 2u

^{1}H NMR (600 MHz, $CDCl_3$): δ 7.98 (d, *J* = 8.4 Hz, 1H), 7.85 (s, 1H), 7.53 (t, *J* = 8.4 Hz, 2H), 7.47-7.44 (m, 3H), 5.09 (s, 1H), 4.09 (s, 2H); **^{13}C NMR** (150 MHz, $CDCl_3$): δ 140.6, 139.6, 139.4, 135.7, 132.4, 130.4, 130.1, 129.8, 129.7, 128.9, 128.4, 127.7, 47.4.; **HRMS** (EI): 279.0123 (calculated $C_{13}H_{10}ClNO_2S$ 279.0121).

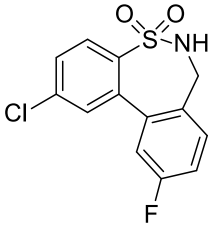

2-chloro-10-fluoro-6,7-dihydrodibenzo[d,f][1,2]thiazepine 5,5-dioxide, 2v

^{1}H NMR (600 MHz, $CDCl_3$): δ 7.99 (d, *J* = 9.0 Hz, 1H), 7.57 (t, *J* = 4.2 Hz, 2H), 7.42 (t, *J* = 6.9 Hz, 1H), 7.18–7.13 (m, 2H), 5.05 (s, 1H), 4.06 (s, 2H); **^{13}C NMR** (150 MHz, $CDCl_3$): δ 164.1, 162.4, 141.7(2C), 139.6, 139.4, 135.8, 132.3, 132.2, 129.9, 129.0, 128.2, 127.9, 116.4, 116.3, 116.2, 116.0, 46.6.; **HRMS** (ESI): 297.0030 (calculated $C_{13}H_9ClFNO_2S$ 297.0027).

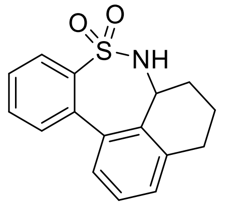

2,3,3a,4-tetrahydro-1H-benzo[f]naphtho[1,8-cd][1,2]thiazepine 5,5-dioxide, 2w

^{1}H NMR (400 MHz, $CDCl_3$) δ 8.03 (q, *J* = 2.9 Hz, 1H), 7.72 (m, *J* = 3.3 Hz, 1H), 7.58 (m, *J* = 3.6 Hz, 2H), 7.41 (t, *J* = 7.6 Hz, 1H), 7.25 (d, *J* = 8.0 Hz, 2H), 4.92 (s, 1H), 4.17 (s, 1H), 3.03 (q, *J* = 7.5 Hz, 1H), 2.85 (q, *J* = 5.9 Hz, 1H), 2.28 (m, *J* = 4.2 Hz, 1H), 1.84 (m, *J* = 6.8 Hz, 3H).; ^{13}C NMR (101 MHz, $CDCl_3$) δ 140.8, 139.2, 138.0, 136.8, 133.2, 130.3, 130.2, 128.9, 128.4, 126.6, 125.7, 50.2 (benzylic C), 29.6, 29.3, 17.0.; **HRMS** [(ESI), $(M+Na)^+$]: 307.0725. (calculated $C_{16}H_{15}NNaO_2S$ 307.0721).

5,6-dihydrobenzo[f]pyrido[2,3-d][1,2]thiazepine 7,7-dioxide, 2x

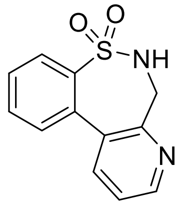

^{1}H NMR (400 MHz, $CDCl_3$) δ 8.07 (q, *J* = 2.9 Hz, 1H), 7.74 (m, *J* = 3.3 Hz, 1H), 7.60 (m, *J* = 3.0 Hz, 2H), 7.46 (q, *J* = 4.6 Hz, 1H), 7.22 (m, *J* = 3.9 Hz, 2H), 5.16 (s, 1H), 4.07 (d, *J* = 2.5 Hz, 2H); **^{13}C NMR** (101 MHz, $CDCl_3$) δ 164.1, 137.0, 134.6, 133.4, 130.7, 130.1, 128.6, 126.4, 117.7, 117.5, 116.7, 116.5, 47.2 (benzylic C).; **HRMS** [(ESI), $(M+Na)^+$]: 269.0309 (calculated $C_{12}H_{10}N_2O_2S$ 269.0361).

ORTEP and x-ray data of 2p (ccdc-2491640)

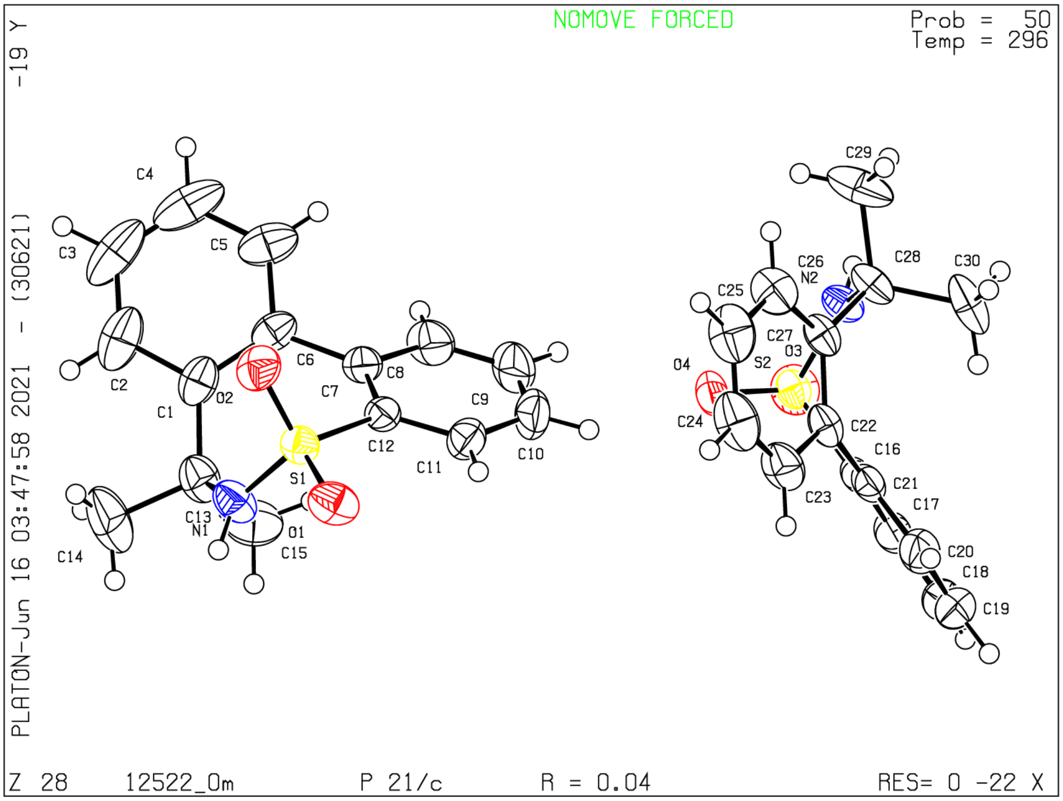

Table S1. Crystal data and structure refinement for 2p.

Identification code	12522_0m	
Empirical formula	C30 H30 N2 O4 S2	
Formula weight	546.68	
Temperature	296(2) K	
Wavelength	0.71073 Å	
Crystal system	Monoclinic	
Space group	$P2_1/c$	
Unit cell dimensions	a = 14.7205(4) Å	a= 90°.
	b = 7.4291(2) Å	b= 91.0459(13)°.
	c = 25.1584(6) Å	g = 90°.
Volume	2750.87(12) $Å^3$	
Z	4	
Density (calculated)	1.320 Mg/m^3	
Absorption coefficient	0.232 mm^{-1}	
F(000)	1152	
Crystal size	0.500 x 0.400 x 0.200 mm^3	
Theta range for data collection	1.384 to 28.304°.	
Index ranges	-18<=h<=19, -9<=k<=9, -30<=l<=33	
Reflections collected	48327	
Independent reflections	6816 [R(int) = 0.0281]	
Completeness to theta = 25.242°	100.0 %	
Absorption correction	None	
Refinement method	Full-matrix least-squares on F^2	
Data / restraints / parameters	6816 / 0 / 347	
Goodness-of-fit on F^2	1.032	
Final R indices [I>2sigma(I)]	R1 = 0.0439, wR2 = 0.1136	
R indices (all data)	R1 = 0.0584, wR2 = 0.1232	
Extinction coefficient	n/a	
Largest diff. peak and hole	0.385 and -0.436 e.$Å^{-3}$	

Table S2. Atomic coordinates (x 10^4) and equivalent isotropic displacement parameters ($Å^2$x 10^3) for **2p**. U(eq) is defined as one third of the trace of the orthogonalized U^{ij} tensor.

	x	y	z	U(eq)
S(1)	4521(1)	2601(1)	4496(1)	36(1)
S(2)	9463(1)	7207(1)	3200(1)	32(1)
O(1)	4891(1)	2530(2)	5027(1)	51(1)
O(2)	3692(1)	3564(2)	4406(1)	48(1)
O(3)	9784(1)	6530(2)	2703(1)	50(1)
O(4)	8626(1)	6519(2)	3393(1)	42(1)
N(1)	4408(1)	533(2)	4318(1)	46(1)
N(2)	9392(1)	9344(2)	3131(1)	35(1)
C(1)	3838(1)	1292(3)	3408(1)	47(1)
C(2)	2987(2)	979(4)	3175(1)	72(1)
C(3)	2589(2)	2161(6)	2824(1)	89(1)
C(4)	3029(2)	3690(5)	2686(1)	85(1)
C(5)	3872(2)	4077(4)	2914(1)	62(1)
C(6)	4281(1)	2909(3)	3280(1)	42(1)
C(7)	5159(1)	3531(2)	3524(1)	37(1)
C(8)	5835(1)	4256(3)	3203(1)	53(1)
C(9)	6625(2)	4950(3)	3419(1)	62(1)
C(10)	6776(1)	4957(3)	3957(1)	58(1)
C(11)	6132(1)	4222(3)	4290(1)	44(1)
C(12)	5336(1)	3518(2)	4071(1)	33(1)
C(13)	4276(1)	-145(3)	3767(1)	49(1)
C(14)	3676(2)	-1821(4)	3840(1)	86(1)
C(15)	5191(2)	-751(3)	3548(1)	64(1)
C(16)	10303(1)	6854(2)	3695(1)	34(1)
C(17)	11093(1)	5933(2)	3568(1)	45(1)
C(18)	11749(1)	5645(3)	3955(1)	59(1)
C(19)	11624(2)	6309(3)	4457(1)	64(1)
C(20)	10843(1)	7242(3)	4584(1)	53(1)
C(21)	10154(1)	7525(2)	4206(1)	36(1)
C(22)	9287(1)	8421(2)	4356(1)	36(1)
C(23)	8886(2)	7790(3)	4819(1)	50(1)

C(24)	8064(2)	8428(3)	4988(1)	61(1)
C(25)	7619(2)	9712(3)	4694(1)	61(1)
C(26)	8005(1)	10379(3)	4237(1)	50(1)
C(27)	8842(1)	9771(2)	4059(1)	35(1)
C(28)	9265(1)	10659(2)	3570(1)	39(1)
C(29)	8659(2)	12122(3)	3323(1)	69(1)
C(30)	10184(2)	11494(3)	3723(1)	60(1)

Table s3. Bond lengths [Å] and angles [°] for **2p**.

S(1)-O(2)	1.4289(13)
S(1)-O(1)	1.4354(14)
S(1)-N(1)	1.6079(16)
S(1)-C(12)	1.7582(16)
S(2)-O(4)	1.4264(12)
S(2)-O(3)	1.4368(13)
S(2)-N(2)	1.6010(14)
S(2)-C(16)	1.7586(17)
N(1)-C(13)	1.484(2)
N(1)-H(1B)	0.8600
N(2)-C(28)	1.489(2)
N(2)-H(2B)	0.8600
C(1)-C(2)	1.394(3)
C(1)-C(6)	1.406(3)
C(1)-C(13)	1.532(3)
C(2)-C(3)	1.369(4)
C(2)-H(2C)	0.9300
C(3)-C(4)	1.356(5)
C(3)-H(3B)	0.9300
C(4)-C(5)	1.388(4)
C(4)-H(4B)	0.9300
C(5)-C(6)	1.393(3)
C(5)-H(5A)	0.9300
C(6)-C(7)	1.495(2)
C(7)-C(12)	1.394(2)
C(7)-C(8)	1.402(2)
C(8)-C(9)	1.375(3)
C(8)-H(8A)	0.9300
C(9)-C(10)	1.367(3)
C(9)-H(9A)	0.9300
C(10)-C(11)	1.390(3)
C(10)-H(10A)	0.9300
C(11)-C(12)	1.388(2)
C(11)-H(11A)	0.9300

C(13)-C(15)	1.531(3)
C(13)-C(14)	1.540(3)
C(14)-H(14A)	0.9600
C(14)-H(14B)	0.9600
C(14)-H(14C)	0.9600
C(15)-H(15A)	0.9600
C(15)-H(15B)	0.9600
C(15)-H(15C)	0.9600
C(16)-C(17)	1.391(2)
C(16)-C(21)	1.400(2)
C(17)-C(18)	1.375(3)
C(17)-H(17A)	0.9300
C(18)-C(19)	1.373(4)
C(18)-H(18A)	0.9300
C(19)-C(20)	1.386(3)
C(19)-H(19A)	0.9300
C(20)-C(21)	1.394(3)
C(20)-H(20A)	0.9300
C(21)-C(22)	1.494(2)
C(22)-C(23)	1.397(2)
C(22)-C(27)	1.406(2)
C(23)-C(24)	1.375(3)
C(23)-H(23A)	0.9300
C(24)-C(25)	1.367(4)
C(24)-H(24A)	0.9300
C(25)-C(26)	1.383(3)
C(25)-H(25A)	0.9300
C(26)-C(27)	1.394(3)
C(26)-H(26A)	0.9300
C(27)-C(28)	1.537(2)
C(28)-C(29)	1.531(3)
C(28)-C(30)	1.532(3)
C(29)-H(29A)	0.9600
C(29)-H(29B)	0.9600
C(29)-H(29C)	0.9600
C(30)-H(30A)	0.9600

C(30)-H(30B)	0.9600
C(30)-H(30C)	0.9600
O(2)-S(1)-O(1)	118.26(9)
O(2)-S(1)-N(1)	110.54(9)
O(1)-S(1)-N(1)	105.08(8)
O(2)-S(1)-C(12)	107.43(8)
O(1)-S(1)-C(12)	109.26(9)
N(1)-S(1)-C(12)	105.59(8)
O(4)-S(2)-O(3)	118.13(8)
O(4)-S(2)-N(2)	109.70(8)
O(3)-S(2)-N(2)	105.91(8)
O(4)-S(2)-C(16)	107.85(8)
O(3)-S(2)-C(16)	109.01(8)
N(2)-S(2)-C(16)	105.56(7)
C(13)-N(1)-S(1)	126.57(13)
C(13)-N(1)-H(1B)	116.7
S(1)-N(1)-H(1B)	116.7
C(28)-N(2)-S(2)	125.37(11)
C(28)-N(2)-H(2B)	117.3
S(2)-N(2)-H(2B)	117.3
C(2)-C(1)-C(6)	117.6(2)
C(2)-C(1)-C(13)	119.8(2)
C(6)-C(1)-C(13)	122.59(16)
C(3)-C(2)-C(1)	122.4(3)
C(3)-C(2)-H(2C)	118.8
C(1)-C(2)-H(2C)	118.8
C(4)-C(3)-C(2)	120.1(2)
C(4)-C(3)-H(3B)	120.0
C(2)-C(3)-H(3B)	120.0
C(3)-C(4)-C(5)	119.7(3)
C(3)-C(4)-H(4B)	120.2
C(5)-C(4)-H(4B)	120.2
C(4)-C(5)-C(6)	121.1(3)
C(4)-C(5)-H(5A)	119.4
C(6)-C(5)-H(5A)	119.4

C(5)-C(6)-C(1)	119.15(19)
C(5)-C(6)-C(7)	116.04(19)
C(1)-C(6)-C(7)	124.77(16)
C(12)-C(7)-C(8)	116.79(17)
C(12)-C(7)-C(6)	123.21(15)
C(8)-C(7)-C(6)	119.93(16)
C(9)-C(8)-C(7)	121.3(2)
C(9)-C(8)-H(8A)	119.3
C(7)-C(8)-H(8A)	119.3
C(10)-C(9)-C(8)	120.92(19)
C(10)-C(9)-H(9A)	119.5
C(8)-C(9)-H(9A)	119.5
C(9)-C(10)-C(11)	119.72(19)
C(9)-C(10)-H(10A)	120.1
C(11)-C(10)-H(10A)	120.1
C(12)-C(11)-C(10)	119.24(19)
C(12)-C(11)-H(11A)	120.4
C(10)-C(11)-H(11A)	120.4
C(11)-C(12)-C(7)	122.00(16)
C(11)-C(12)-S(1)	118.94(14)
C(7)-C(12)-S(1)	119.06(13)
N(1)-C(13)-C(15)	109.51(17)
N(1)-C(13)-C(1)	111.09(16)
C(15)-C(13)-C(1)	110.88(17)
N(1)-C(13)-C(14)	103.20(18)
C(15)-C(13)-C(14)	108.4(2)
C(1)-C(13)-C(14)	113.5(2)
C(13)-C(14)-H(14A)	109.5
C(13)-C(14)-H(14B)	109.5
H(14A)-C(14)-H(14B)	109.5
C(13)-C(14)-H(14C)	109.5
H(14A)-C(14)-H(14C)	109.5
H(14B)-C(14)-H(14C)	109.5
C(13)-C(15)-H(15A)	109.5
C(13)-C(15)-H(15B)	109.5
H(15A)-C(15)-H(15B)	109.5

C(13)-C(15)-H(15C)	109.5
H(15A)-C(15)-H(15C)	109.5
H(15B)-C(15)-H(15C)	109.5
C(17)-C(16)-C(21)	122.15(17)
C(17)-C(16)-S(2)	119.39(14)
C(21)-C(16)-S(2)	118.46(13)
C(18)-C(17)-C(16)	119.6(2)
C(18)-C(17)-H(17A)	120.2
C(16)-C(17)-H(17A)	120.2
C(19)-C(18)-C(17)	119.4(2)
C(19)-C(18)-H(18A)	120.3
C(17)-C(18)-H(18A)	120.3
C(18)-C(19)-C(20)	121.2(2)
C(18)-C(19)-H(19A)	119.4
C(20)-C(19)-H(19A)	119.4
C(19)-C(20)-C(21)	121.0(2)
C(19)-C(20)-H(20A)	119.5
C(21)-C(20)-H(20A)	119.5
C(20)-C(21)-C(16)	116.66(18)
C(20)-C(21)-C(22)	120.59(17)
C(16)-C(21)-C(22)	122.68(15)
C(23)-C(22)-C(27)	118.86(17)
C(23)-C(22)-C(21)	115.90(17)
C(27)-C(22)-C(21)	125.19(14)
C(24)-C(23)-C(22)	122.0(2)
C(24)-C(23)-H(23A)	119.0
C(22)-C(23)-H(23A)	119.0
C(25)-C(24)-C(23)	119.26(19)
C(25)-C(24)-H(24A)	120.4
C(23)-C(24)-H(24A)	120.4
C(24)-C(25)-C(26)	120.1(2)
C(24)-C(25)-H(25A)	120.0
C(26)-C(25)-H(25A)	120.0
C(25)-C(26)-C(27)	122.0(2)
C(25)-C(26)-H(26A)	119.0
C(27)-C(26)-H(26A)	119.0

C(26)-C(27)-C(22)	117.84(16)
C(26)-C(27)-C(28)	119.45(16)
C(22)-C(27)-C(28)	122.63(15)
N(2)-C(28)-C(29)	104.11(15)
N(2)-C(28)-C(30)	109.28(15)
C(29)-C(28)-C(30)	108.69(18)
N(2)-C(28)-C(27)	111.73(13)
C(29)-C(28)-C(27)	112.82(16)
C(30)-C(28)-C(27)	109.99(15)
C(28)-C(29)-H(29A)	109.5
C(28)-C(29)-H(29B)	109.5
H(29A)-C(29)-H(29B)	109.5
C(28)-C(29)-H(29C)	109.5
H(29A)-C(29)-H(29C)	109.5
H(29B)-C(29)-H(29C)	109.5
C(28)-C(30)-H(30A)	109.5
C(28)-C(30)-H(30B)	109.5
H(30A)-C(30)-H(30B)	109.5
C(28)-C(30)-H(30C)	109.5
H(30A)-C(30)-H(30C)	109.5
H(30B)-C(30)-H(30C)	109.5

Symmetry transformations used to generate equivalent atoms:

Table S4. Anisotropic displacement parameters ($Å^2$x 10^3) for **2p**. The anisotropic displacement factor exponent takes the form: $-2p^2[h^2 a^{*2}U^{11} + ... + 2 h k a^* b^* U^{12}]$

	U^{11}	U^{22}	U^{33}	U^{23}	U^{13}	U^{12}
S(1)	38(1)	39(1)	32(1)	2(1)	5(1)	0(1)
S(2)	36(1)	26(1)	34(1)	-6(1)	6(1)	-2(1)
O(1)	72(1)	50(1)	31(1)	2(1)	-1(1)	1(1)
O(2)	35(1)	58(1)	51(1)	1(1)	11(1)	6(1)
O(3)	61(1)	49(1)	42(1)	-18(1)	11(1)	4(1)
O(4)	37(1)	35(1)	53(1)	-4(1)	3(1)	-11(1)
N(1)	63(1)	38(1)	38(1)	5(1)	2(1)	-8(1)
N(2)	50(1)	28(1)	29(1)	2(1)	9(1)	1(1)
C(1)	37(1)	64(1)	39(1)	-10(1)	-1(1)	-4(1)
C(2)	49(1)	107(2)	60(1)	-20(1)	-10(1)	-13(1)
C(3)	50(1)	152(3)	63(2)	-26(2)	-23(1)	12(2)
C(4)	75(2)	132(3)	49(1)	-3(2)	-21(1)	43(2)
C(5)	67(1)	80(2)	40(1)	5(1)	-5(1)	23(1)
C(6)	38(1)	57(1)	31(1)	-1(1)	0(1)	9(1)
C(7)	35(1)	39(1)	38(1)	6(1)	5(1)	4(1)
C(8)	51(1)	60(1)	49(1)	13(1)	16(1)	1(1)
C(9)	45(1)	63(1)	80(2)	14(1)	21(1)	-8(1)
C(10)	31(1)	51(1)	91(2)	-4(1)	4(1)	-7(1)
C(11)	36(1)	43(1)	55(1)	-5(1)	-4(1)	2(1)
C(12)	29(1)	32(1)	39(1)	2(1)	4(1)	3(1)
C(13)	57(1)	43(1)	47(1)	-6(1)	1(1)	-10(1)
C(14)	110(2)	61(2)	88(2)	-9(1)	1(2)	-43(2)
C(15)	80(2)	55(1)	58(1)	-5(1)	6(1)	18(1)
C(16)	32(1)	24(1)	44(1)	2(1)	4(1)	-5(1)
C(17)	37(1)	33(1)	66(1)	2(1)	10(1)	-1(1)
C(18)	35(1)	47(1)	97(2)	15(1)	-1(1)	0(1)
C(19)	46(1)	63(1)	82(2)	26(1)	-20(1)	-10(1)
C(20)	54(1)	56(1)	48(1)	12(1)	-8(1)	-14(1)
C(21)	40(1)	31(1)	37(1)	7(1)	2(1)	-9(1)
C(22)	44(1)	33(1)	30(1)	-2(1)	7(1)	-11(1)
C(23)	61(1)	53(1)	37(1)	7(1)	10(1)	-13(1)

C(24)	69(1)	69(1)	46(1)	-1(1)	28(1)	-20(1)
C(25)	54(1)	70(1)	60(1)	-12(1)	29(1)	-7(1)
C(26)	55(1)	44(1)	52(1)	-6(1)	16(1)	4(1)
C(27)	44(1)	28(1)	34(1)	-5(1)	12(1)	-6(1)
C(28)	58(1)	23(1)	36(1)	-1(1)	14(1)	-4(1)
C(29)	106(2)	36(1)	65(1)	15(1)	32(1)	24(1)
C(30)	84(2)	46(1)	51(1)	-5(1)	17(1)	-36(1)

Table S5. Hydrogen coordinates (x 10^4) and isotropic displacement parameters ($Å^2$x 10 ^{3})for **2p**.

	x	y	z	U(eq)
H(1B)	4426	-260	4567	55
H(2B)	9430	9768	2814	43
H(2C)	2678	-71	3260	86
H(3B)	2015	1916	2680	106
H(4B)	2768	4475	2439	102
H(5A)	4169	5133	2822	75
H(8A)	5748	4266	2836	64
H(9A)	7063	5423	3196	75
H(10A)	7307	5452	4099	69
H(11A)	6232	4201	4656	53
H(14A)	3107	-1470	3990	130
H(14B)	3979	-2654	4075	130
H(14C)	3567	-2385	3502	130
H(15A)	5590	267	3525	97
H(15B)	5099	-1263	3201	97
H(15C)	5456	-1636	3781	97
H(17A)	11176	5515	3224	55
H(18A)	12273	5006	3876	71
H(19A)	12073	6130	4717	76
H(20A)	10776	7685	4927	63
H(23A)	9185	6912	5020	60
H(24A)	7812	7991	5299	73
H(25A)	7057	10138	4801	73
H(26A)	7696	11260	4043	60
H(29A)	8927	12555	3002	103
H(29B)	8599	13099	3569	103
H(29C)	8070	11629	3242	103
H(30A)	10439	12066	3418	91
H(30B)	10589	10569	3850	91
H(30C)	10102	12371	3999	91

ORTEP and x-ray data of 5 (ccdc-2491641)

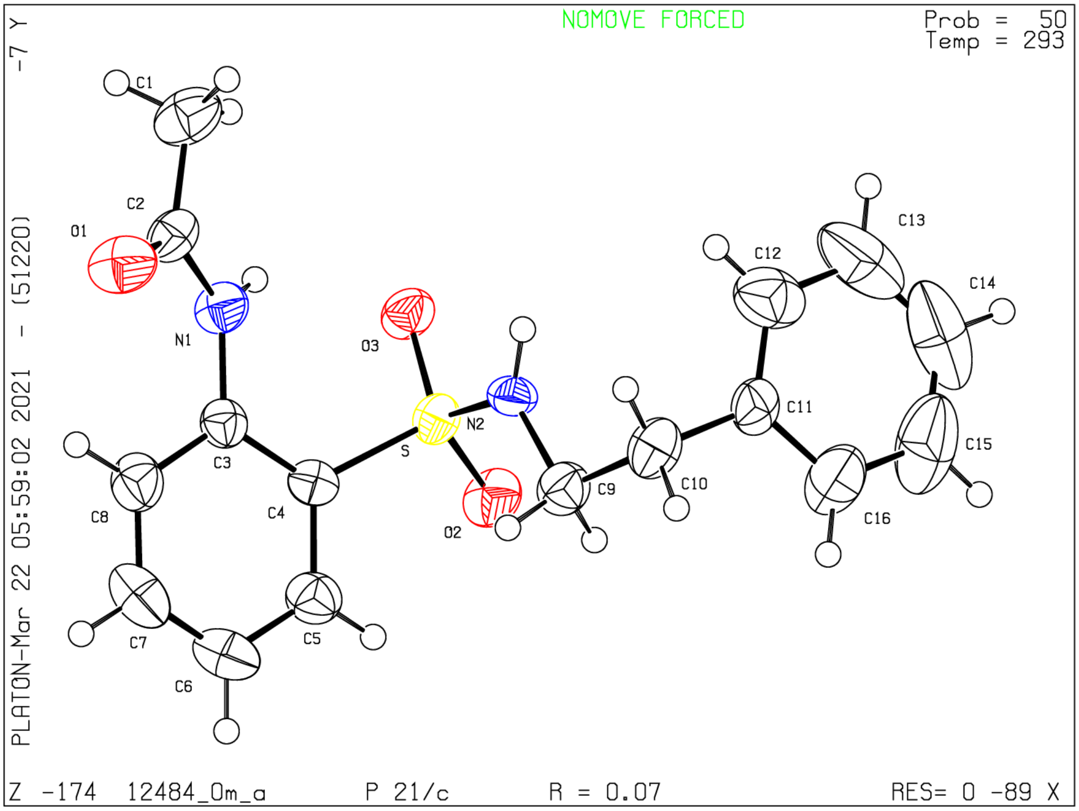

Table S6. Crystal data and structure refinement for **5**.

Identification code	12484_0m_a	
Empirical formula	C16 H18 N2 O3 S	
Formula weight	318.38	
Temperature	293(2) K	
Wavelength	0.71073 Å	
Crystal system	Monoclinic	
Space group	$P2_1/c$	
Unit cell dimensions	a = 12.182(3) Å	a= 90°.
	b = 8.791(2) Å	b= 112.063(16)°.
	c = 15.927(4) Å	g = 90°.
Volume	1580.8(7) $Å^3$	
Z	4	
Density (calculated)	1.338 Mg/m^3	
Absorption coefficient	0.219 mm^{-1}	
F(000)	672	
Crystal size	0.200 x 0.200 x 0.100 mm^3	
Theta range for data collection	1.804 to 25.997°.	
Index ranges	-13<=h<=15, -10<=k<=10, -19<=l<=16	
Reflections collected	13341	
Independent reflections	3099 [R(int) = 0.1116]	
Completeness to theta = 25.242°	99.9 %	
Absorption correction	Semi-empirical from equivalents	
Max. and min. transmission	0.7457 and 0.6630	
Refinement method	Full-matrix least-squares on F^2	
Data / restraints / parameters	3099 / 0 / 199	
Goodness-of-fit on F^2	1.000	
Final R indices [I>2sigma(I)]	R1 = 0.0684, wR2 = 0.1149	
R indices (all data)	R1 = 0.1819, wR2 = 0.1454	
Extinction coefficient	n/a	
Largest diff. peak and hole	0.348 and -0.390 e.$Å^{-3}$	

Table S7. Atomic coordinates (x 10^4) and equivalent isotropic displacement parameters ($Å^2$x 10^3) for **5**. U(eq) is defined as one third of the trace of the orthogonalized U^{ij} tensor.

	x	y	z	U(eq)
S	7938(1)	1274(1)	3744(1)	36(1)
O(1)	11368(3)	5073(3)	4784(2)	55(1)
O(2)	7104(2)	253(3)	3138(2)	49(1)
O(3)	8786(2)	683(3)	4575(2)	43(1)
N(1)	10408(3)	2806(3)	4492(2)	42(1)
N(2)	7211(3)	2592(3)	4004(2)	35(1)
C(1)	11627(4)	3411(5)	6030(3)	57(1)
C(2)	11140(3)	3855(5)	5051(3)	41(1)
C(3)	9803(3)	2909(4)	3552(3)	35(1)
C(4)	8718(3)	2168(4)	3133(3)	33(1)
C(5)	8138(4)	2211(4)	2193(3)	45(1)
C(6)	8622(4)	3001(5)	1671(3)	57(1)
C(7)	9676(4)	3739(5)	2084(3)	56(1)
C(8)	10270(4)	3695(5)	3009(3)	48(1)
C(9)	6198(3)	3293(4)	3294(3)	44(1)
C(10)	5450(3)	4180(4)	3705(3)	48(1)
C(11)	4784(4)	3189(5)	4116(3)	43(1)
C(12)	5223(5)	2815(6)	5012(4)	68(1)
C(13)	4619(8)	1858(8)	5381(5)	100(2)
C(14)	3548(7)	1292(7)	4832(7)	108(3)
C(15)	3102(5)	1639(7)	3938(6)	104(2)
C(16)	3708(4)	2590(6)	3580(4)	72(2)

Table S8. Bond lengths [Å] and angles [°] for 5.

S-O(2)	1.426(2)
S-O(3)	1.435(3)
S-N(2)	1.604(3)
S-C(4)	1.778(4)
O(1)-C(2)	1.222(4)
N(1)-C(2)	1.357(5)
N(1)-C(3)	1.402(4)
N(1)-H(1A)	0.8600
N(2)-C(9)	1.461(4)
N(2)-H(2B)	0.8600
C(1)-C(2)	1.497(5)
C(1)-H(1B)	0.9600
C(1)-H(1C)	0.9600
C(1)-H(1D)	0.9600
C(3)-C(8)	1.386(5)
C(3)-C(4)	1.398(5)
C(4)-C(5)	1.395(5)
C(5)-C(6)	1.375(5)
C(5)-H(5A)	0.9300
C(6)-C(7)	1.367(6)
C(6)-H(6A)	0.9300
C(7)-C(8)	1.376(5)
C(7)-H(7A)	0.9300
C(8)-H(8A)	0.9300
C(9)-C(10)	1.523(5)
C(9)-H(9A)	0.9700
C(9)-H(9B)	0.9700
C(10)-C(11)	1.497(5)
C(10)-H(10A)	0.9700
C(10)-H(10B)	0.9700
C(11)-C(12)	1.363(6)
C(11)-C(16)	1.372(6)
C(12)-C(13)	1.386(8)
C(12)-H(12A)	0.9300

C(13)-C(14)	1.363(9)
C(13)-H(13A)	0.9300
C(14)-C(15)	1.355(8)
C(14)-H(14A)	0.9300
C(15)-C(16)	1.373(7)
C(15)-H(15A)	0.9300
C(16)-H(16A)	0.9300
O(2)-S-O(3)	118.62(17)
O(2)-S-N(2)	107.89(16)
O(3)-S-N(2)	107.40(16)
O(2)-S-C(4)	107.42(17)
O(3)-S-C(4)	108.49(17)
N(2)-S-C(4)	106.41(16)
C(2)-N(1)-C(3)	127.5(3)
C(2)-N(1)-H(1A)	116.3
C(3)-N(1)-H(1A)	116.3
C(9)-N(2)-S	119.5(3)
C(9)-N(2)-H(2B)	120.3
S-N(2)-H(2B)	120.3
C(2)-C(1)-H(1B)	109.5
C(2)-C(1)-H(1C)	109.5
H(1B)-C(1)-H(1C)	109.5
C(2)-C(1)-H(1D)	109.5
H(1B)-C(1)-H(1D)	109.5
H(1C)-C(1)-H(1D)	109.5
O(1)-C(2)-N(1)	123.2(4)
O(1)-C(2)-C(1)	122.5(4)
N(1)-C(2)-C(1)	114.3(4)
C(8)-C(3)-C(4)	118.1(4)
C(8)-C(3)-N(1)	121.6(4)
C(4)-C(3)-N(1)	120.2(3)
C(5)-C(4)-C(3)	120.4(4)
C(5)-C(4)-S	116.1(3)
C(3)-C(4)-S	123.3(3)
C(6)-C(5)-C(4)	120.2(4)

C(6)-C(5)-H(5A)	119.9
C(4)-C(5)-H(5A)	119.9
C(7)-C(6)-C(5)	119.2(4)
C(7)-C(6)-H(6A)	120.4
C(5)-C(6)-H(6A)	120.4
C(6)-C(7)-C(8)	121.5(4)
C(6)-C(7)-H(7A)	119.2
C(8)-C(7)-H(7A)	119.2
C(7)-C(8)-C(3)	120.5(4)
C(7)-C(8)-H(8A)	119.7
C(3)-C(8)-H(8A)	119.7
N(2)-C(9)-C(10)	110.5(3)
N(2)-C(9)-H(9A)	109.5
C(10)-C(9)-H(9A)	109.5
N(2)-C(9)-H(9B)	109.5
C(10)-C(9)-H(9B)	109.5
H(9A)-C(9)-H(9B)	108.1
C(11)-C(10)-C(9)	113.6(3)
C(11)-C(10)-H(10A)	108.9
C(9)-C(10)-H(10A)	108.9
C(11)-C(10)-H(10B)	108.9
C(9)-C(10)-H(10B)	108.8
H(10A)-C(10)-H(10B)	107.7
C(12)-C(11)-C(16)	117.7(4)
C(12)-C(11)-C(10)	122.3(4)
C(16)-C(11)-C(10)	120.0(4)
C(11)-C(12)-C(13)	122.0(6)
C(11)-C(12)-H(12A)	119.0
C(13)-C(12)-H(12A)	119.0
C(14)-C(13)-C(12)	118.8(6)
C(14)-C(13)-H(13A)	120.6
C(12)-C(13)-H(13A)	120.6
C(15)-C(14)-C(13)	120.1(6)
C(15)-C(14)-H(14A)	120.0
C(13)-C(14)-H(14A)	120.0
C(14)-C(15)-C(16)	120.5(6)

C(14)-C(15)-H(15A)	119.8
C(16)-C(15)-H(15A)	119.8
C(11)-C(16)-C(15)	120.9(5)
C(11)-C(16)-H(16A)	119.6
C(15)-C(16)-H(16A)	119.6

Symmetry transformations used to generate equivalent atoms:

Table S9. Anisotropic displacement parameters (Å^{2}x 10^3) for **5**. The anisotropic displacement factor exponent takes the form: -2p^2[h^2 a*^{2}U^{11} + ... + 2 h k a* b* U^{12}]

	U^{11}	U^{22}	U^{33}	U^{23}	U^{13}	U^{12}
S	34(1)	32(1)	40(1)	-3(1)	12(1)	-6(1)
O(1)	66(2)	39(2)	52(2)	-2(2)	11(2)	-16(2)
O(2)	48(2)	38(2)	54(2)	-15(1)	13(2)	-18(1)
O(3)	37(2)	44(2)	42(2)	11(1)	8(1)	1(1)
N(1)	42(2)	35(2)	42(2)	4(2)	11(2)	-11(2)
N(2)	36(2)	44(2)	24(2)	-3(2)	10(2)	6(2)
C(1)	56(3)	46(3)	56(3)	-1(2)	5(2)	-11(2)
C(2)	34(3)	38(3)	45(3)	-7(2)	9(2)	-2(2)
C(3)	36(3)	34(2)	37(3)	-3(2)	16(2)	0(2)
C(4)	40(3)	27(2)	32(3)	-2(2)	14(2)	3(2)
C(5)	50(3)	45(3)	41(3)	-5(2)	18(2)	-2(2)
C(6)	76(4)	60(3)	38(3)	-1(3)	25(3)	-1(3)
C(7)	80(4)	46(3)	59(3)	-5(3)	45(3)	-7(3)
C(8)	48(3)	48(3)	53(3)	-10(2)	23(2)	-12(2)
C(9)	40(3)	45(3)	45(3)	4(2)	13(2)	0(2)
C(10)	39(3)	44(3)	59(3)	1(2)	15(2)	7(2)
C(11)	33(3)	46(3)	51(3)	9(2)	17(2)	14(2)
C(12)	78(4)	74(4)	56(4)	-2(3)	28(3)	18(3)
C(13)	145(7)	102(5)	81(5)	37(4)	75(5)	47(5)
C(14)	125(7)	79(5)	173(8)	42(5)	116(7)	28(5)
C(15)	63(4)	87(5)	161(7)	31(5)	42(5)	-11(3)
C(16)	47(3)	78(4)	80(4)	26(3)	11(3)	-6(3)

Table S10. Hydrogen coordinates (x 10^4) and isotropic displacement parameters ($Å^2$x 10^{3}) for **5**.

	x	y	z	U(eq)
H(1A)	10304	1982	4744	50
H(2B)	7416	2883	4558	42
H(1B)	11343	2418	6096	86
H(1C)	12477	3398	6249	86
H(1D)	11375	4133	6372	86
H(5A)	7422	1704	1919	54
H(6A)	8238	3032	1045	69
H(7A)	9999	4284	1732	68
H(8A)	10991	4197	3271	58
H(9A)	5718	2510	2894	53
H(9B)	6477	3973	2937	53
H(10A)	5962	4855	4169	58
H(10B)	4889	4805	3238	58
H(12A)	5949	3215	5386	82
H(13A)	4940	1606	5993	120
H(14A)	3123	667	5072	130
H(15A)	2380	1231	3564	125
H(16A)	3386	2830	2967	86

^{1}H NMR of 1a

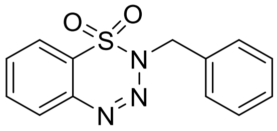

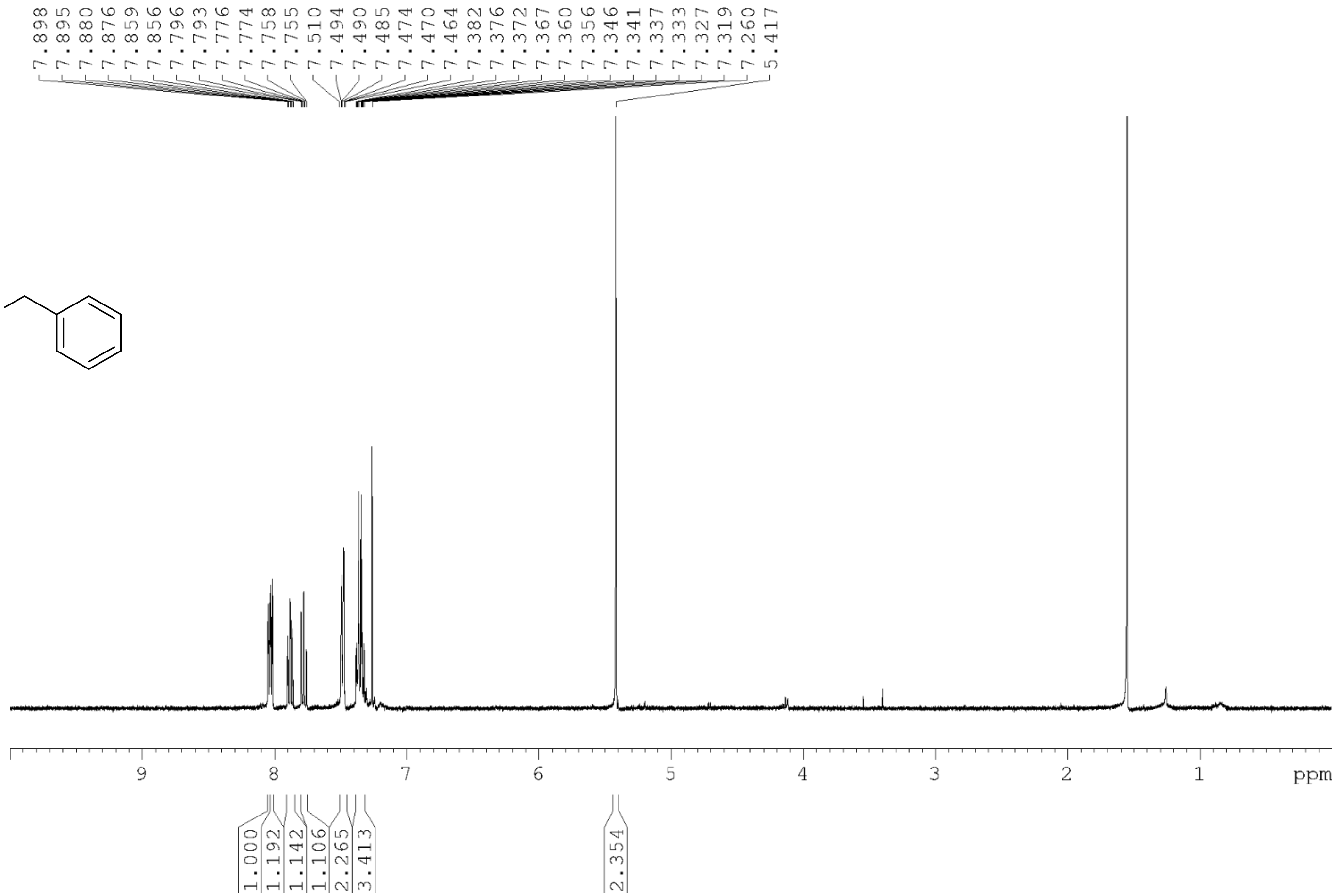

7.898
7.895
7.880
7.876
7.859
7.856
7.796
7.793
7.776
7.774
7.758
7.755
7.510
7.494
7.490
7.485
7.474
7.470
7.464
7.382
7.376
7.372
7.367
7.360
7.356
7.346
7.341
7.337
7.333
7.327
7.319
7.260
5.417
9
8
7
6
5
4
3
2
1
ppm
1.000
1.192
1.142
1.106
2.265
3.413
2.354

^{13}C NMR of 1a

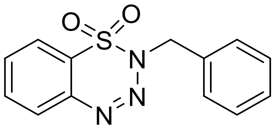

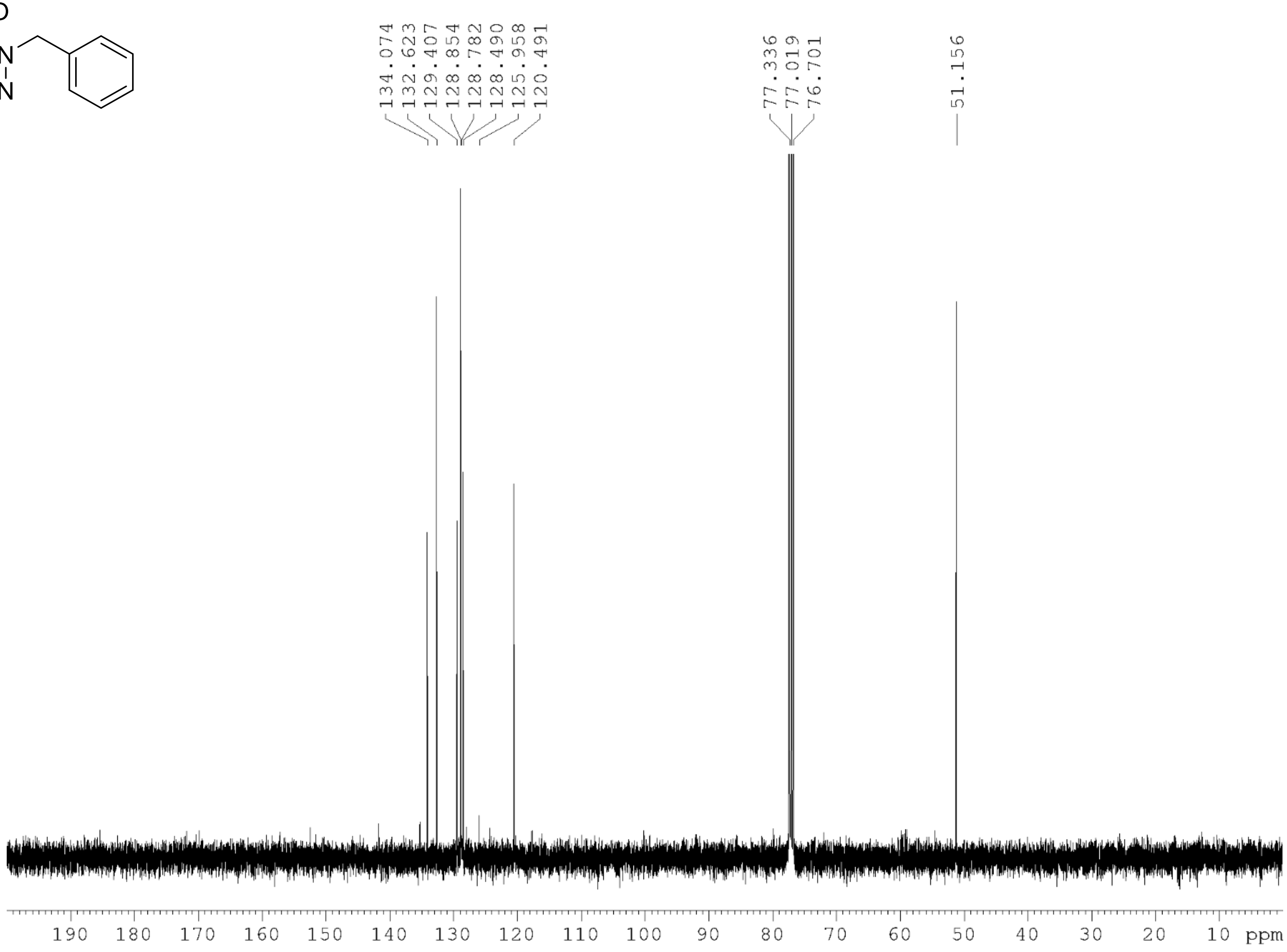

134.074
132.623
129.407
128.854
128.782
128.490
125.958
120.491
77.336
77.019
76.701
51.156
190 180 170 160 150 140 130 120 110 100 90 80 70 60 50 40 30 20 10 ppm

^{1}H NMR of 1b

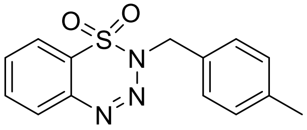

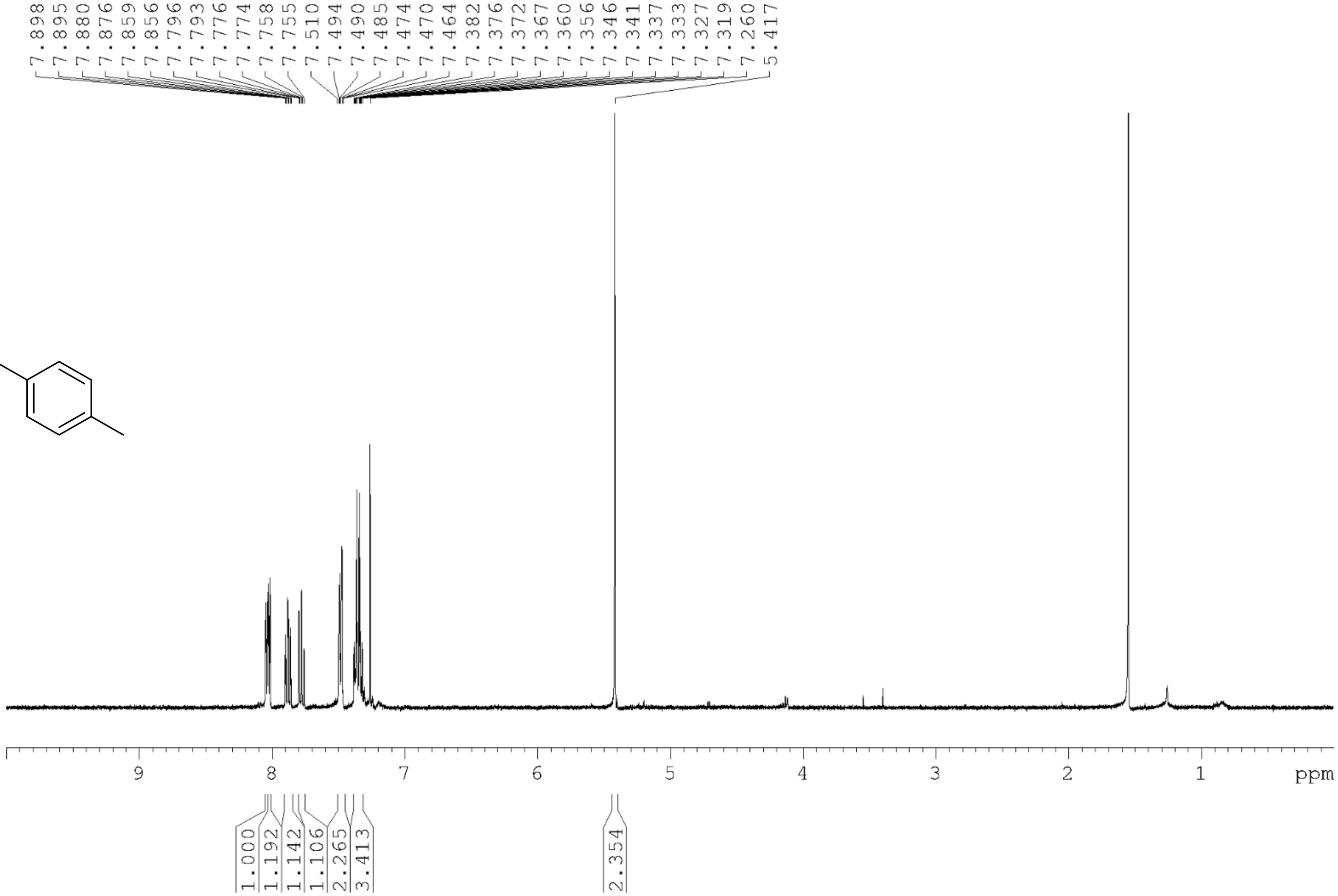
7.898
7.895
7.880
7.876
7.859
7.856
7.796
7.793
7.776
7.774
7.758
7.755
7.510
7.494
7.490
7.485
7.474
7.470
7.464
7.382
7.376
7.372
7.367
7.360
7.356
7.346
7.341
7.337
7.333
7.327
7.319
7.260
5.417
9
8
7
6
5
4
3
2
1
ppm
1.000
1.192
1.142
1.106
2.265
3.413
2.354

^{13}C NMR of 1b

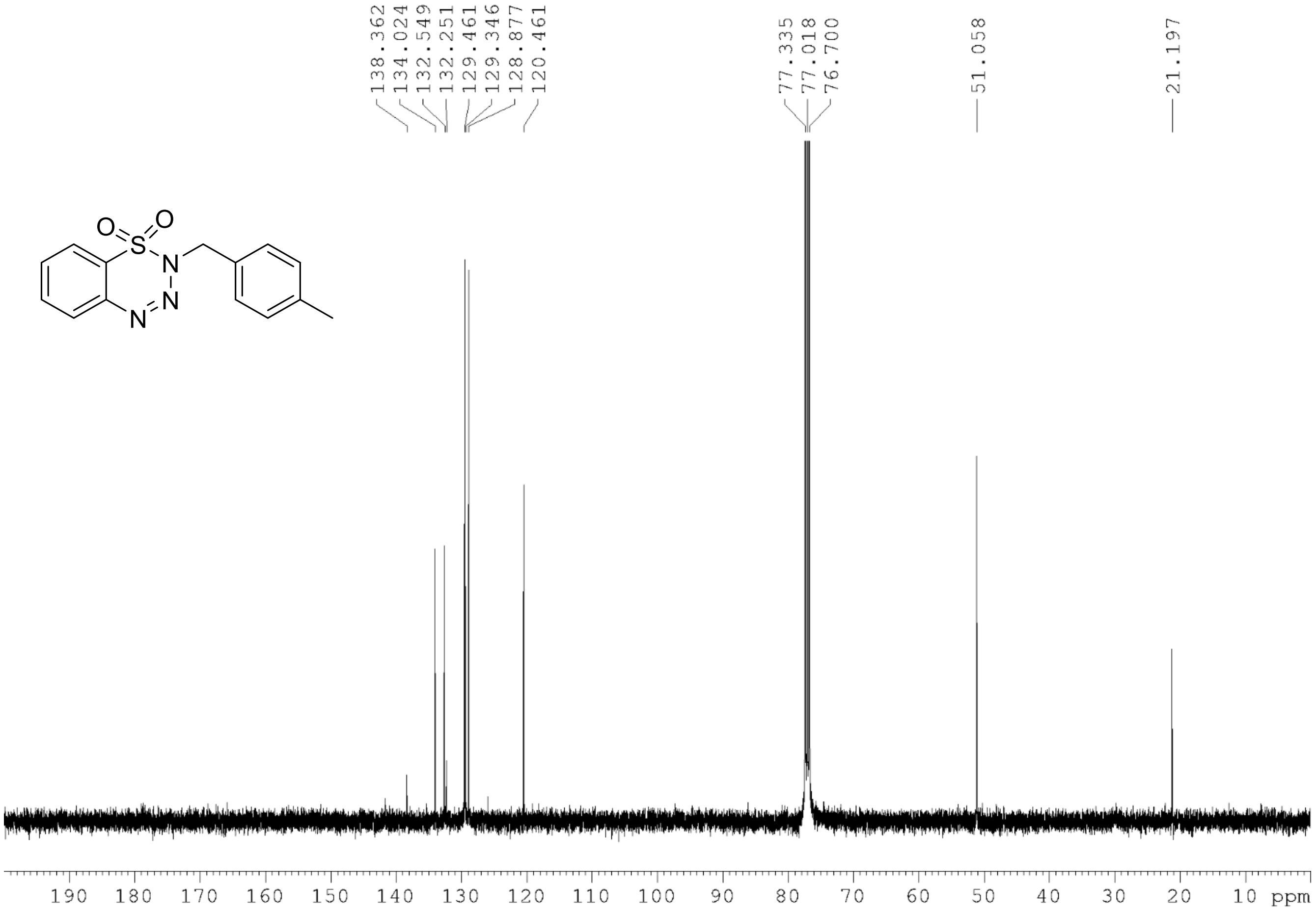
138.362
134.024
132.549
132.251
129.461
129.346
128.877
120.461
77.335
77.018
76.700
51.058
21.197
190 180 170 160 150 140 130 120 110 100 90 80 70 60 50 40 30 20 10 ppm

^{1}H NMR of 1c

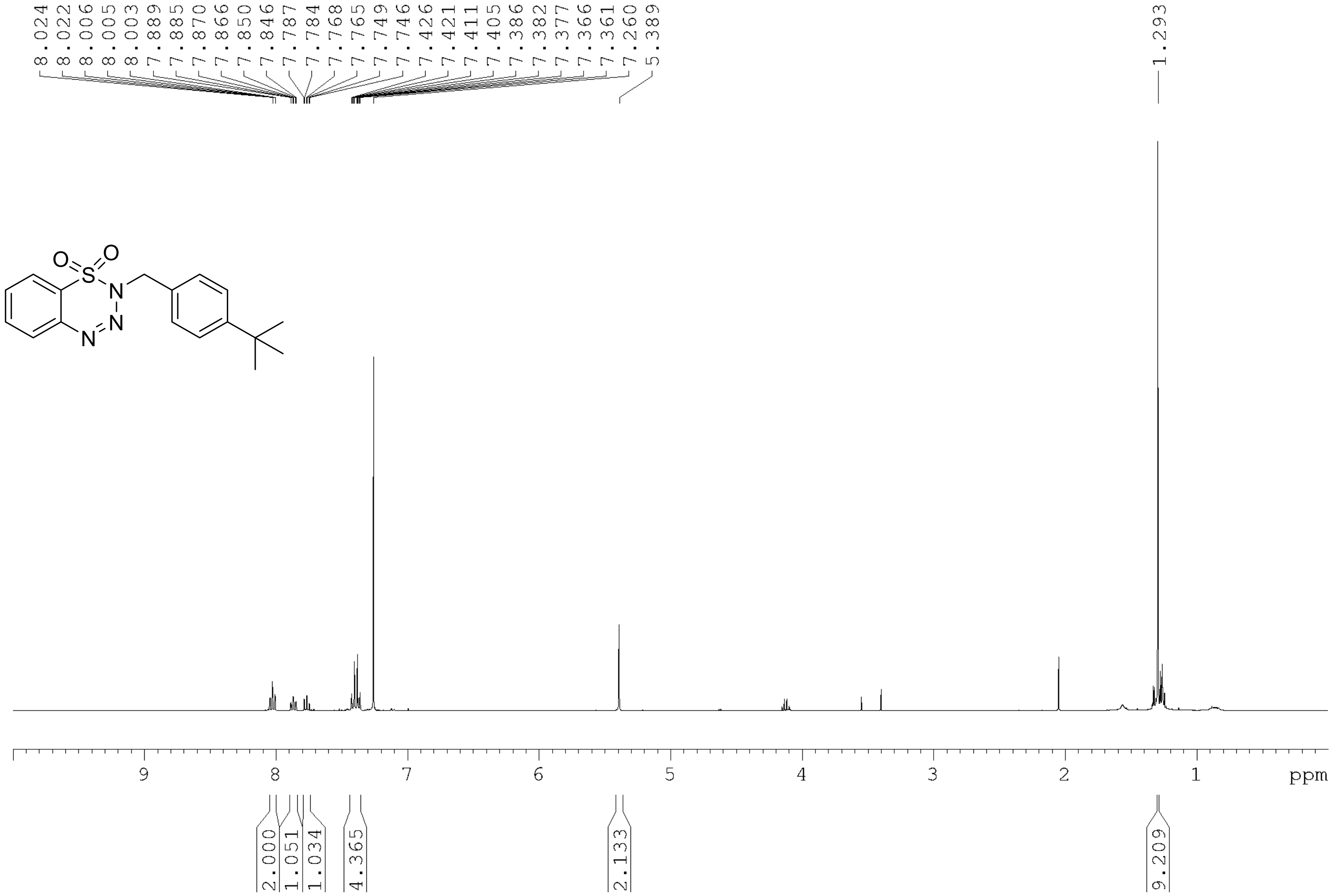
O
O
S
N
N
N
8.024
8.022
8.006
8.005
8.003
7.889
7.885
7.870
7.866
7.850
7.846
7.787
7.784
7.768
7.765
7.749
7.746
7.426
7.421
7.411
7.405
7.386
7.382
7.377
7.366
7.361
7.260
5.389
1.293
9
8
7
6
5
4
3
2
1
ppm
2.000
1.051
1.034
4.365
2.133
9.209

^{13}C NMR of 1c

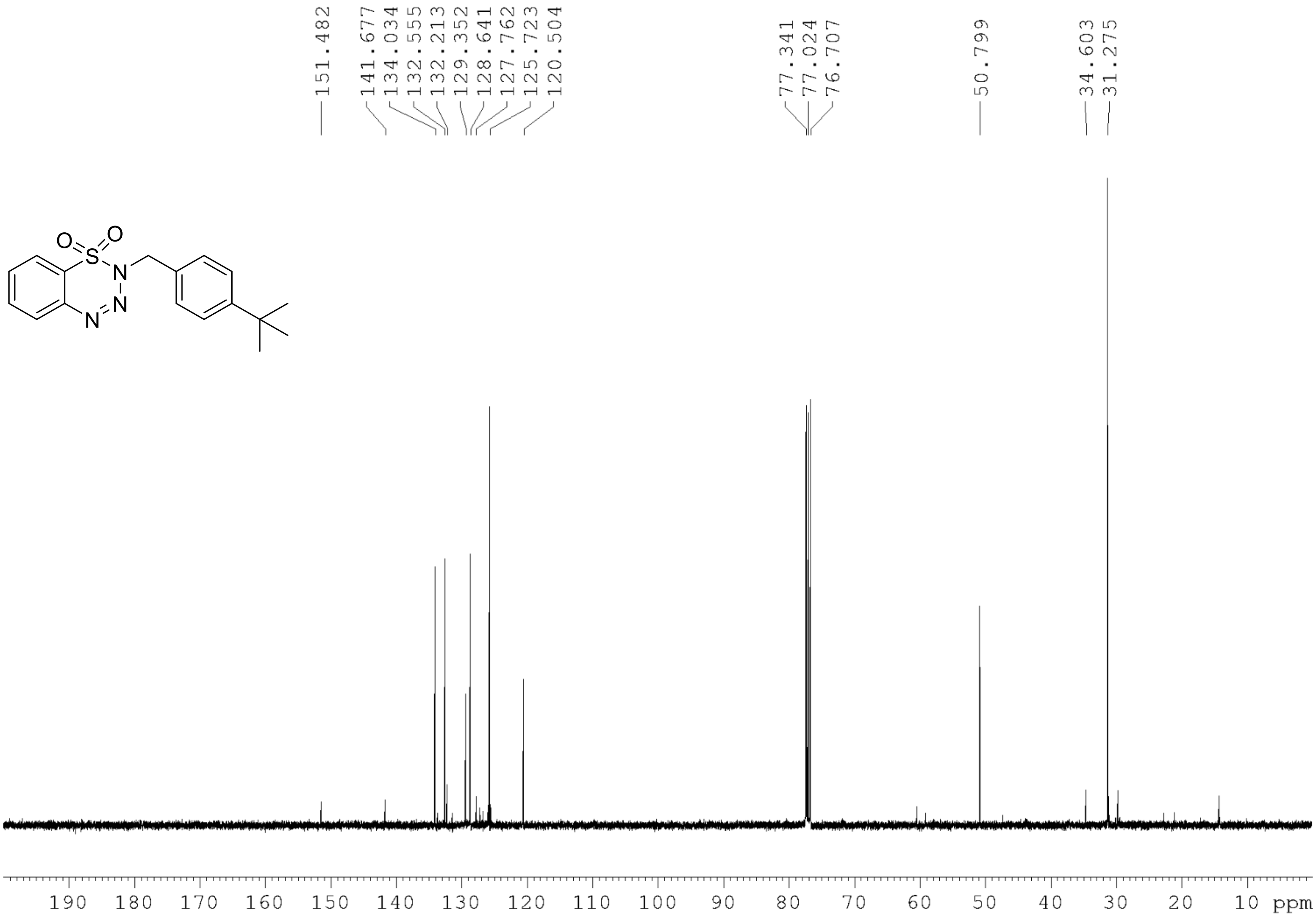
151.482
141.677
134.034
132.555
132.213
129.352
128.641
127.762
125.723
120.504
77.341
77.024
76.707
50.799
34.603
31.275
O
O
S
N
N
N
190 180 170 160 150 140 130 120 110 100 90 80 70 60 50 40 30 20 10 ppm

^{1}H NMR of 1d

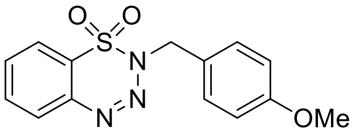

O
O
S
N
N
N
OMe

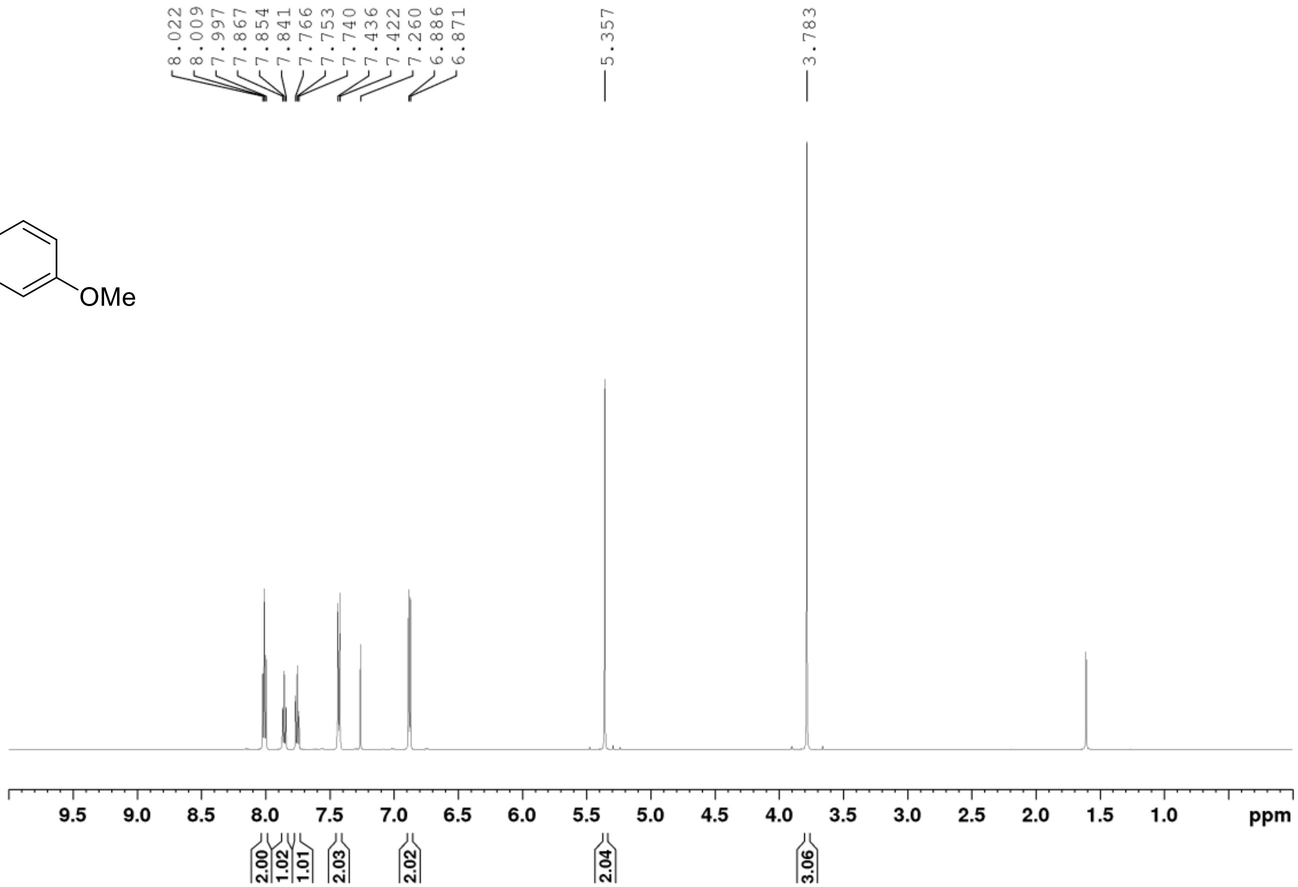

8.022
8.009
7.997
7.867
7.854
7.841
7.766
7.753
7.740
7.436
7.422
7.260
6.886
6.871
5.357
3.783
9.5
9.0
8.5
8.0
7.5
7.0
6.5
6.0
5.5
5.0
4.5
4.0
3.5
3.0
2.5
2.0
1.5
1.0
ppm
2.00
1.02
1.01
2.03
2.02
2.04
3.06

^{13}C NMR of 1d

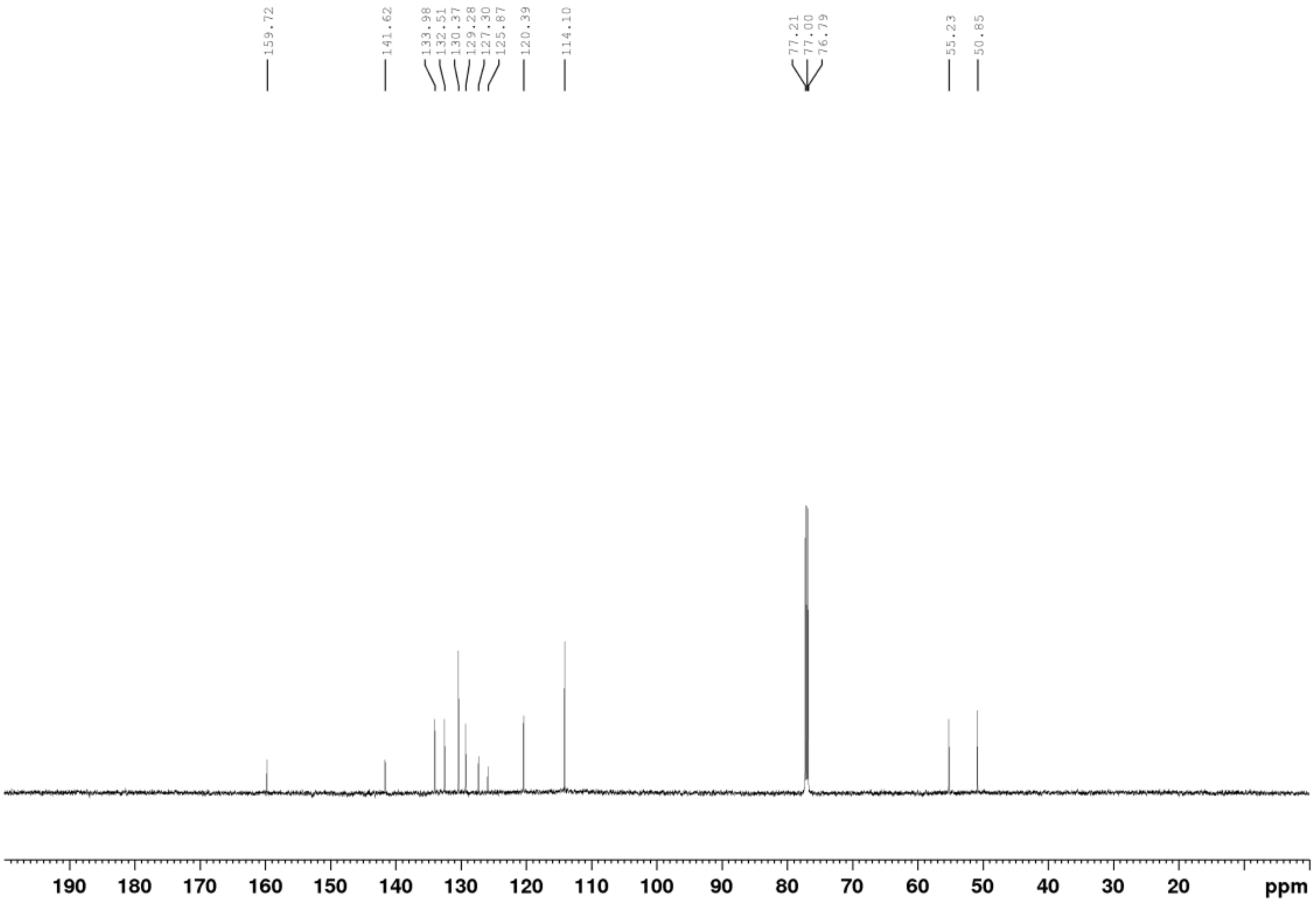

159.72
141.62
133.98
132.51
130.37
129.28
127.30
125.87
120.39
114.10
77.21
77.00
76.79
55.23
50.85
190
180
170
160
150
140
130
120
110
100
90
80
70
60
50
40
30
20
ppm

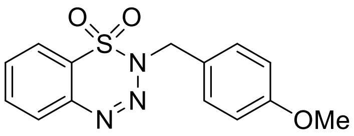
O
O
S
N
N
N
OMe

^{1}H NMR of 1e

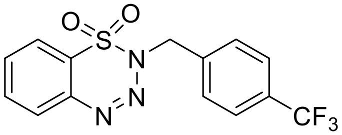

O
O
S
N
N
N
CF3

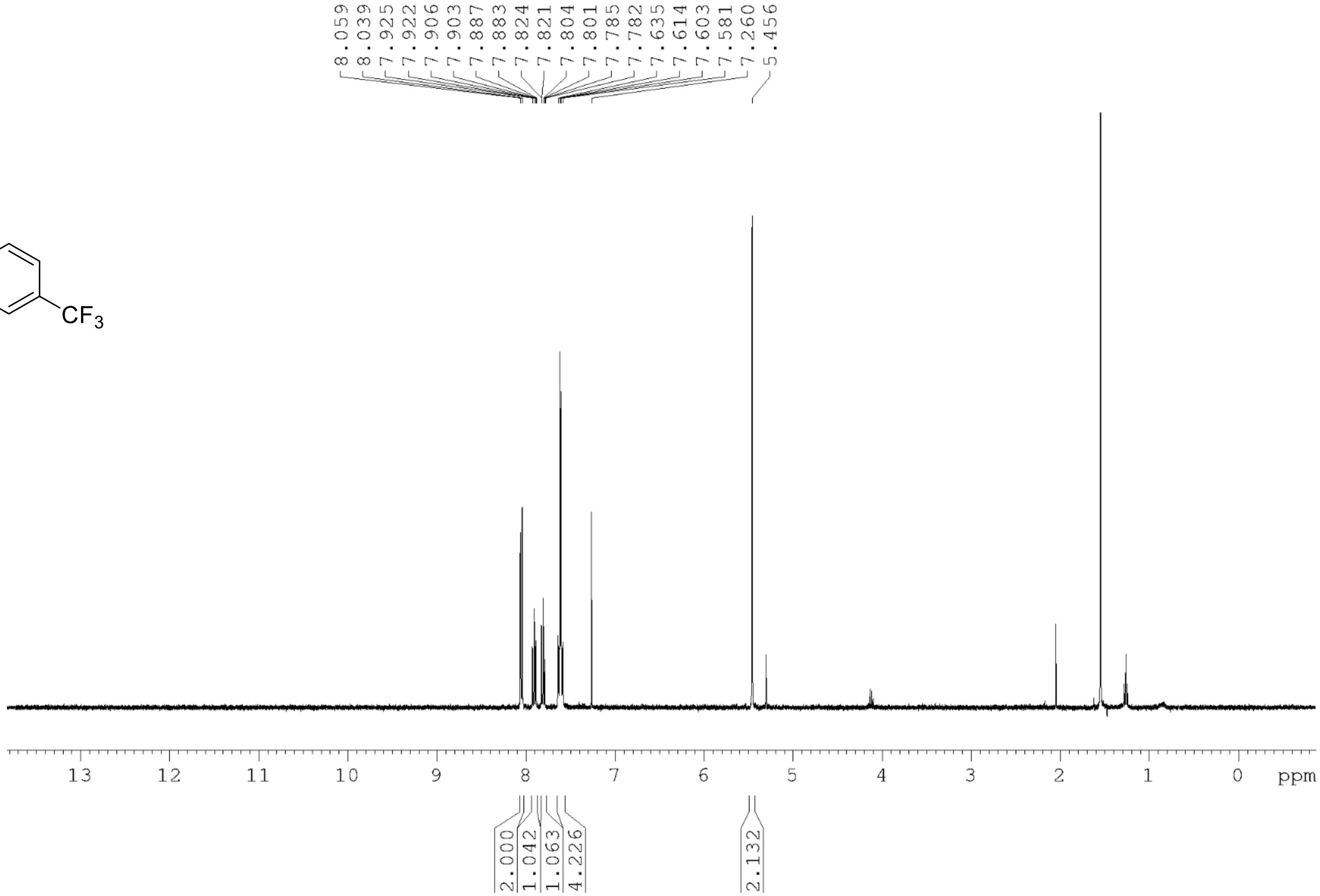

8.059
8.039
7.925
7.922
7.906
7.903
7.887
7.883
7.824
7.821
7.804
7.801
7.785
7.782
7.635
7.614
7.603
7.581
7.260
5.456
13
12
11
10
9
8
7
6
5
4
3
2
1
0
ppm
2.000
1.042
1.063
4.226
2.132

^{13}C NMR of 1e

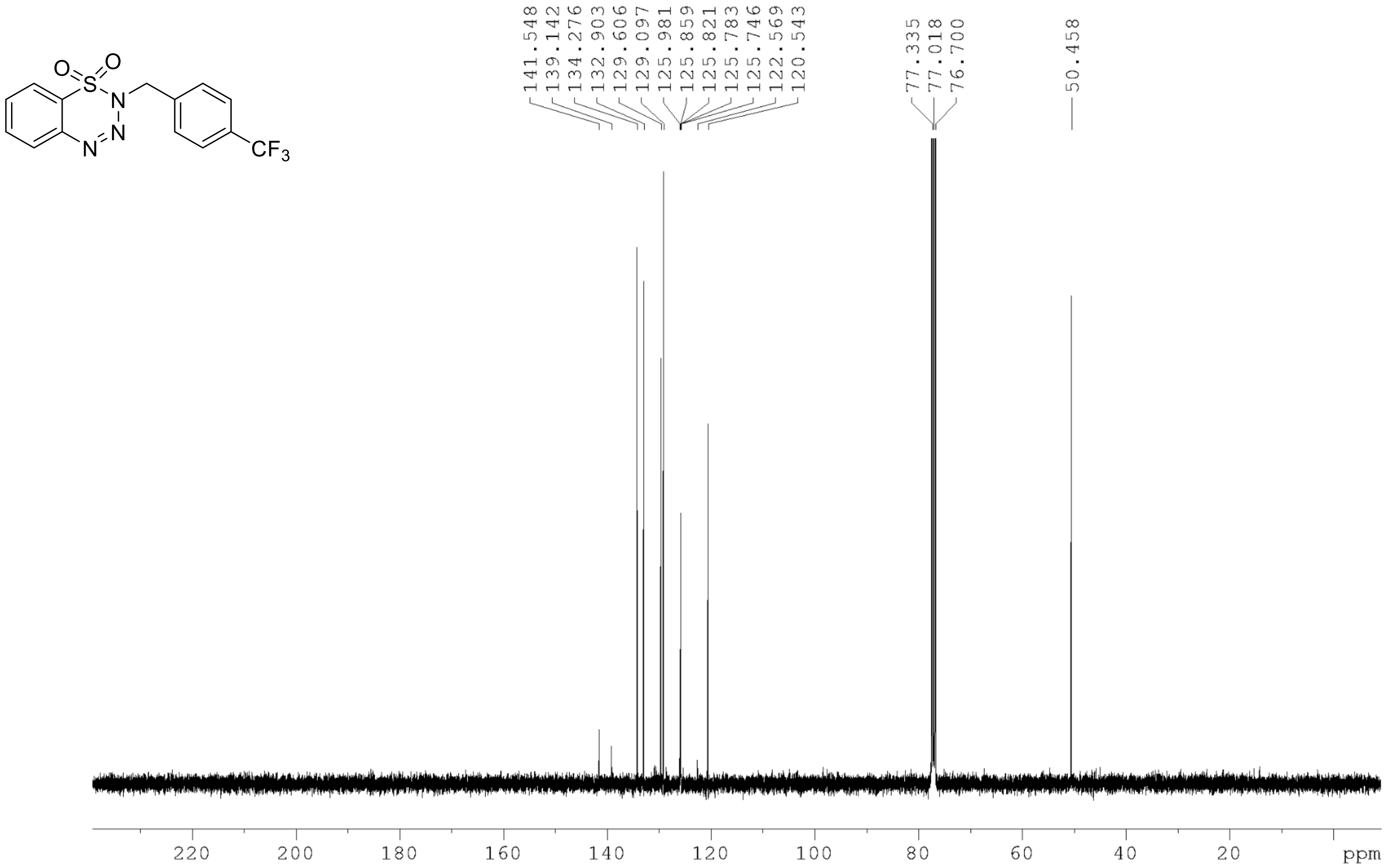
O
O
S
N
N
N
CF3
141.548
139.142
134.276
132.903
129.606
129.097
125.981
125.859
125.821
125.783
125.746
122.569
120.543
77.335
77.018
76.700
50.458
220
200
180
160
140
120
100
80
60
40
20
ppm

^{1}H NMR of 1f

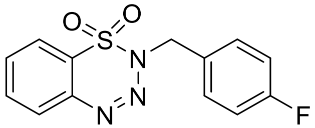

O
O
S
N
N
N
F

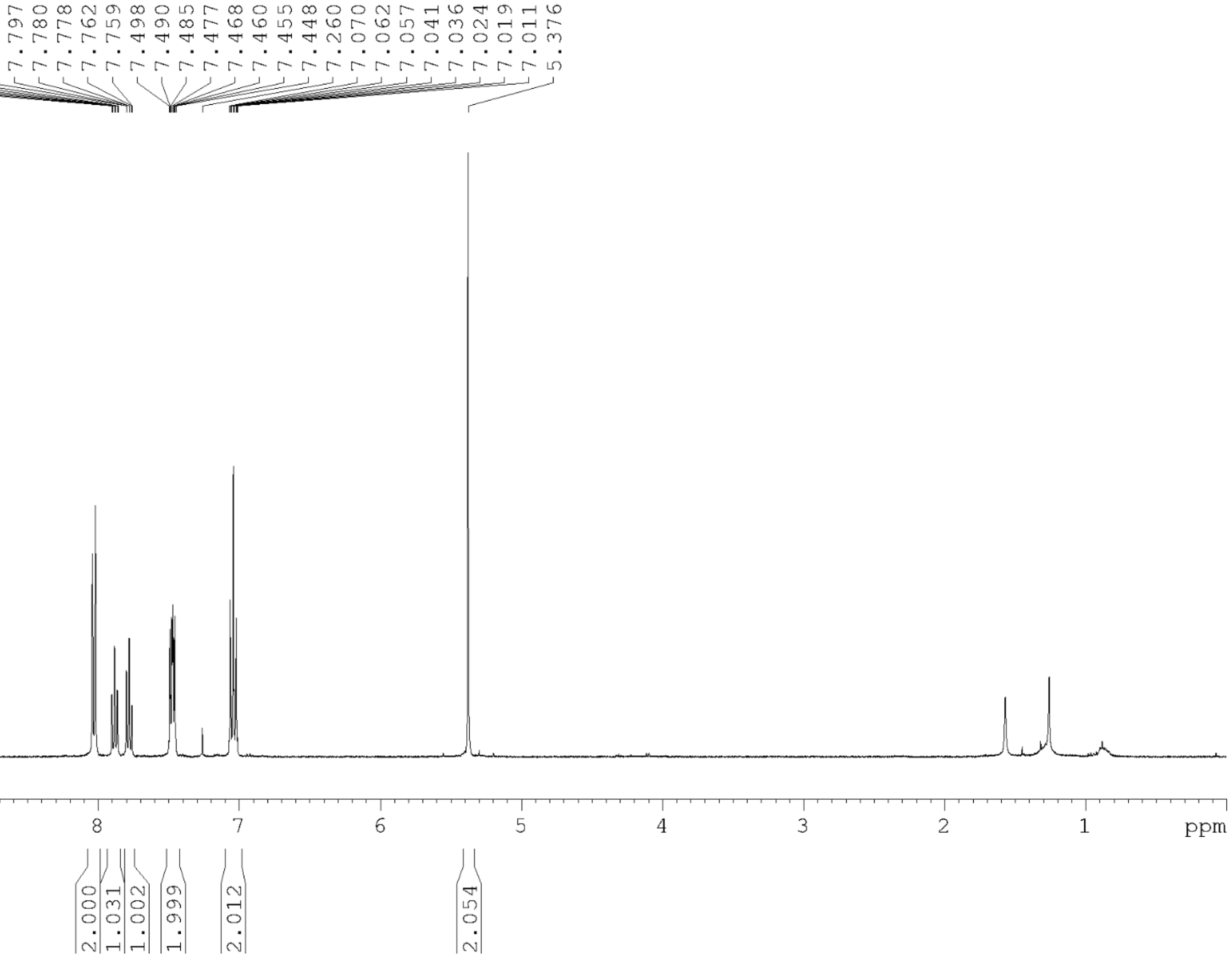

7.902
7.898
7.883
7.880
7.863
7.859
7.800
7.797
7.780
7.778
7.762
7.759
7.498
7.490
7.485
7.477
7.468
7.460
7.455
7.448
7.260
7.070
7.062
7.057
7.041
7.036
7.024
7.019
7.011
5.376
9
8
7
6
5
4
3
2
1
ppm
2.000
1.031
1.002
1.999
2.012
2.054

^{13}C NMR of 1f

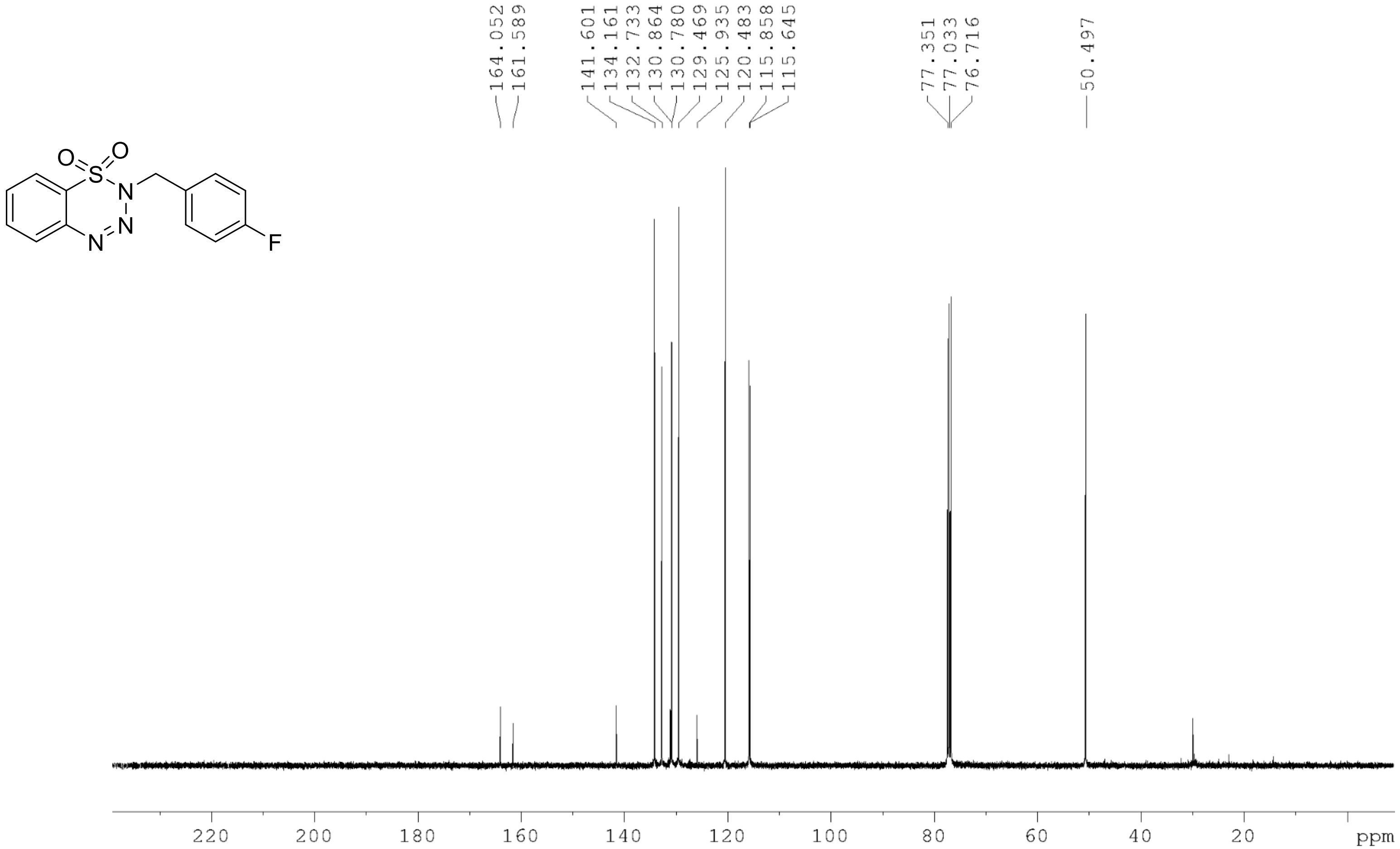
164.052
161.589
141.601
134.161
132.733
130.864
130.780
129.469
125.935
120.483
115.858
115.645
77.351
77.033
76.716
50.497
220
200
180
160
140
120
100
80
60
40
20
ppm

^{1}H NMR of 1g

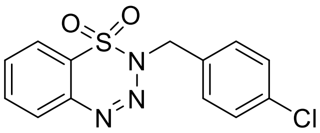

O
O
S
N
N
N
Cl

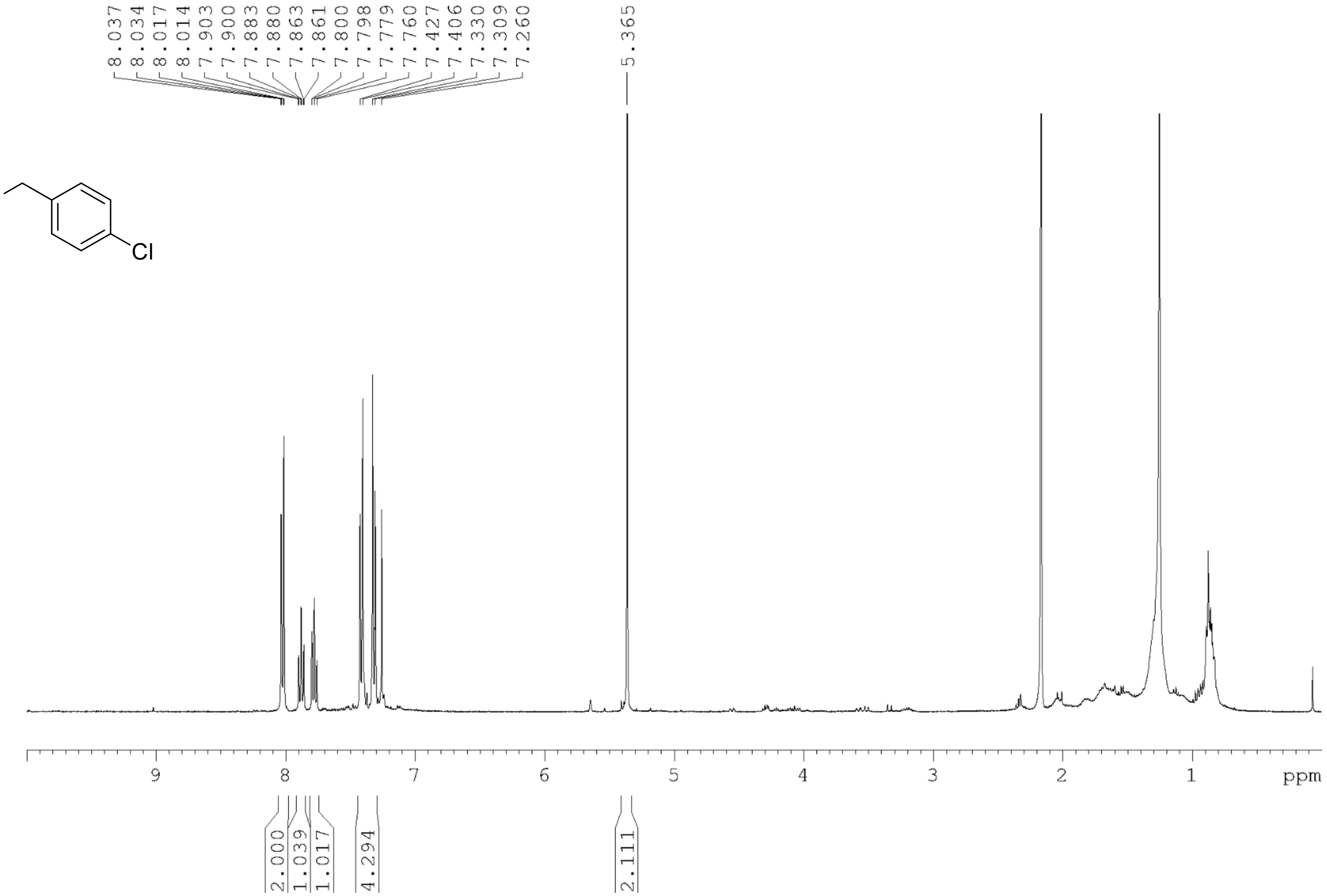

8.037
8.034
8.017
8.014
7.903
7.900
7.883
7.880
7.863
7.861
7.800
7.798
7.779
7.760
7.427
7.406
7.330
7.309
7.260
5.365
9
8
7
6
5
4
3
2
1
ppm
2.000
1.039
1.017
4.294
2.111

^{13}C NMR of 1g

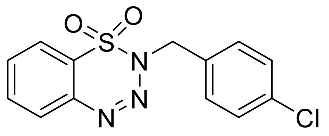

O
O
S
N
N
N
Cl

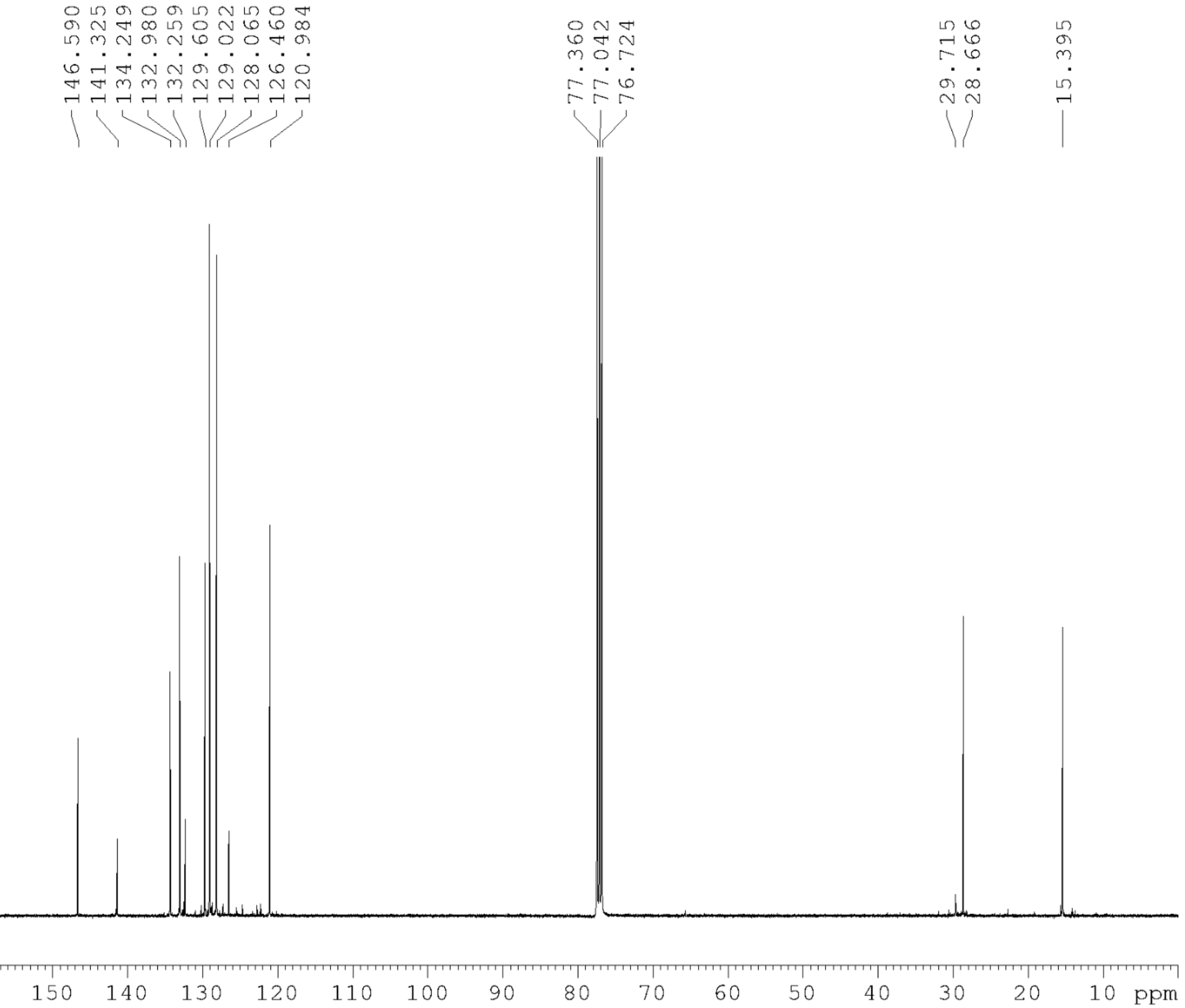

146.590
141.325
134.249
132.980
132.259
129.605
129.022
128.065
126.460
120.984
77.360
77.042
76.724
29.715
28.666
15.395
190
180
170
160
150
140
130
120
110
100
90
80
70
60
50
40
30
20
10
ppm

^{1}H NMR of 1h

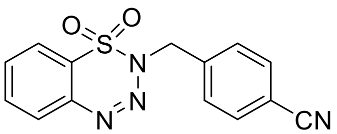
O
O
S
N
N
N
CN

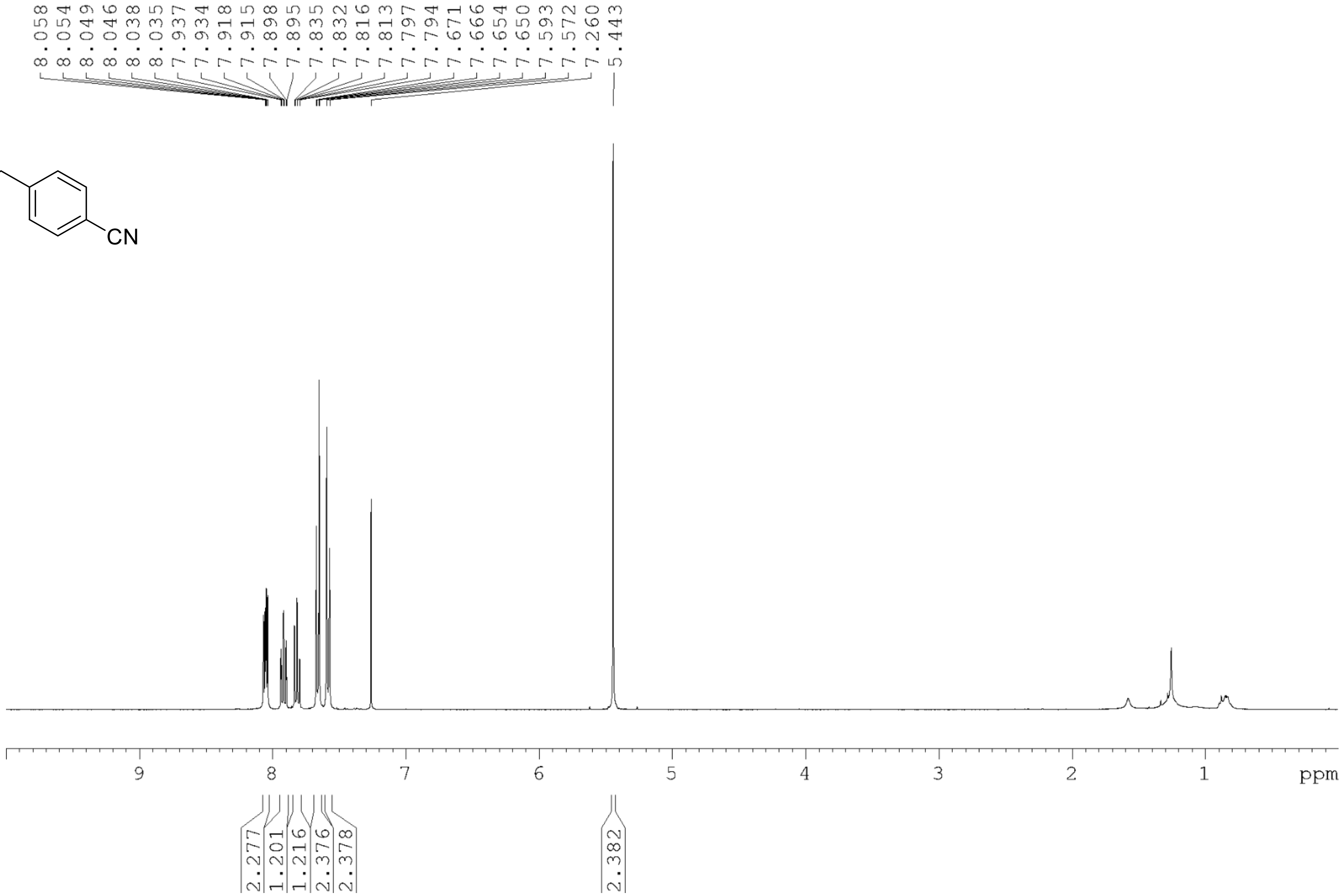
8.058
8.054
8.049
8.046
8.038
8.035
7.937
7.934
7.918
7.915
7.898
7.895
7.835
7.832
7.816
7.813
7.797
7.794
7.671
7.666
7.654
7.650
7.593
7.572
7.260
5.443
9
8
7
6
5
4
3
2
1
ppm
2.277
1.201
1.216
2.376
2.378
2.382

^{13}C NMR of 1h

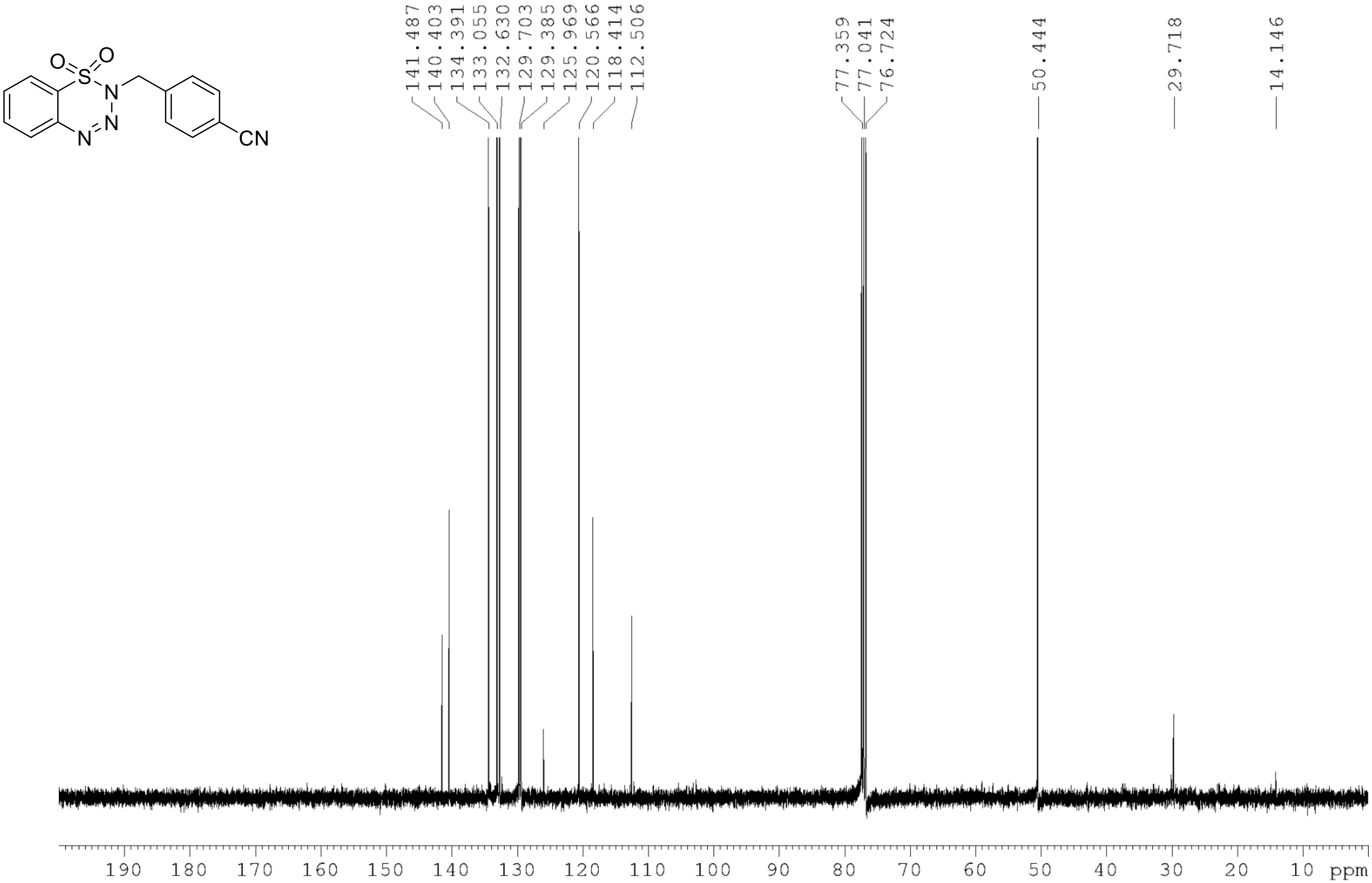
O
O
S
N
N
N
CN
141.487
140.403
134.391
133.055
132.630
129.703
129.385
125.969
120.566
118.414
112.506
77.359
77.041
76.724
50.444
29.718
14.146
190 180 170 160 150 140 130 120 110 100 90 80 70 60 50 40 30 20 10 ppm

^{1}H NMR of 1i

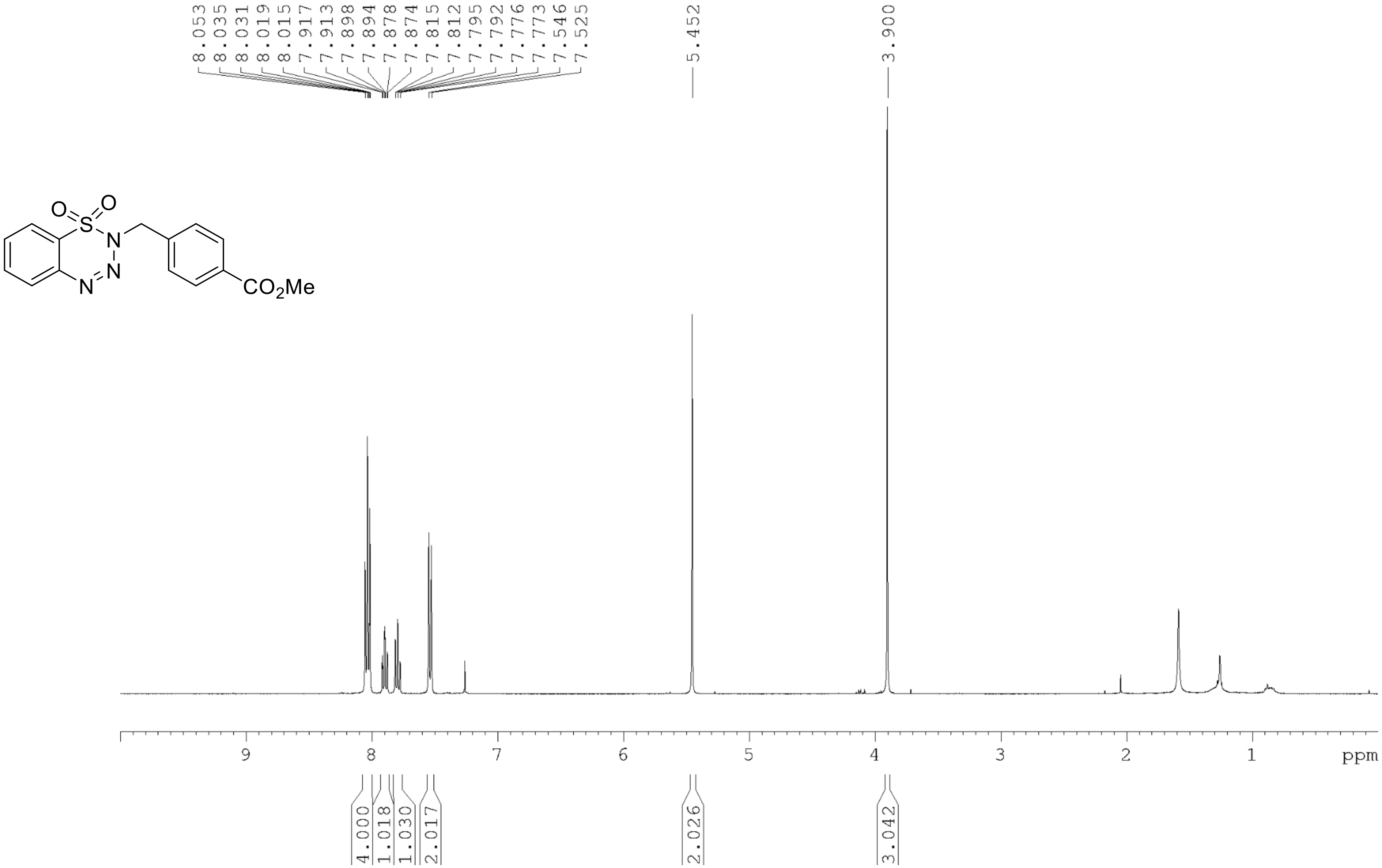

O
O
S
N
N
N
CO2Me
8.053 8.035 8.031 8.019 8.015 7.917 7.913 7.898 7.894 7.878 7.874 7.815 7.812 7.795 7.792 7.776 7.773 7.546 7.525
5.452
3.900
9 8 7 6 5 4 3 2 1 ppm
4.000 1.018 1.030 2.017
2.026
3.042

^{13}C NMR of 1i

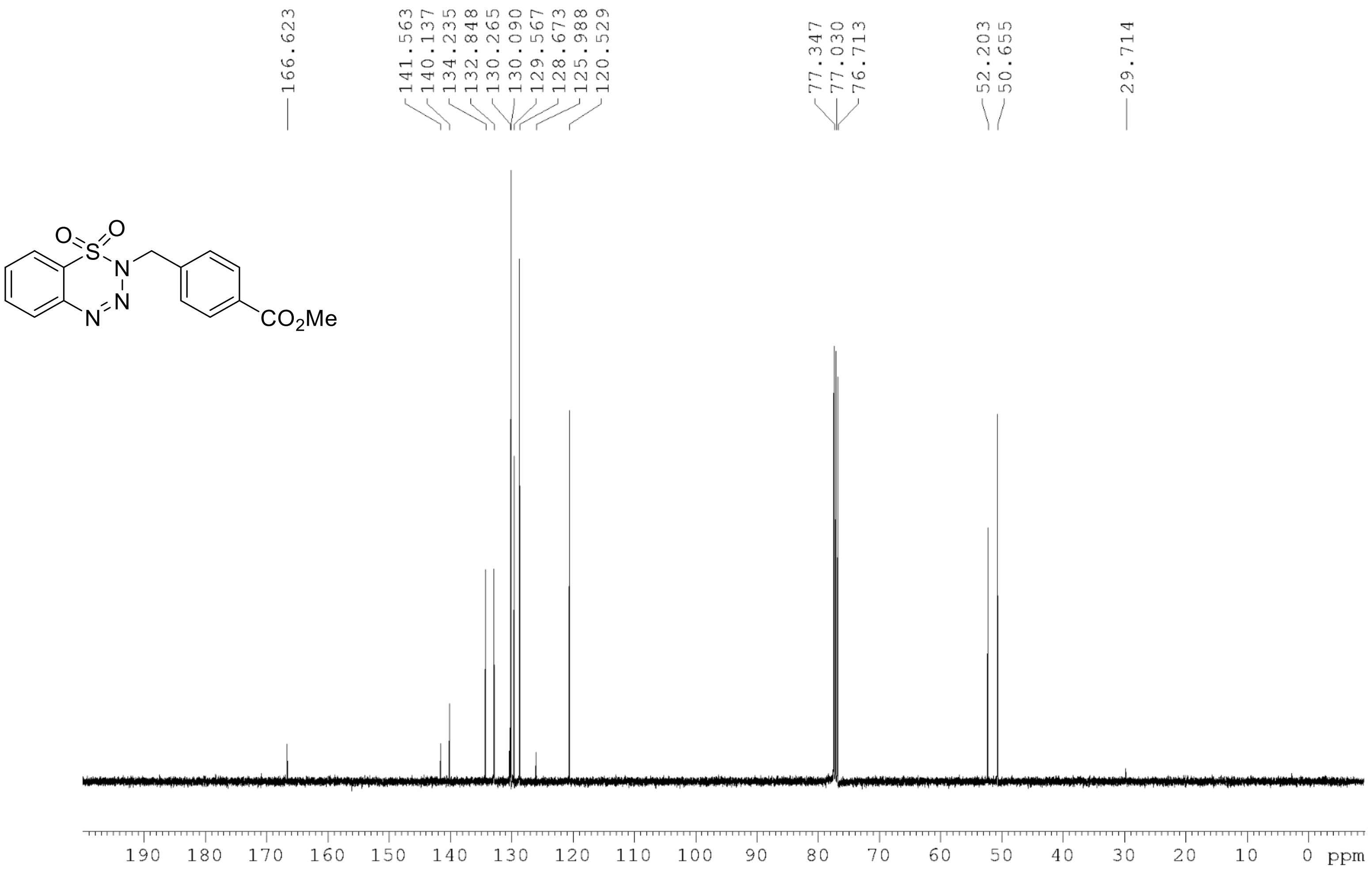

166.623
141.563
140.137
134.235
132.848
130.265
130.090
129.567
128.673
125.988
120.529
77.347
77.030
76.713
52.203
50.655
29.714
190 180 170 160 150 140 130 120 110 100 90 80 70 60 50 40 30 20 10 0 ppm

^{1}H NMR of 1j

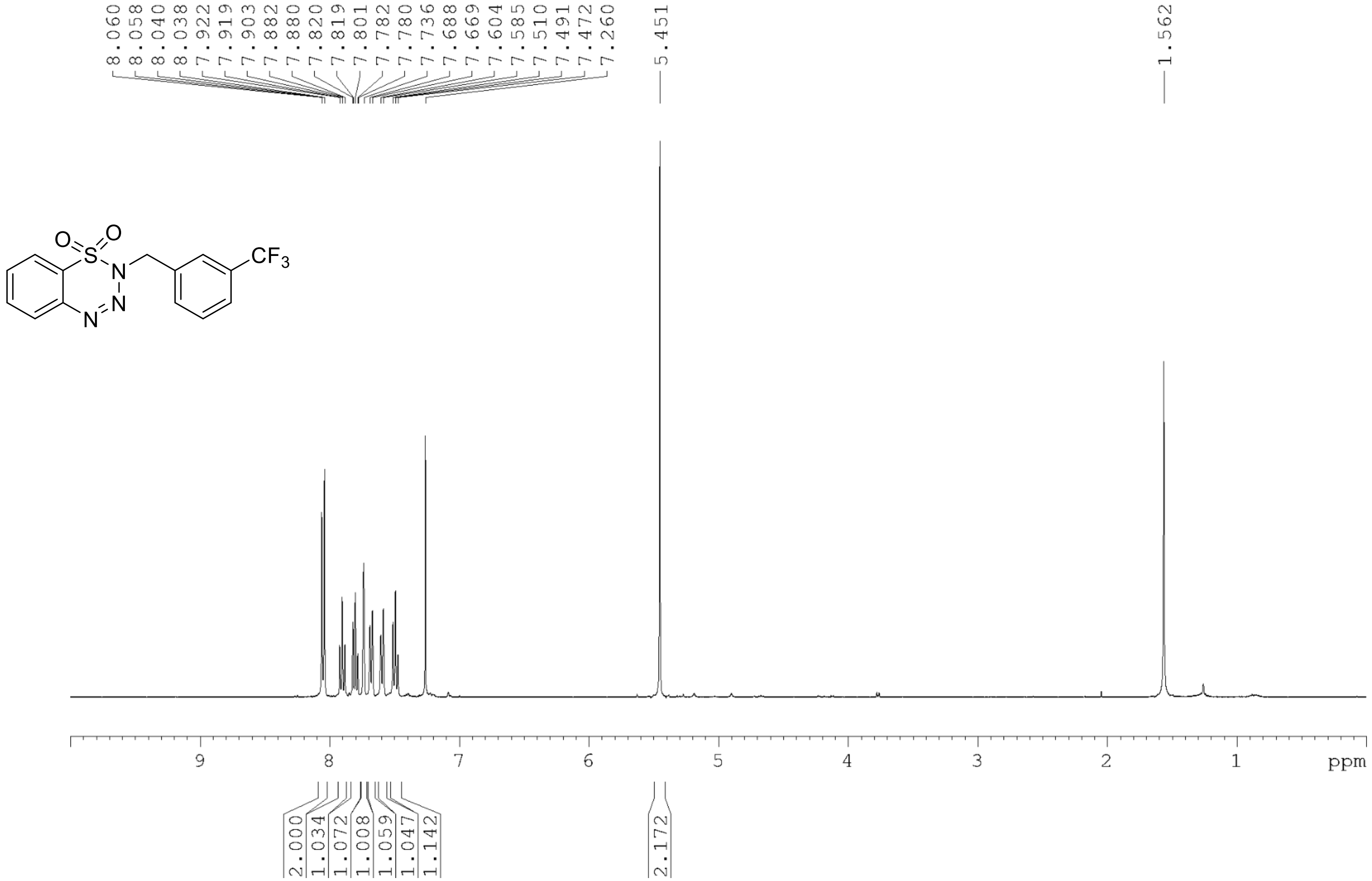
8.060
8.058
8.040
8.038
7.922
7.919
7.903
7.882
7.880
7.820
7.819
7.801
7.782
7.780
7.736
7.688
7.669
7.604
7.585
7.510
7.491
7.472
7.260
5.451
1.562
9
8
7
6
5
4
3
2
1
ppm
2.000
1.034
1.072
1.008
1.059
1.047
1.142
2.172
O
S
N
N
N
CF3

^{13}C NMR of 1j

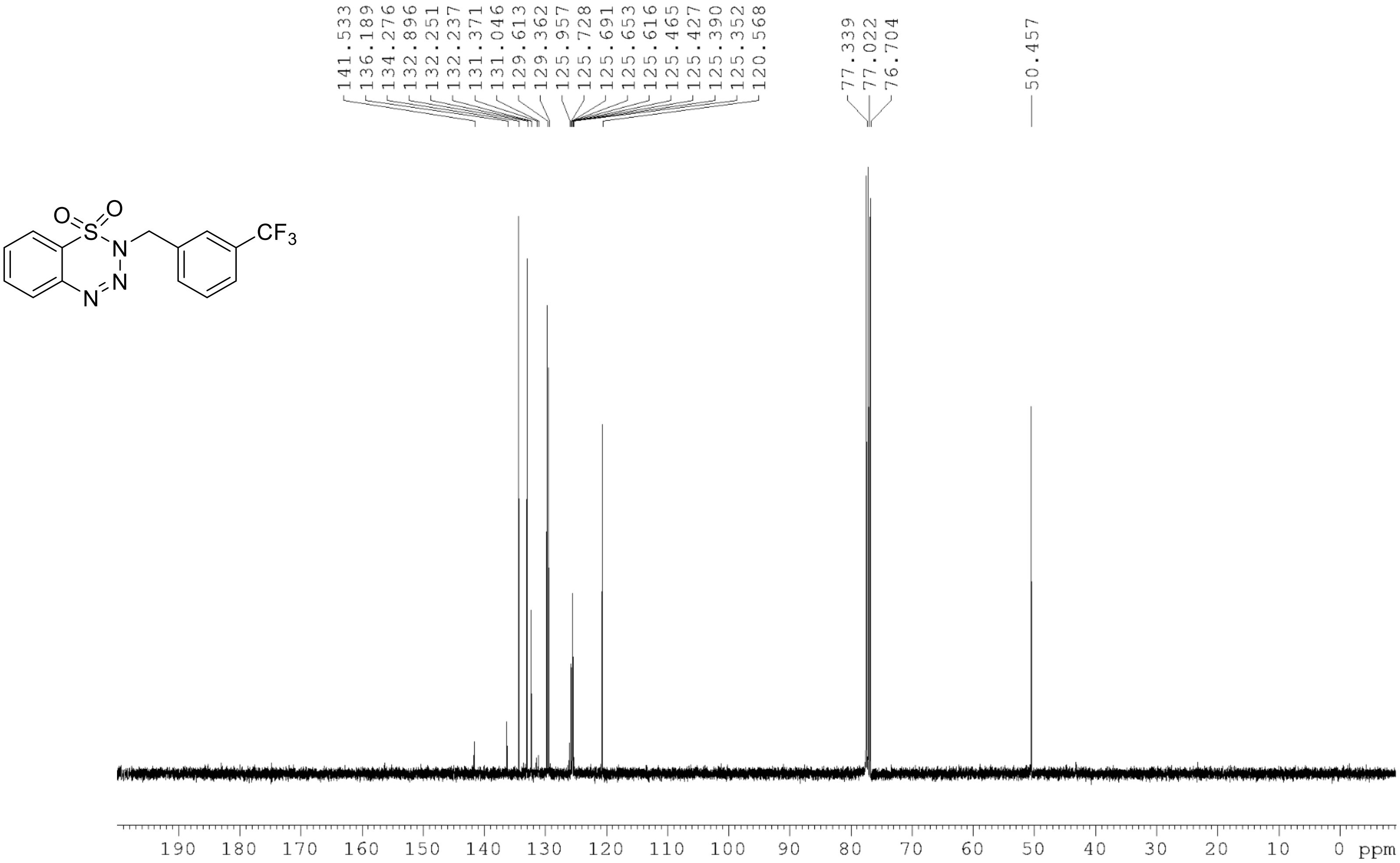

O
O
S
N
N
N
CF3
141.533
136.189
134.276
132.896
132.251
132.237
131.371
131.046
129.613
129.362
125.957
125.728
125.691
125.653
125.616
125.465
125.427
125.390
125.352
120.568
77.339
77.022
76.704
50.457
190 180 170 160 150 140 130 120 110 100 90 80 70 60 50 40 30 20 10 0 ppm

^{1}H NMR of 1k

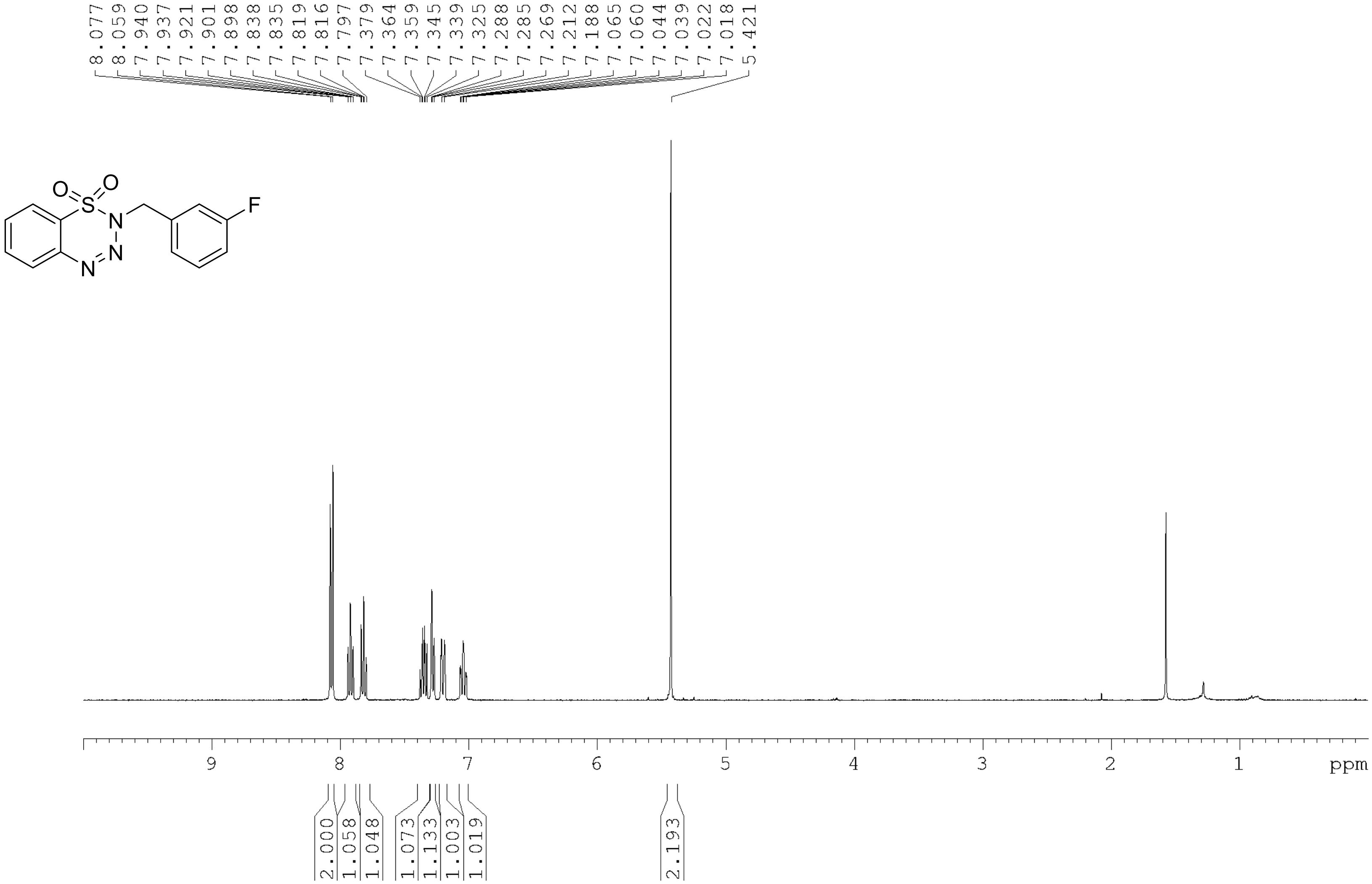

O
O
S
N
N
N
F
8.077
8.059
7.940
7.937
7.921
7.901
7.898
7.838
7.835
7.819
7.816
7.797
7.379
7.364
7.359
7.345
7.339
7.325
7.288
7.285
7.269
7.212
7.188
7.065
7.060
7.044
7.039
7.022
7.018
5.421
9
8
7
6
5
4
3
2
1
ppm
2.000
1.058
1.048
1.073
1.133
1.003
1.019
2.193

^{13}C NMR of 1k

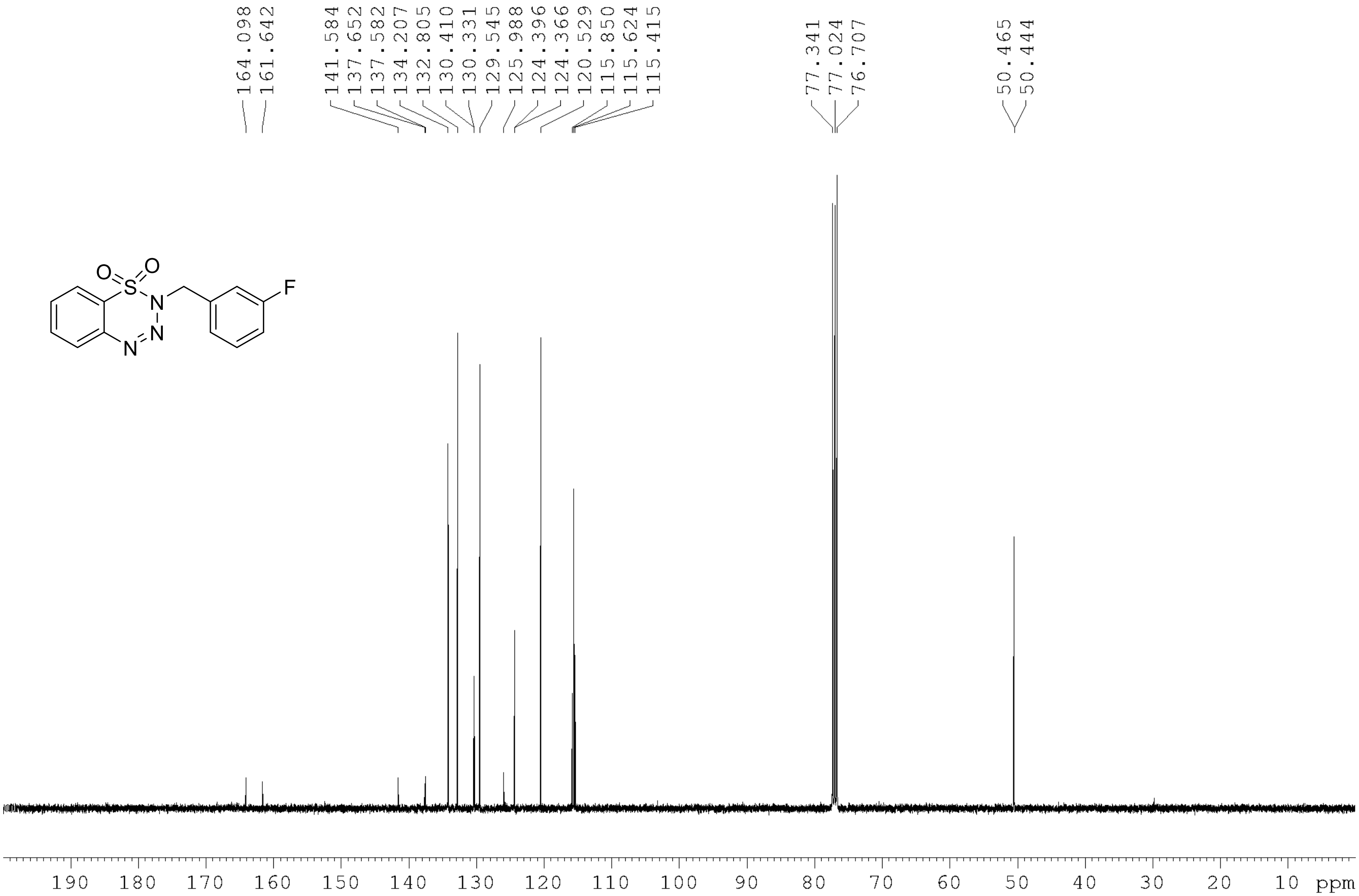
164.098
161.642
141.584
137.652
137.582
134.207
132.805
130.410
130.331
129.545
125.988
124.396
124.366
120.529
115.850
115.624
115.415
77.341
77.024
76.707
50.465
50.444
O
S
O
N
N
N
F
190 180 170 160 150 140 130 120 110 100 90 80 70 60 50 40 30 20 10 ppm

^{1}H NMR of 1l

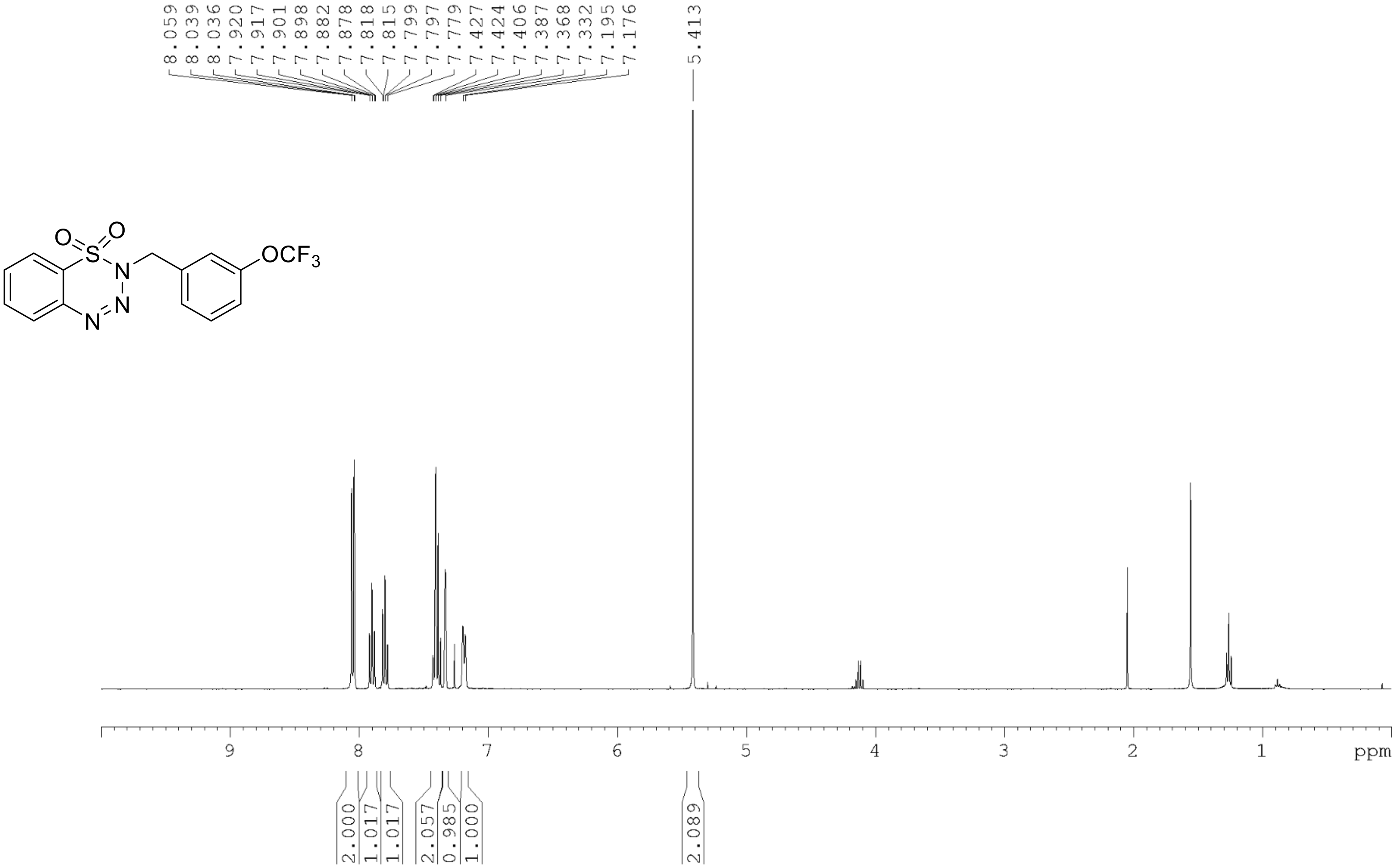

O
O
S
N
N
N
OCF3
8.059
8.039
8.036
7.920
7.917
7.901
7.898
7.882
7.878
7.818
7.815
7.799
7.797
7.779
7.427
7.424
7.406
7.387
7.368
7.332
7.195
7.176
5.413
9
8
7
6
5
4
3
2
1
ppm
2.000
1.017
1.017
2.057
0.985
1.000
2.089

^{13}C NMR of 1l

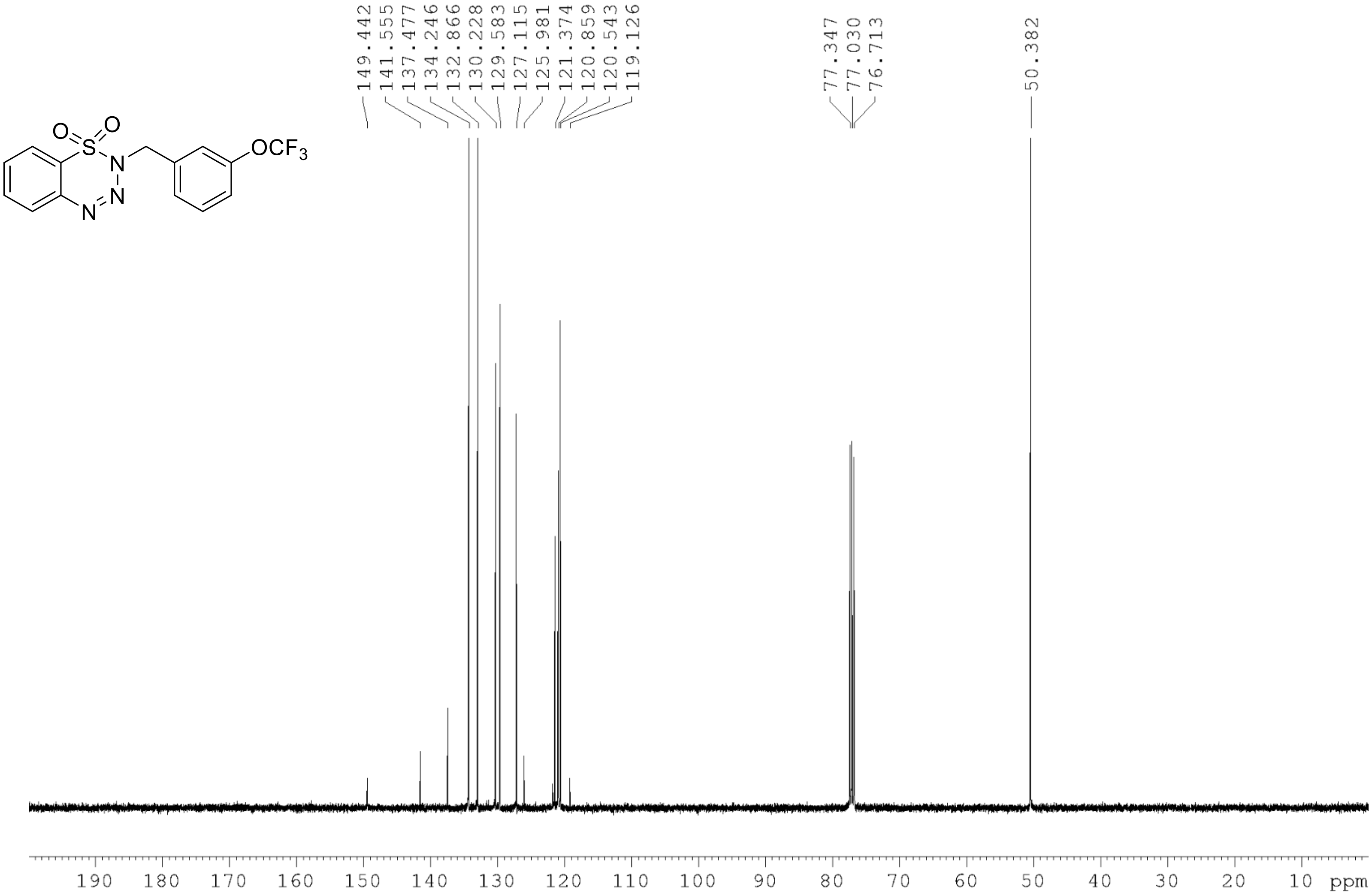

O
O
S
N
N
N
OCF3
149.442
141.555
137.477
134.246
132.866
130.228
129.583
127.115
125.981
121.374
120.859
120.543
119.126
77.347
77.030
76.713
50.382
190 180 170 160 150 140 130 120 110 100 90 80 70 60 50 40 30 20 10 ppm

^{1}H NMR of 1m

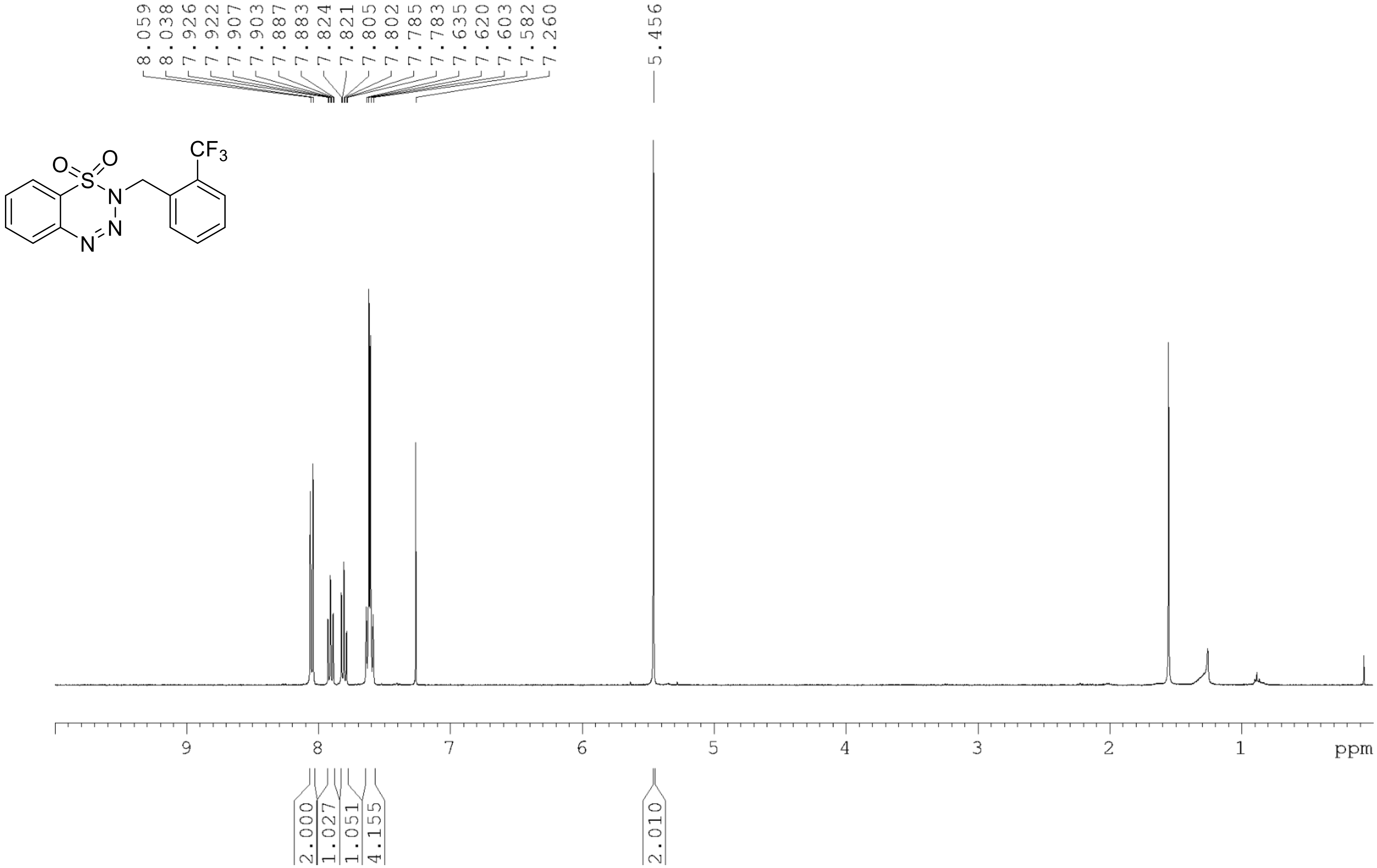

8.059
8.038
7.926
7.922
7.907
7.903
7.887
7.883
7.824
7.821
7.805
7.802
7.785
7.783
7.635
7.620
7.603
7.582
7.260
5.456
CF3
O
S
O
N
N
N
9
8
7
6
5
4
3
2
1
ppm
2.000
1.027
1.051
4.155
2.010

^{13}C NMR of 1m

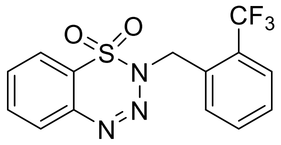

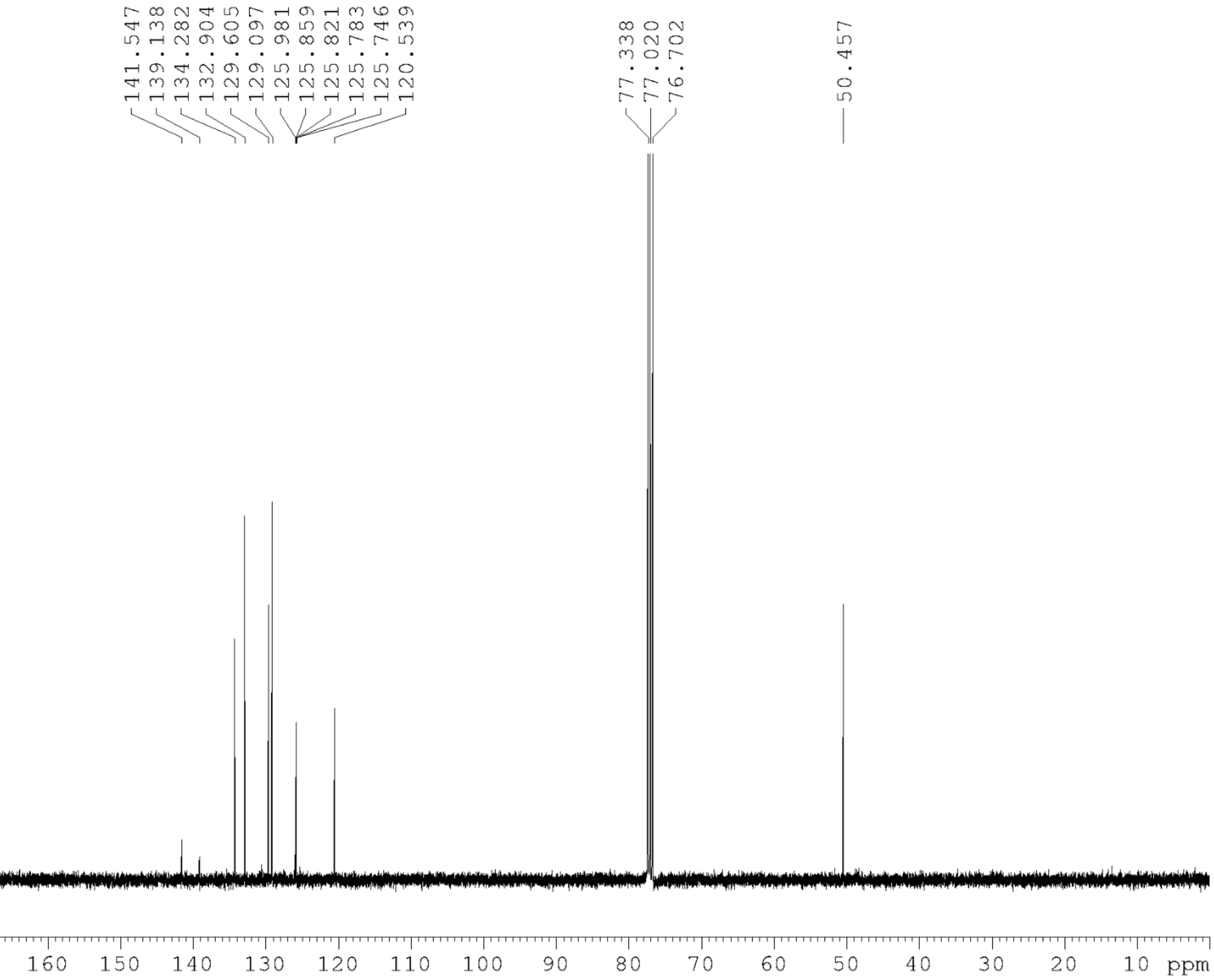
141.547
139.138
134.282
132.904
129.605
129.097
125.981
125.859
125.821
125.783
125.746
120.539
77.338
77.020
76.702
50.457
190 180 170 160 150 140 130 120 110 100 90 80 70 60 50 40 30 20 10 ppm

^{13}H NMR of 1n

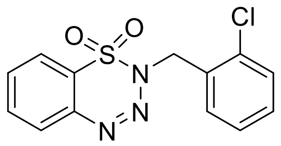

^{13}C NMR of 1n

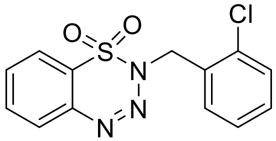

O
O
S
N
N
N
Cl

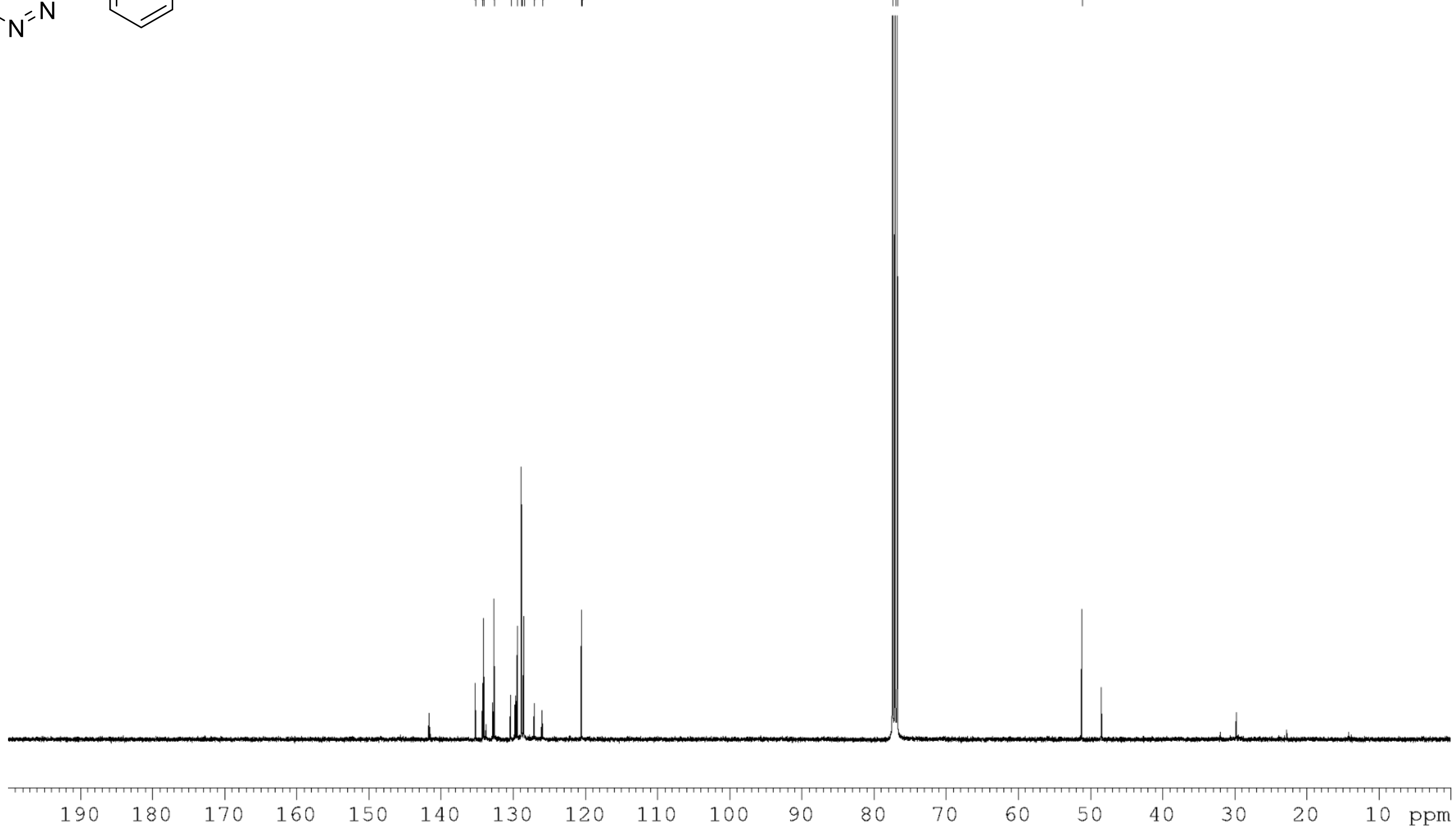

135.239
134.208
134.079
132.631
130.326
129.401
128.854
128.785
128.490
127.084
125.951
120.544
120.490
77.346
77.028
76.710
51.156
190
180
170
160
150
140
130
120
110
100
90
80
70
60
50
40
30
20
10
ppm

^{1}H NMR of 1o

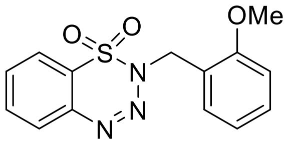

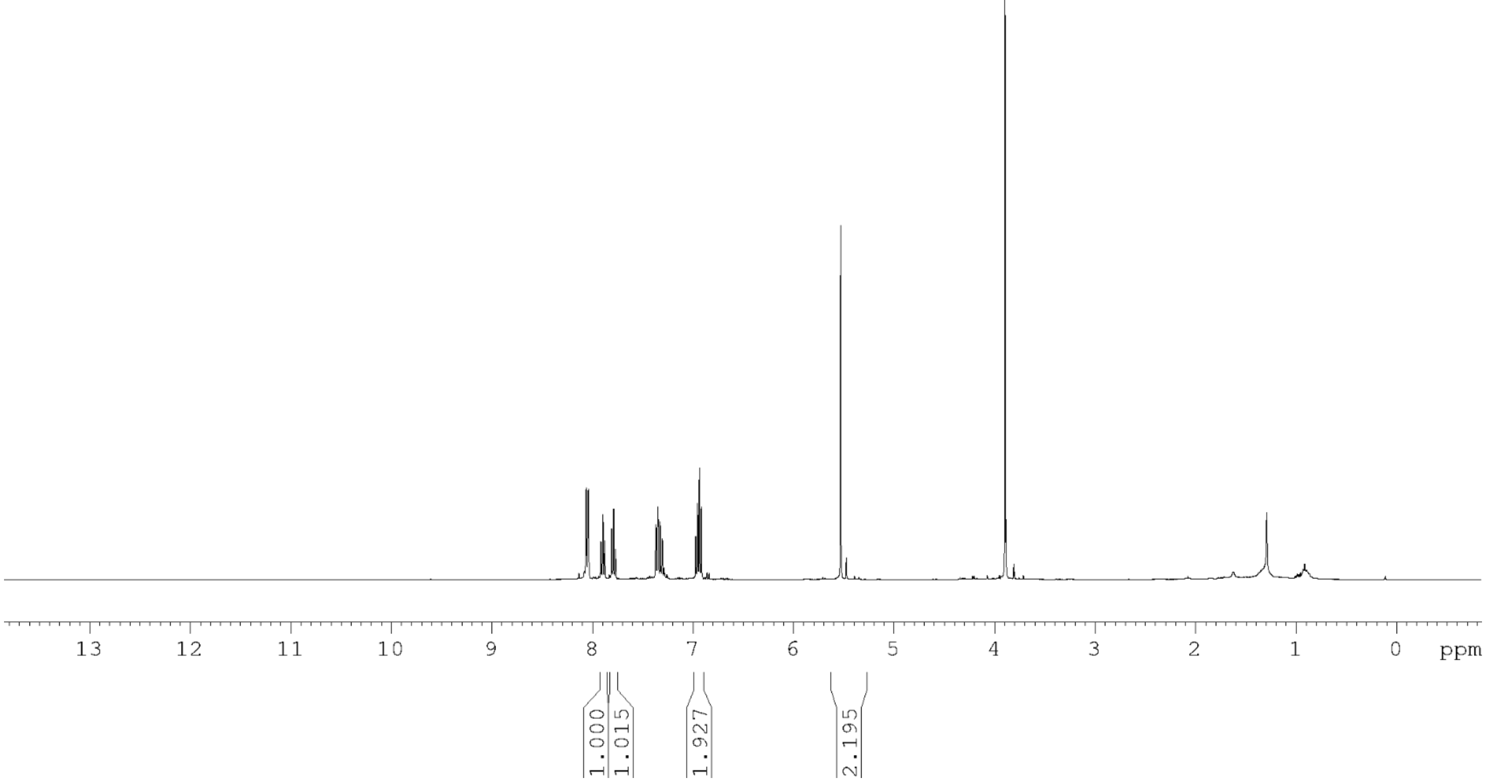

^{13}C NMR of 1o

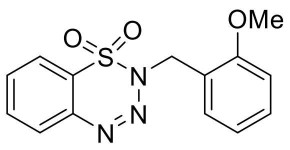
OMe
O
S
O
N
N
N

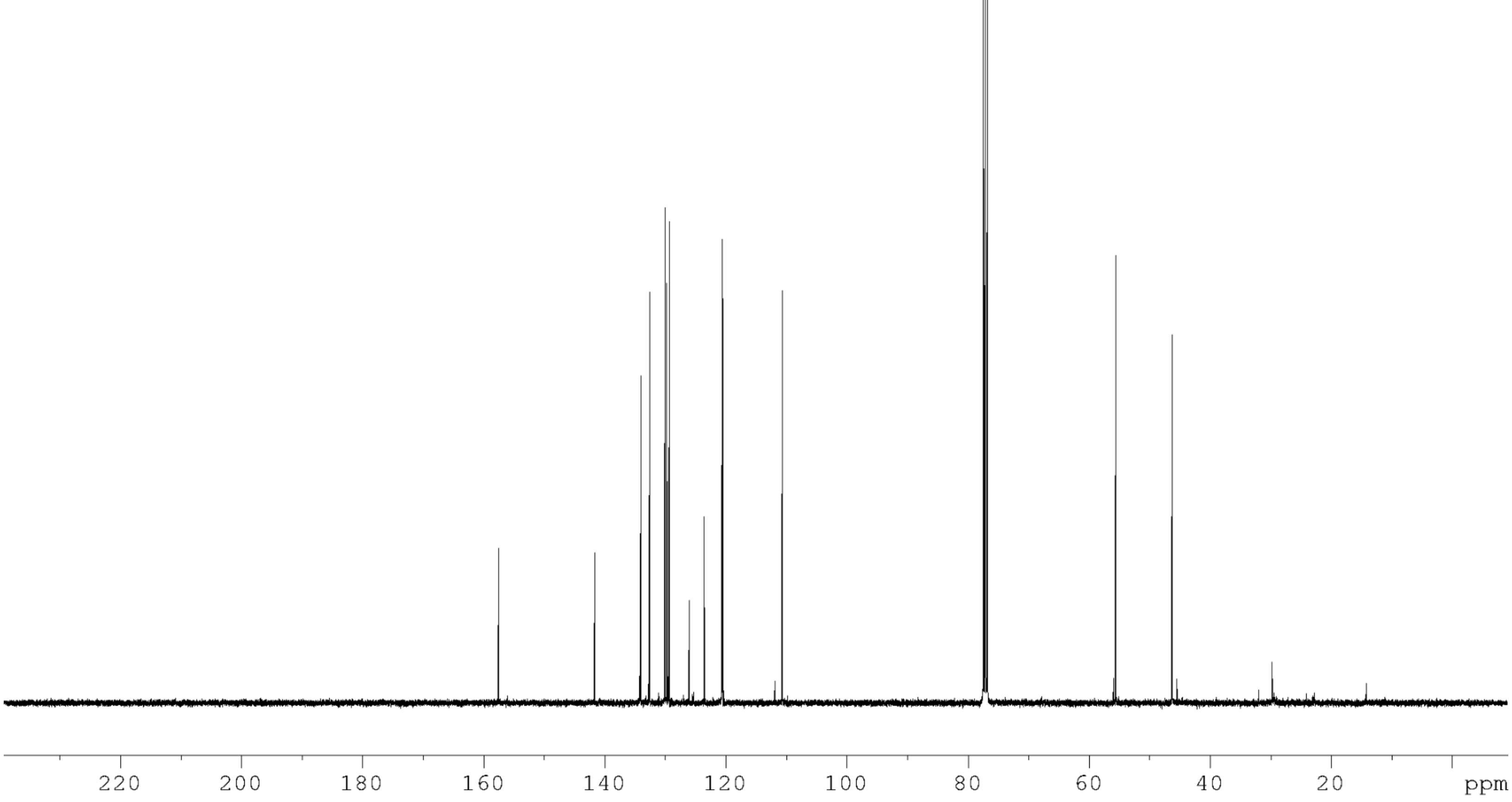
157.449
141.639
133.983
132.509
130.007
129.696
129.256
125.997
123.508
120.536
120.437
111.853
110.603
77.376
77.058
76.740
55.538
46.195
220
200
180
160
140
120
100
80
60
40
20
ppm

^{1}H NMR of 1p

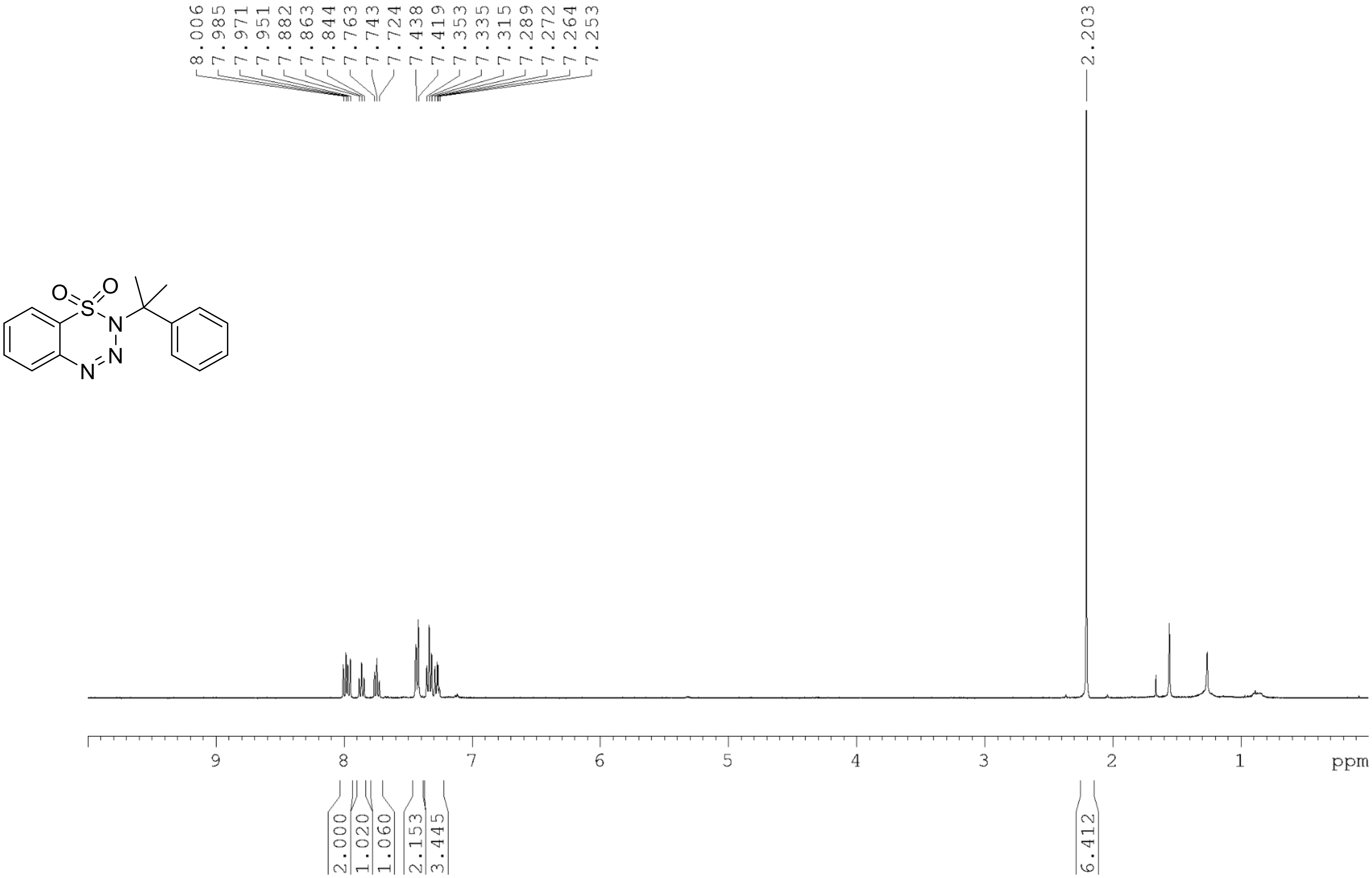
8.006
7.985
7.971
7.951
7.882
7.863
7.844
7.763
7.743
7.724
7.438
7.419
7.353
7.335
7.315
7.289
7.272
7.264
7.253
2.203
9
8
7
6
5
4
3
2
1
ppm
2.000
1.020
1.060
2.153
3.445
6.412

^{13}C NMR of 1p

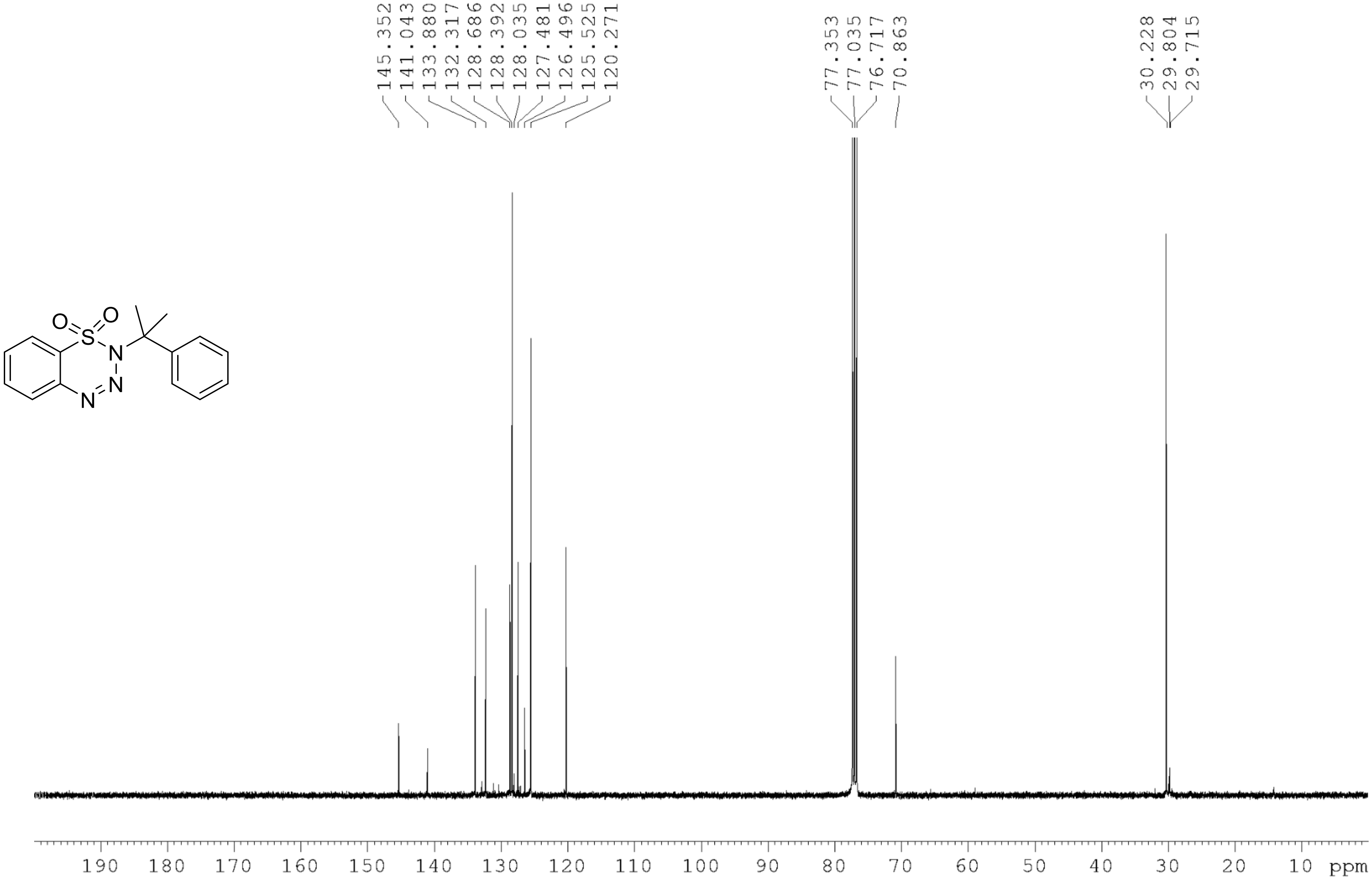
145.352
141.043
133.880
132.317
128.686
128.392
128.035
127.481
126.496
125.525
120.271
77.353
77.035
76.717
70.863
30.228
29.804
29.715
190 180 170 160 150 140 130 120 110 100 90 80 70 60 50 40 30 20 10 ppm

^{1}H NMR of 1q

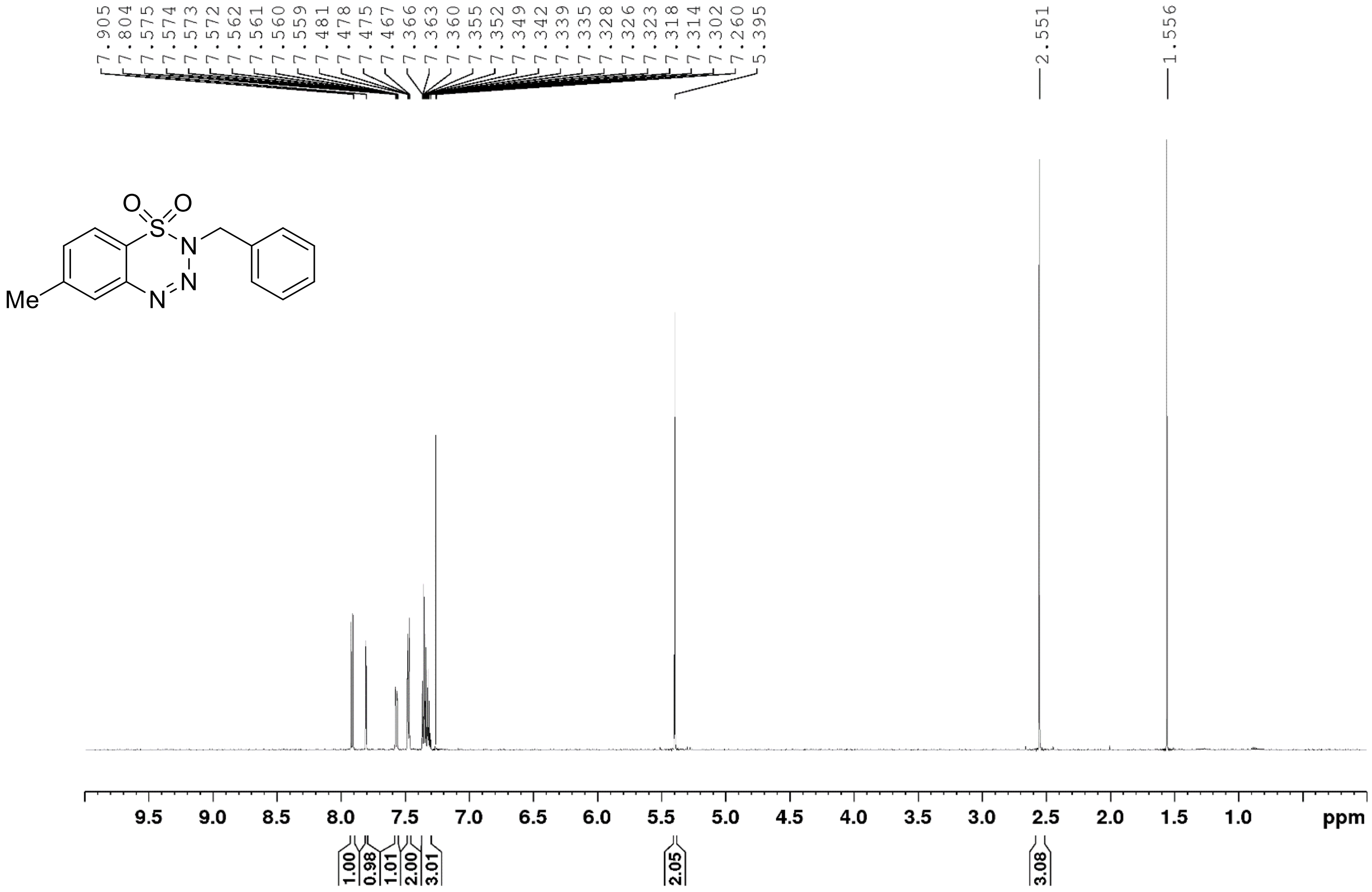

Me
7.905
7.804
7.575
7.574
7.573
7.572
7.562
7.561
7.560
7.559
7.481
7.478
7.475
7.467
7.366
7.363
7.360
7.355
7.352
7.349
7.342
7.339
7.335
7.328
7.326
7.323
7.318
7.314
7.302
7.260
5.395
2.551
1.556
9.5
9.0
8.5
8.0
7.5
7.0
6.5
6.0
5.5
5.0
4.5
4.0
3.5
3.0
2.5
2.0
1.5
1.0
ppm
1.00
0.98
1.01
2.00
3.01
2.05
3.08

^{13}C NMR of 1q

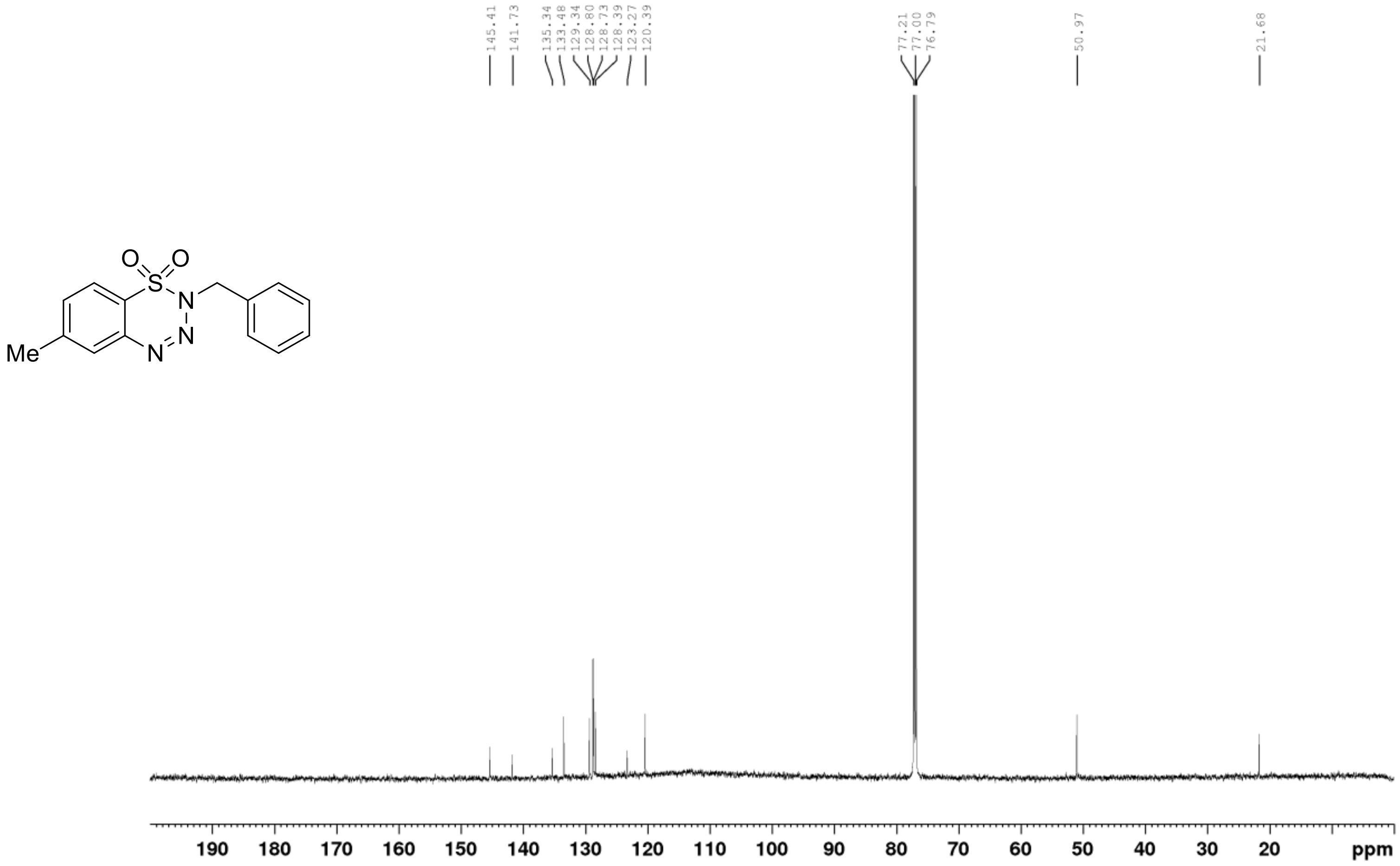
O
O
S
N
N
N
Me
145.41
141.73
135.34
133.48
129.34
128.80
128.73
128.39
123.27
120.39
77.21
77.00
76.79
50.97
21.68
190 180 170 160 150 140 130 120 110 100 90 80 70 60 50 40 30 20 ppm

^{1}H NMR of 1r

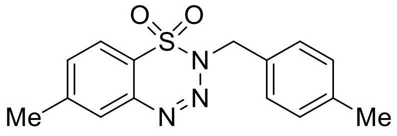
O
O
S
N
N
N
Me
Me

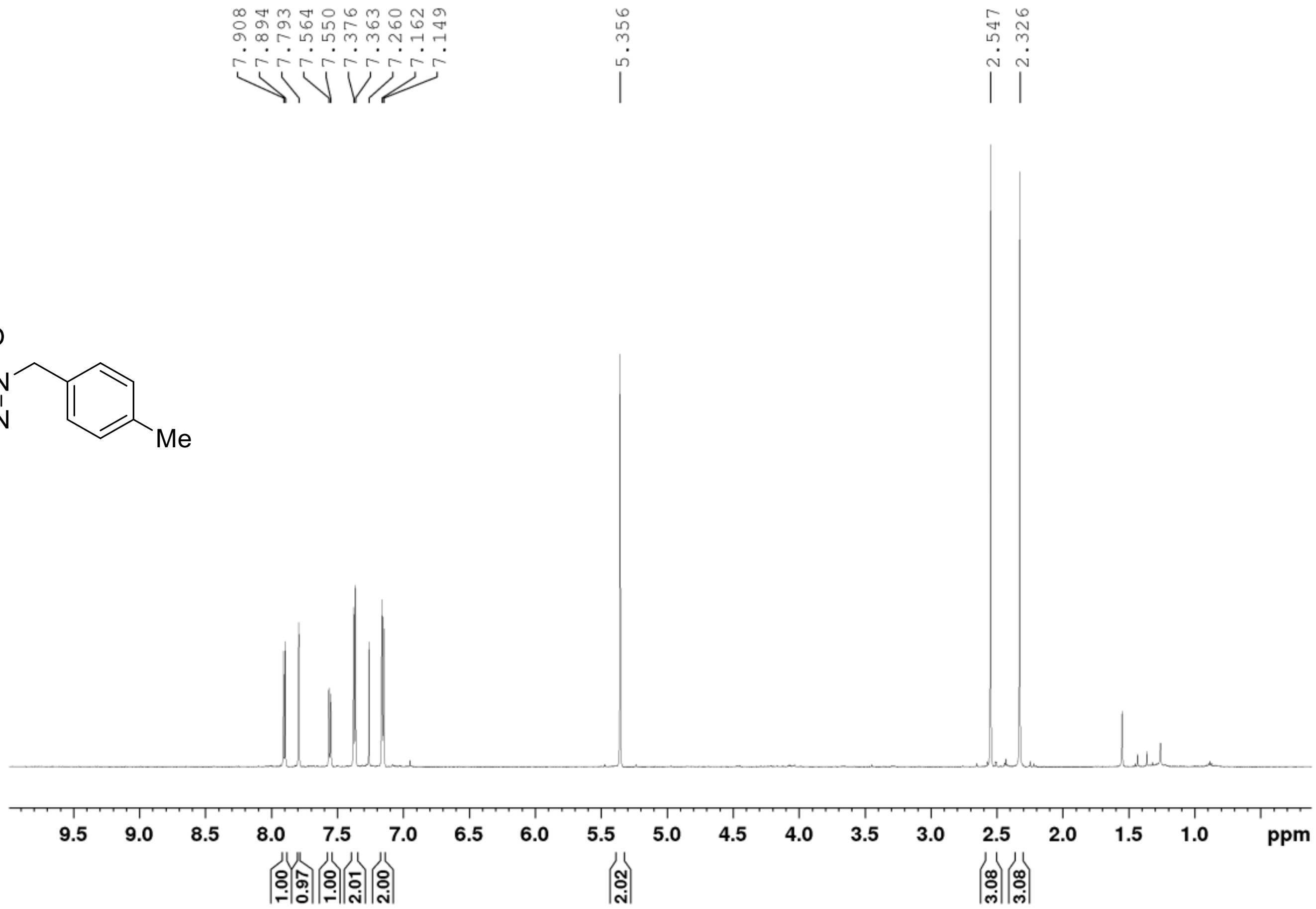
7.908
7.894
7.793
7.564
7.550
7.376
7.363
7.260
7.162
7.149
5.356
2.547
2.326
9.5
9.0
8.5
8.0
7.5
7.0
6.5
6.0
5.5
5.0
4.5
4.0
3.5
3.0
2.5
2.0
1.5
1.0
ppm
1.00
0.97
1.00
2.01
2.00
2.02
3.08
3.08

^{13}C NMR of 1r

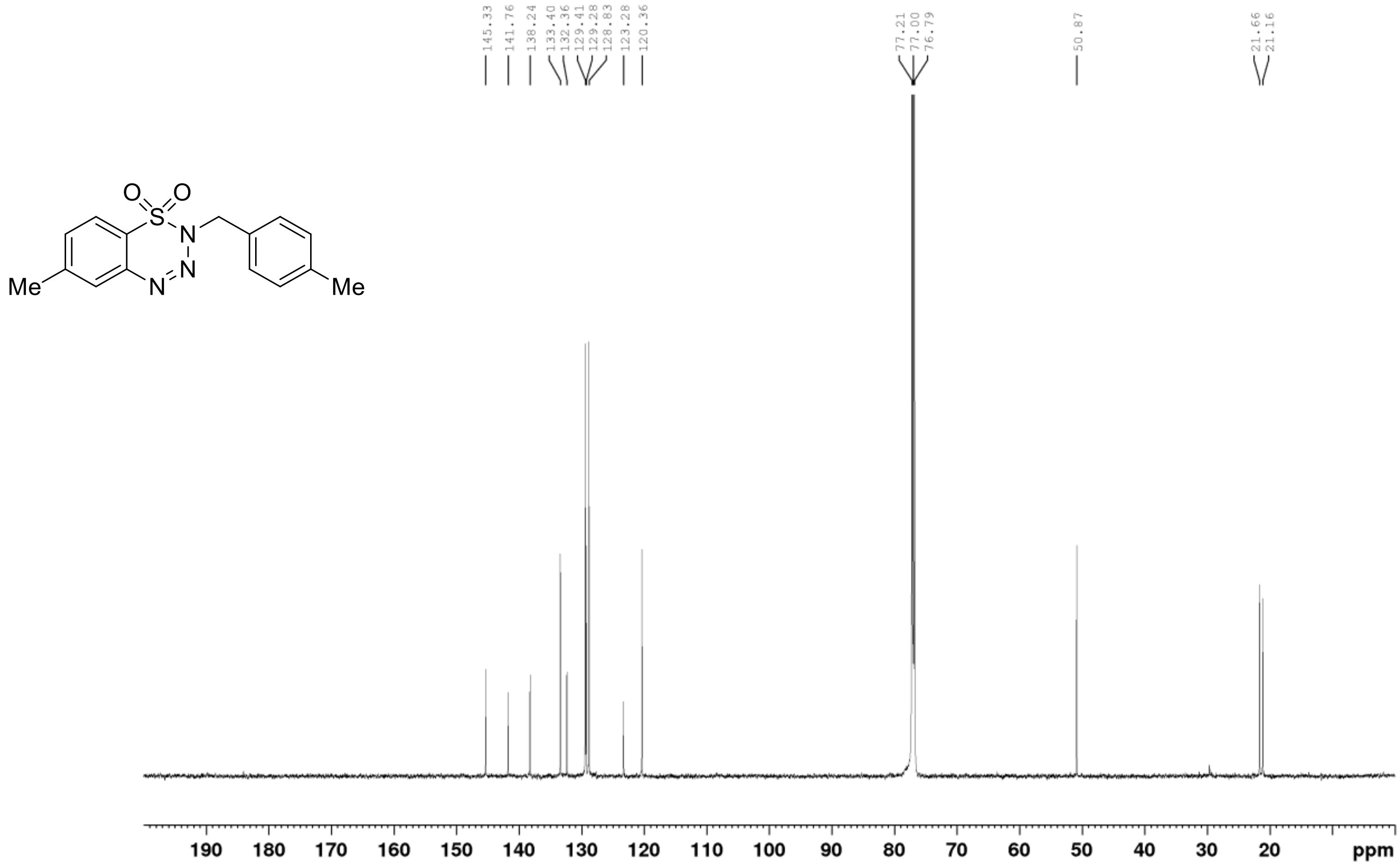

145.33
141.76
138.24
133.40
132.36
129.41
129.28
128.83
123.28
120.36
77.21
77.00
76.79
50.87
21.66
21.16
190
180
170
160
150
140
130
120
110
100
90
80
70
60
50
40
30
20
ppm
Me
Me

^{1}H NMR of 1s

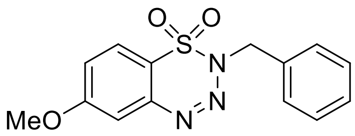

MeO

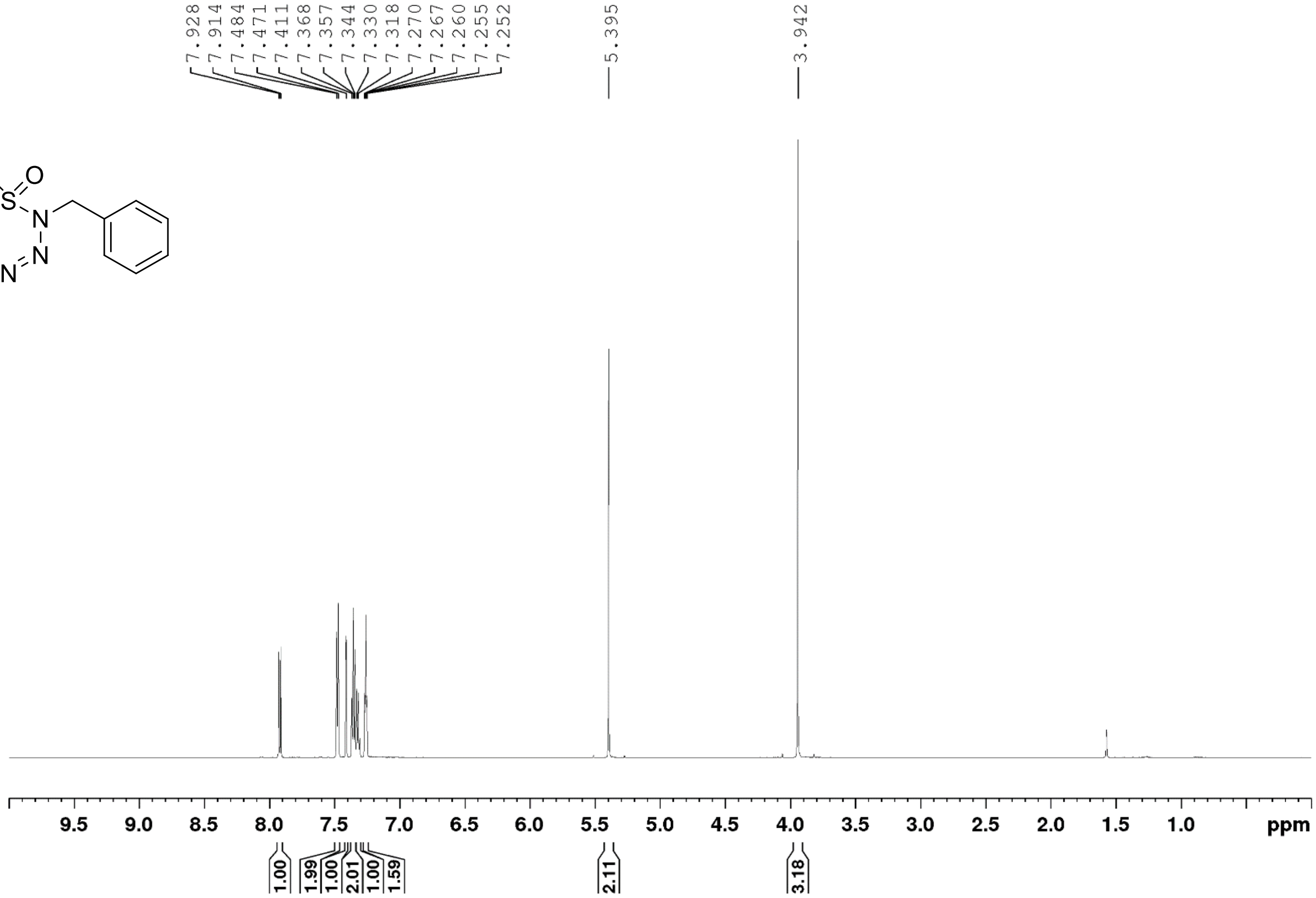

7.928
7.914
7.484
7.471
7.411
7.368
7.357
7.344
7.330
7.318
7.270
7.267
7.260
7.255
7.252
5.395
3.942
9.5
9.0
8.5
8.0
7.5
7.0
6.5
6.0
5.5
5.0
4.5
4.0
3.5
3.0
2.5
2.0
1.5
1.0
ppm
1.00
1.99
1.00
2.01
1.00
1.59
2.11
3.18

^{13}C NMR of 1s

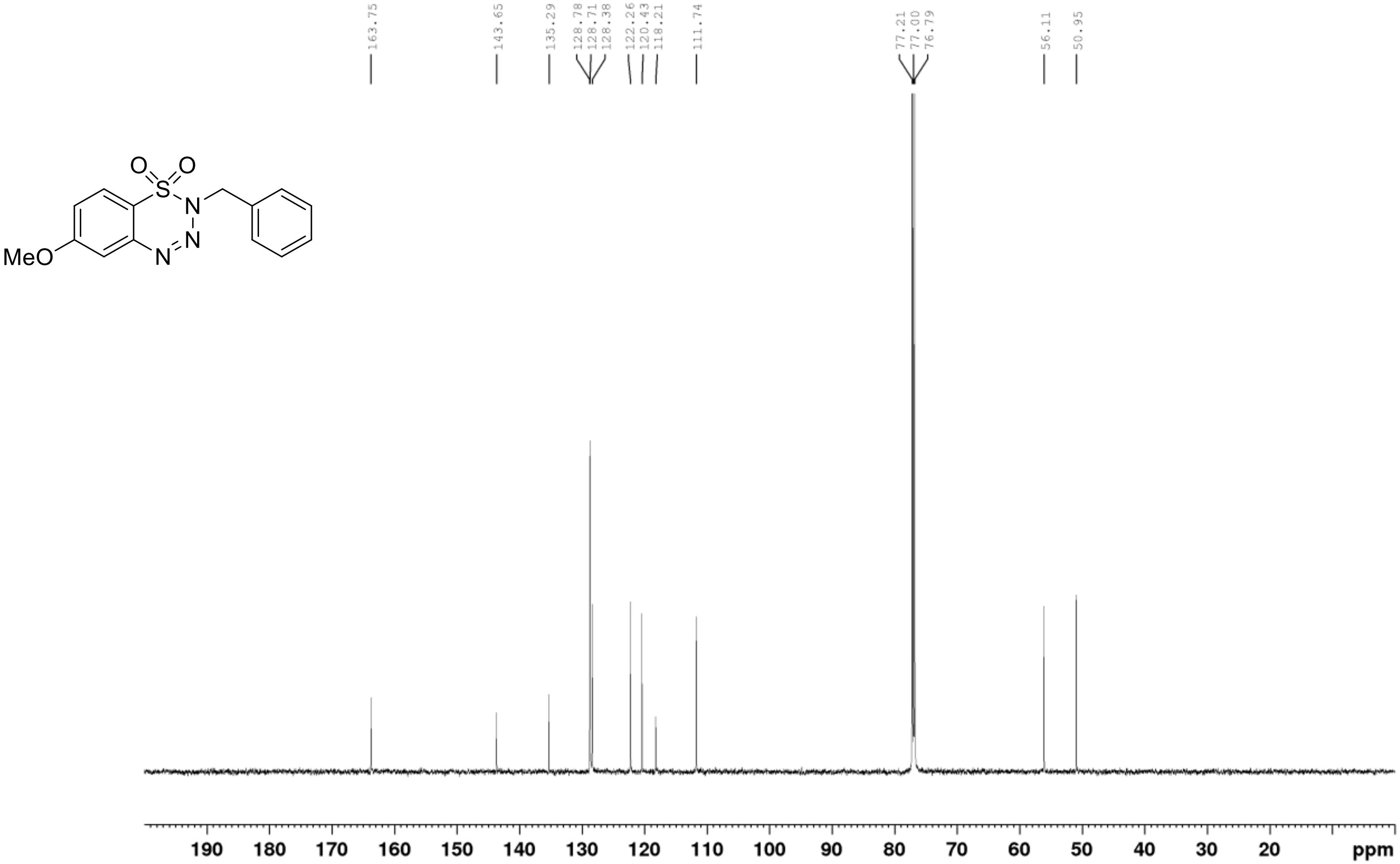
MeO
O
O
S
N
N
N
163.75
143.65
135.29
128.78
128.71
128.38
122.26
120.43
118.21
111.74
77.21
77.00
76.79
56.11
50.95
190 180 170 160 150 140 130 120 110 100 90 80 70 60 50 40 30 20 ppm

^{1}H NMR of 1t

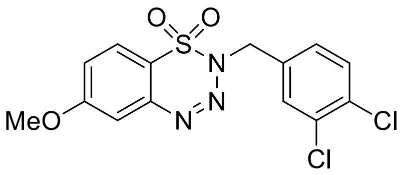
MeO
O
O
S
N
N
N
Cl
Cl

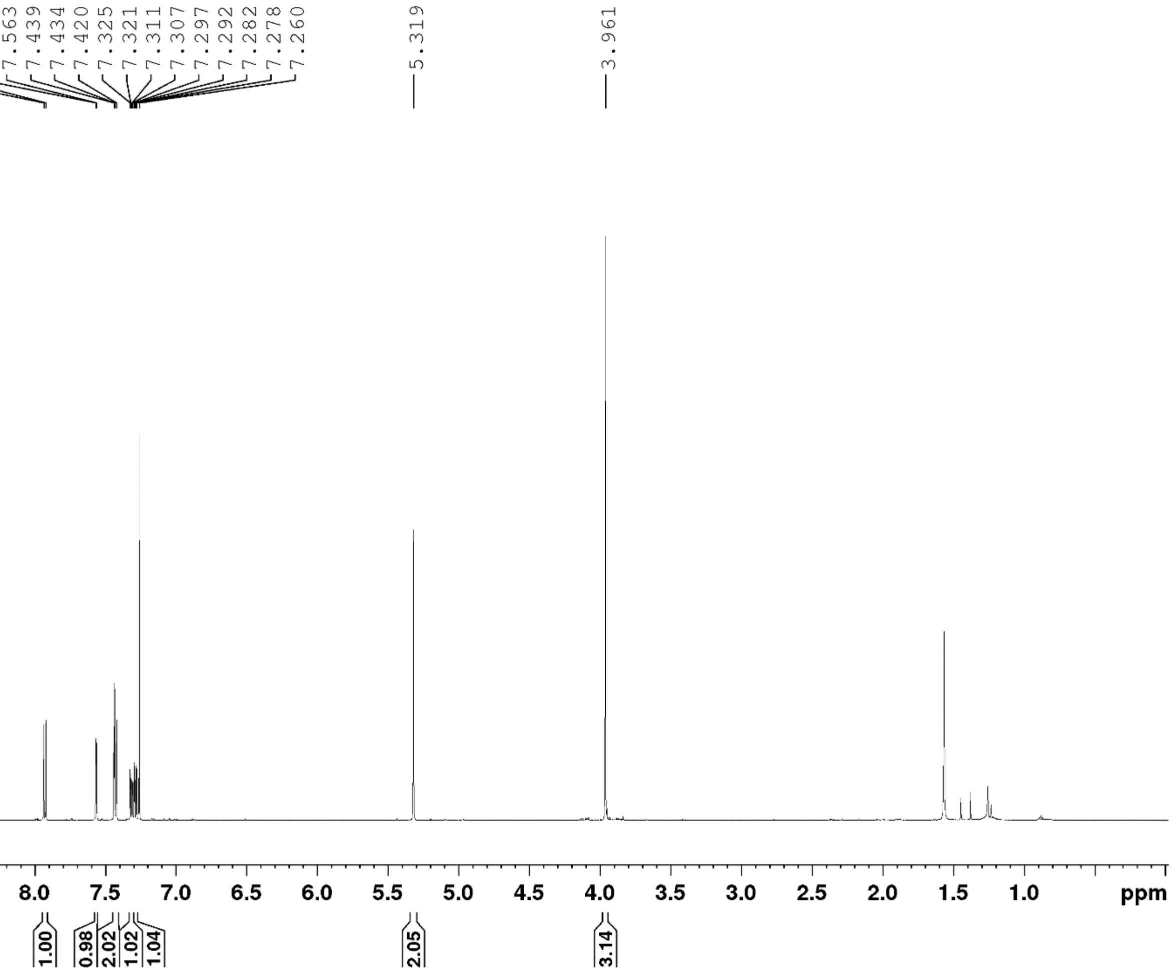
7.936
7.921
7.566
7.563
7.439
7.434
7.420
7.325
7.321
7.311
7.307
7.297
7.292
7.282
7.278
7.260
5.319
3.961
9.5 9.0 8.5 8.0 7.5 7.0 6.5 6.0 5.5 5.0 4.5 4.0 3.5 3.0 2.5 2.0 1.5 1.0 ppm
1.00
0.98
2.02
1.02
1.04
2.05
3.14

^{13}C NMR of 1t

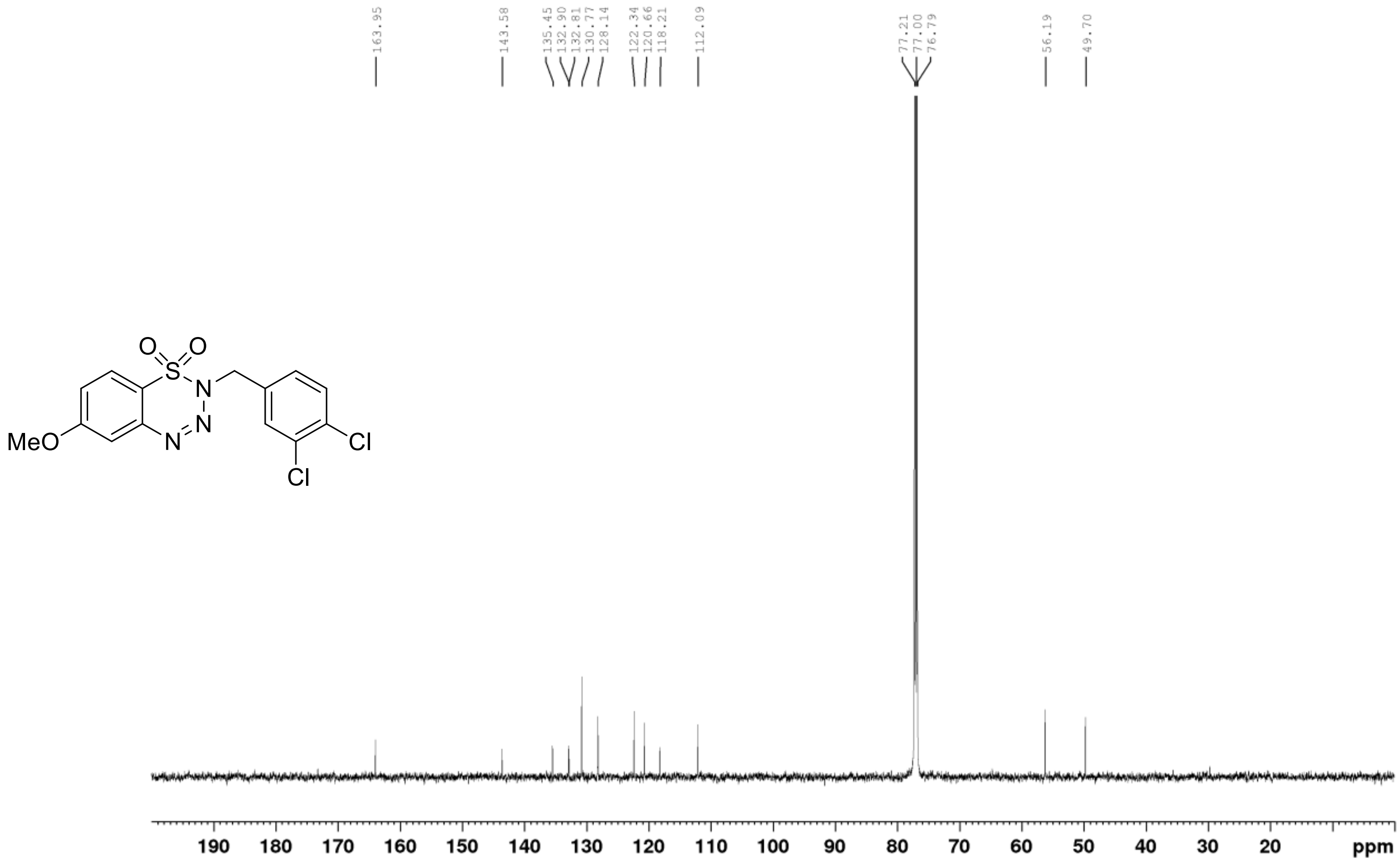
163.95
143.58
135.45
132.90
132.81
130.77
128.14
122.34
120.66
118.21
112.09
77.21
77.00
76.79
56.19
49.70
190 180 170 160 150 140 130 120 110 100 90 80 70 60 50 40 30 20 ppm

^{1}H NMR of 1u

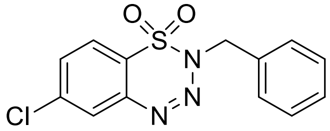

O
O
S
N
N
N
Cl

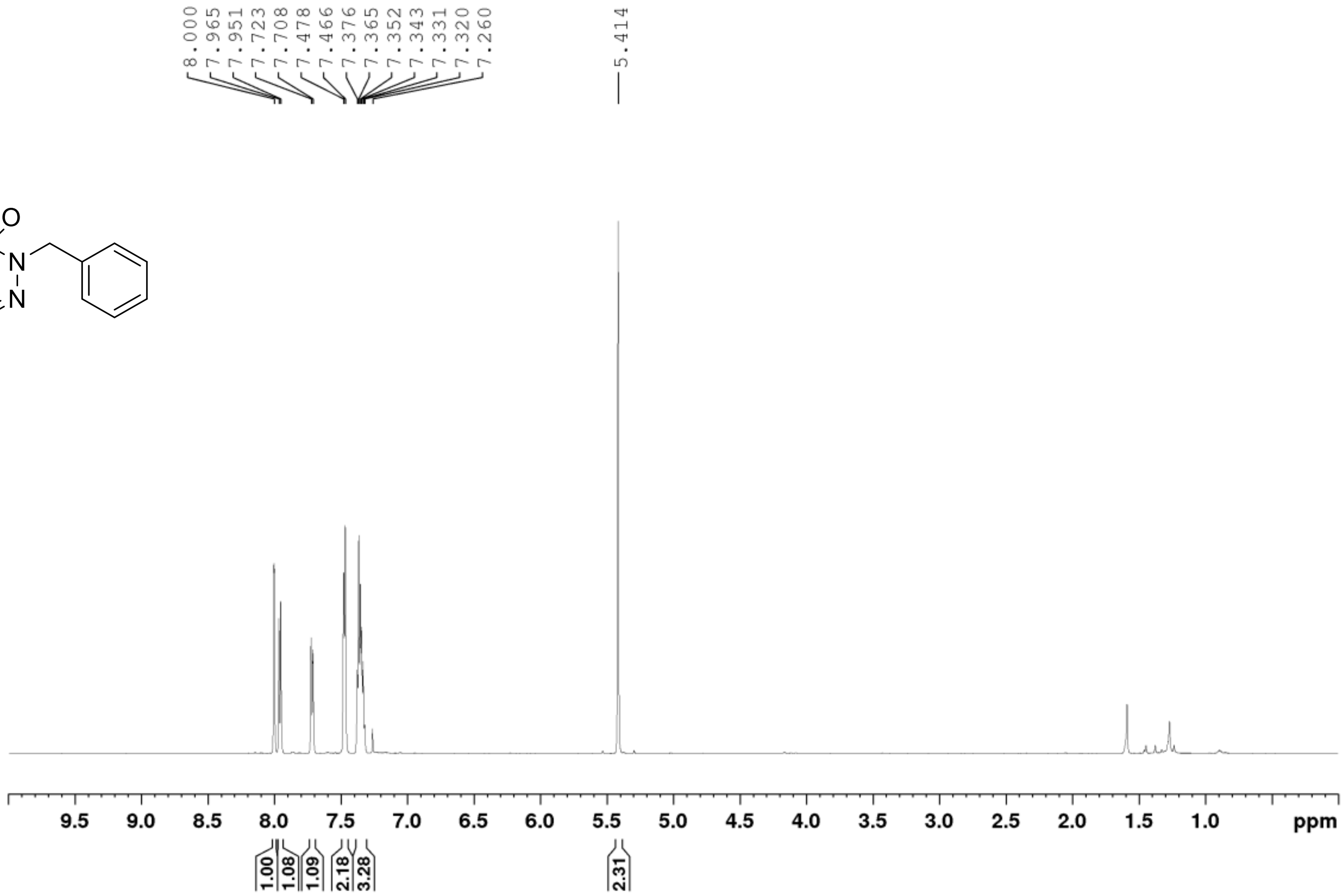

8.000
7.965
7.951
7.723
7.708
7.478
7.466
7.376
7.365
7.352
7.343
7.331
7.320
7.260
5.414
9.5
9.0
8.5
8.0
7.5
7.0
6.5
6.0
5.5
5.0
4.5
4.0
3.5
3.0
2.5
2.0
1.5
1.0
ppm
1.00
1.08
1.09
2.18
3.28
2.31

^{13}C NMR of 1u

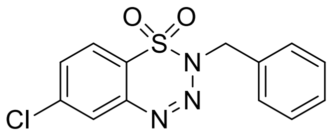
O O
S
N
N
N
N
Cl

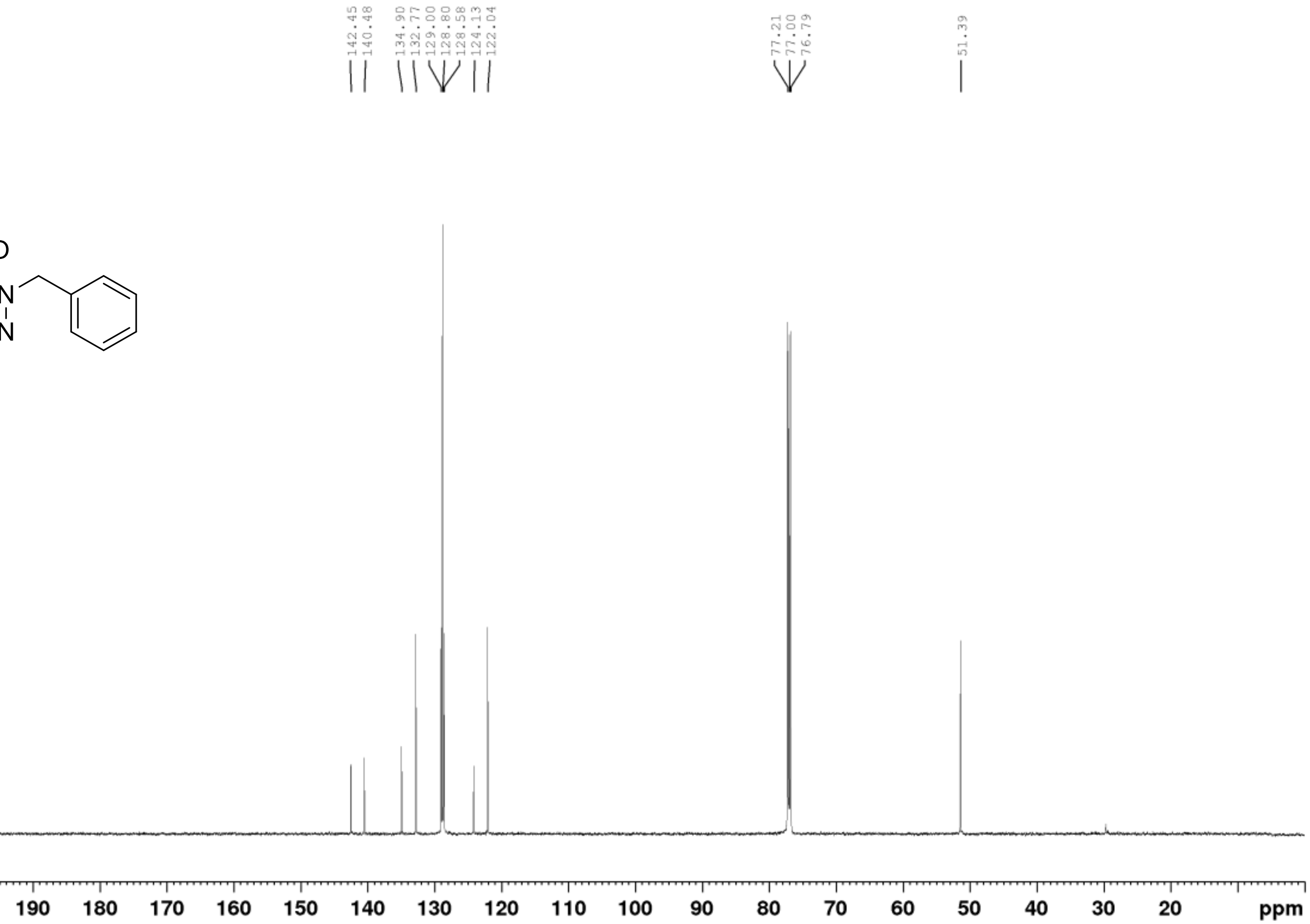
142.45
140.48
134.90
132.77
129.00
128.80
128.58
124.13
122.04
77.21
77.00
76.79
51.39
190 180 170 160 150 140 130 120 110 100 90 80 70 60 50 40 30 20 ppm

^{1}H NMR of 1v

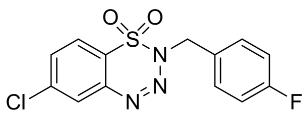
Cl
N
N
N
S
O
O
F

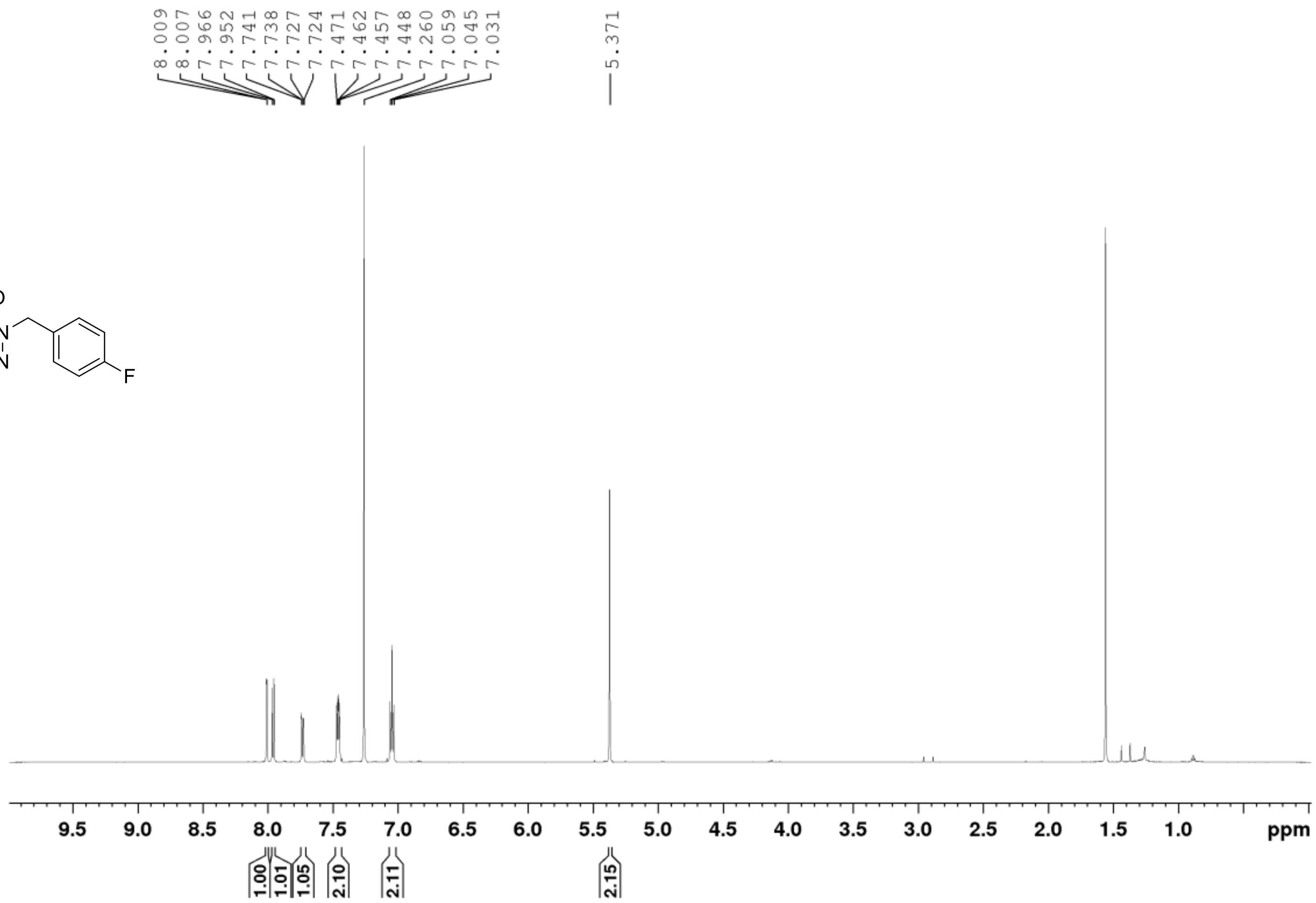
8.009
8.007
7.966
7.952
7.741
7.738
7.727
7.724
7.471
7.462
7.457
7.448
7.260
7.059
7.045
7.031
5.371
9.5
9.0
8.5
8.0
7.5
7.0
6.5
6.0
5.5
5.0
4.5
4.0
3.5
3.0
2.5
2.0
1.5
1.0
ppm
1.00
1.01
1.05
2.10
2.11
2.15

^{13}C NMR of 1v

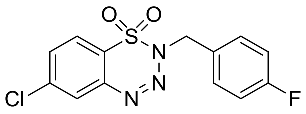
O
O
S
N
N
N
Cl
F

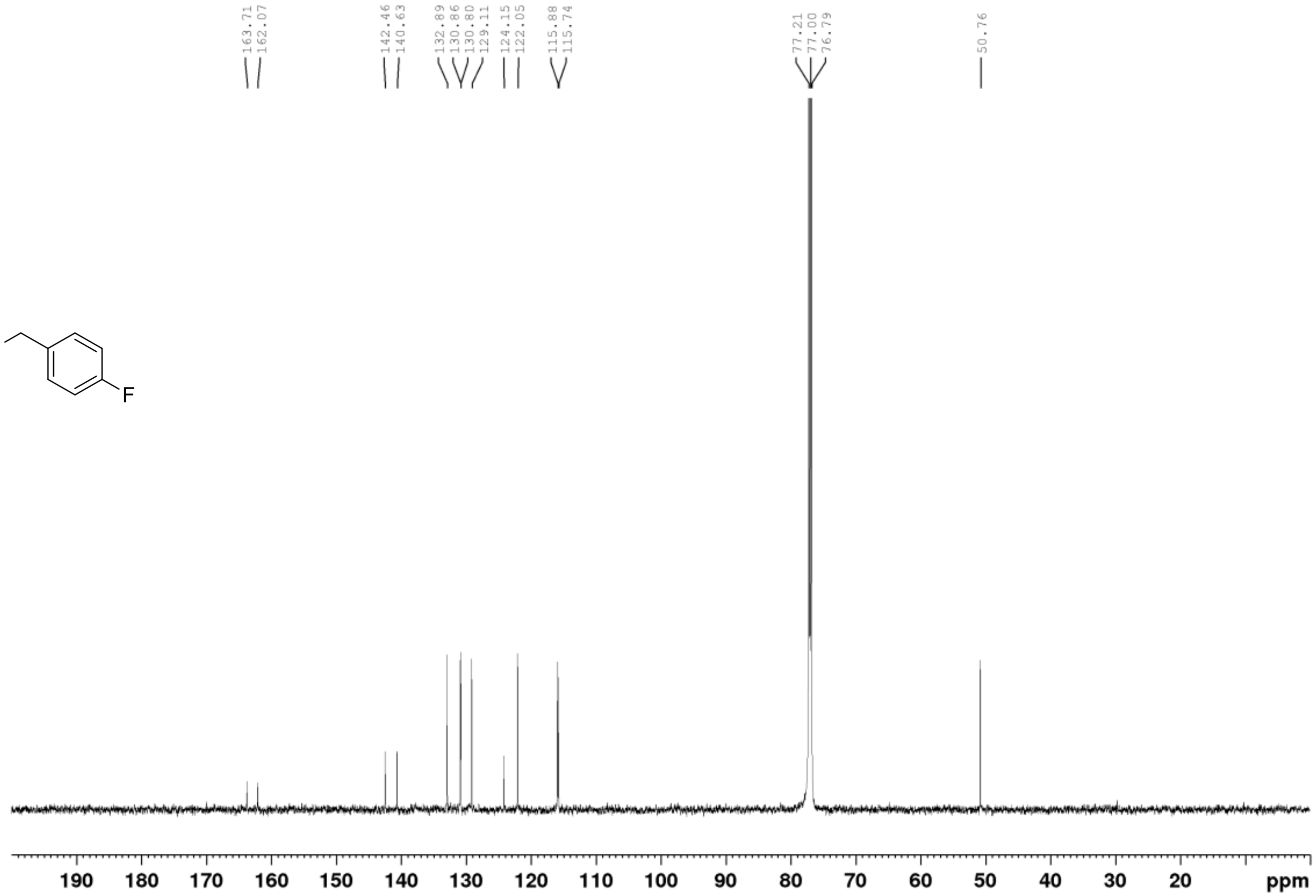
163.71
162.07
142.46
140.63
132.89
130.86
130.80
129.11
124.15
122.05
115.88
115.74
77.21
77.00
76.79
50.76
190 180 170 160 150 140 130 120 110 100 90 80 70 60 50 40 30 20 ppm

^{1}H NMR of 1w

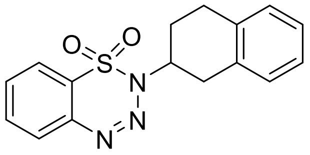

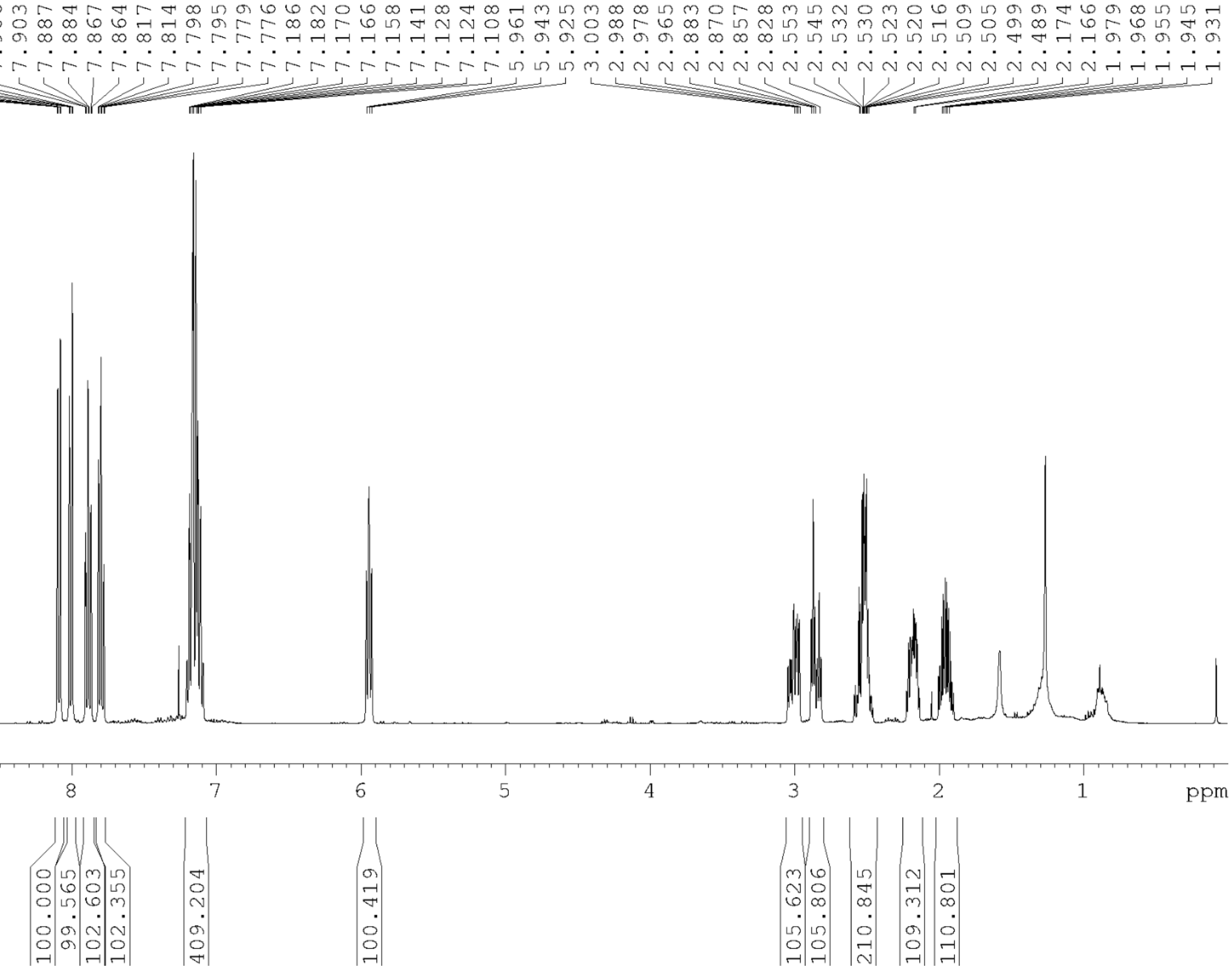
8.100
8.097
8.081
8.078
8.018
8.015
7.997
7.995
7.906
7.903
7.887
7.884
7.867
7.864
7.817
7.814
7.798
7.795
7.779
7.776
7.186
7.182
7.170
7.166
7.158
7.141
7.128
7.124
7.108
5.961
5.943
5.925
3.003
2.988
2.978
2.965
2.883
2.870
2.857
2.828
2.553
2.545
2.532
2.530
2.523
2.520
2.516
2.509
2.505
2.499
2.489
2.174
2.166
1.979
1.968
1.955
1.945
1.931
9
8
7
6
5
4
3
2
1
ppm
100.000
99.565
102.603
102.355
409.204
100.419
105.623
105.806
210.845
109.312
110.801

^{13}C NMR of 1w

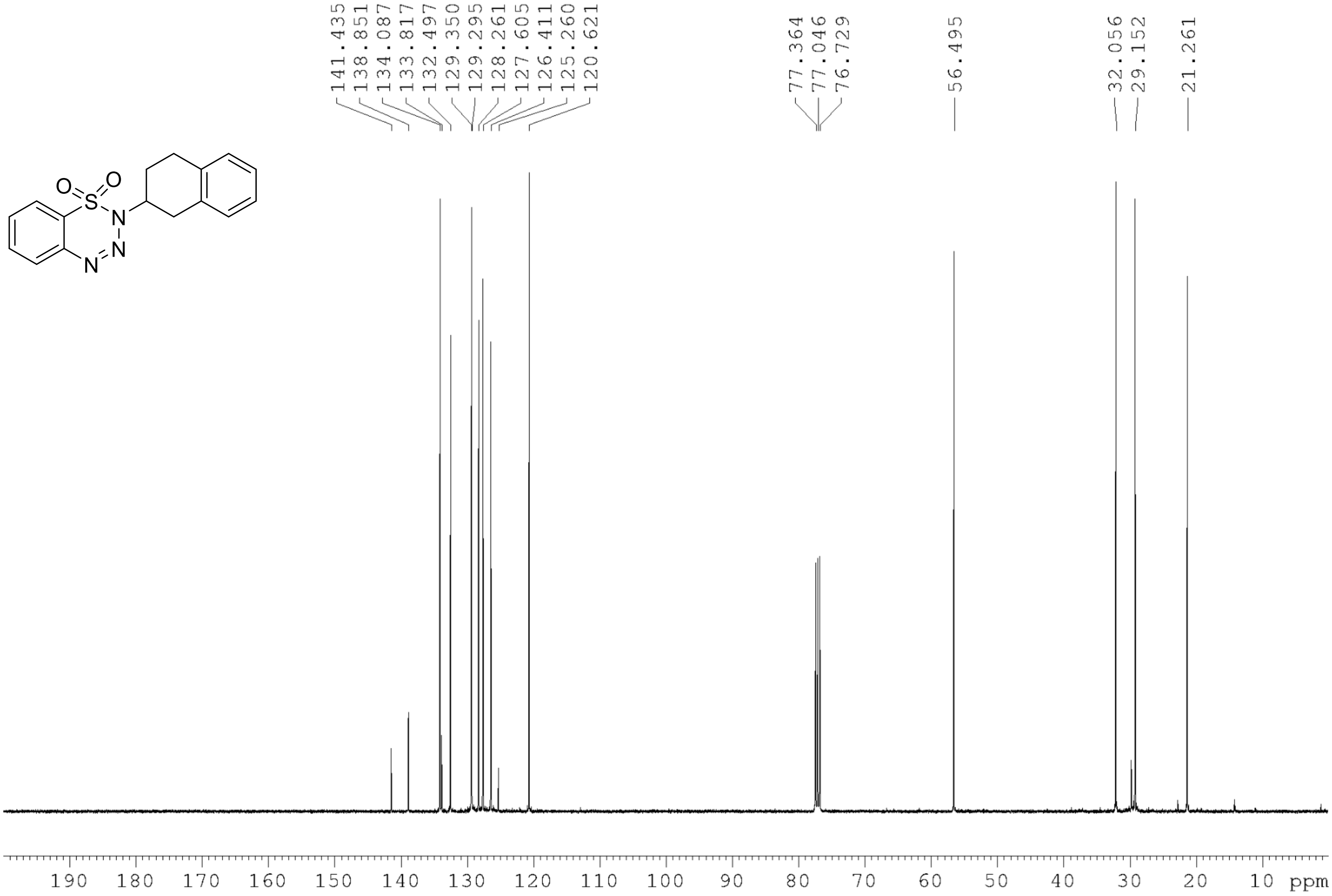

O
O
S
N
N
N
141.435
138.851
134.087
133.817
132.497
129.350
129.295
128.261
127.605
126.411
125.260
120.621
77.364
77.046
76.729
56.495
32.056
29.152
21.261
190 180 170 160 150 140 130 120 110 100 90 80 70 60 50 40 30 20 10 ppm

^{1}H NMR of 1x

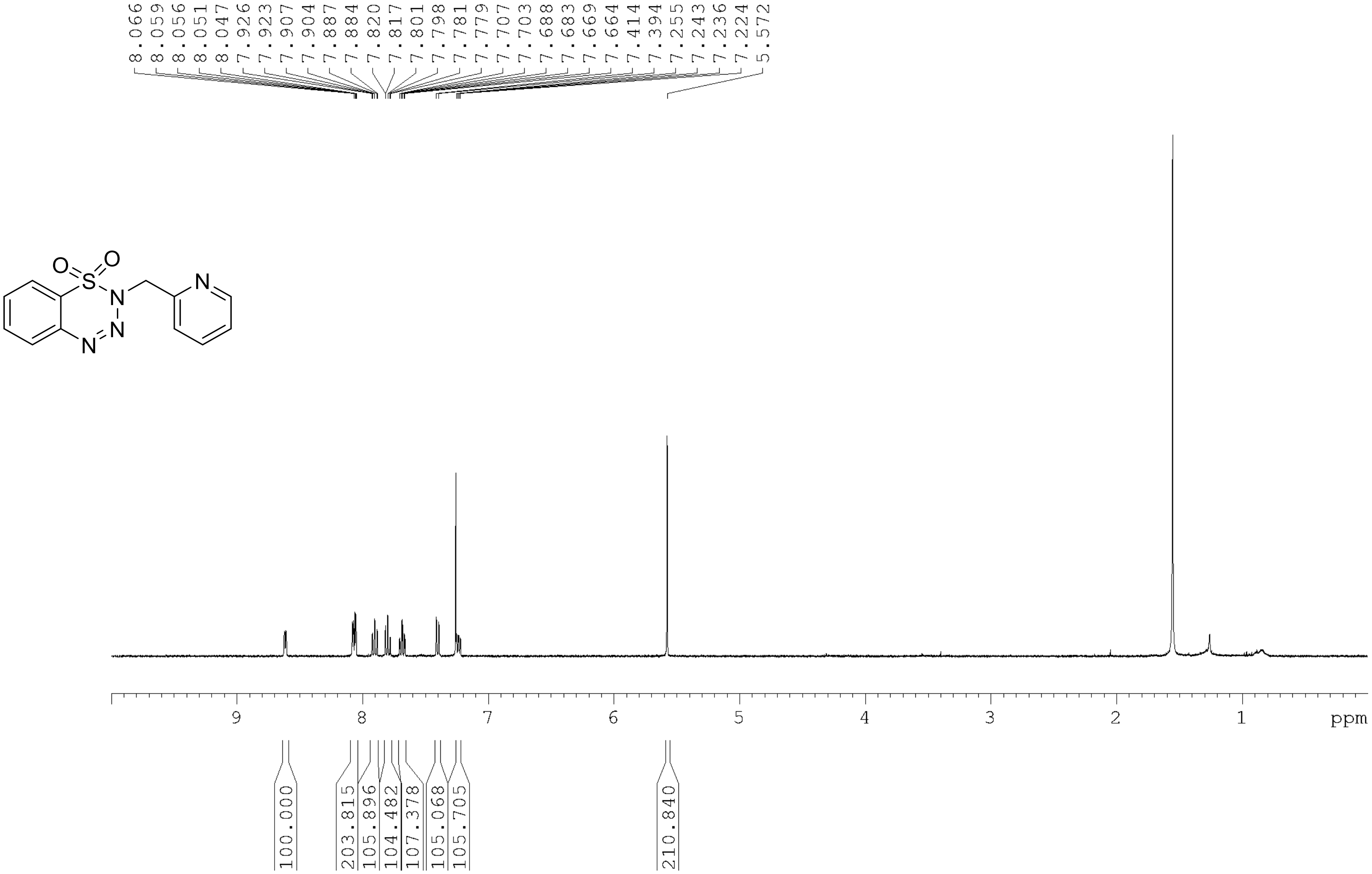
8.066
8.059
8.056
8.051
8.047
7.926
7.923
7.907
7.904
7.887
7.884
7.820
7.817
7.801
7.798
7.781
7.779
7.707
7.703
7.688
7.683
7.669
7.664
7.414
7.394
7.255
7.243
7.236
7.224
5.572
9
8
7
6
5
4
3
2
1
ppm
100.000
203.815
105.896
104.482
107.378
105.068
105.705
210.840

^{13}C NMR of 1x

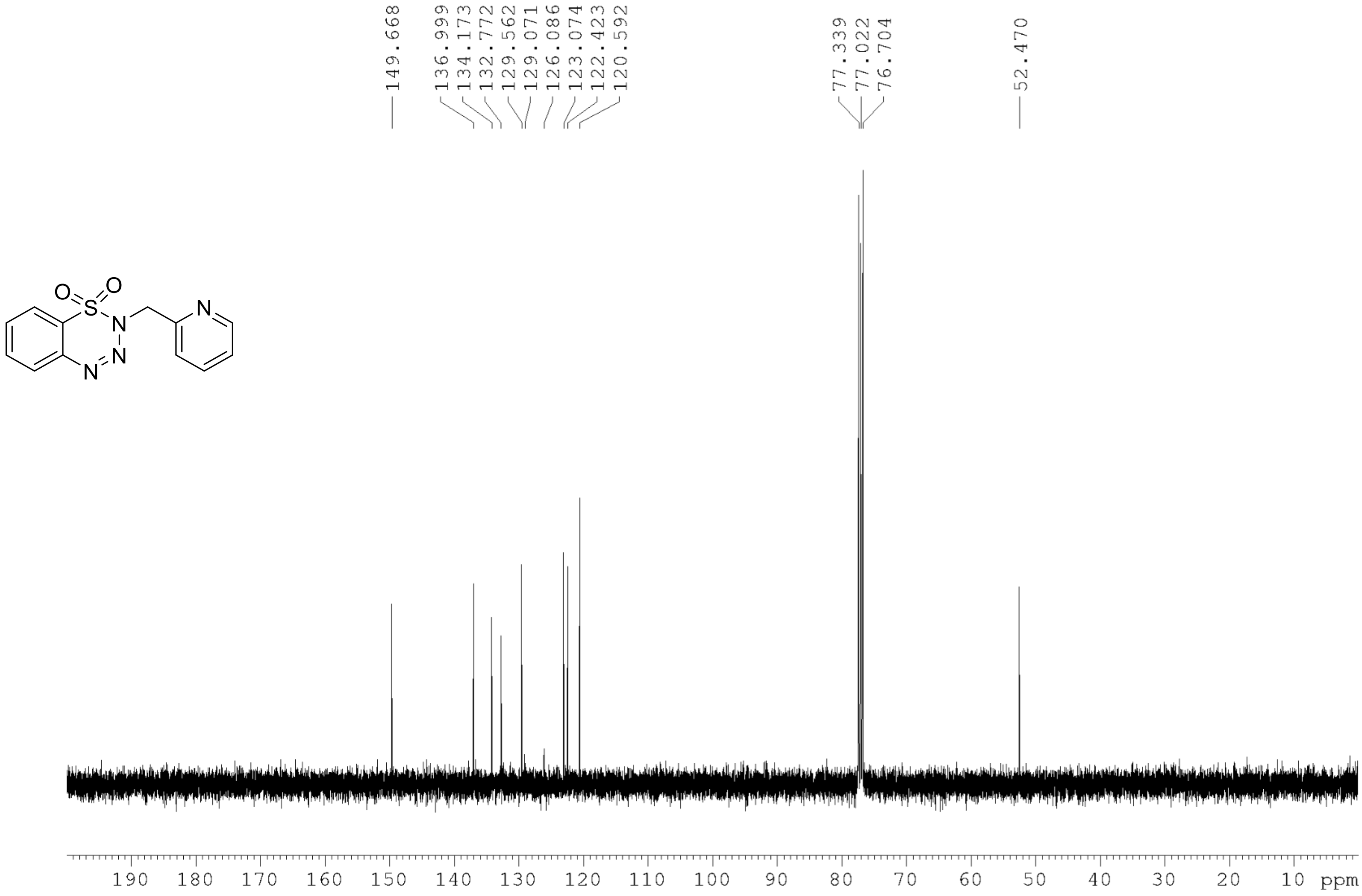

O
O
S
N
N
N
N
149.668
136.999
134.173
132.772
129.562
129.071
126.086
123.074
122.423
120.592
77.339
77.022
76.704
52.470
190 180 170 160 150 140 130 120 110 100 90 80 70 60 50 40 30 20 10 ppm

^{1}H NMR of 2a

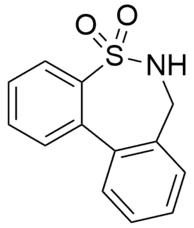

^{13}C NMR of 2a

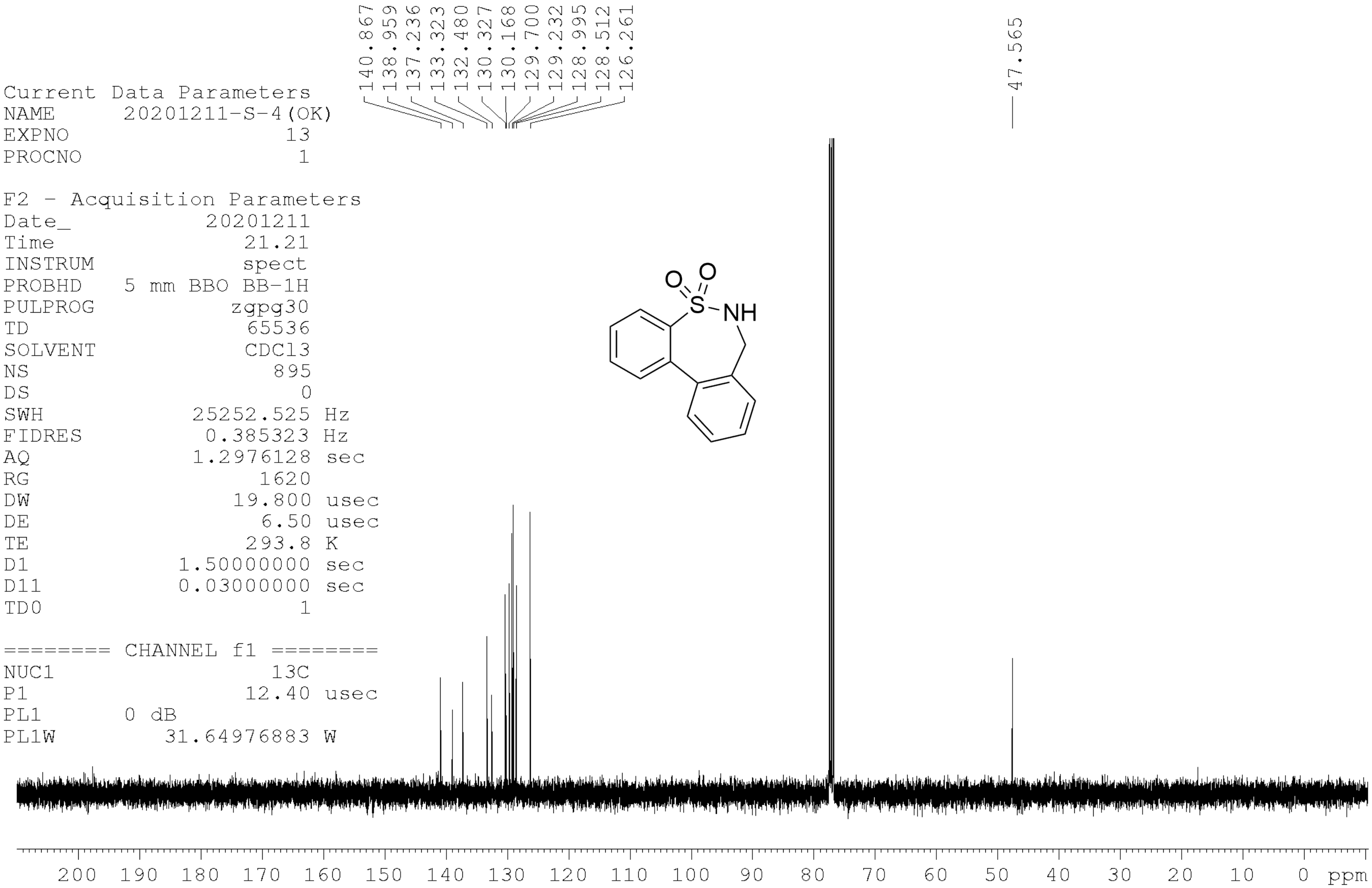

140.867
138.959
137.236
133.323
132.480
130.327
130.168
129.700
129.232
128.995
128.512
126.261
47.565
Current Data Parameters
NAME 20201211-S-4(OK)
EXPNO 13
PROCNO 1
F2 - Acquisition Parameters
Date_ 20201211
Time 21.21
INSTRUM spect
PROBHD 5 mm BBO BB-1H
PULPROG zgpg30
TD 65536
SOLVENT CDCl3
NS 895
DS 0
SWH 25252.525 Hz
FIDRES 0.385323 Hz
AQ 1.2976128 sec
RG 1620
DW 19.800 usec
DE 6.50 usec
TE 293.8 K
D1 1.50000000 sec
D11 0.03000000 sec
TD0 1
======== CHANNEL f1 ========
NUC1 13C
P1 12.40 usec
PL1 0 dB
PL1W 31.64976883 W
O
O
S
NH
200
190
180
170
160
150
140
130
120
110
100
90
80
70
60
50
40
30
20
10
0
ppm

^{1}H NMR of 2b

```
Current Data Parameters
NAME      20210616-S-10
EXPNO                16
PROCNO                1

F2 - Acquisition Parameters
Date_          20210616
Time              17.11 h
INSTRUM  Avance NANOBAY
PROBHD   Z163739_0358 (
PULPROG            zg30
TD                32768
SOLVENT           CDCl3
NS                    8
DS                    0
SWH            5882.353 Hz
FIDRES         0.359030 Hz
AQ            2.7852800 sec
RG              97.1264
DW               85.000 usec
DE                 9.26 usec
TE                296.2 K
D1           1.50000000 sec
TD0                   1
SFO1        400.1526010 MHz
NUC1                 1H
P0                 2.67 usec
P1                 8.00 usec
PLW1        23.43799973 W

F2 - Processing parameters
SI                32768
SF          400.1500000 MHz
```

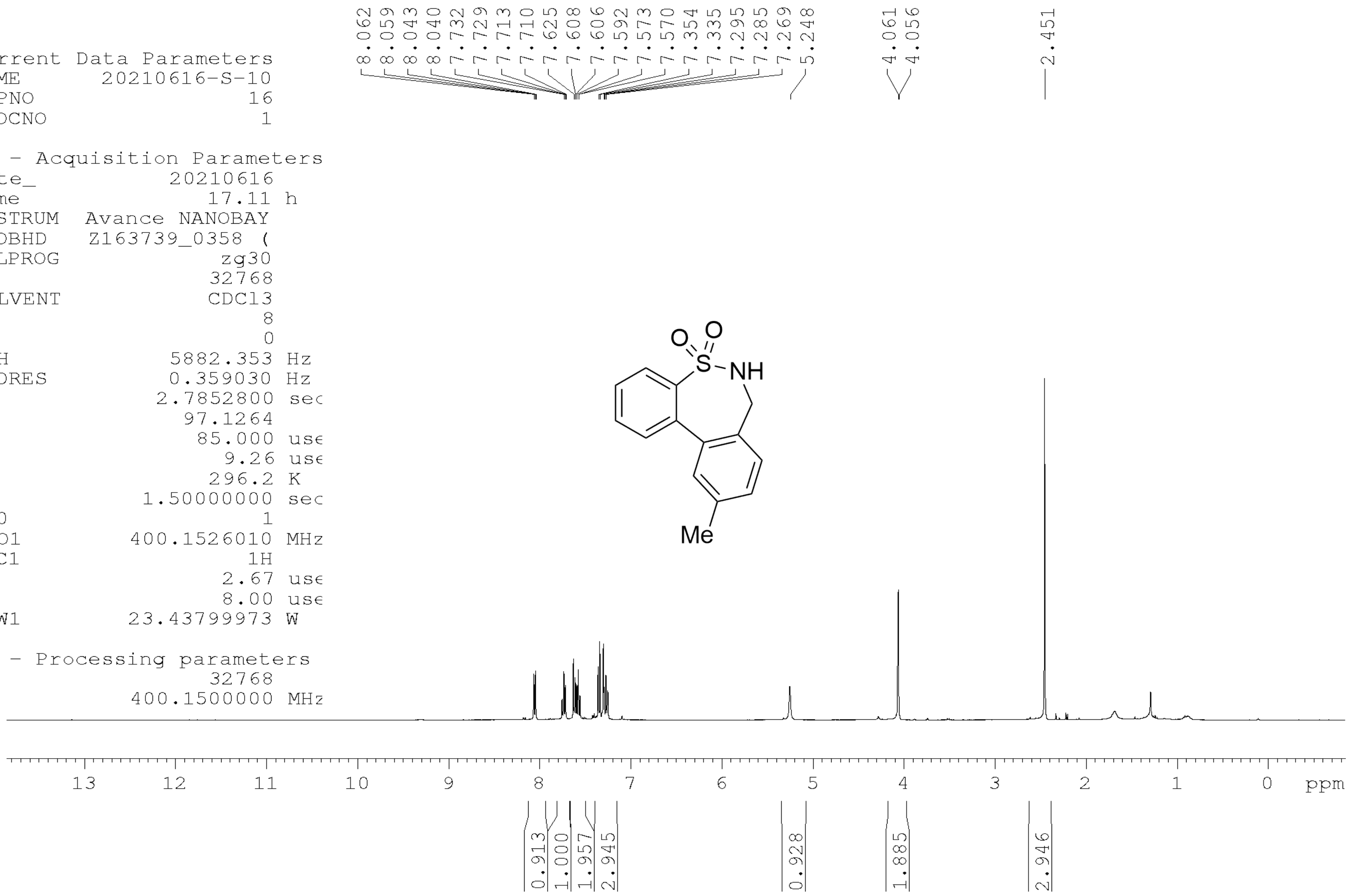

^{13}C NMR of 2b

Current Data Parameters
NAME 20210616-S-10
EXPNO 17
PROCNO 1

F2 - Acquisition Parameters
Date_ 20210616
Time 18.04 h
INSTRUM Avance NANOBAY
PROBHD Z163739_0358 (
PULPROG zgpg30
TD 65536
SOLVENT CDCl3
NS 1000
DS 0
SWH 25000.000 Hz
FIDRES 0.762939 Hz
AQ 1.3107200 sec
RG 101
DW 20.000 usec
DE 6.50 usec
TE 297.7 K
D1 1.50000000 sec
D11 0.03000000 sec
TD0 1
SFO1 100.6293690 MHz
NUC1 13C
P0 2.67 usec
P1 8.00 usec
PLW1 97.02799988 W
SFO2 400.1518007 MHz
NUC2 1H
CPDPRG[2 waltz65
PCPD2 90.00 usec
PLW2 23.43799973 W
PLW12 0.18519001 W
PLW13 0.09314800 W

[illegible]

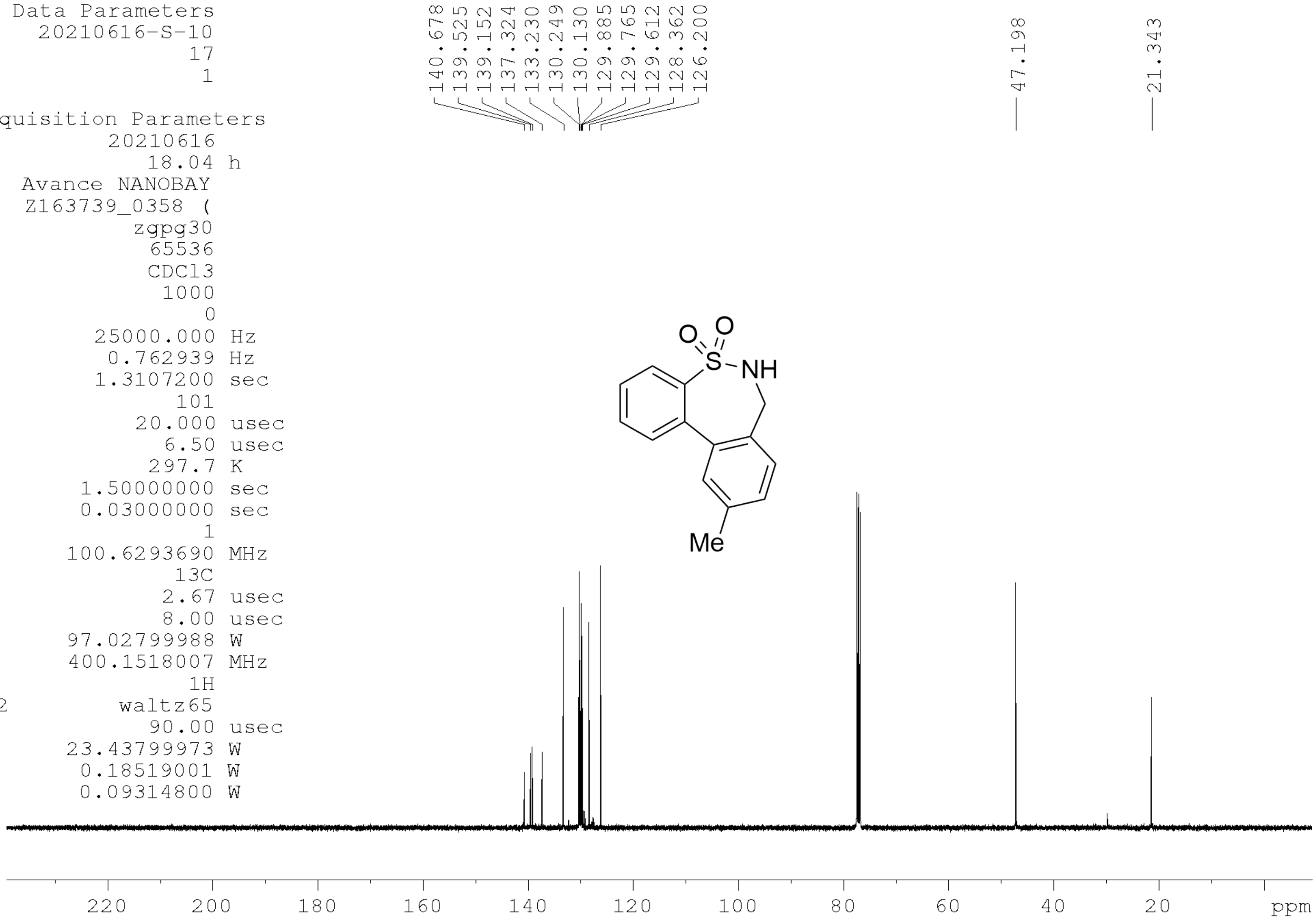

^{1}H NMR of 2c

Current Data Parameters
NAME 20211006-S20 (ter but)
EXPNO 18
PROCNO 1

F2 - Acquisition Parameters
Date_ 20211006
Time 22.09 h
INSTRUM Avance NANOBAY
PROBHD Z163739_0358 (
PULPROG zg30
TD 32768
SOLVENT CDCl3
NS 41
DS 0
SWH 5882.353 Hz
FIDRES 0.359030 Hz
AQ 2.7852800 sec
RG 48.3721
DW 85.000 usec
DE 9.26 usec
TE 297.2 K
D1 1.50000000 sec
TD0 1
SFO1 400.1526010 MHz
NUC1 1H
P0 2.67 usec
P1 8.00 usec
PLW1 23.43799973 W

F2 - Processing parameters
SI 32768
SF 400.1500000 MHz
WDW EM

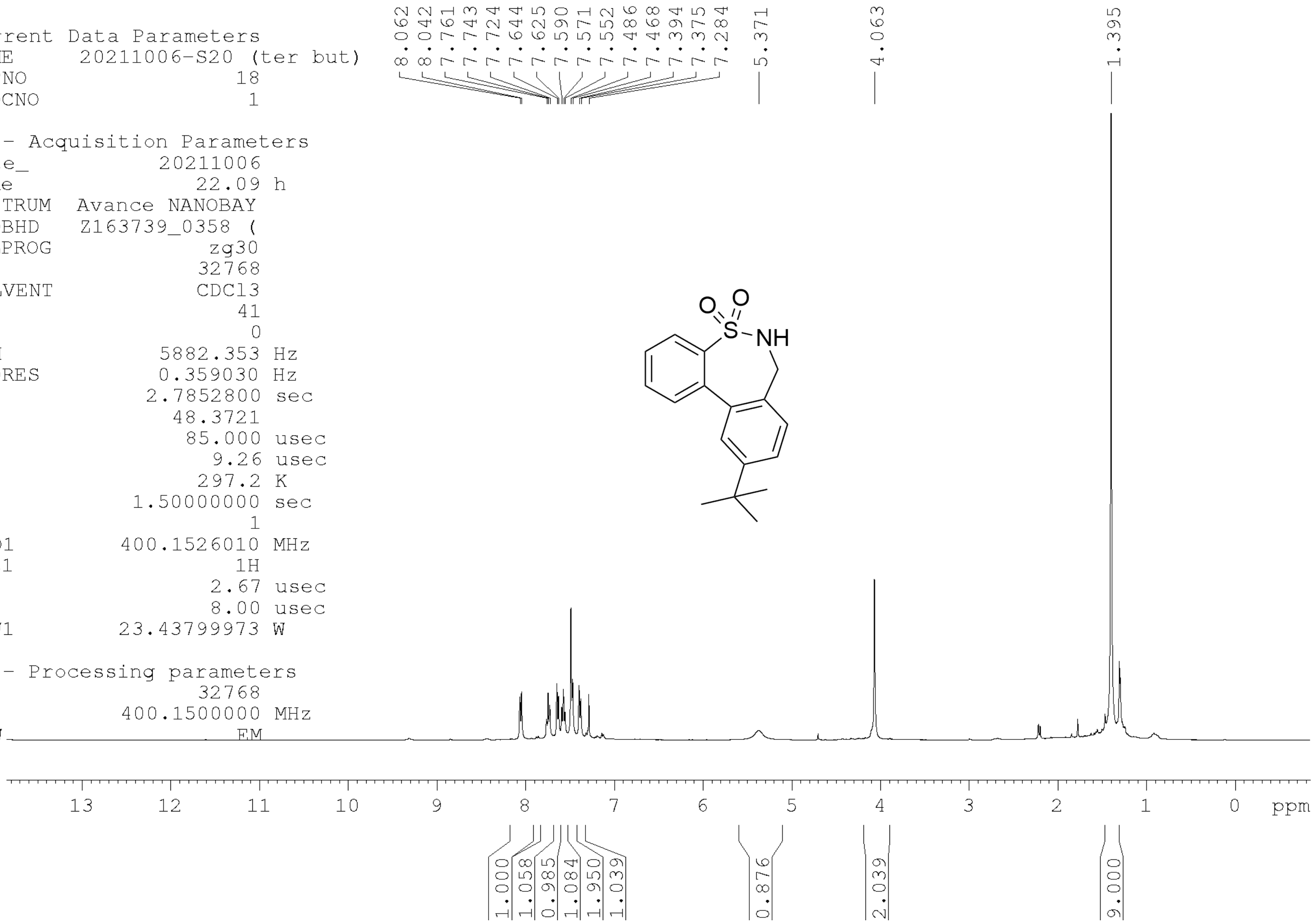

^{13}C NMR of 2c

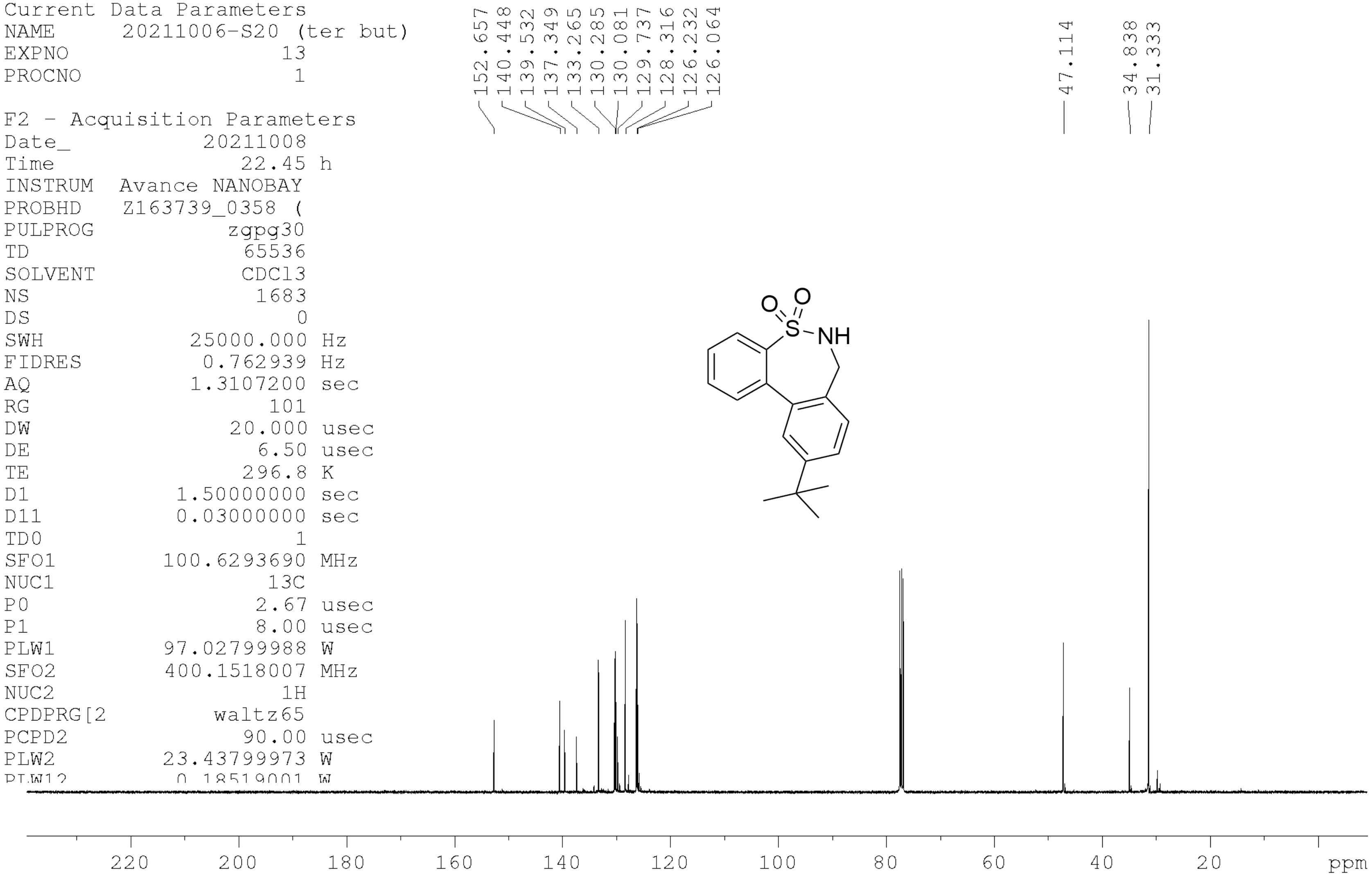

Current Data Parameters
NAME 20211006-S20 (ter but)
EXPNO 13
PROCNO 1
F2 - Acquisition Parameters
Date_ 20211008
Time 22.45 h
INSTRUM Avance NANOBAY
PROBHD Z163739_0358 (
PULPROG zgpg30
TD 65536
SOLVENT CDCl3
NS 1683
DS 0
SWH 25000.000 Hz
FIDRES 0.762939 Hz
AQ 1.3107200 sec
RG 101
DW 20.000 usec
DE 6.50 usec
TE 296.8 K
D1 1.50000000 sec
D11 0.03000000 sec
TD0 1
SFO1 100.6293690 MHz
NUC1 13C
P0 2.67 usec
P1 8.00 usec
PLW1 97.02799988 W
SFO2 400.1518007 MHz
NUC2 1H
CPDPRG[2 waltz65
PCPD2 90.00 usec
PLW2 23.43799973 W
PLW12 0.18519001 W
152.657
140.448
139.532
137.349
133.265
130.285
130.081
129.737
128.316
126.232
126.064
47.114
34.838
31.333
O
O
S
NH
220
200
180
160
140
120
100
80
60
40
20
ppm

^{1}H NMR of 2d

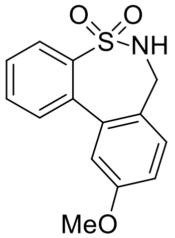

MeO
NH
O
S
O

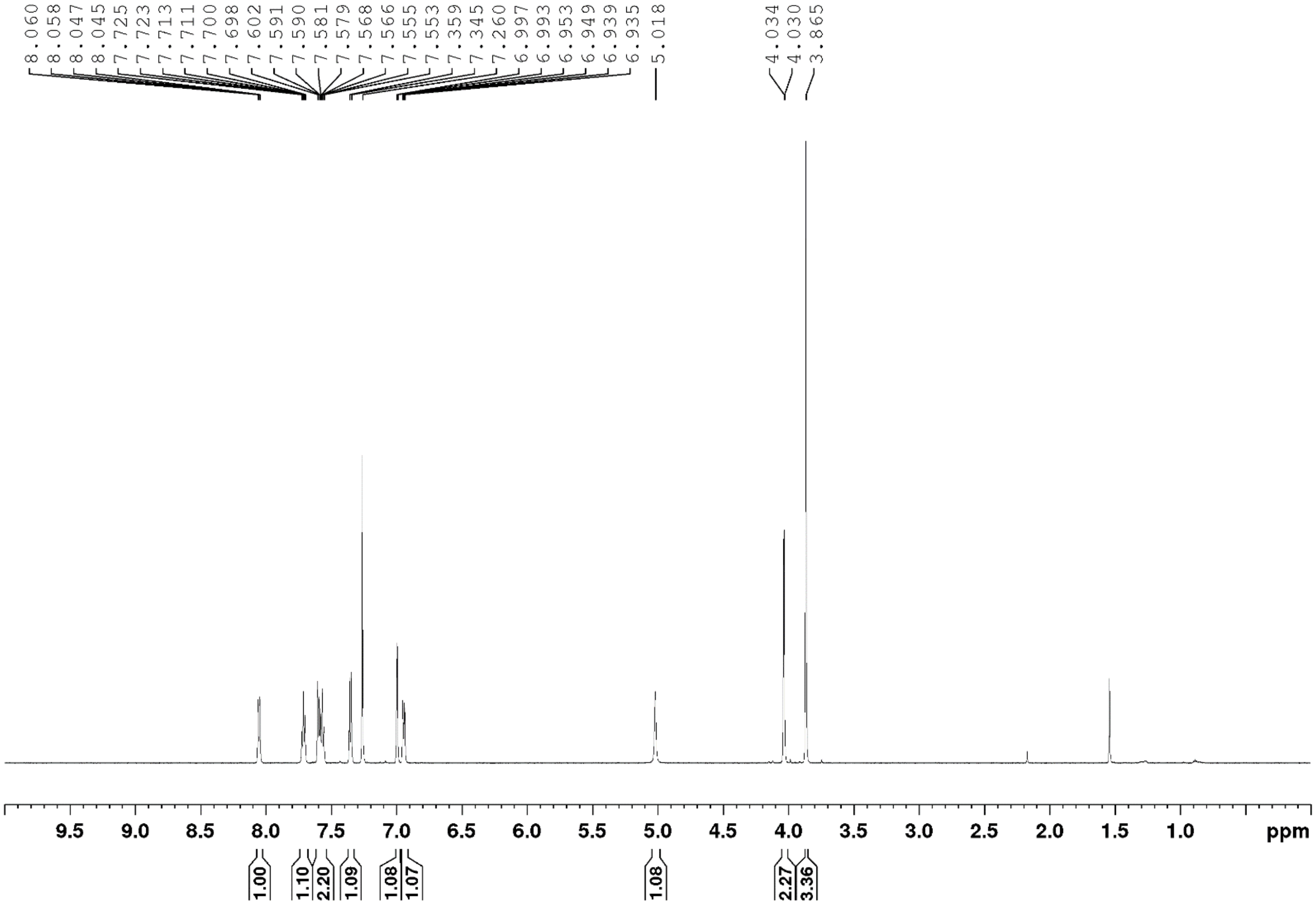

8.060
8.058
8.047
8.045
7.725
7.723
7.713
7.711
7.700
7.698
7.602
7.591
7.590
7.581
7.579
7.568
7.566
7.555
7.553
7.359
7.345
7.260
6.997
6.993
6.953
6.949
6.939
6.935
5.018
4.034
4.030
3.865
9.5
9.0
8.5
8.0
7.5
7.0
6.5
6.0
5.5
5.0
4.5
4.0
3.5
3.0
2.5
2.0
1.5
1.0
ppm
1.00
1.10
2.20
1.09
1.08
1.07
1.08
2.27
3.36

^{13}C NMR of 2d

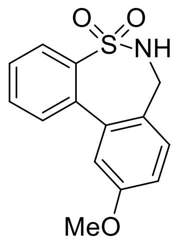

O
O
S
NH
MeO

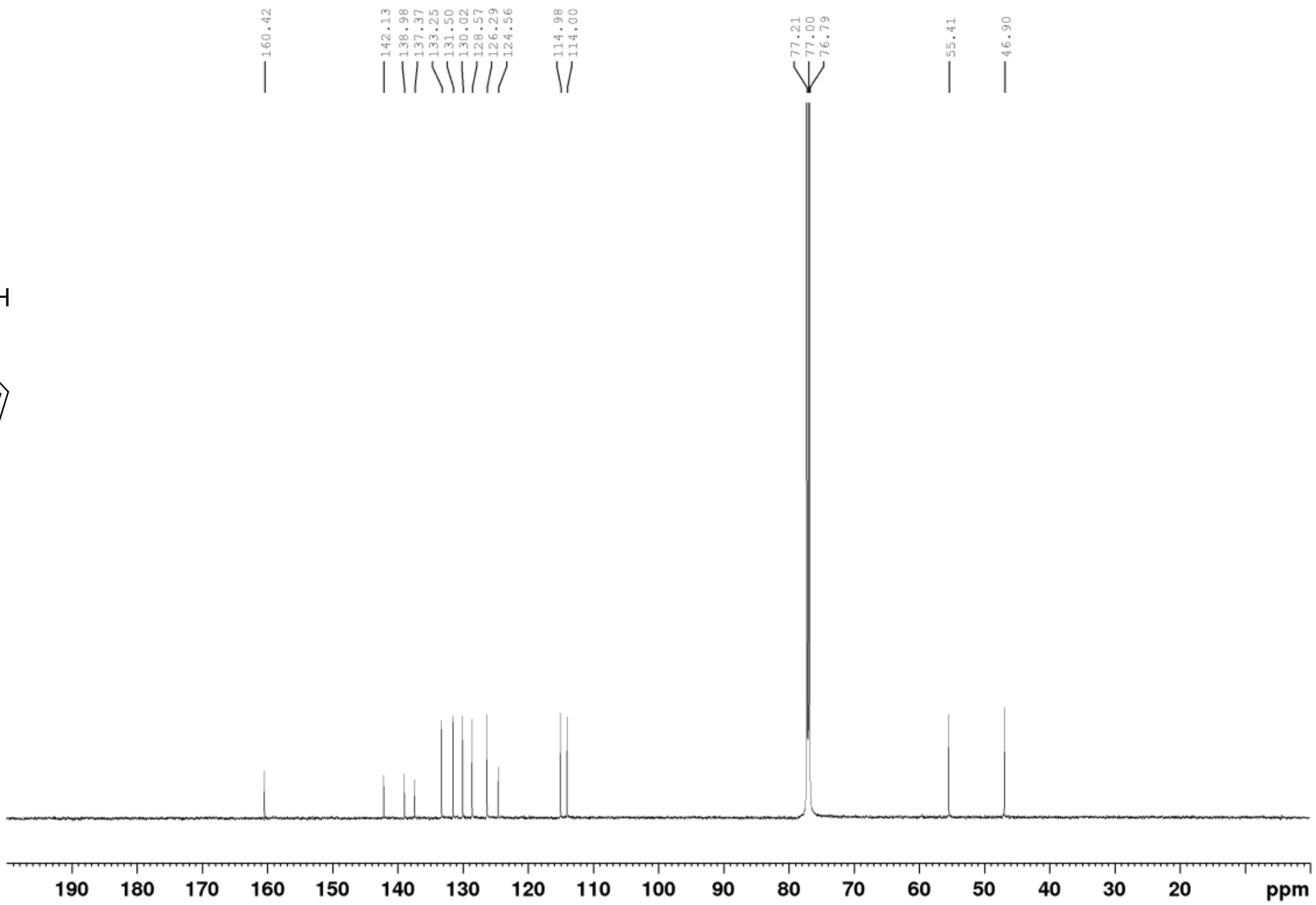

160.42
142.13
138.98
137.37
133.25
131.50
130.02
128.57
126.29
124.56
114.98
114.00
77.21
77.00
76.79
55.41
46.90
190
180
170
160
150
140
130
120
110
100
90
80
70
60
50
40
30
20
ppm

^{1}H NMR of 2e

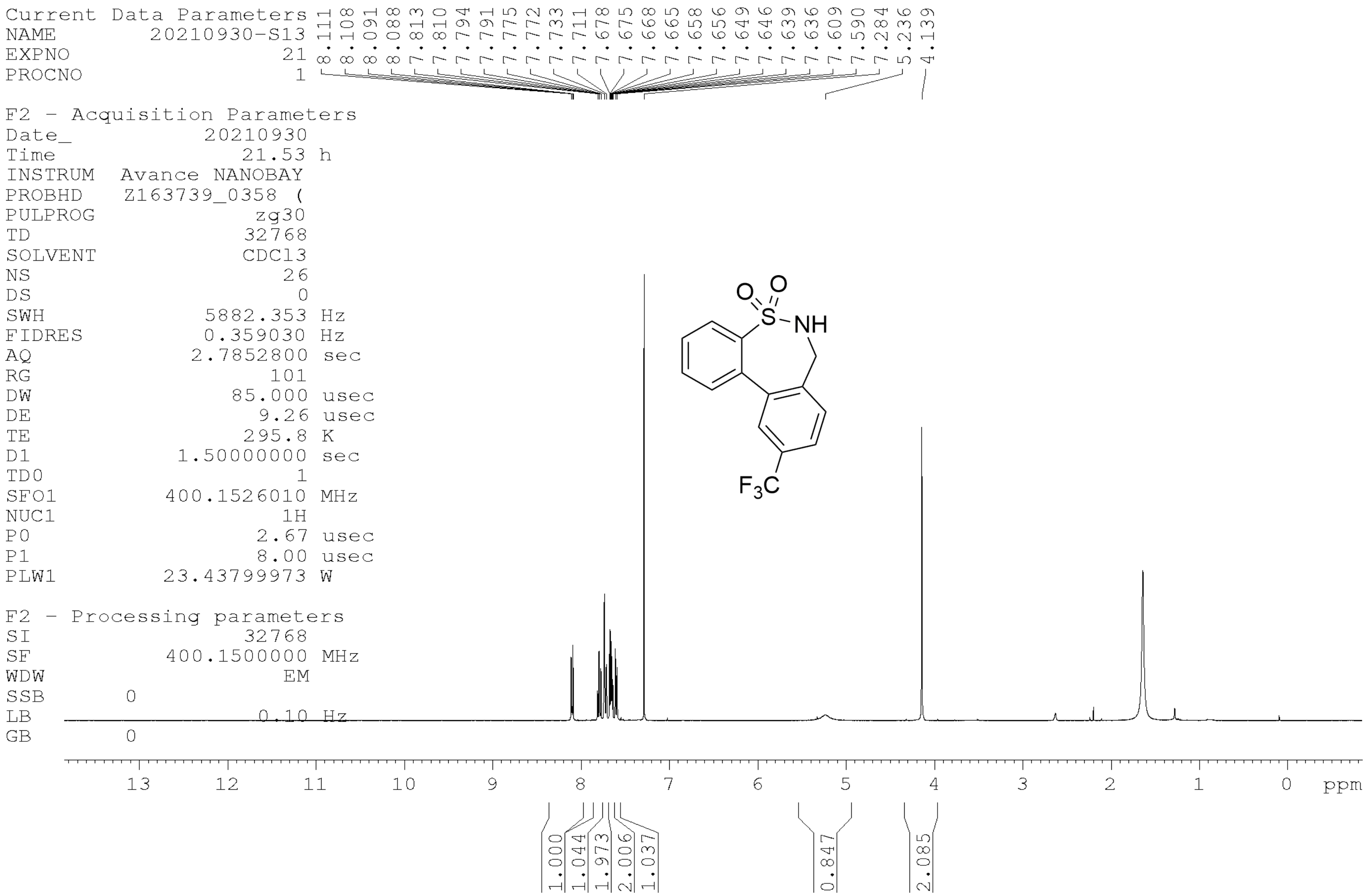
Current Data Parameters
NAME 20210930-S13
EXPNO 21
PROCNO 1
F2 - Acquisition Parameters
Date_ 20210930
Time 21.53 h
INSTRUM Avance NANOBAY
PROBHD Z163739_0358 (
PULPROG zg30
TD 32768
SOLVENT CDCl3
NS 26
DS 0
SWH 5882.353 Hz
FIDRES 0.359030 Hz
AQ 2.7852800 sec
RG 101
DW 85.000 usec
DE 9.26 usec
TE 295.8 K
D1 1.50000000 sec
TD0 1
SFO1 400.1526010 MHz
NUC1 1H
P0 2.67 usec
P1 8.00 usec
PLW1 23.43799973 W
F2 - Processing parameters
SI 32768
SF 400.1500000 MHz
WDW EM
SSB 0
LB 0.10 Hz
GB 0
8.111 8.108 8.091 8.088 7.813 7.810 7.794 7.791 7.775 7.772 7.733 7.711 7.678 7.675 7.668 7.665 7.658 7.656 7.649 7.646 7.639 7.636 7.609 7.590 7.284 5.236 4.139
O O S NH
F3C
13 12 11 10 9 8 7 6 5 4 3 2 1 0 ppm
1.000 1.044 1.973 2.006 1.037 0.847 2.085

^{13}C NMR of 2e

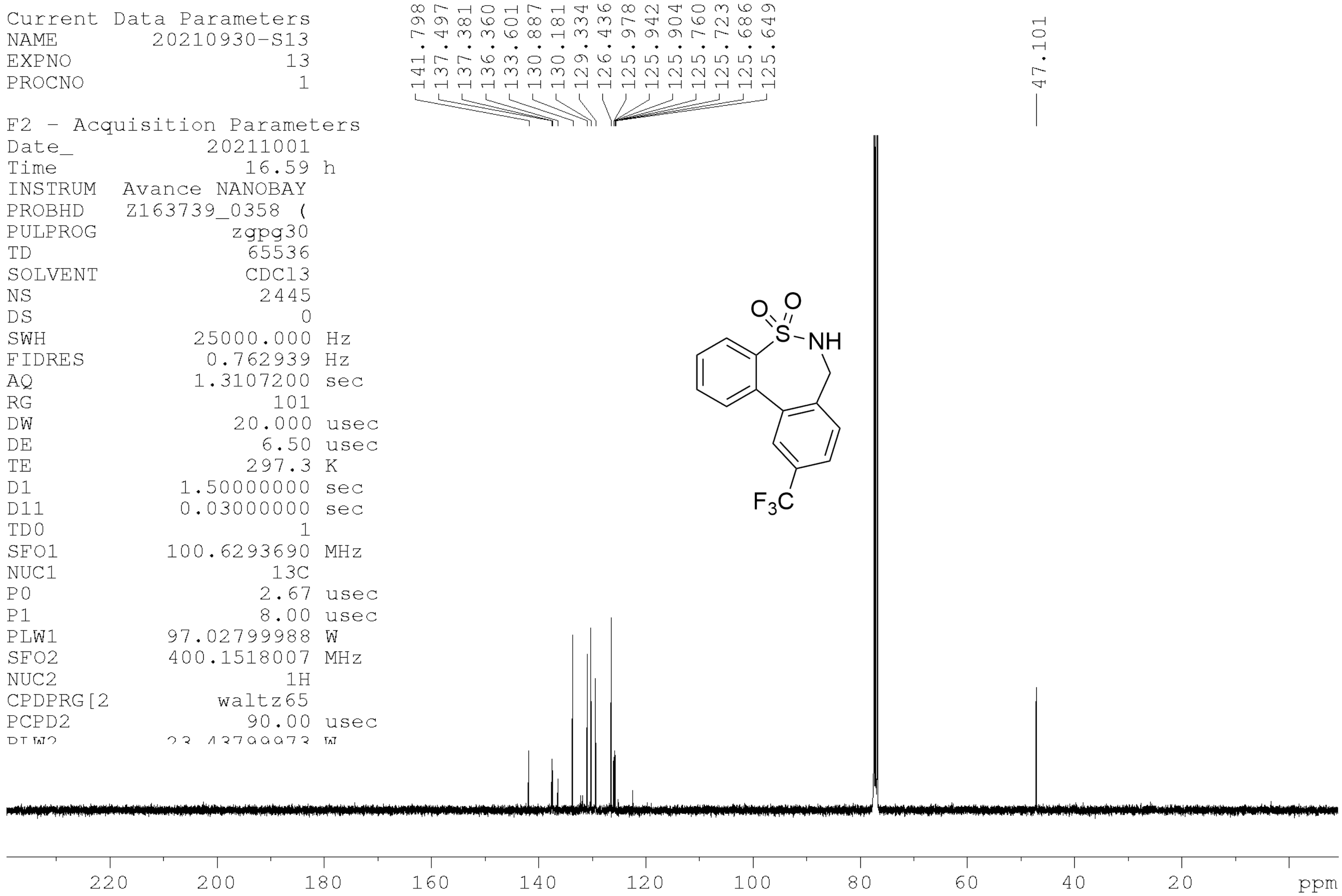
Current Data Parameters
NAME 20210930-S13
EXPNO 13
PROCNO 1
F2 - Acquisition Parameters
Date_ 20211001
Time 16.59 h
INSTRUM Avance NANOBAY
PROBHD Z163739_0358 (
PULPROG zgpg30
TD 65536
SOLVENT CDCl3
NS 2445
DS 0
SWH 25000.000 Hz
FIDRES 0.762939 Hz
AQ 1.3107200 sec
RG 101
DW 20.000 usec
DE 6.50 usec
TE 297.3 K
D1 1.50000000 sec
D11 0.03000000 sec
TD0 1
SFO1 100.6293690 MHz
NUC1 13C
P0 2.67 usec
P1 8.00 usec
PLW1 97.02799988 W
SFO2 400.1518007 MHz
NUC2 1H
CPDPRG[2 waltz65
PCPD2 90.00 usec
141.798
137.497
137.381
136.360
133.601
130.887
130.181
129.334
126.436
125.978
125.942
125.904
125.760
125.723
125.686
125.649
47.101
O
O
S
NH
F3C
220
200
180
160
140
120
100
80
60
40
20
ppm

^{1}H NMR of 2f

```
Current Data Parameters
NAME        20210909-S-14
EXPNO                   8
PROCNO                  1

F2 - Acquisition Parameters
Date_          20210909
Time              21.41 h
INSTRUM  Avance NANOBAY
PROBHD   Z163739_0358 (
PULPROG            zg30
TD                32768
SOLVENT           CDCl3
NS                   29
DS                    0
SWH            5882.353 Hz
FIDRES         0.359030 Hz
AQ            2.7852800 sec
RG                  101
DW               85.000 usec
DE                 9.26 usec
TE                297.4 K
D1           1.50000000 sec
TD0                   1
SFO1        400.1526010 MHz
NUC1                 1H
P0                 2.67 usec
P1                 8.00 usec
PLW1        23.43799973 W

F2 - Processing parameters
SI                32768
SF          400.1500000 MHz
WDW                  EM
SSB      0
LB       [illegible]
```

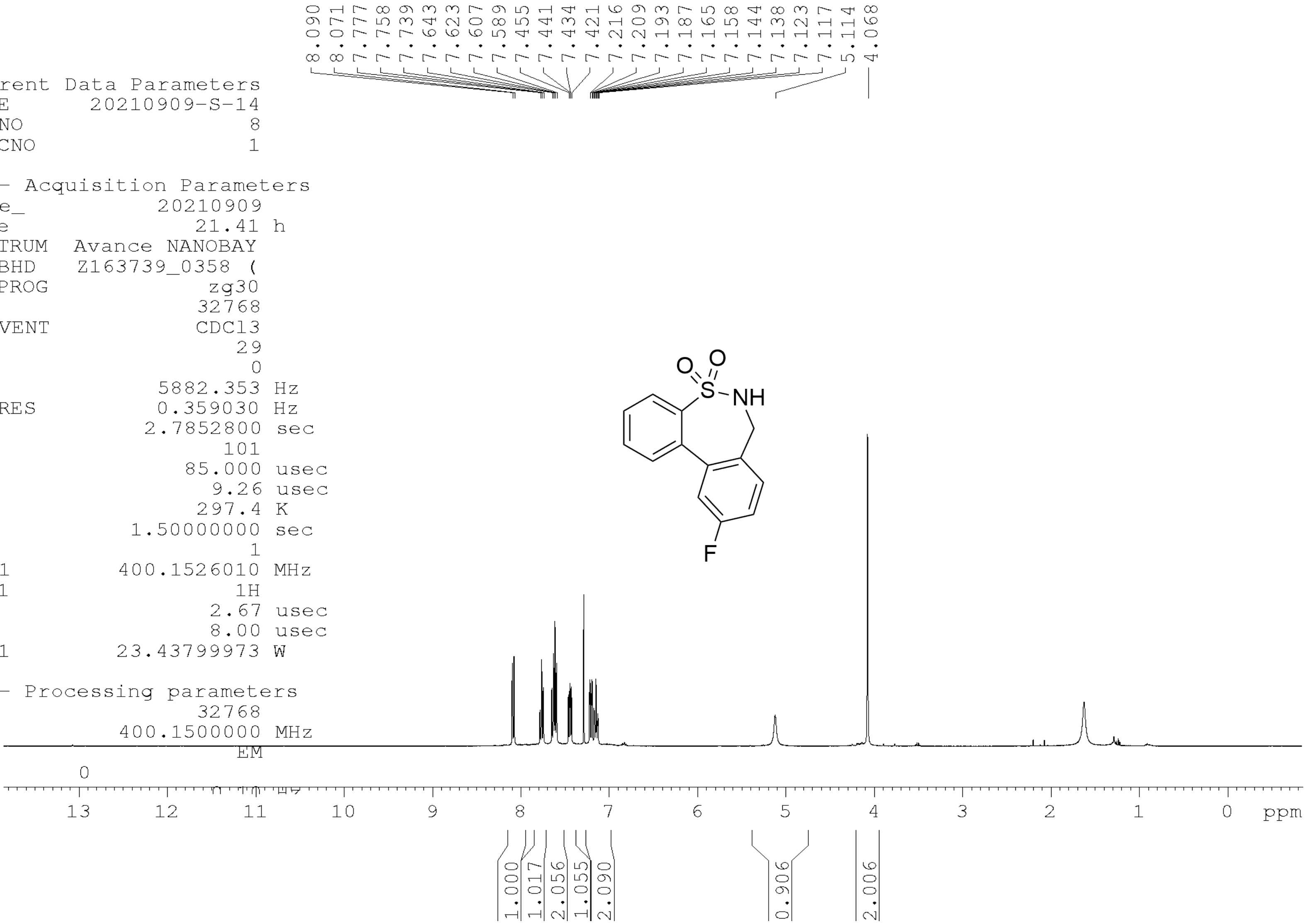

^{13}C NMR of 2f

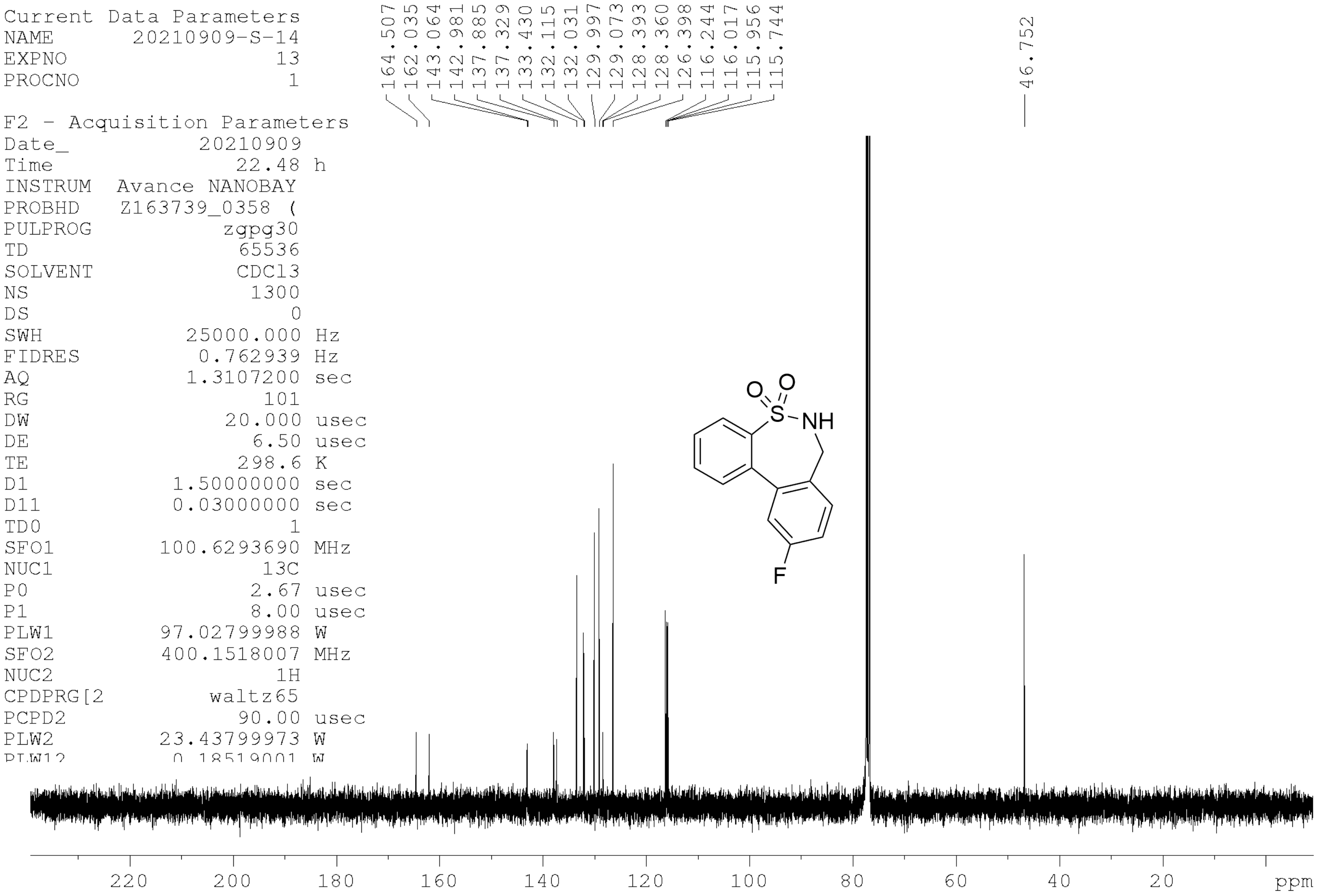
Current Data Parameters
NAME 20210909-S-14
EXPNO 13
PROCNO 1
F2 - Acquisition Parameters
Date_ 20210909
Time 22.48 h
INSTRUM Avance NANOBAY
PROBHD Z163739_0358 (
PULPROG zgpg30
TD 65536
SOLVENT CDCl3
NS 1300
DS 0
SWH 25000.000 Hz
FIDRES 0.762939 Hz
AQ 1.3107200 sec
RG 101
DW 20.000 usec
DE 6.50 usec
TE 298.6 K
D1 1.50000000 sec
D11 0.03000000 sec
TD0 1
SFO1 100.6293690 MHz
NUC1 13C
P0 2.67 usec
P1 8.00 usec
PLW1 97.02799988 W
SFO2 400.1518007 MHz
NUC2 1H
CPDPRG[2 waltz65
PCPD2 90.00 usec
PLW2 23.43799973 W
164.507
162.035
143.064
142.981
137.885
137.329
133.430
132.115
132.031
129.997
129.073
128.393
128.360
126.398
116.244
116.017
115.956
115.744
46.752
O
O
S
NH
F
220
200
180
160
140
120
100
80
60
40
20
ppm

^{1}H NMR of 2g

Current Data Parameters
NAME 20211112-S19
EXPNO 3
PROCNO 1

F2 - Acquisition Parameters
Date_ 20211112
Time 20.45 h
INSTRUM Avance NANOBAY
PROBHD Z163739_0358 (
PULPROG zg30
TD 32768
SOLVENT Acetone
NS 60
DS 0
SWH 5882.353 Hz
FIDRES 0.359030 Hz
AQ 2.7852800 sec
RG 101
DW 85.000 usec
DE 9.26 usec
TE 295.7 K
D1 1.50000000 sec
TD0 1
SFO1 400.1526010 MHz
NUC1 1H
P0 2.67 usec
P1 8.00 usec
PLW1 23.43799973 W

F2 - Processing parameters
SI 32768
SF 400.1500000 MHz
WDW EM

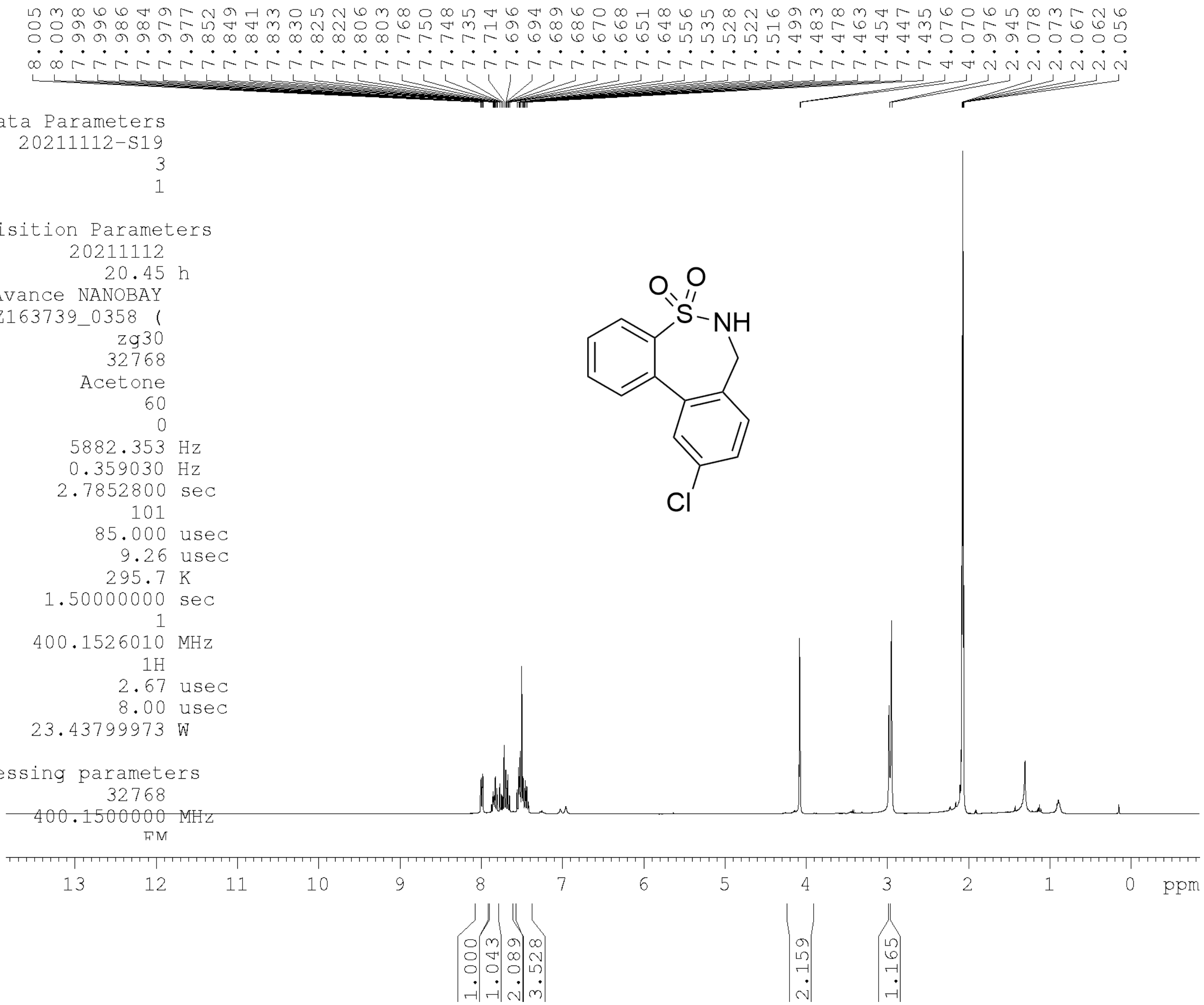

^{13}C NMR of 2g

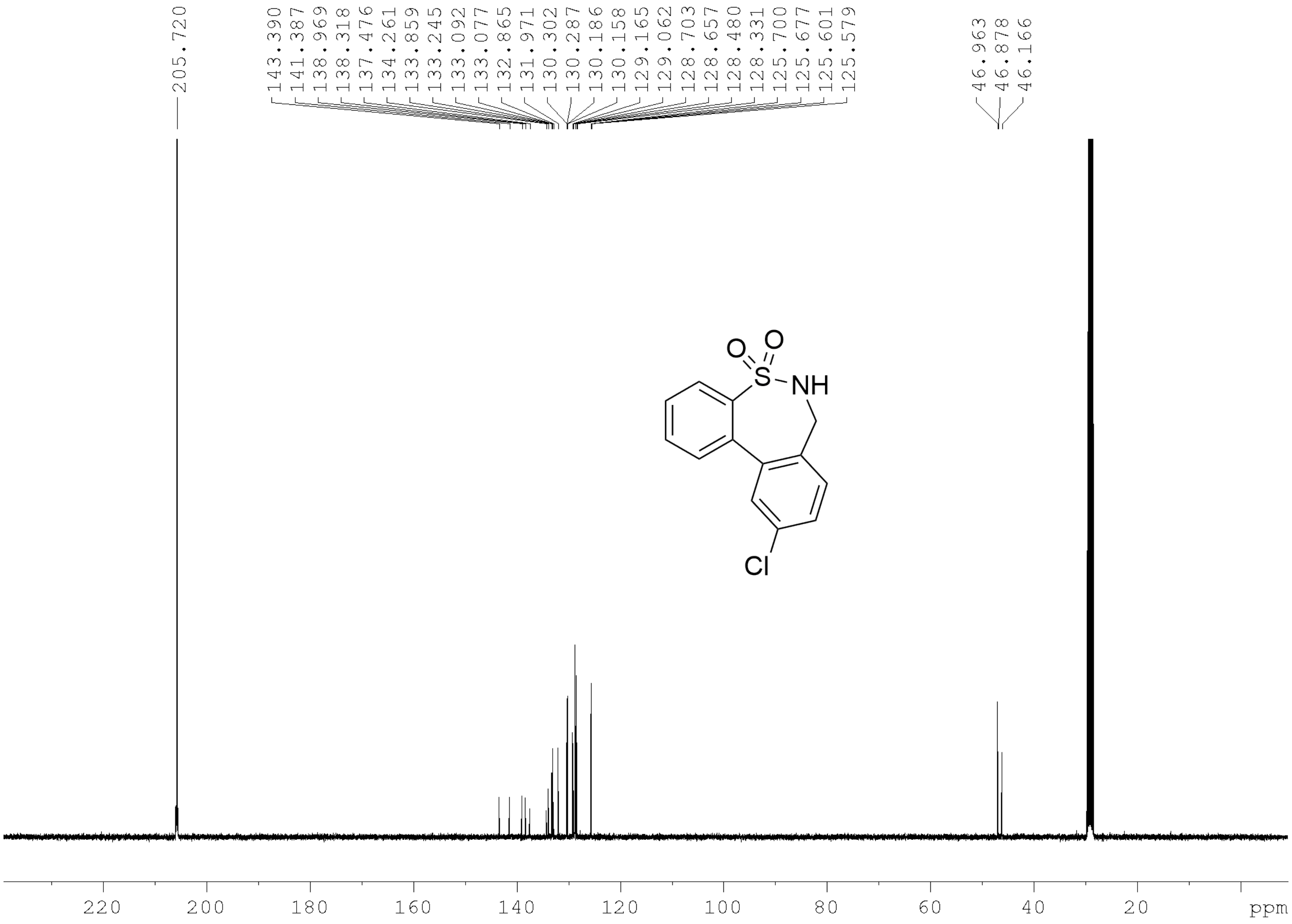
205.720
143.390
141.387
138.969
138.318
137.476
134.261
133.859
133.245
133.092
133.077
132.865
131.971
130.302
130.287
130.186
130.158
129.165
129.062
128.703
128.657
128.480
128.331
125.700
125.677
125.601
125.579
46.963
46.878
46.166
O
O
S
NH
Cl
220
200
180
160
140
120
100
80
60
40
20
ppm

^{1}H NMR of 2h

Current Data Parameters
NAME 20210825-S23 cyano C1
EXPNO 2
PROCNO 1

F2 - Acquisition Parameters
Date_ 20210825
Time 14.25 h
INSTRUM Avance NANOBAY
PROBHD Z163739_0358 (
PULPROG zg30
TD 32768
SOLVENT CDCl3
NS 2
DS 0
SWH 5882.353 Hz
FIDRES 0.359030 Hz
AQ 2.7852800 sec
RG 101
DW 85.000 usec
DE 9.26 usec
TE 295.8 K
D1 1.50000000 sec
TD0 1
SFO1 400.1526010 MHz
NUC1 1H
P0 2.67 usec
P1 8.00 usec
PLW1 23.43799973 W

F2 - Processing parameters
SI 32768
SF 400.1500000 MHz
WDW EM
SSB 0
LB 0.10 Hz

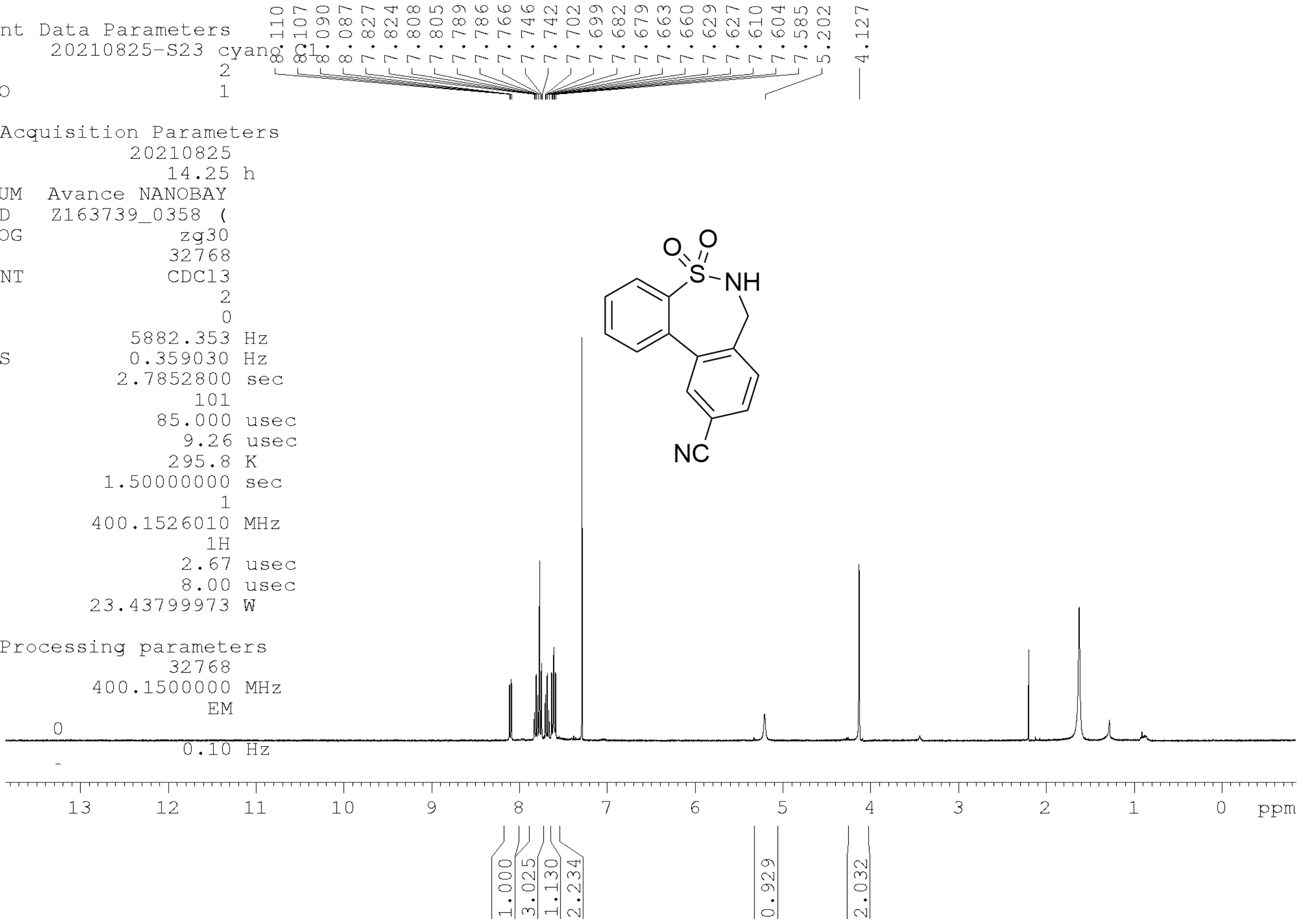

^{13}C NMR of 2h

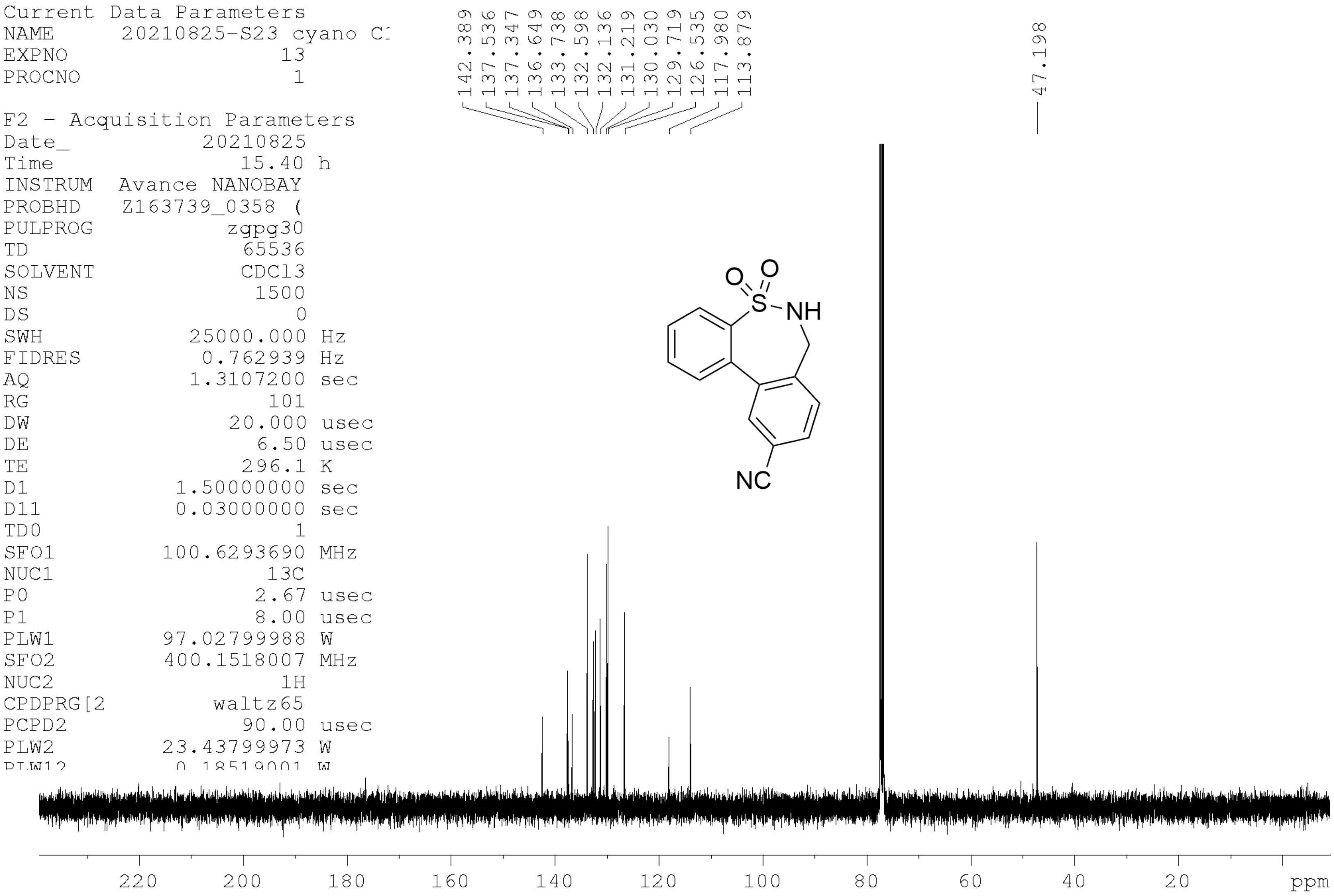

Current Data Parameters
NAME 20210825-S23 cyano [illegible]
EXPNO 13
PROCNO 1
F2 - Acquisition Parameters
Date_ 20210825
Time 15.40 h
INSTRUM Avance NANOBAY
PROBHD Z163739_0358 (
PULPROG zgpg30
TD 65536
SOLVENT CDCl3
NS 1500
DS 0
SWH 25000.000 Hz
FIDRES 0.762939 Hz
AQ 1.3107200 sec
RG 101
DW 20.000 usec
DE 6.50 usec
TE 296.1 K
D1 1.50000000 sec
D11 0.03000000 sec
TD0 1
SFO1 100.6293690 MHz
NUC1 13C
P0 2.67 usec
P1 8.00 usec
PLW1 97.02799988 W
SFO2 400.1518007 MHz
NUC2 1H
CPDPRG[2 waltz65
PCPD2 90.00 usec
PLW2 23.43799973 W
PLW12 0.18519001 W
142.389
137.536
137.347
136.649
133.738
132.598
132.136
131.219
130.030
129.719
126.535
117.980
113.879
47.198
O
O
S
NH
NC
220
200
180
160
140
120
100
80
60
40
20
ppm

^{1}H NMR of 2i

Current Data Parameters
NAME 20211008-S22
EXPNO 1
PROCNO 1

F2 - Acquisition Parameters
Date_ 20211008
Time 17.55
INSTRUM spect
PROBHD 5 mm BBO BB-1H
PULPROG zg30
TD 32768
SOLVENT CDCl3
NS 25
DS 0
SWH 6009.615 Hz
FIDRES 0.183399 Hz
AQ 2.7262976 sec
RG 512
DW 83.200 usec
DE 6.50 usec
TE 295.6 K
D1 1.50000000 sec
TD0 1

======== CHANNEL f1 ========
NUC1 1H
P1 14.00 usec
PL1 -1.00 dB
PL1W 7.55784369 W
SFO1 400.1326010 MHz

F2 - Processing parameters
SI 32768
SF 400.1300087 MHz
WDW EM

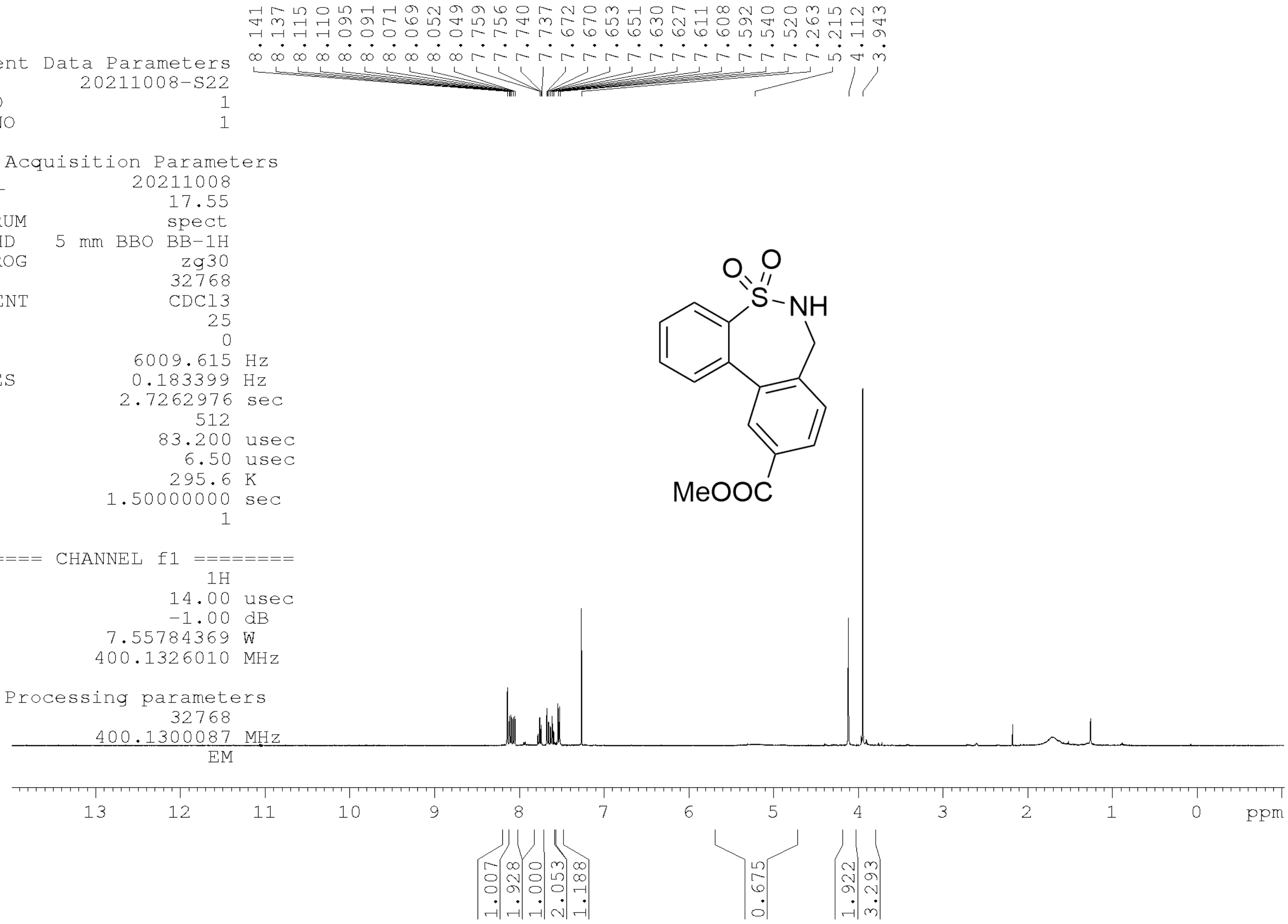

^{13}C NMR of 2i

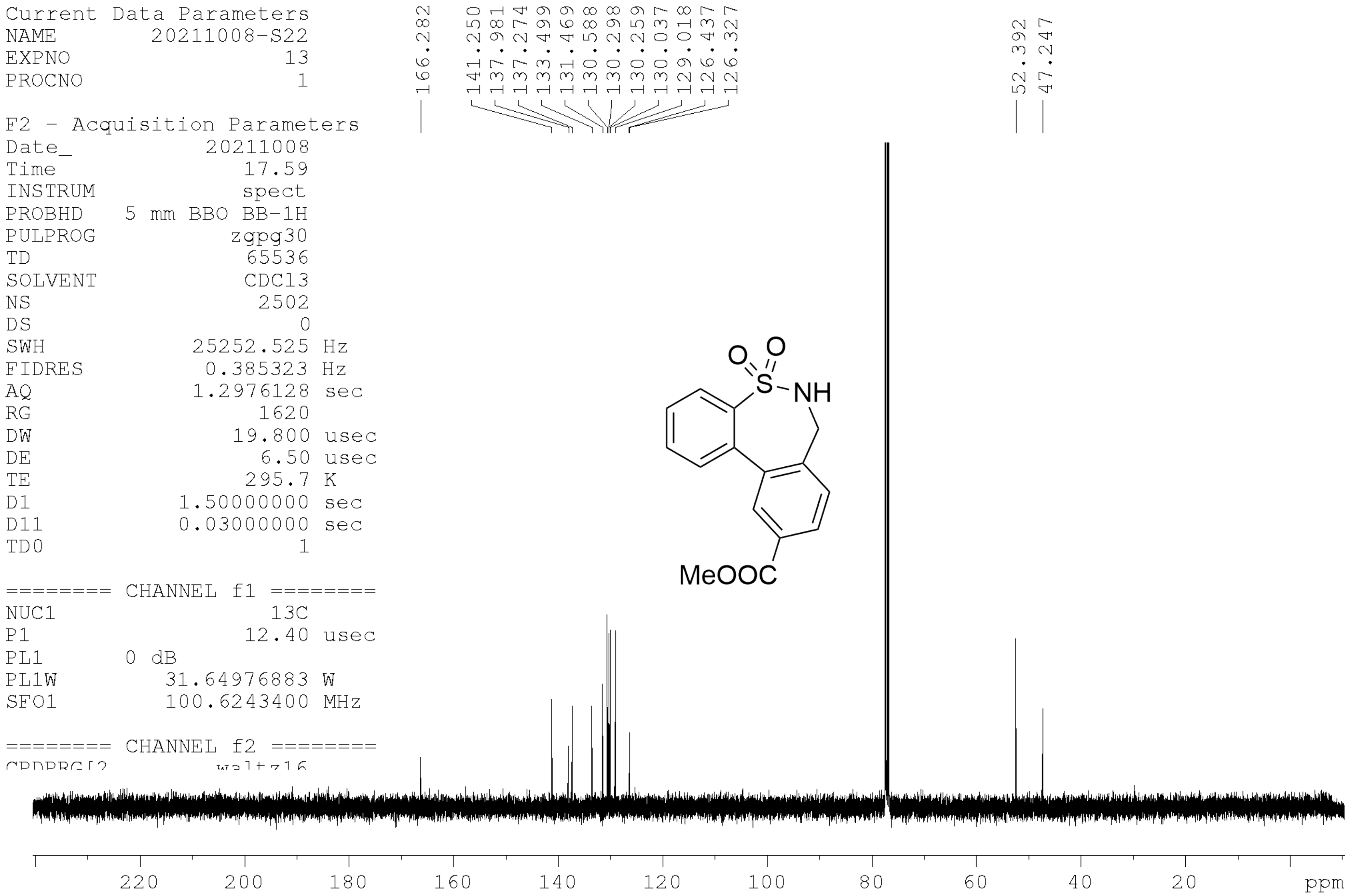

Current Data Parameters
NAME 20211008-S22
EXPNO 13
PROCNO 1
F2 - Acquisition Parameters
Date_ 20211008
Time 17.59
INSTRUM spect
PROBHD 5 mm BBO BB-1H
PULPROG zgpg30
TD 65536
SOLVENT CDCl3
NS 2502
DS 0
SWH 25252.525 Hz
FIDRES 0.385323 Hz
AQ 1.2976128 sec
RG 1620
DW 19.800 usec
DE 6.50 usec
TE 295.7 K
D1 1.50000000 sec
D11 0.03000000 sec
TD0 1
======== CHANNEL f1 ========
NUC1 13C
P1 12.40 usec
PL1 0 dB
PL1W 31.64976883 W
SFO1 100.6243400 MHz
======== CHANNEL f2 ========
166.282
141.250
137.981
137.274
133.499
131.469
130.588
130.298
130.259
130.037
129.018
126.437
126.327
52.392
47.247
O
O
S
NH
MeOOC
220
200
180
160
140
120
100
80
60
40
20
ppm

^{1}H NMR of 2j

Current Data Parameters
NAME 20210908-S-12 purif:
EXPNO 6
PROCNO 1

F2 - Acquisition Parameters
Date_ 20210908
Time 15.42 h
INSTRUM Avance NANOBAY
PROBHD Z163739_0358 (
PULPROG zg30
TD 32768
SOLVENT CDCl3
NS 24
DS 0
SWH 5882.353 Hz
FIDRES 0.359030 Hz
AQ 2.7852800 sec
RG 101
DW 85.000 usec
DE 9.26 usec
TE 295.5 K
D1 1.50000000 sec
TD0 1
SFO1 400.1526010 MHz
NUC1 1H
P0 2.67 usec
P1 8.00 usec
PLW1 23.43799973 W

F2 - Processing parameters
SI 32768
SF 400.1500000 MHz
WDW EM
SSB 0

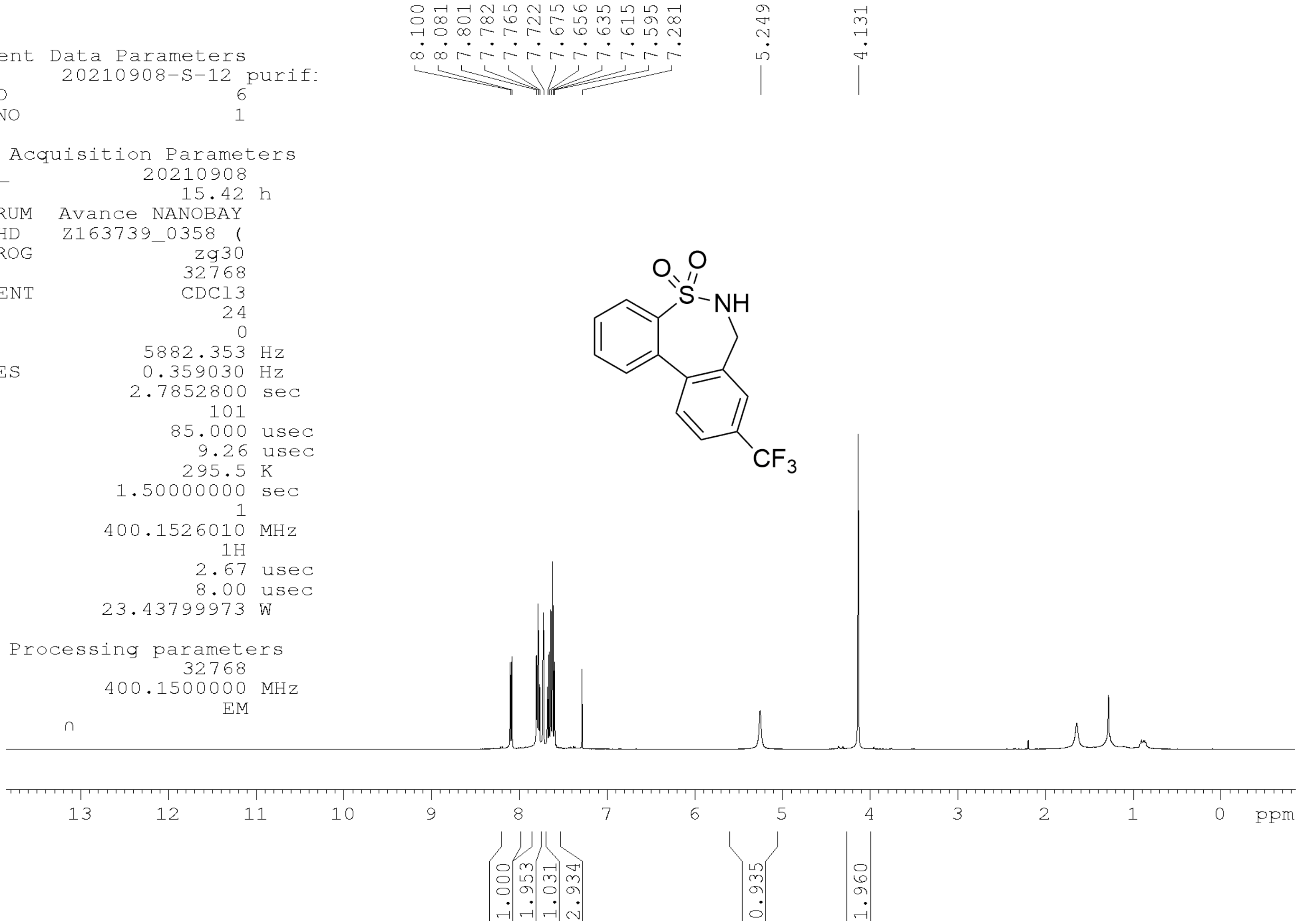

^{13}C NMR of 2j

Current Data Parameters
NAME 20210908-S-12 purifie
EXPNO 13
PROCNO 1

F2 - Acquisition Parameters
Date_ 20210908
Time 17.01 h
INSTRUM Avance NANOBAY
PROBHD Z163739_0358 (
PULPROG zgpg30
TD 65536
SOLVENT CDCl3
NS 1256
DS 0
SWH 25000.000 Hz
FIDRES 0.762939 Hz
AQ 1.3107200 sec
RG 101
DW 20.000 usec
DE 6.50 usec
TE 296.7 K
D1 1.50000000 sec
D11 0.03000000 sec
TD0 1
SFO1 100.6293690 MHz
NUC1 13C
P0 2.67 usec
P1 8.00 usec
PLW1 97.02799988 W
SFO2 400.1518007 MHz
NUC2 1H
CPDPRG[2 waltz65
PCPD2 90.00 usec
PLW2 23.43799973 W
PLW12 0.18519001 W

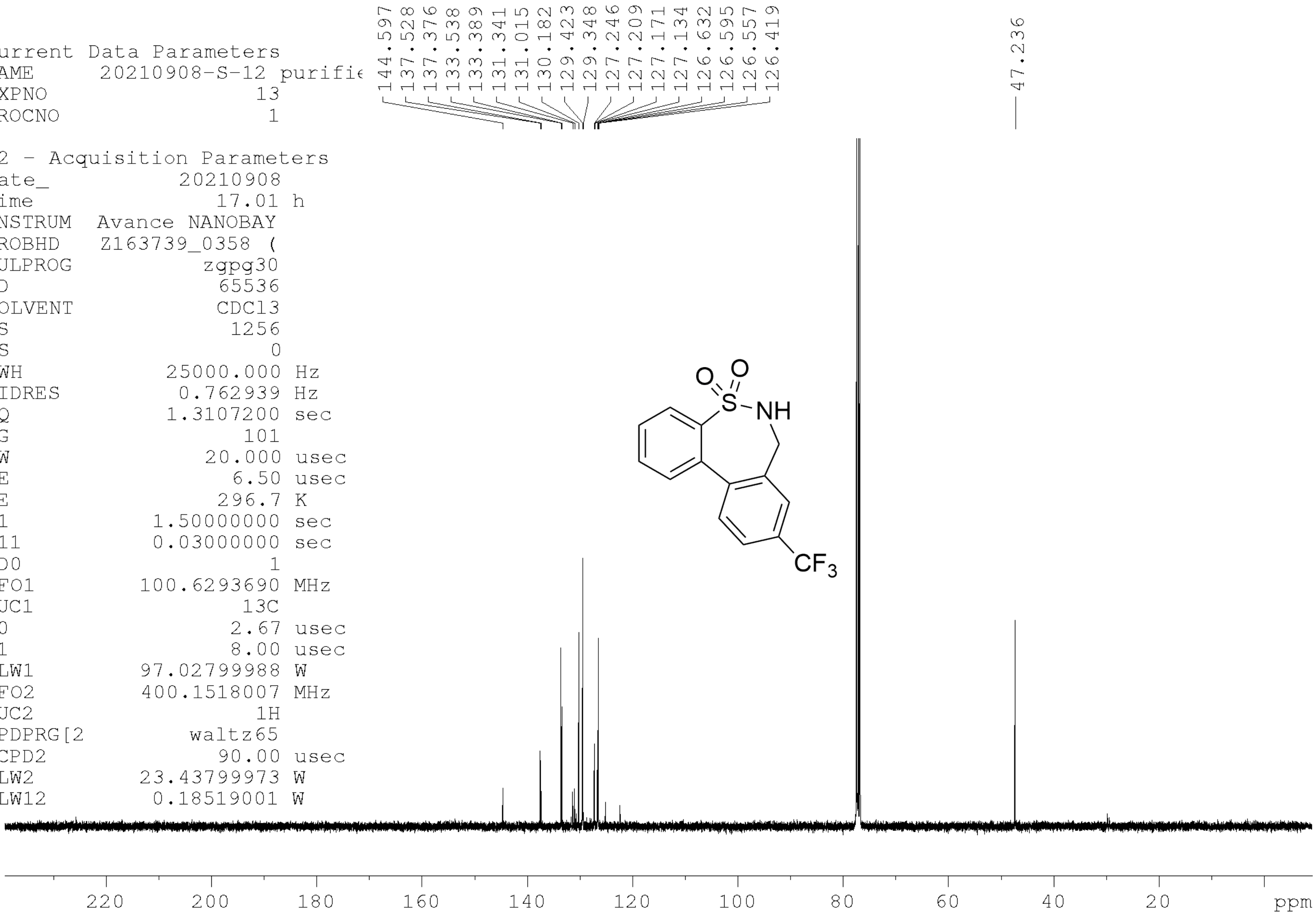

^{1}H NMR of 2k

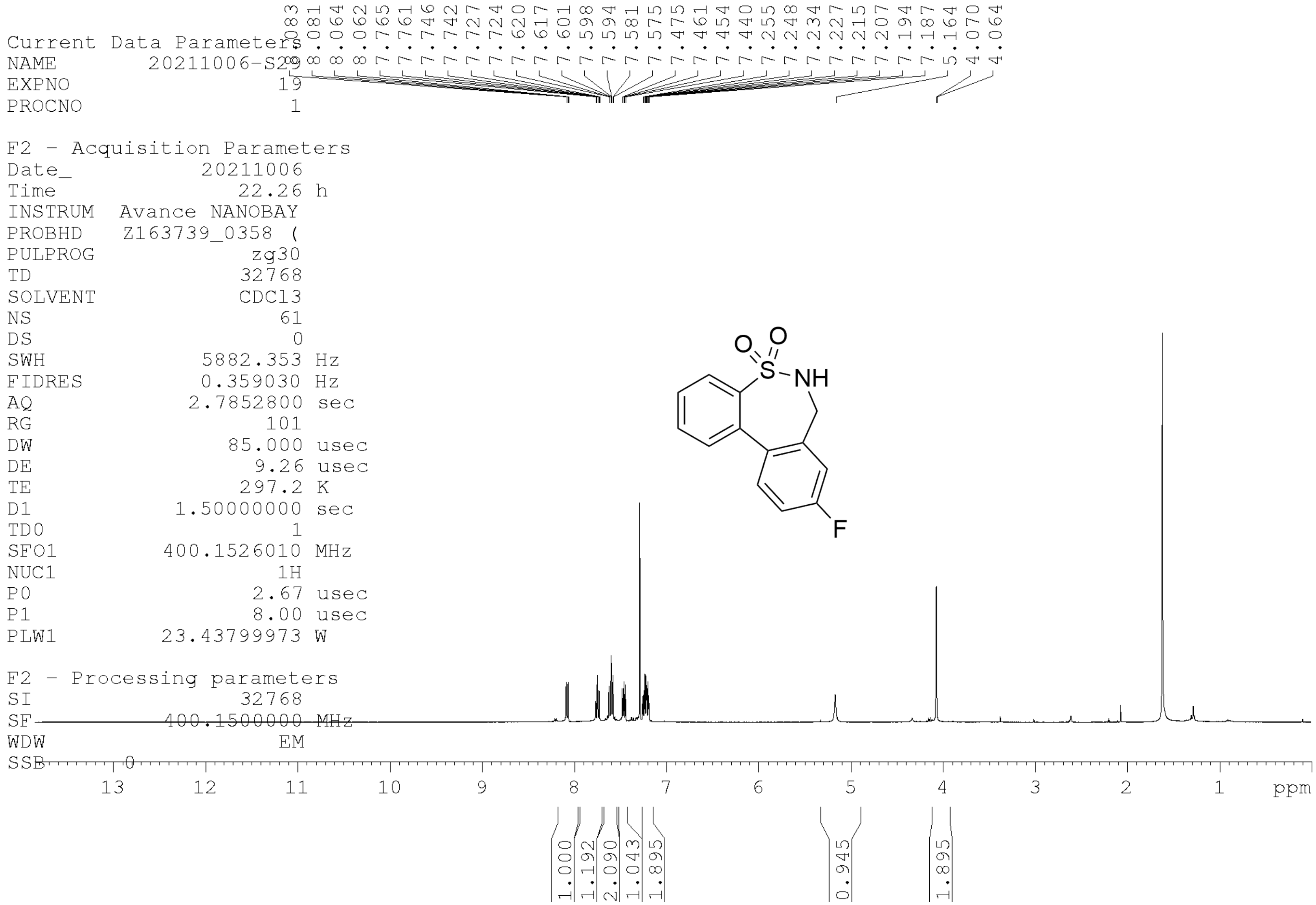
Current Data Parameters
NAME 20211006-S29
EXPNO 19
PROCNO 1
F2 - Acquisition Parameters
Date_ 20211006
Time 22.26 h
INSTRUM Avance NANOBAY
PROBHD Z163739_0358 (
PULPROG zg30
TD 32768
SOLVENT CDCl3
NS 61
DS 0
SWH 5882.353 Hz
FIDRES 0.359030 Hz
AQ 2.7852800 sec
RG 101
DW 85.000 usec
DE 9.26 usec
TE 297.2 K
D1 1.50000000 sec
TD0 1
SFO1 400.1526010 MHz
NUC1 1H
P0 2.67 usec
P1 8.00 usec
PLW1 23.43799973 W
F2 - Processing parameters
SI 32768
SF 400.1500000 MHz
WDW EM
SSB 0
8.083 8.081 8.064 8.062 7.765 7.761 7.746 7.742 7.727 7.724 7.620 7.617 7.601 7.598 7.594 7.581 7.575 7.475 7.461 7.454 7.440 7.255 7.248 7.234 7.227 7.215 7.207 7.194 7.187 5.164 4.070 4.064
O O
S
NH
F
13 12 11 10 9 8 7 6 5 4 3 2 1 ppm
1.000 1.192 2.090 1.043 1.895 0.945 1.895

^{13}C NMR of 2k

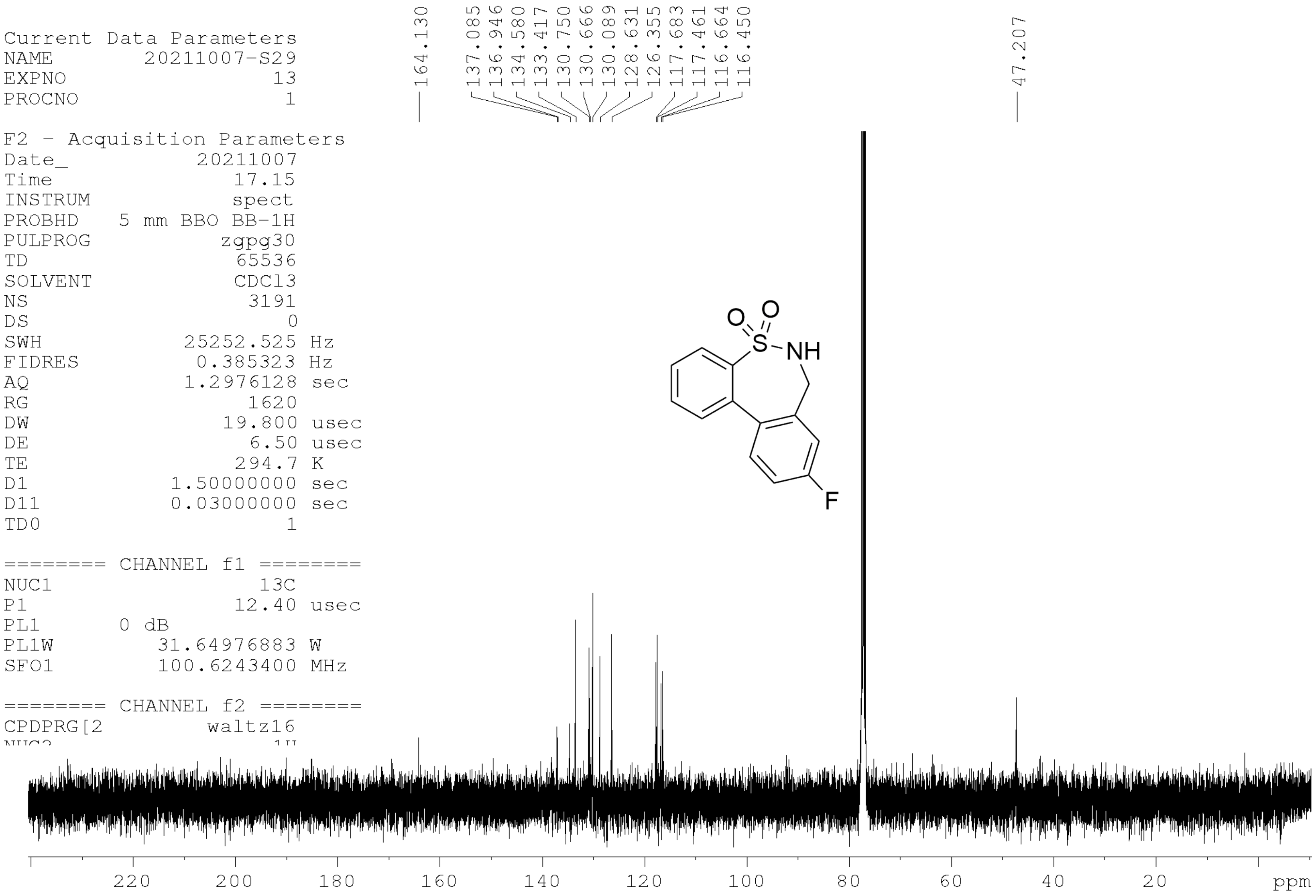
Current Data Parameters
NAME 20211007-S29
EXPNO 13
PROCNO 1
F2 - Acquisition Parameters
Date_ 20211007
Time 17.15
INSTRUM spect
PROBHD 5 mm BBO BB-1H
PULPROG zgpg30
TD 65536
SOLVENT CDCl3
NS 3191
DS 0
SWH 25252.525 Hz
FIDRES 0.385323 Hz
AQ 1.2976128 sec
RG 1620
DW 19.800 usec
DE 6.50 usec
TE 294.7 K
D1 1.50000000 sec
D11 0.03000000 sec
TD0 1
======== CHANNEL f1 ========
NUC1 13C
P1 12.40 usec
PL1 0 dB
PL1W 31.64976883 W
SFO1 100.6243400 MHz
======== CHANNEL f2 ========
CPDPRG[2 waltz16
164.130
137.085
136.946
134.580
133.417
130.750
130.666
130.089
128.631
126.355
117.683
117.461
116.664
116.450
47.207
O
O
S
NH
F
220
200
180
160
140
120
100
80
60
40
20
ppm

^{1}H NMR of 2l

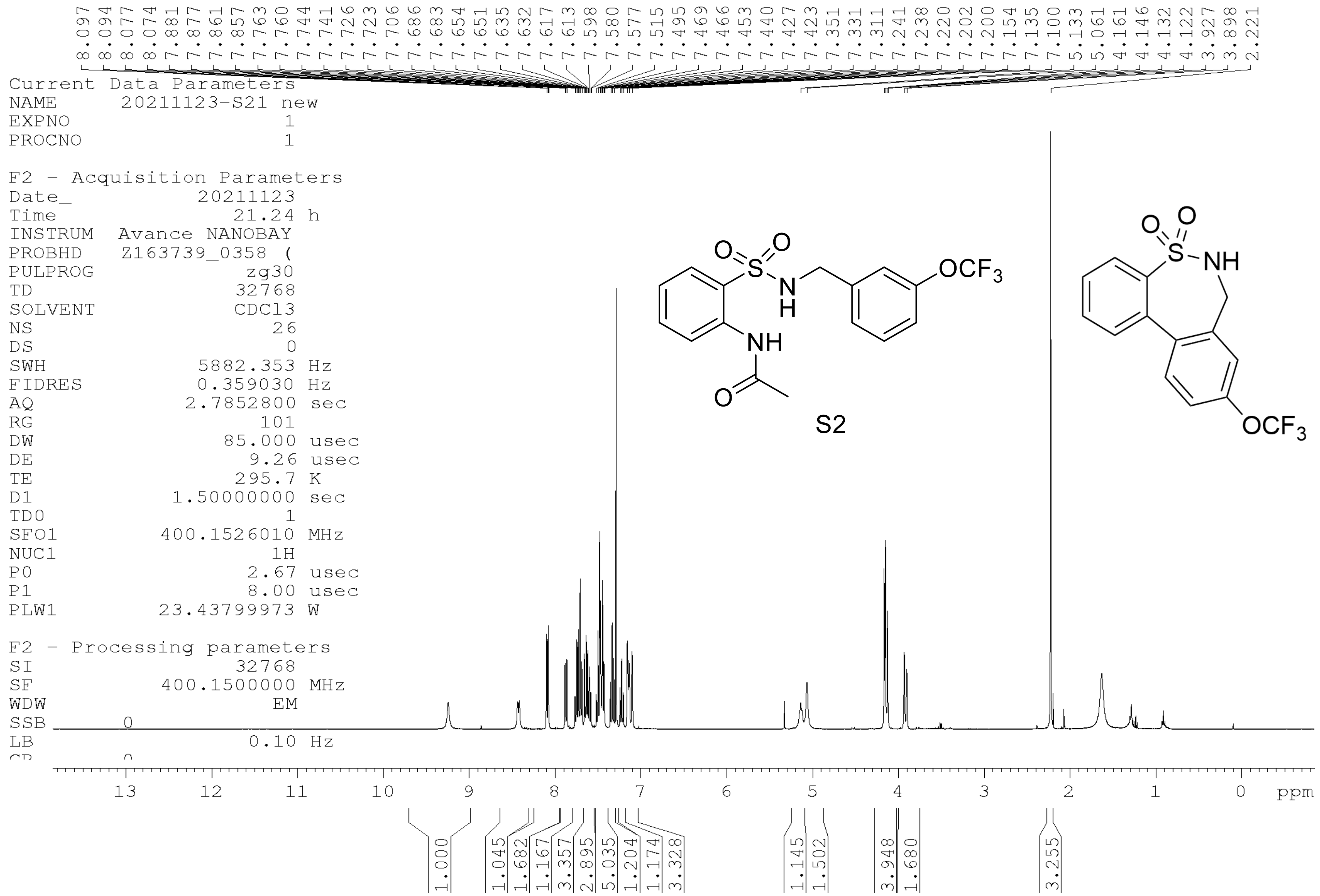
8.097 8.094 8.077 8.074 7.881 7.877 7.861 7.857 7.763 7.760 7.744 7.741 7.726 7.723 7.706 7.686 7.683 7.654 7.651 7.635 7.632 7.617 7.613 7.598 7.580 7.577 7.515 7.495 7.469 7.466 7.453 7.440 7.427 7.423 7.351 7.331 7.311 7.241 7.238 7.220 7.202 7.200 7.154 7.135 7.100 5.133 5.061 4.161 4.146 4.132 4.122 3.927 3.898 2.221
Current Data Parameters
NAME 20211123-S21 new
EXPNO 1
PROCNO 1
F2 - Acquisition Parameters
Date_ 20211123
Time 21.24 h
INSTRUM Avance NANOBAY
PROBHD Z163739_0358 (
PULPROG zg30
TD 32768
SOLVENT CDCl3
NS 26
DS 0
SWH 5882.353 Hz
FIDRES 0.359030 Hz
AQ 2.7852800 sec
RG 101
DW 85.000 usec
DE 9.26 usec
TE 295.7 K
D1 1.50000000 sec
TD0 1
SFO1 400.1526010 MHz
NUC1 1H
P0 2.67 usec
P1 8.00 usec
PLW1 23.43799973 W
F2 - Processing parameters
SI 32768
SF 400.1500000 MHz
WDW EM
SSB 0
LB 0.10 Hz
OCF3
NH
S2
O
S
NH
OCF3
13 12 11 10 9 8 7 6 5 4 3 2 1 0 ppm
1.000 1.045 1.682 1.167 3.357 2.895 5.035 1.204 1.174 3.328 1.145 1.502 3.948 1.680 3.255

^{13}C NMR of 2l

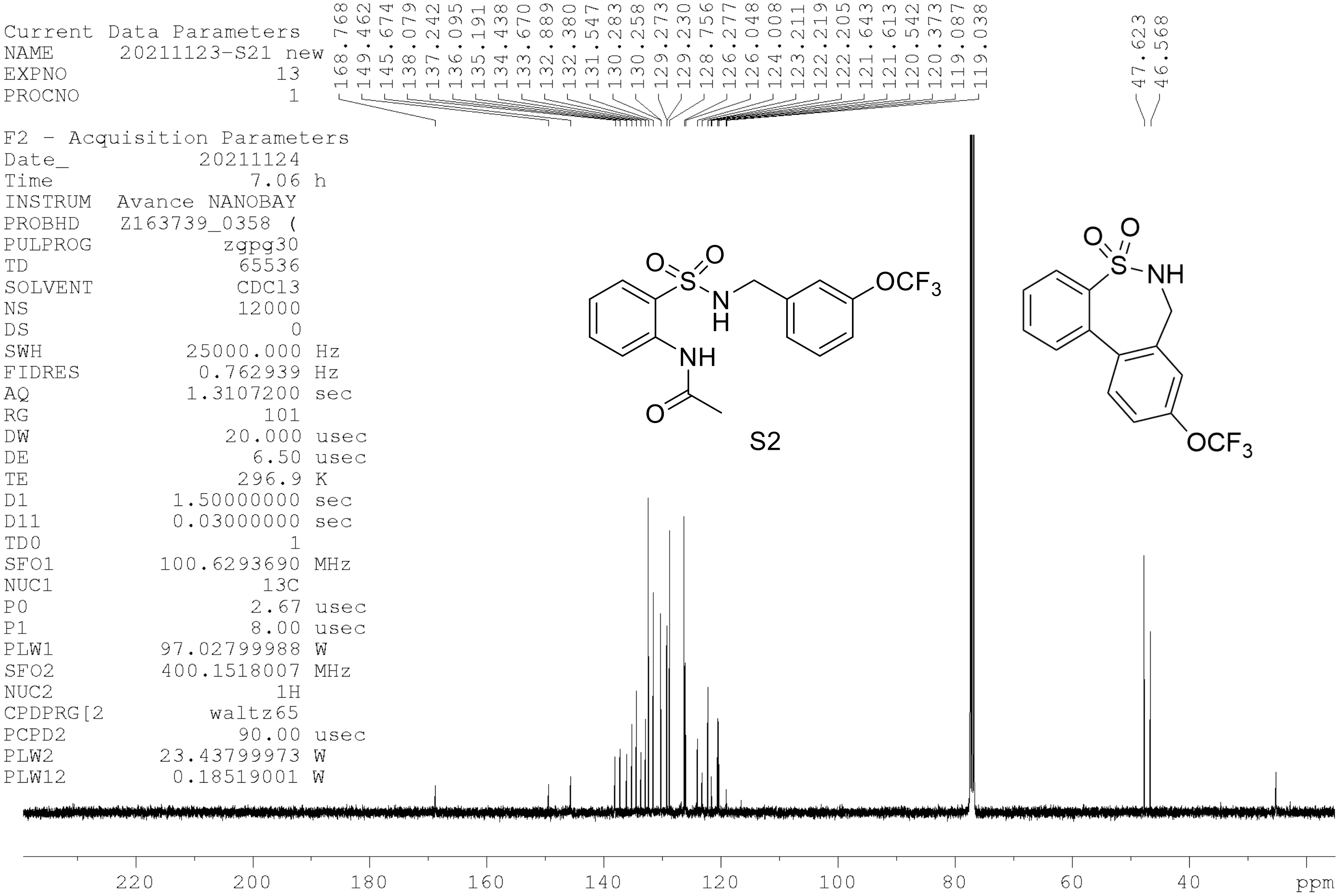
Current Data Parameters
NAME 20211123-S21 new
EXPNO 13
PROCNO 1
F2 - Acquisition Parameters
Date_ 20211124
Time 7.06 h
INSTRUM Avance NANOBAY
PROBHD Z163739_0358 (
PULPROG zgpg30
TD 65536
SOLVENT CDCl3
NS 12000
DS 0
SWH 25000.000 Hz
FIDRES 0.762939 Hz
AQ 1.3107200 sec
RG 101
DW 20.000 usec
DE 6.50 usec
TE 296.9 K
D1 1.50000000 sec
D11 0.03000000 sec
TD0 1
SFO1 100.6293690 MHz
NUC1 13C
P0 2.67 usec
P1 8.00 usec
PLW1 97.02799988 W
SFO2 400.1518007 MHz
NUC2 1H
CPDPRG[2 waltz65
PCPD2 90.00 usec
PLW2 23.43799973 W
PLW12 0.18519001 W
168.768
149.462
145.674
138.079
137.242
136.095
135.191
134.438
133.670
132.889
132.380
131.547
130.283
130.258
129.273
129.230
128.756
126.277
126.048
124.008
123.211
122.219
122.205
121.643
121.613
120.542
120.373
119.087
119.038
47.623
46.568
NH
O
S2
OCF3
220
200
180
160
140
120
100
80
60
40
ppm

^{1}H NMR of 2m

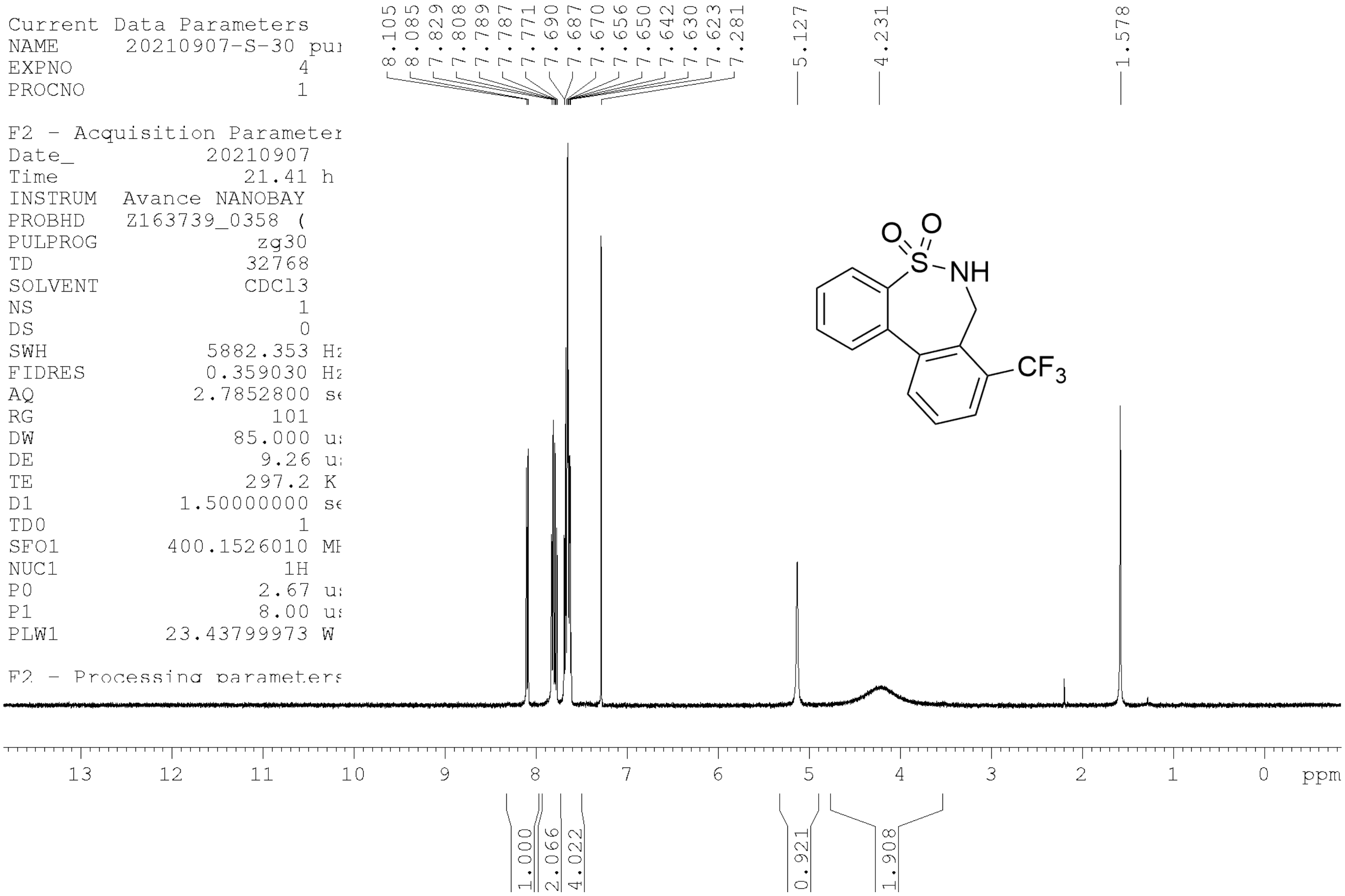
Current Data Parameters
NAME 20210907-S-30 pu
EXPNO 4
PROCNO 1
F2 - Acquisition Parameter
Date_ 20210907
Time 21.41 h
INSTRUM Avance NANOBAY
PROBHD Z163739_0358 (
PULPROG zg30
TD 32768
SOLVENT CDCl3
NS 1
DS 0
SWH 5882.353 Hz
FIDRES 0.359030 Hz
AQ 2.7852800 se
RG 101
DW 85.000 us
DE 9.26 us
TE 297.2 K
D1 1.50000000 se
TD0 1
SFO1 400.1526010 MH
NUC1 1H
P0 2.67 us
P1 8.00 us
PLW1 23.43799973 W
F2 - Processing parameters
8.105 8.085 7.829 7.808 7.789 7.787 7.771 7.690 7.687 7.670 7.656 7.650 7.642 7.630 7.623 7.281
5.127
4.231
1.578
O O S NH CF3
13 12 11 10 9 8 7 6 5 4 3 2 1 0 ppm
1.000 2.066 4.022
0.921
1.908

^{13}C NMR of 2m

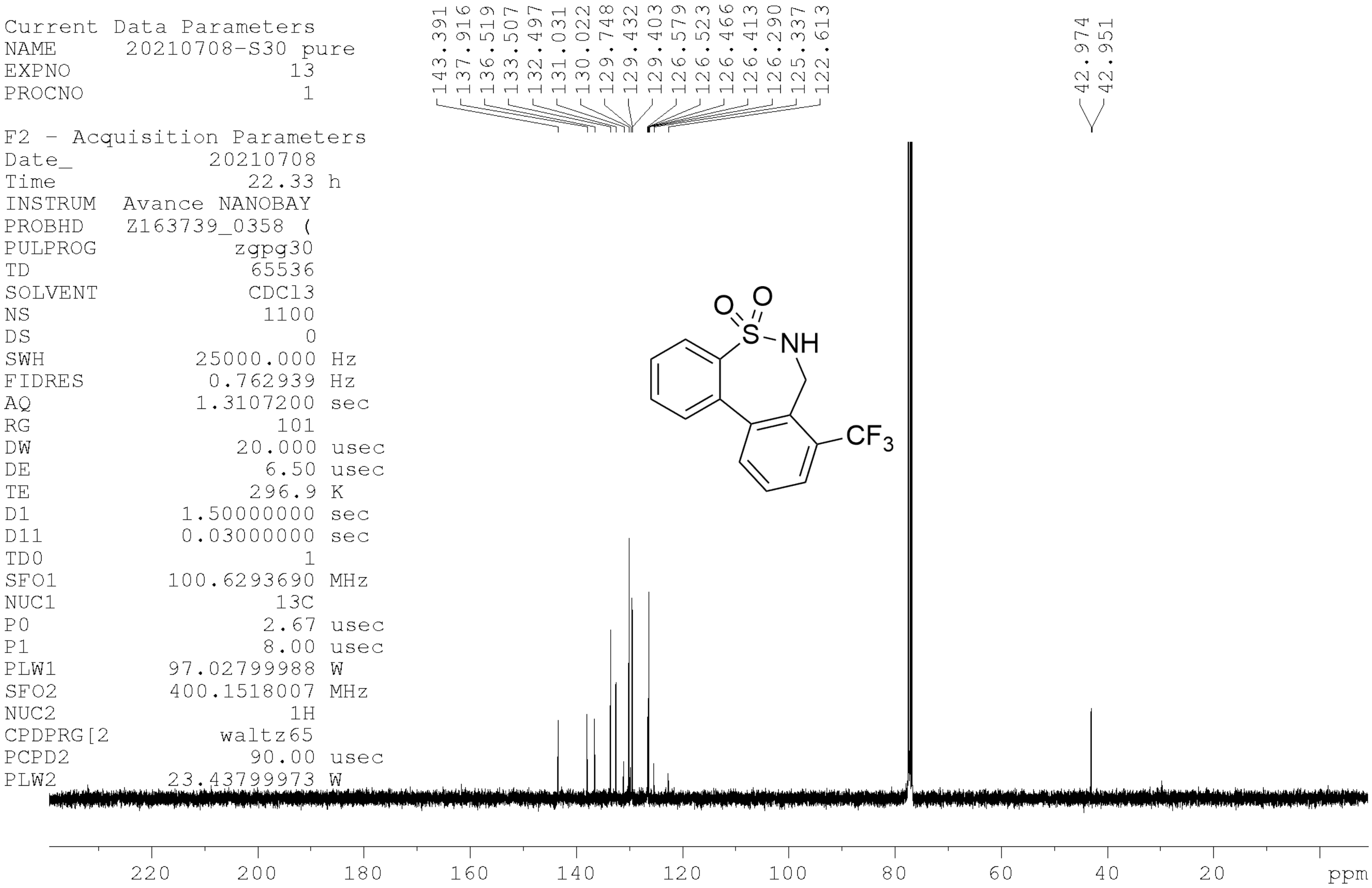
Current Data Parameters
NAME 20210708-S30 pure
EXPNO 13
PROCNO 1
F2 - Acquisition Parameters
Date_ 20210708
Time 22.33 h
INSTRUM Avance NANOBAY
PROBHD Z163739_0358 (
PULPROG zgpg30
TD 65536
SOLVENT CDCl3
NS 1100
DS 0
SWH 25000.000 Hz
FIDRES 0.762939 Hz
AQ 1.3107200 sec
RG 101
DW 20.000 usec
DE 6.50 usec
TE 296.9 K
D1 1.50000000 sec
D11 0.03000000 sec
TD0 1
SFO1 100.6293690 MHz
NUC1 13C
P0 2.67 usec
P1 8.00 usec
PLW1 97.02799988 W
SFO2 400.1518007 MHz
NUC2 1H
CPDPRG[2 waltz65
PCPD2 90.00 usec
PLW2 23.43799973 W
143.391
137.916
136.519
133.507
132.497
131.031
130.022
129.748
129.432
129.403
126.579
126.523
126.466
126.413
126.290
125.337
122.613
42.974
42.951
O
O
S
NH
CF3
220
200
180
160
140
120
100
80
60
40
20
ppm

^{1}H NMR of 2n

```
Current Data Parameters
NAME        20211123-S32
EXPNO                  6
PROCNO                 1

F2 - Acquisition Parameters
Date_          20211123
Time              21.08 h
INSTRUM  Avance NANOBAY
PROBHD   Z163739_0358 (
PULPROG            zg30
TD                32768
SOLVENT            DMSO
NS                   41
DS                    0
SWH            5882.353 Hz
FIDRES         0.359030 Hz
AQ            2.7852800 sec
RG                  101
DW               85.000 usec
DE                 9.26 usec
TE                295.6 K
D1           1.50000000 sec
TD0                   1
SFO1        400.1526010 MHz
NUC1                 1H
P0                 2.67 usec
P1                 8.00 usec
PLW1        23.43799973 W

F2 - Processing parameters
SI                32768
SF          400.1500000 MHz
WDW                  EM
SSB      0
LB                 0.10 Hz
```

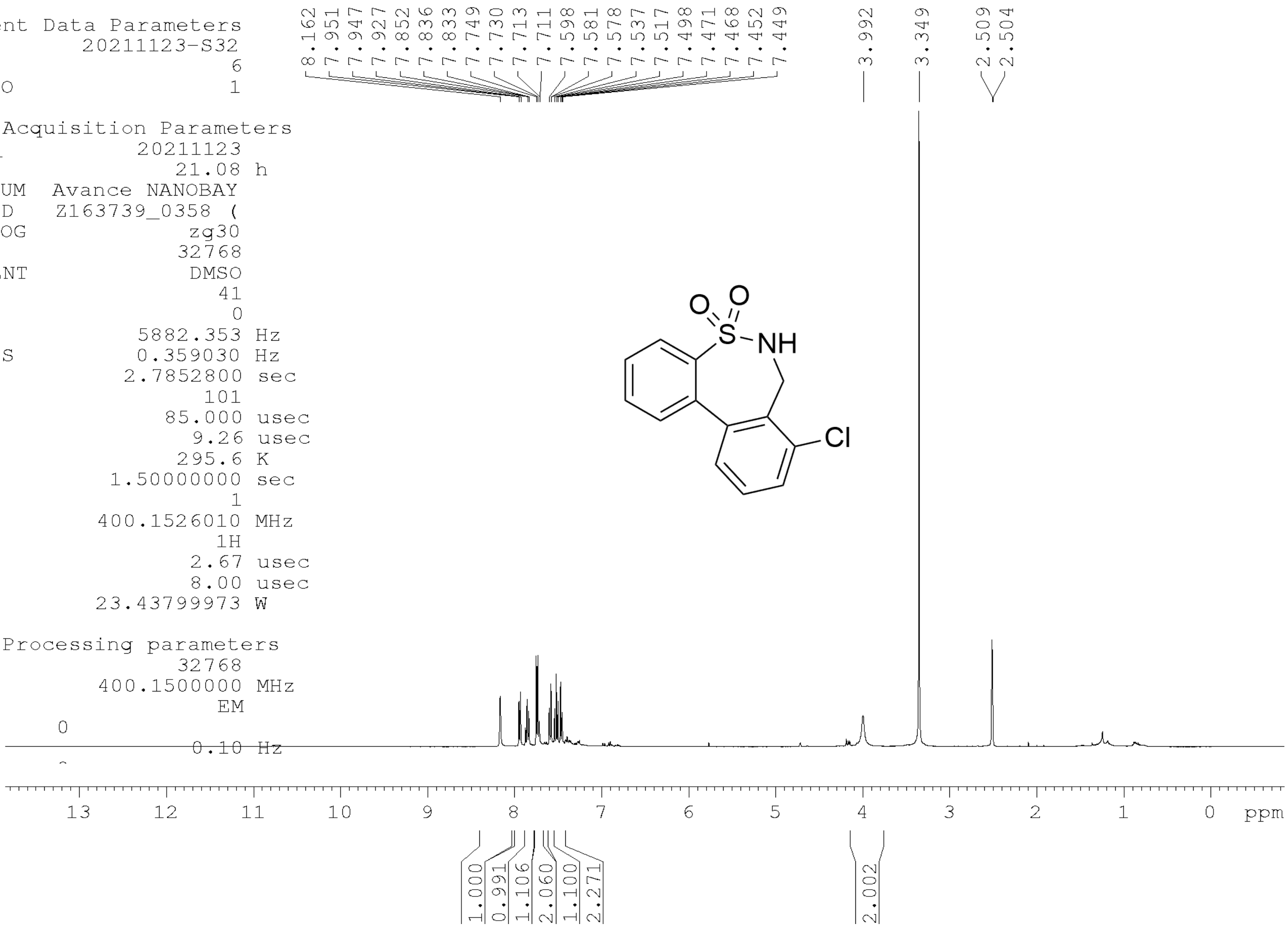

^{13}C NMR of 2n

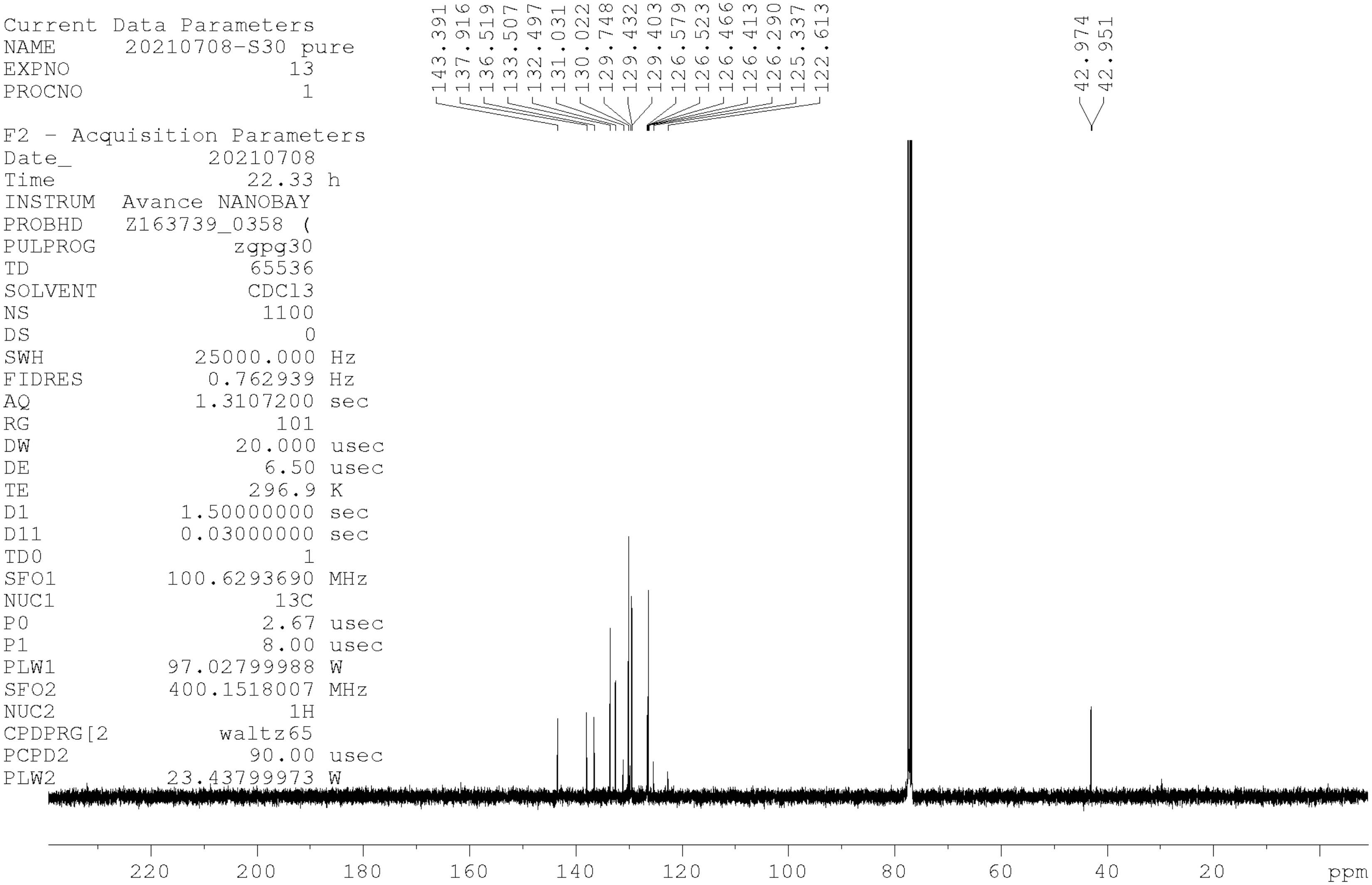
Current Data Parameters
NAME 20210708-S30 pure
EXPNO 13
PROCNO 1
F2 - Acquisition Parameters
Date_ 20210708
Time 22.33 h
INSTRUM Avance NANOBAY
PROBHD Z163739_0358 (
PULPROG zgpg30
TD 65536
SOLVENT CDCl3
NS 1100
DS 0
SWH 25000.000 Hz
FIDRES 0.762939 Hz
AQ 1.3107200 sec
RG 101
DW 20.000 usec
DE 6.50 usec
TE 296.9 K
D1 1.50000000 sec
D11 0.03000000 sec
TD0 1
SFO1 100.6293690 MHz
NUC1 13C
P0 2.67 usec
P1 8.00 usec
PLW1 97.02799988 W
SFO2 400.1518007 MHz
NUC2 1H
CPDPRG[2 waltz65
PCPD2 90.00 usec
PLW2 23.43799973 W
143.391
137.916
136.519
133.507
132.497
131.031
130.022
129.748
129.432
129.403
126.579
126.523
126.466
126.413
126.290
125.337
122.613
42.974
42.951
220
200
180
160
140
120
100
80
60
40
20
ppm

Current Data Parameters
NAME 20211124-S32 dmso
EXPNO 13
PROCNO 1

F2 - Acquisition Parameters
Date_ 20211125
Time 5.51 h
INSTRUM Avance NANOBAY
PROBHD Z163739_0358 (
PULPROG zgpg30
TD 65536
SOLVENT DMSO
NS 10000
DS 0
SWH 25000.000 Hz
FIDRES 0.762939 Hz
AQ 1.3107200 sec
RG 101
DW 20.000 usec
DE 6.50 usec
TE 295.6 K
D1 1.50000000 sec
D11 0.03000000 sec
TD0 1
SFO1 100.6293690 MHz
NUC1 13C
P0 2.67 usec
P1 8.00 usec
PLW1 97.02799988 W
SFO2 400.1518007 MHz
NUC2 1H
CPDPRG[2 waltz65
PCPD2 90.00 usec
PLW2 23.43799973 W
PLW12 0.18519001 W
PLW13 0.09314800 W

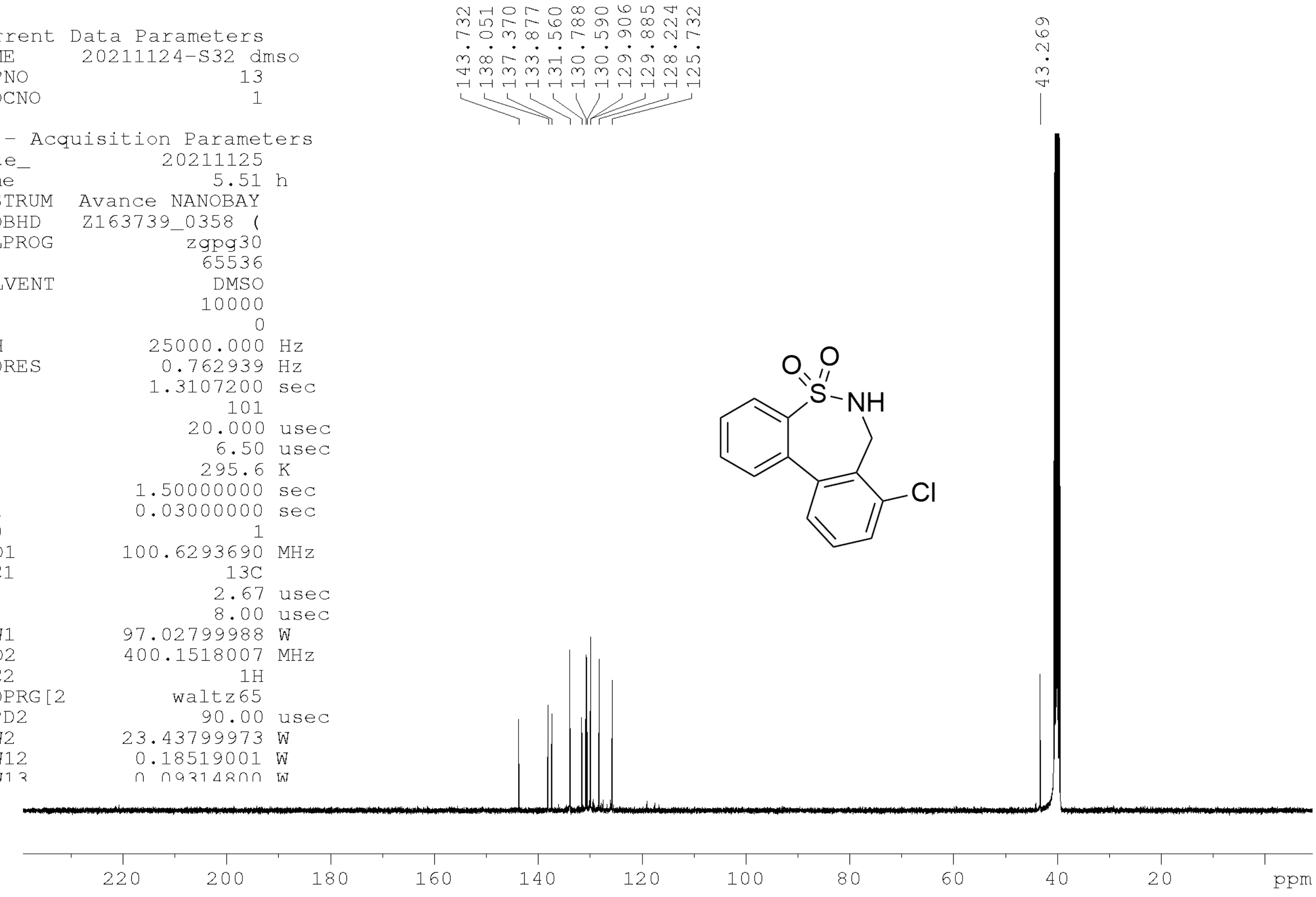

^{1}H NMR of 2o

```
Current Data Parameters
NAME        20210923-S-25
EXPNO                   9
PROCNO                  1

F2 - Acquisition Parameters
Date_            20210923
Time                21.23 h
INSTRUM   Avance NANOBAY
PROBHD    Z163739_0358 (
PULPROG              zg30
TD                  32768
SOLVENT             CDCl3
NS                     41
DS                      0
SWH              5882.353 Hz
FIDRES           0.359030 Hz
AQ              2.7852800 se
RG                    101
DW                 85.000 us
DE                   9.26 us
TE                  296.7 K
D1             1.50000000 se
TD0                     1
SFO1          400.1526010 MH
NUC1                   1H
P0                   2.67 us
P1                   8.00 us
PLW1          23.43799973 W

F2 - Processing parameters
SI                  32768
SF            400.1500000 MH
WDW                    EM
```

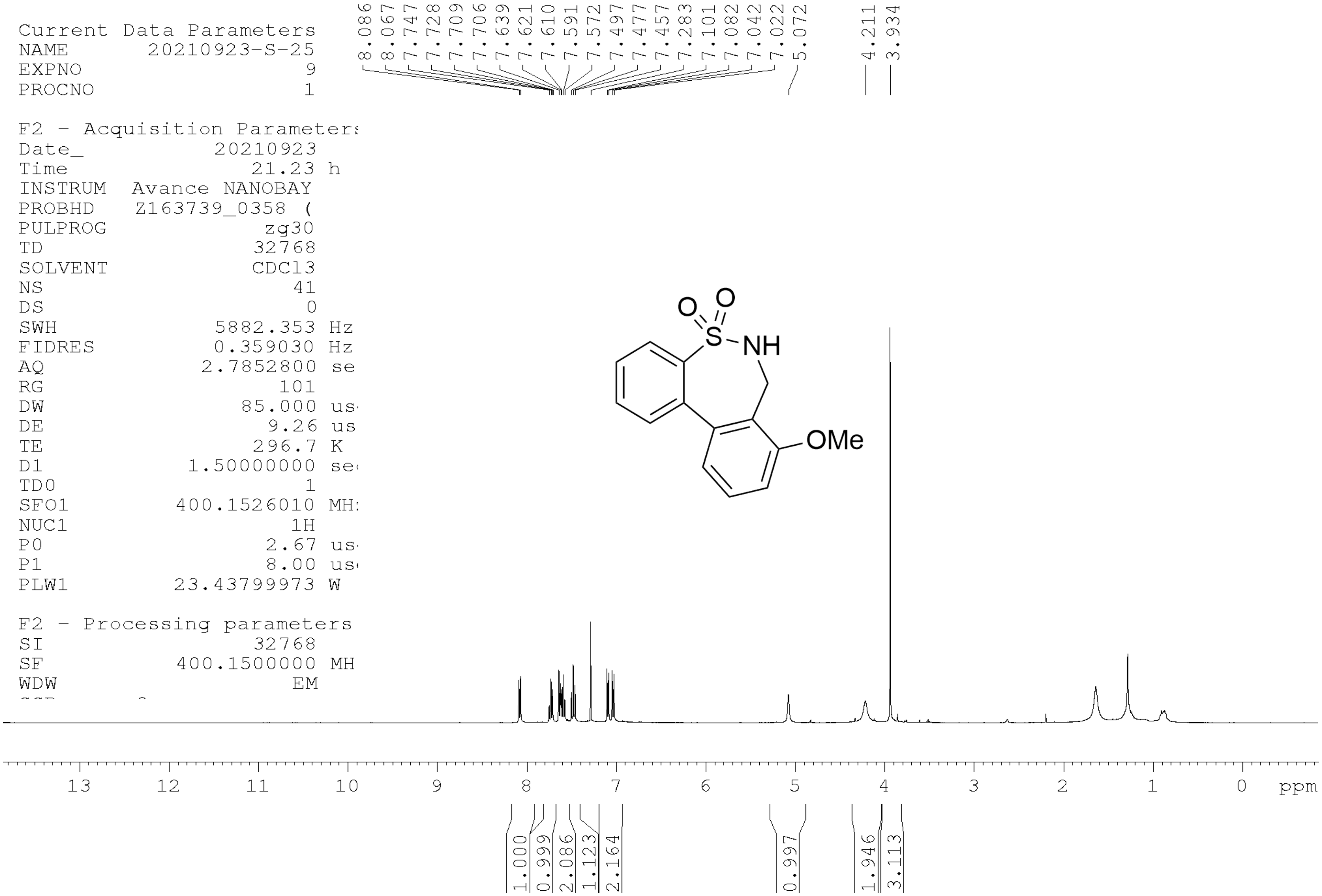

^{13}C NMR of 2o

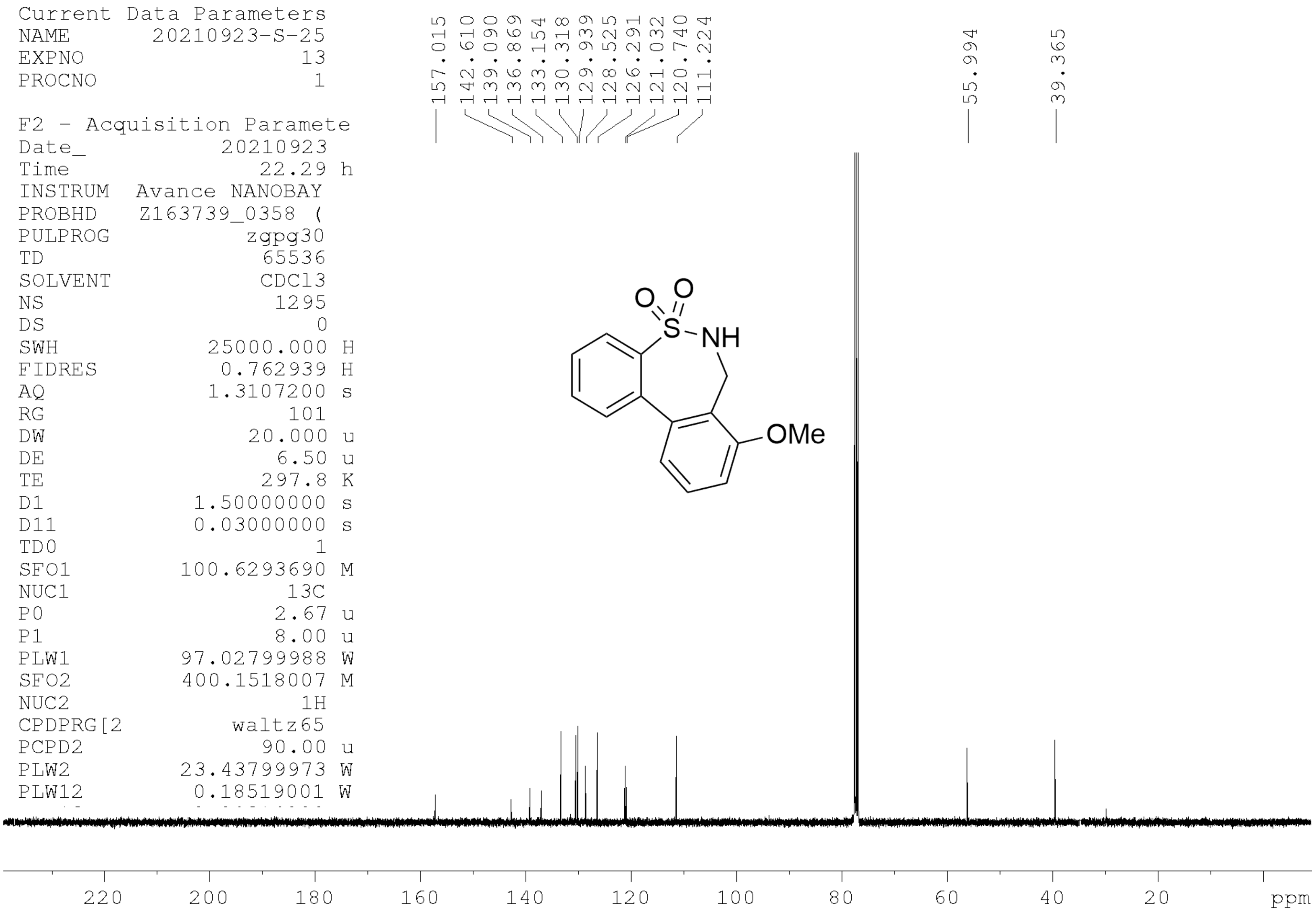
Current Data Parameters
NAME 20210923-S-25
EXPNO 13
PROCNO 1
F2 - Acquisition Paramete
Date_ 20210923
Time 22.29 h
INSTRUM Avance NANOBAY
PROBHD Z163739_0358 (
PULPROG zgpg30
TD 65536
SOLVENT CDCl3
NS 1295
DS 0
SWH 25000.000 H
FIDRES 0.762939 H
AQ 1.3107200 s
RG 101
DW 20.000 u
DE 6.50 u
TE 297.8 K
D1 1.50000000 s
D11 0.03000000 s
TD0 1
SFO1 100.6293690 M
NUC1 13C
P0 2.67 u
P1 8.00 u
PLW1 97.02799988 W
SFO2 400.1518007 M
NUC2 1H
CPDPRG[2 waltz65
PCPD2 90.00 u
PLW2 23.43799973 W
PLW12 0.18519001 W
157.015
142.610
139.090
136.869
133.154
130.318
129.939
128.525
126.291
121.032
120.740
111.224
55.994
39.365
O
O
S
NH
OMe
220
200
180
160
140
120
100
80
60
40
20
ppm

^{1}H NMR of 2p

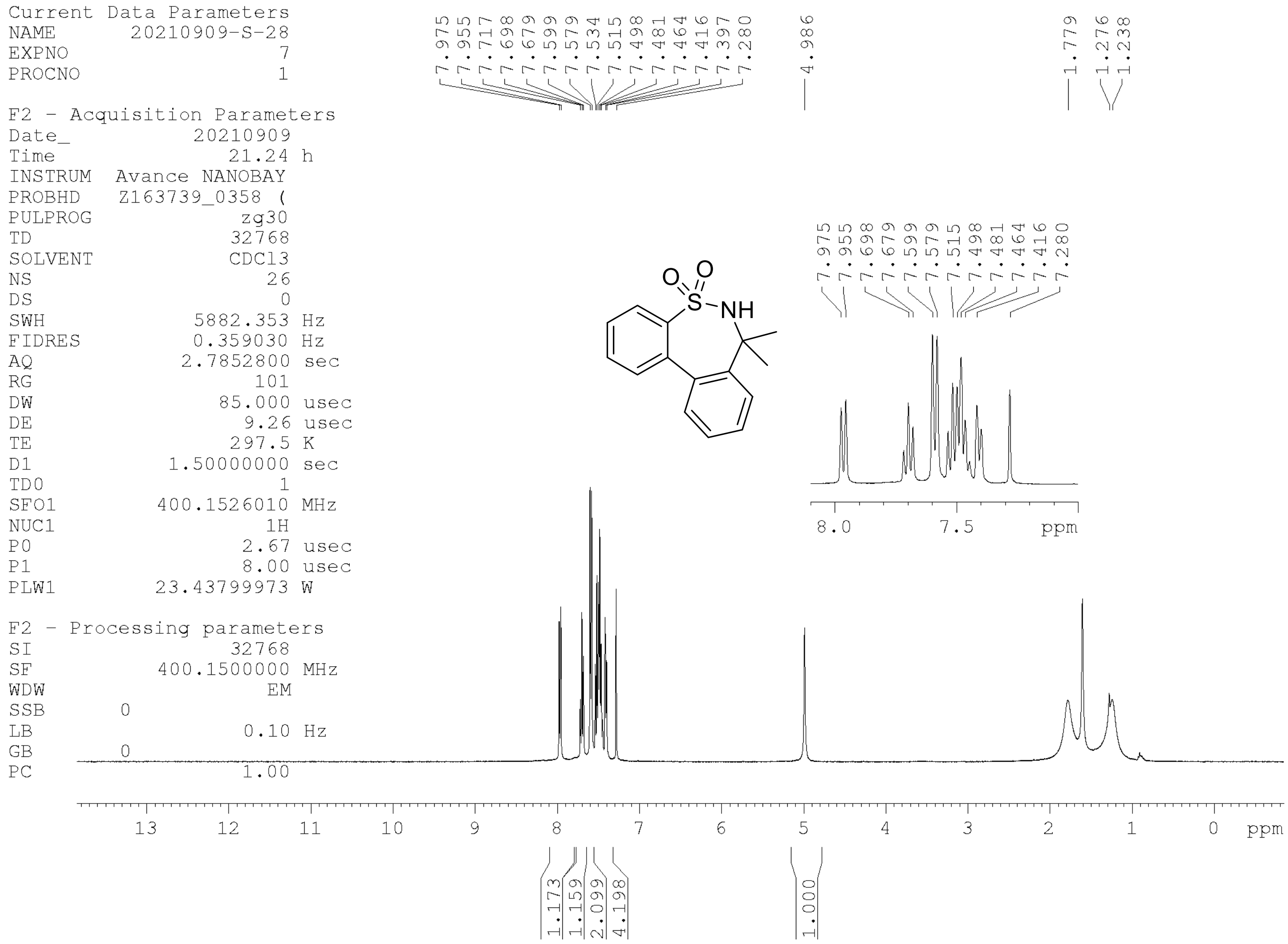
Current Data Parameters
NAME 20210909-S-28
EXPNO 7
PROCNO 1
F2 - Acquisition Parameters
Date_ 20210909
Time 21.24 h
INSTRUM Avance NANOBAY
PROBHD Z163739_0358 (
PULPROG zg30
TD 32768
SOLVENT CDCl3
NS 26
DS 0
SWH 5882.353 Hz
FIDRES 0.359030 Hz
AQ 2.7852800 sec
RG 101
DW 85.000 usec
DE 9.26 usec
TE 297.5 K
D1 1.50000000 sec
TD0 1
SFO1 400.1526010 MHz
NUC1 1H
P0 2.67 usec
P1 8.00 usec
PLW1 23.43799973 W
F2 - Processing parameters
SI 32768
SF 400.1500000 MHz
WDW EM
SSB 0
LB 0.10 Hz
GB 0
PC 1.00
7.975
7.955
7.717
7.698
7.679
7.599
7.579
7.534
7.515
7.498
7.481
7.464
7.416
7.397
7.280
4.986
1.779
1.276
1.238
O
O
S
NH
8.0
7.5
ppm
13
12
11
10
9
8
7
6
5
4
3
2
1
0
ppm
1.173
1.159
2.099
4.198
1.000

^{13}C NMR of 2p

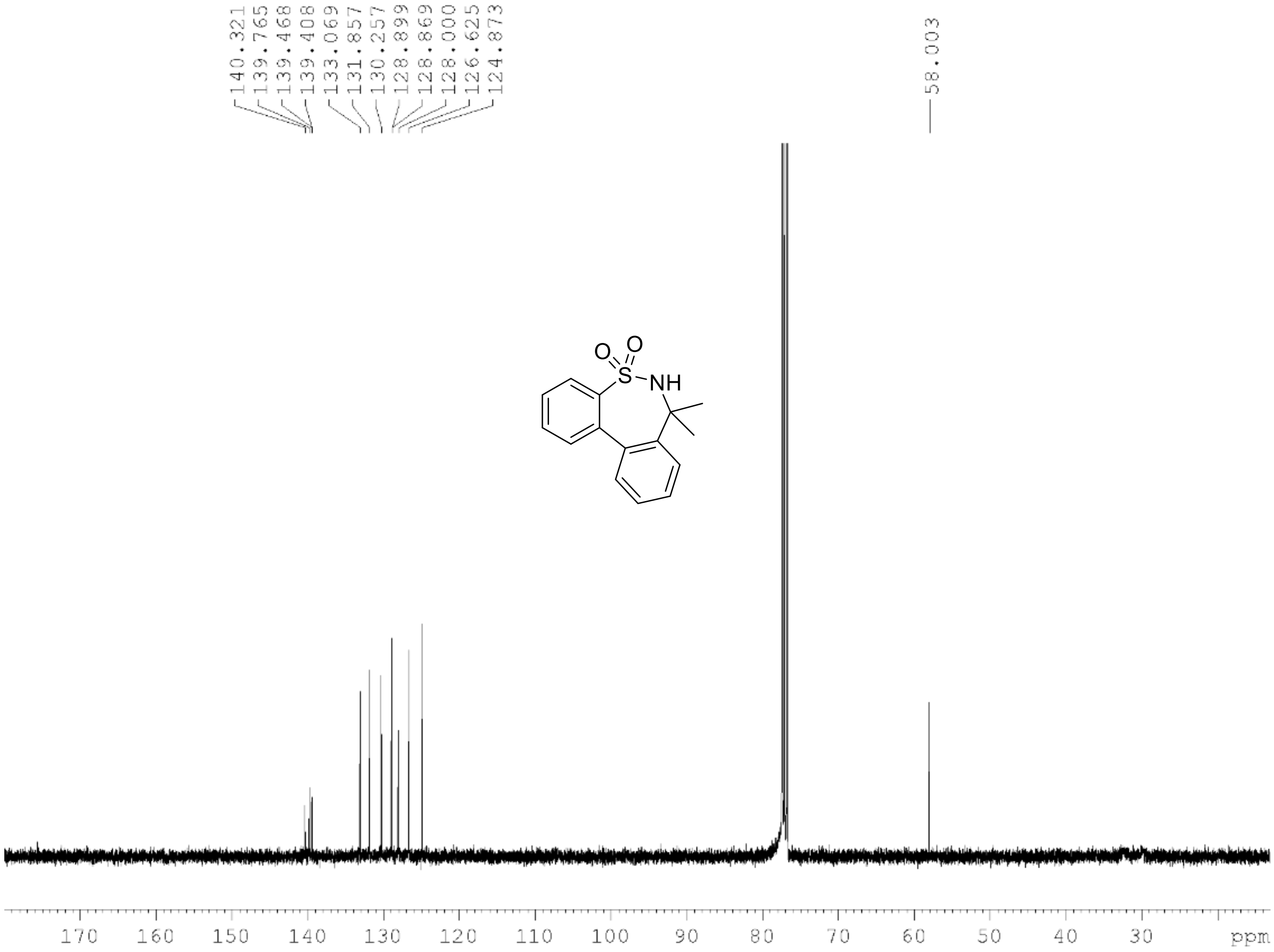
140.321
139.765
139.468
139.408
133.069
131.857
130.257
128.899
128.869
128.000
126.625
124.873
58.003
O
O
S
NH
170
160
150
140
130
120
110
100
90
80
70
60
50
40
30
ppm

^{1}H NMR of 2q

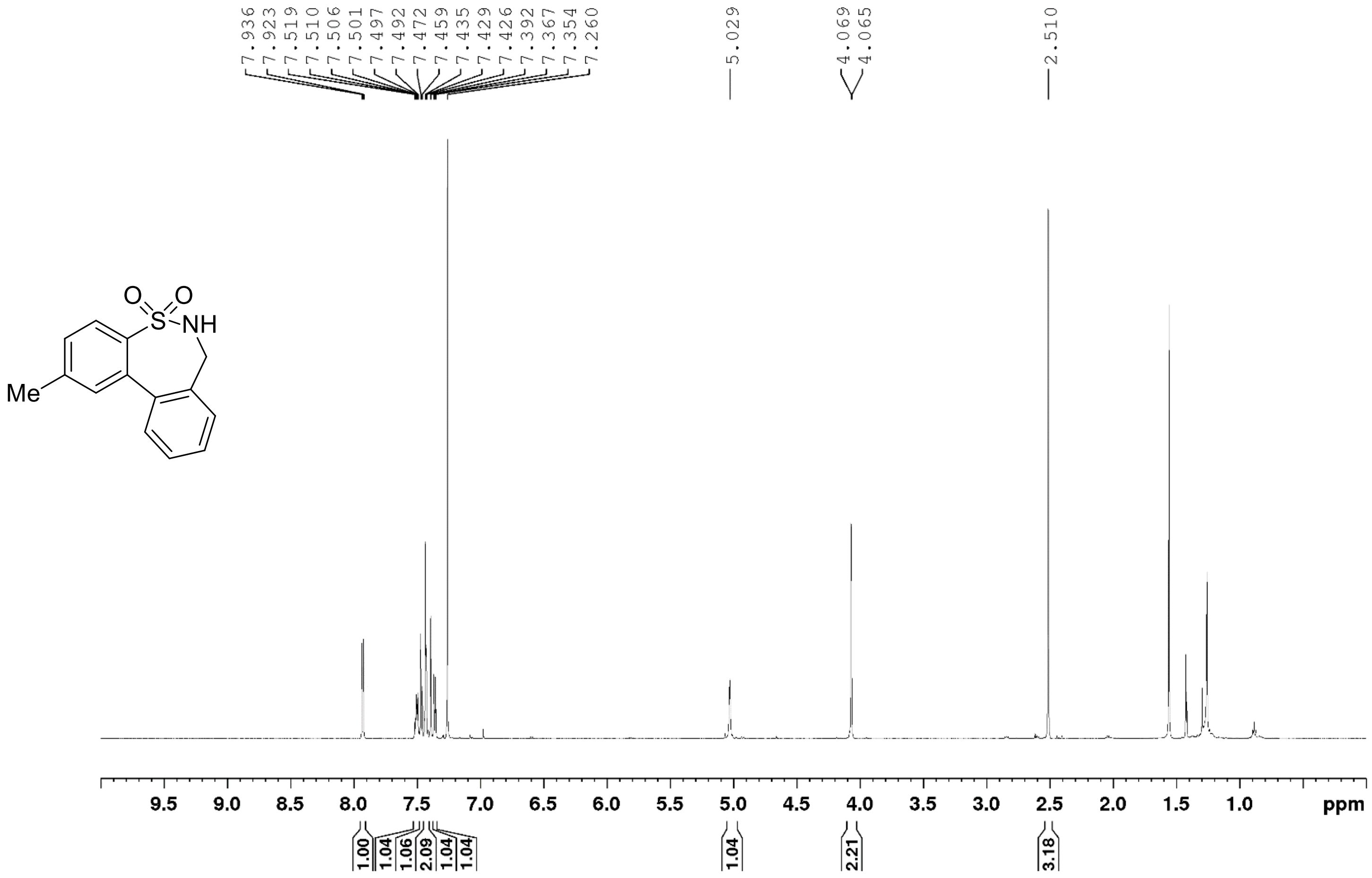

7.936
7.923
7.519
7.510
7.506
7.501
7.497
7.492
7.472
7.459
7.435
7.429
7.426
7.392
7.367
7.354
7.260
5.029
4.069
4.065
2.510
Me
O
O
S
NH
9.5
9.0
8.5
8.0
7.5
7.0
6.5
6.0
5.5
5.0
4.5
4.0
3.5
3.0
2.5
2.0
1.5
1.0
ppm
1.00
1.04
1.06
2.09
1.04
1.04
1.04
2.21
3.18

^{13}C NMR of 2q

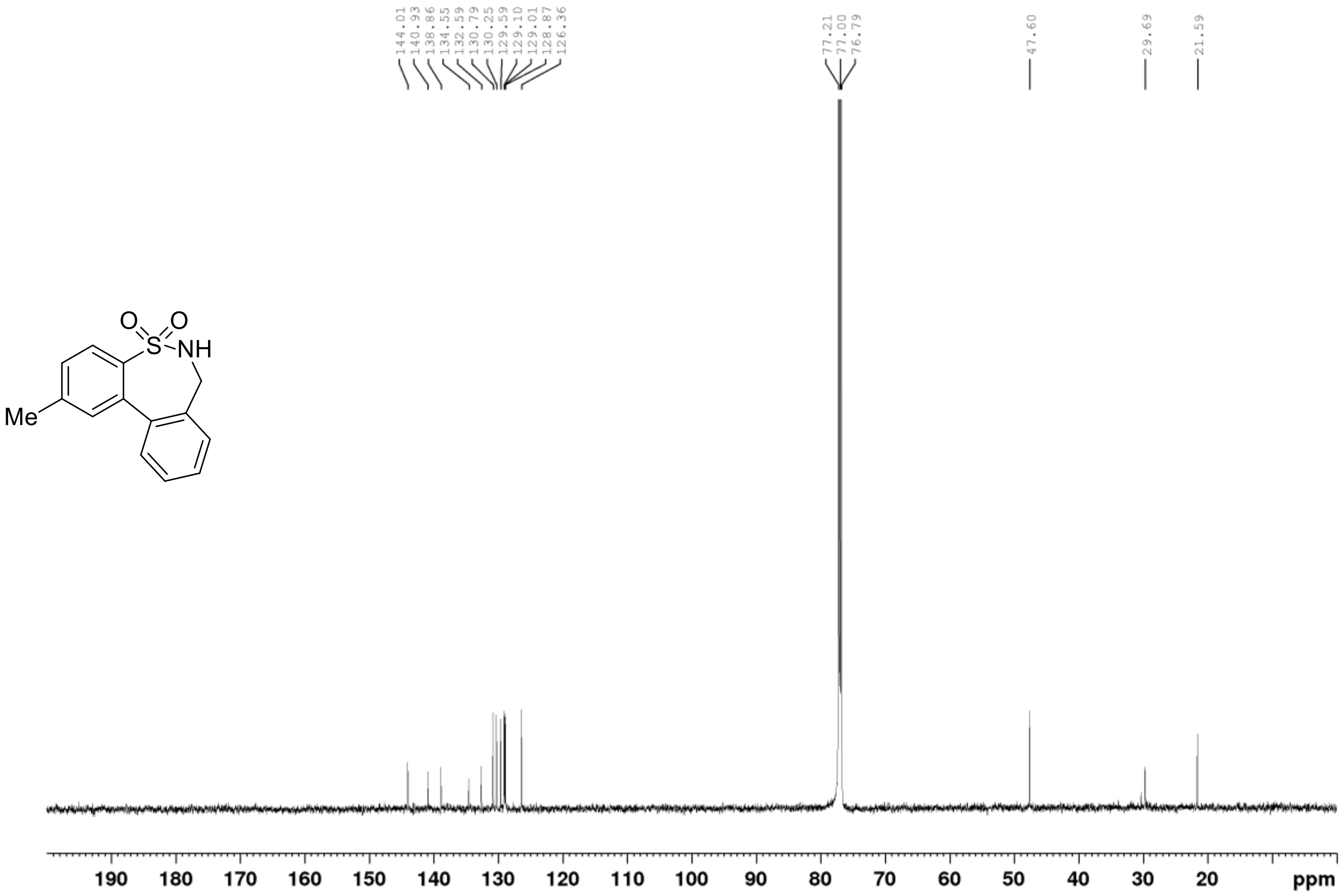

144.01
140.93
138.86
134.55
132.59
130.79
130.25
129.59
129.59
129.10
129.01
128.87
126.36
77.21
77.00
76.79
47.60
29.69
21.59
190
180
170
160
150
140
130
120
110
100
90
80
70
60
50
40
30
20
ppm

^{1}H NMR of 2r

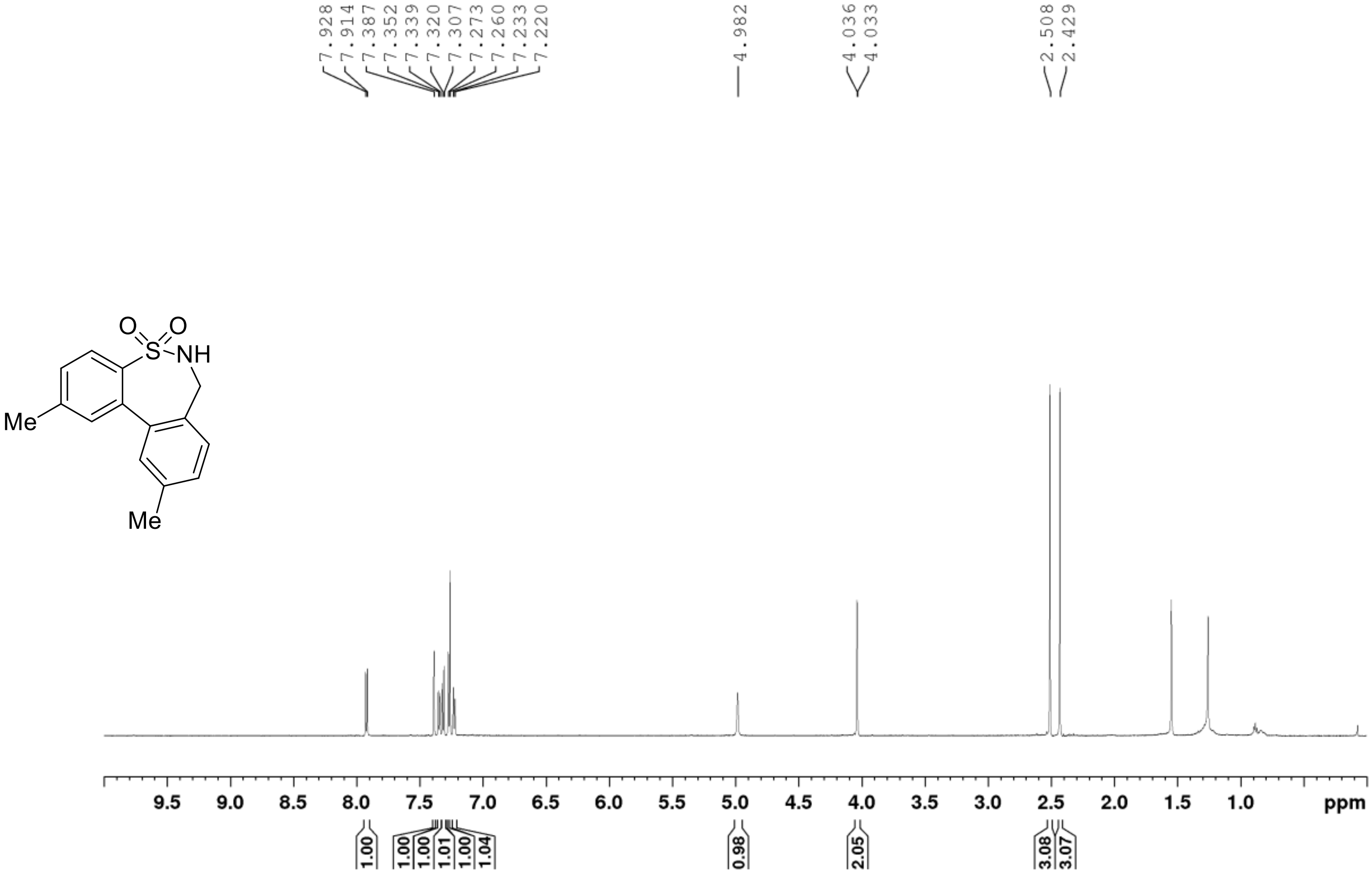

O
O
S
NH
Me
Me
7.928
7.914
7.387
7.352
7.339
7.320
7.307
7.273
7.260
7.233
7.220
4.982
4.036
4.033
2.508
2.429
9.5
9.0
8.5
8.0
7.5
7.0
6.5
6.0
5.5
5.0
4.5
4.0
3.5
3.0
2.5
2.0
1.5
1.0
ppm
1.00
1.00
1.00
1.01
1.00
1.04
0.98
2.05
3.08
3.07

^{13}C NMR of 2r

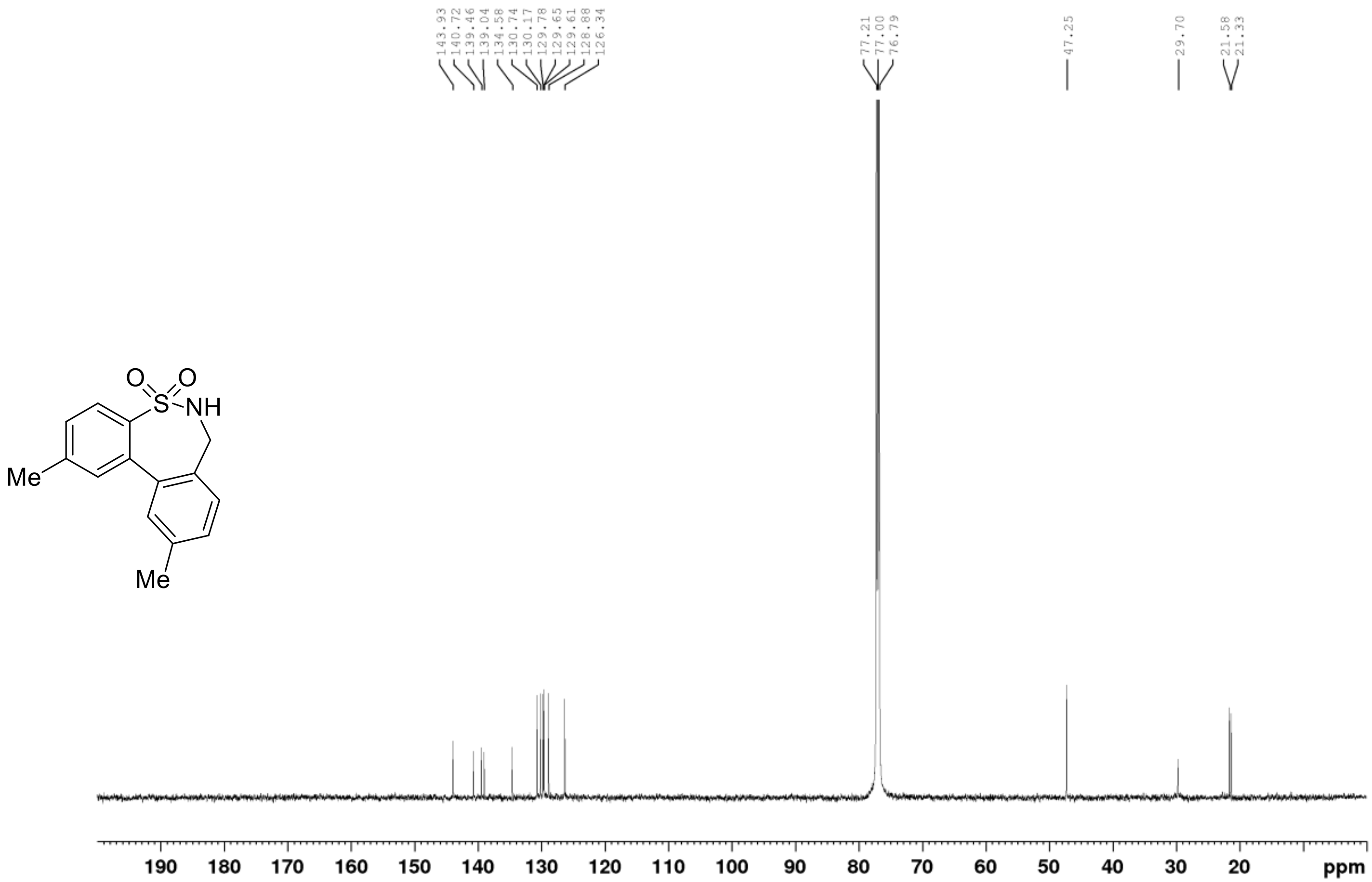

143.93
140.72
139.46
139.04
134.58
130.74
130.17
129.78
129.65
129.61
128.88
126.34
77.21
77.00
76.79
47.25
29.70
21.58
21.33
190
180
170
160
150
140
130
120
110
100
90
80
70
60
50
40
30
20
ppm
O
O
S
NH
Me
Me

^{1}H NMR of 2s

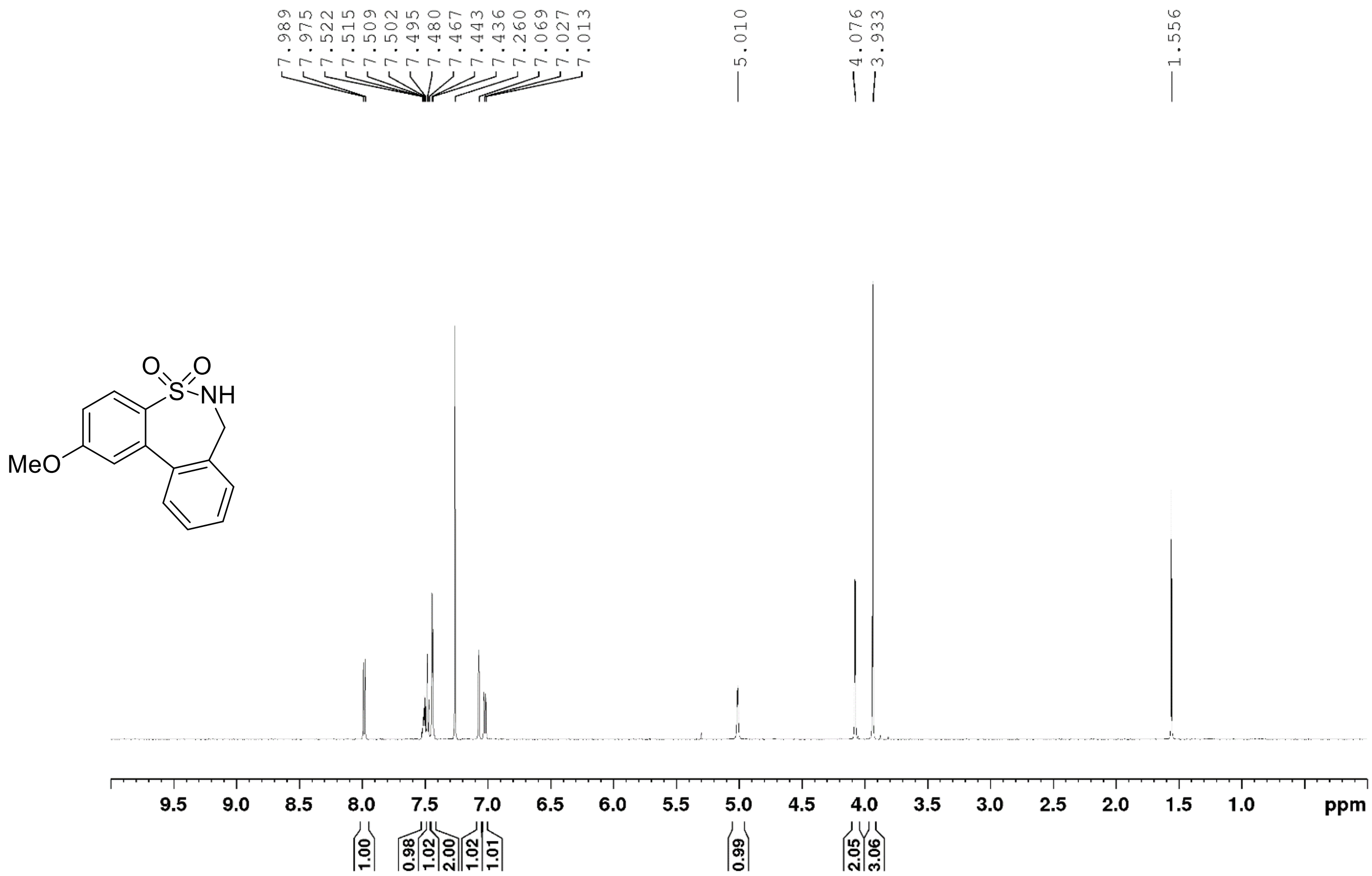

7.989
7.975
7.522
7.515
7.509
7.502
7.495
7.480
7.467
7.443
7.436
7.260
7.069
7.027
7.013
5.010
4.076
3.933
1.556
MeO
O
O
S
NH
9.5
9.0
8.5
8.0
7.5
7.0
6.5
6.0
5.5
5.0
4.5
4.0
3.5
3.0
2.5
2.0
1.5
1.0
ppm
1.00
0.98
1.02
2.00
1.02
1.01
0.99
2.05
3.06

^{13}C NMR of 2s

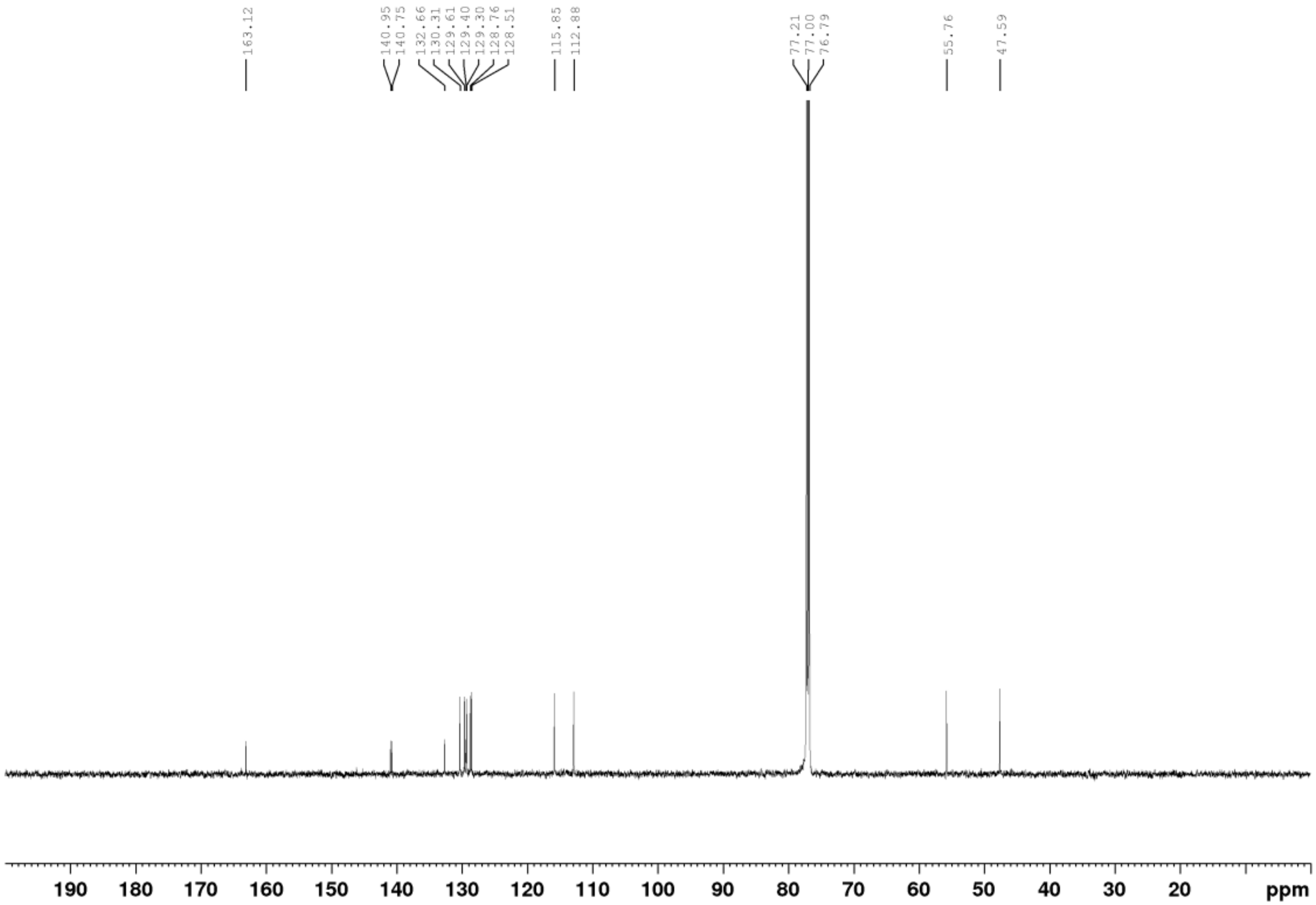
163.12
140.95
140.75
132.66
130.31
129.61
129.40
129.30
128.76
128.51
115.85
112.88
77.21
77.00
76.79
55.76
47.59
190
180
170
160
150
140
130
120
110
100
90
80
70
60
50
40
30
20
ppm

^{1}H NMR of 2t

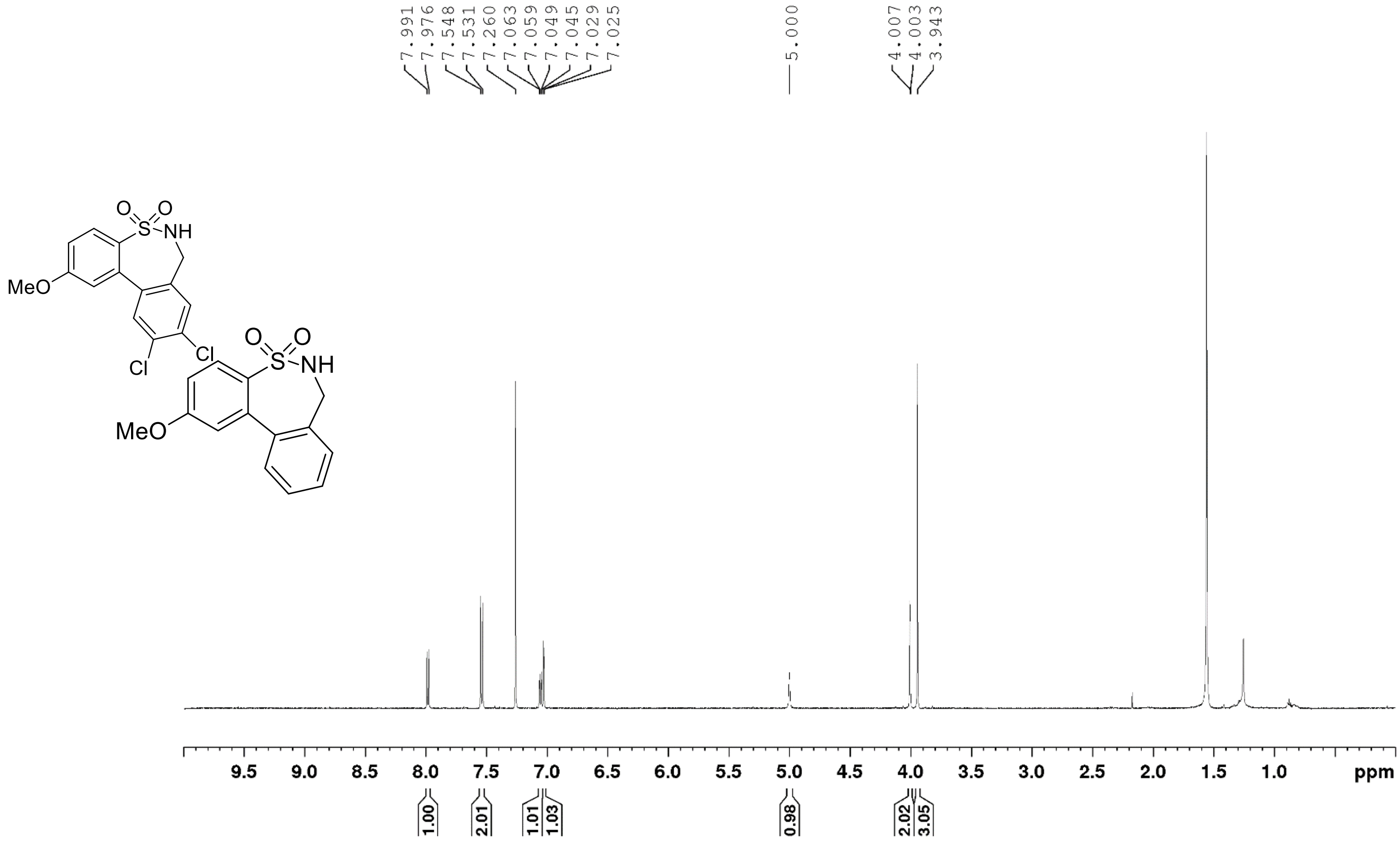

7.991
7.976
7.548
7.531
7.260
7.063
7.059
7.049
7.045
7.029
7.025
5.000
4.007
4.003
3.943
MeO
O
S
NH
Cl
9.5
9.0
8.5
8.0
7.5
7.0
6.5
6.0
5.5
5.0
4.5
4.0
3.5
3.0
2.5
2.0
1.5
1.0
ppm
1.00
2.01
1.01
1.03
0.98
2.02
3.05

^{13}C NMR of 2t

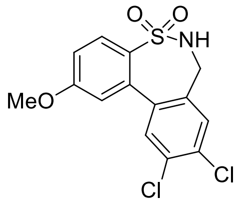
MeO
S
NH
O
O
Cl
Cl

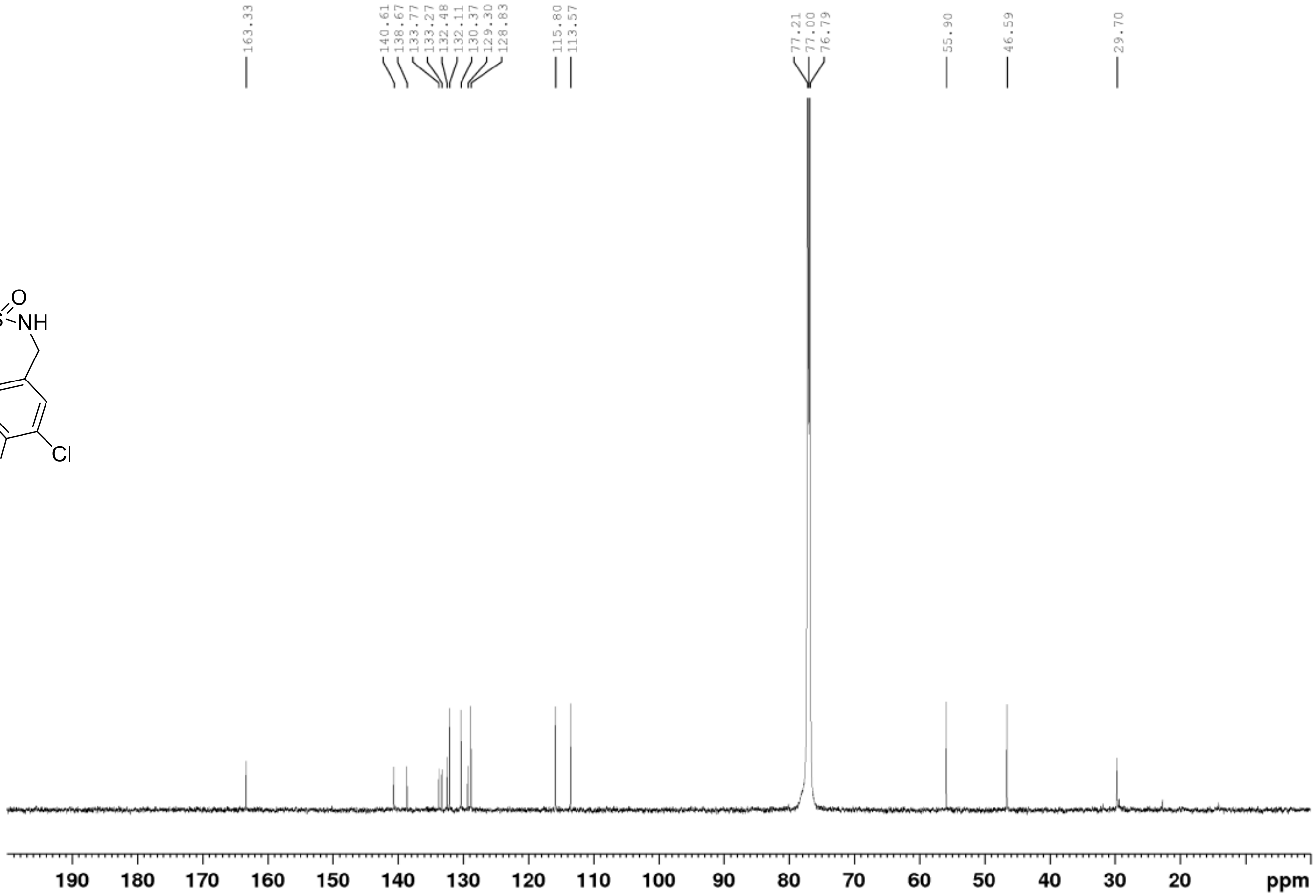
163.33
140.61
138.67
133.77
133.27
132.48
132.11
130.37
129.30
128.83
115.80
113.57
77.21
77.00
76.79
55.90
46.59
29.70
190
180
170
160
150
140
130
120
110
100
90
80
70
60
50
40
30
20
ppm

^{1}H NMR of 2u

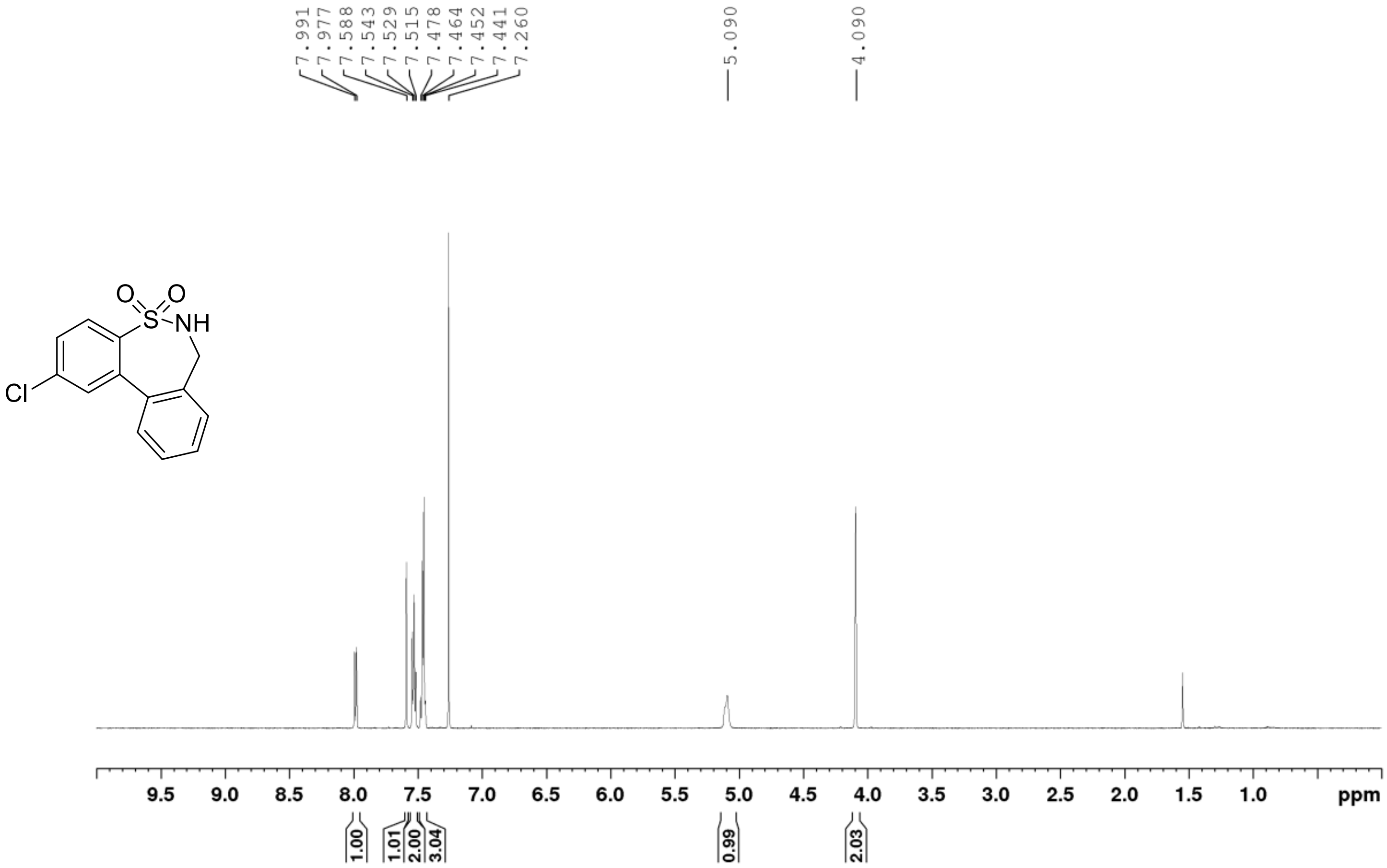

7.991
7.977
7.588
7.543
7.529
7.515
7.478
7.464
7.452
7.441
7.260
5.090
4.090
O
O
S
NH
Cl
9.5 9.0 8.5 8.0 7.5 7.0 6.5 6.0 5.5 5.0 4.5 4.0 3.5 3.0 2.5 2.0 1.5 1.0 ppm
1.00
1.01
2.00
3.04
0.99
2.03

^{13}C NMR of 2u

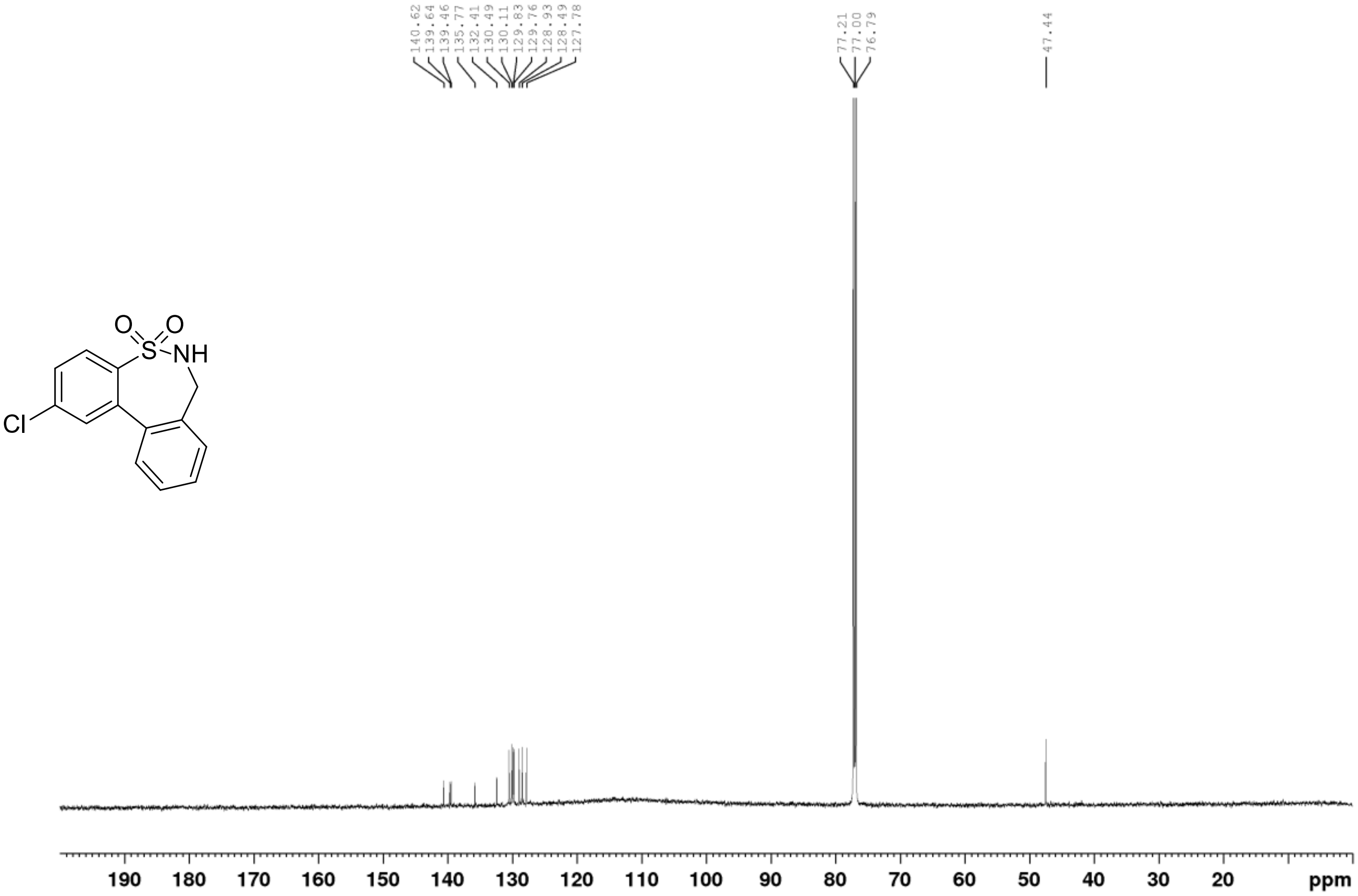

O
O
S
NH
Cl
140.62
139.64
139.46
135.77
132.41
130.49
130.11
129.83
129.76
128.93
128.49
127.78
77.21
77.00
76.79
47.44
190
180
170
160
150
140
130
120
110
100
90
80
70
60
50
40
30
20
ppm

^{1}H NMR of 2v

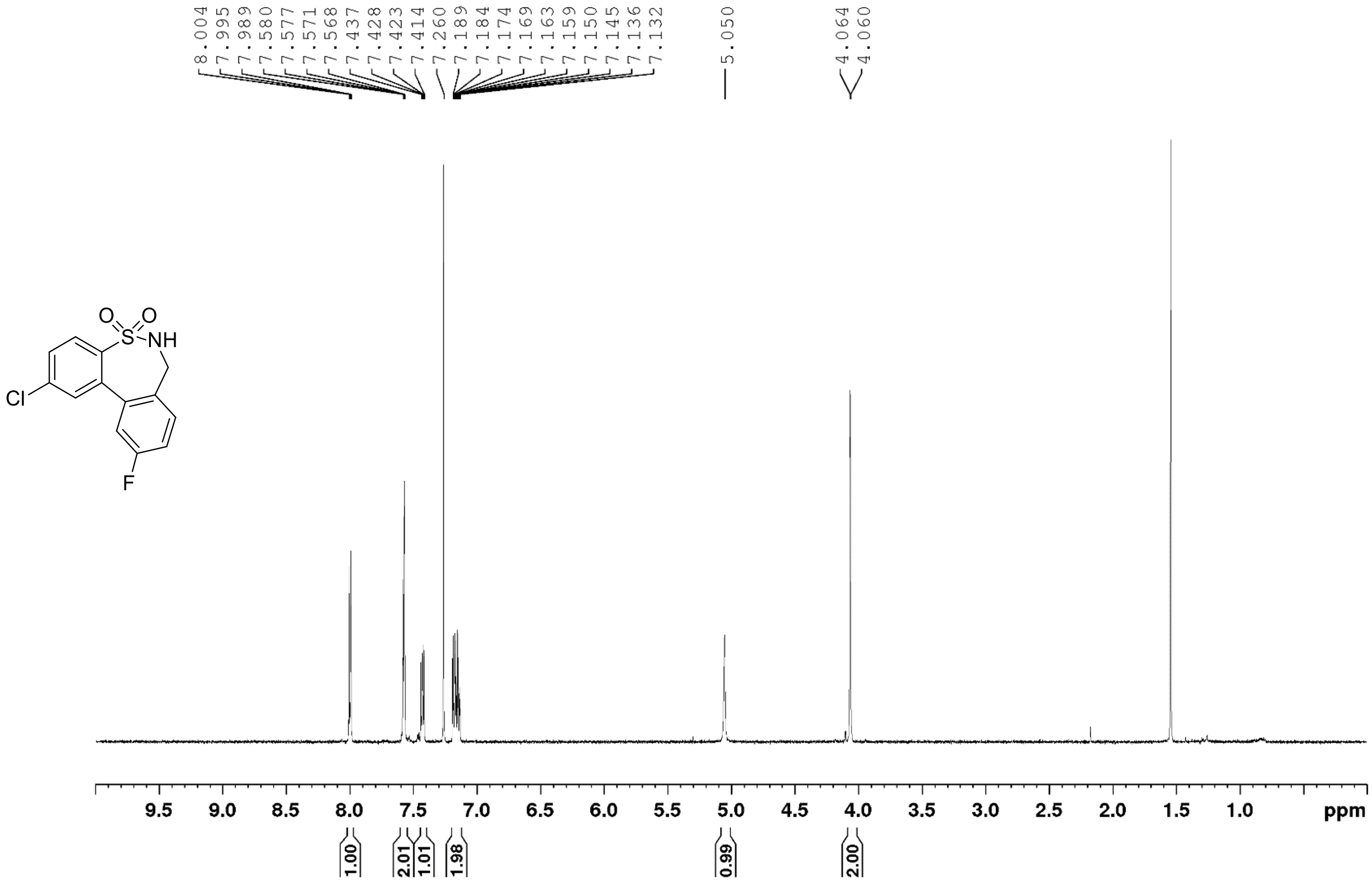

8.004
7.995
7.989
7.580
7.577
7.571
7.568
7.437
7.428
7.423
7.414
7.260
7.189
7.184
7.174
7.169
7.163
7.159
7.150
7.145
7.136
7.132
5.050
4.064
4.060
O
O
S
NH
Cl
F
9.5
9.0
8.5
8.0
7.5
7.0
6.5
6.0
5.5
5.0
4.5
4.0
3.5
3.0
2.5
2.0
1.5
1.0
ppm
1.00
2.01
1.01
1.98
0.99
2.00

^{13}C NMR of 2v

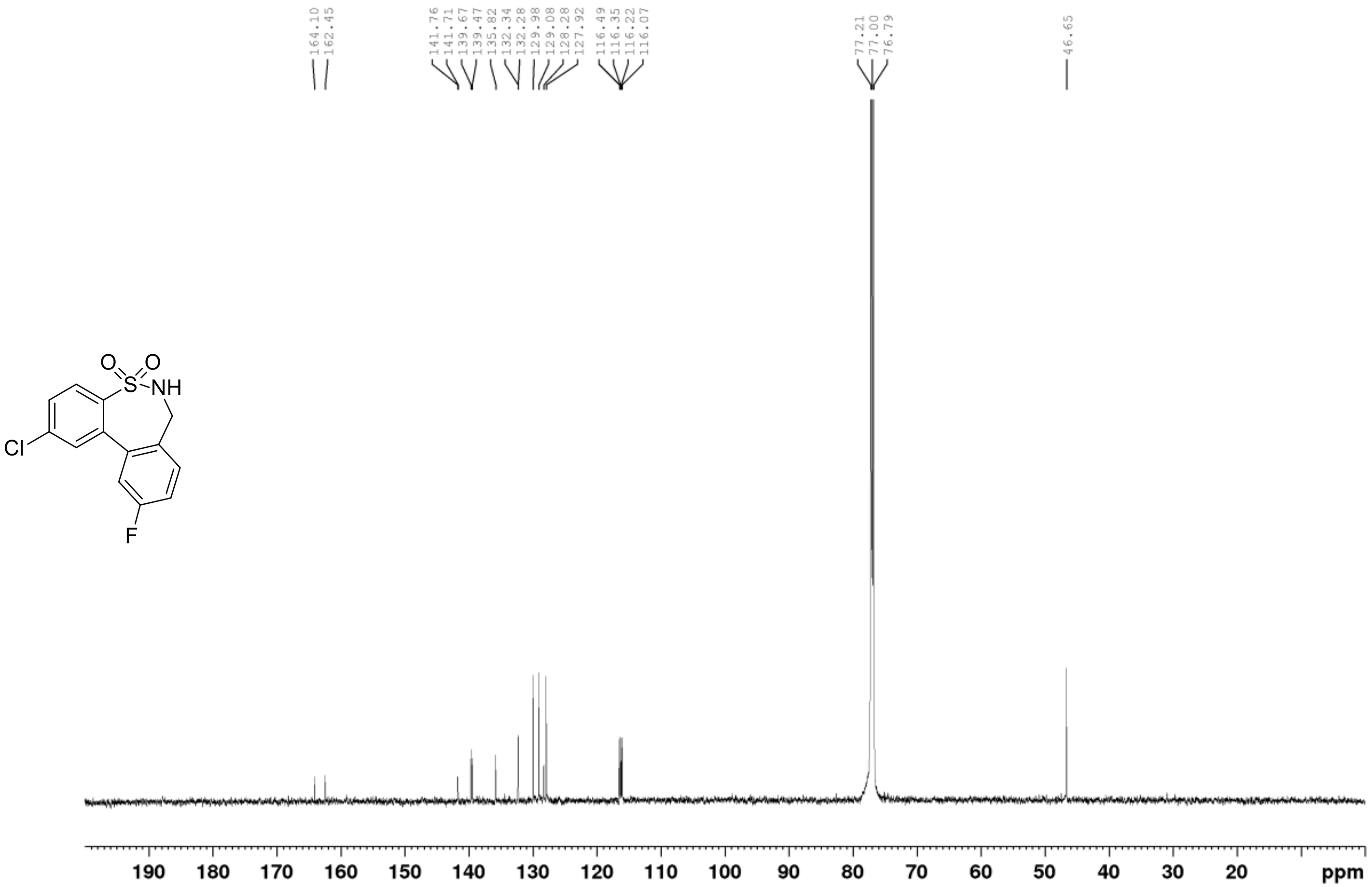
O
O
S
NH
Cl
F
164.10
162.45
141.76
141.71
139.67
139.47
135.82
132.34
132.28
129.98
129.08
128.28
127.92
116.49
116.35
116.22
116.07
77.21
77.00
76.79
46.65
190
180
170
160
150
140
130
120
110
100
90
80
70
60
50
40
30
20
ppm

^{1}H NMR of 2w

```
Current Data Parameters
NAME        20210928 s-18
EXPNO                   1
PROCNO                  1

F2 - Acquisition Parameters
Date_            20210928
Time                14.24
INSTRUM             spect
PROBHD   5 mm BBO BB-1H
PULPROG              zg30
TD                  32768
SOLVENT             CDCl3
NS                     61
DS                      0
SWH              6009.615 Hz
FIDRES           0.183399 Hz
AQ              2.7262976 sec
RG                    362
DW                 83.200 usec
DE                   6.50 usec
TE                  294.8 K
D1             1.50000000 sec
TD0                     1

======== CHANNEL f1 ========
NUC1                   1H
P1                  14.00 usec
PL1                 -1.00 dB
PL1W           7.55784369 W
SFO1          400.1326010 MHz

F2 - Processing parameters
SI                  32768
SF            400.1300087 MHz
WDW                    EM
```

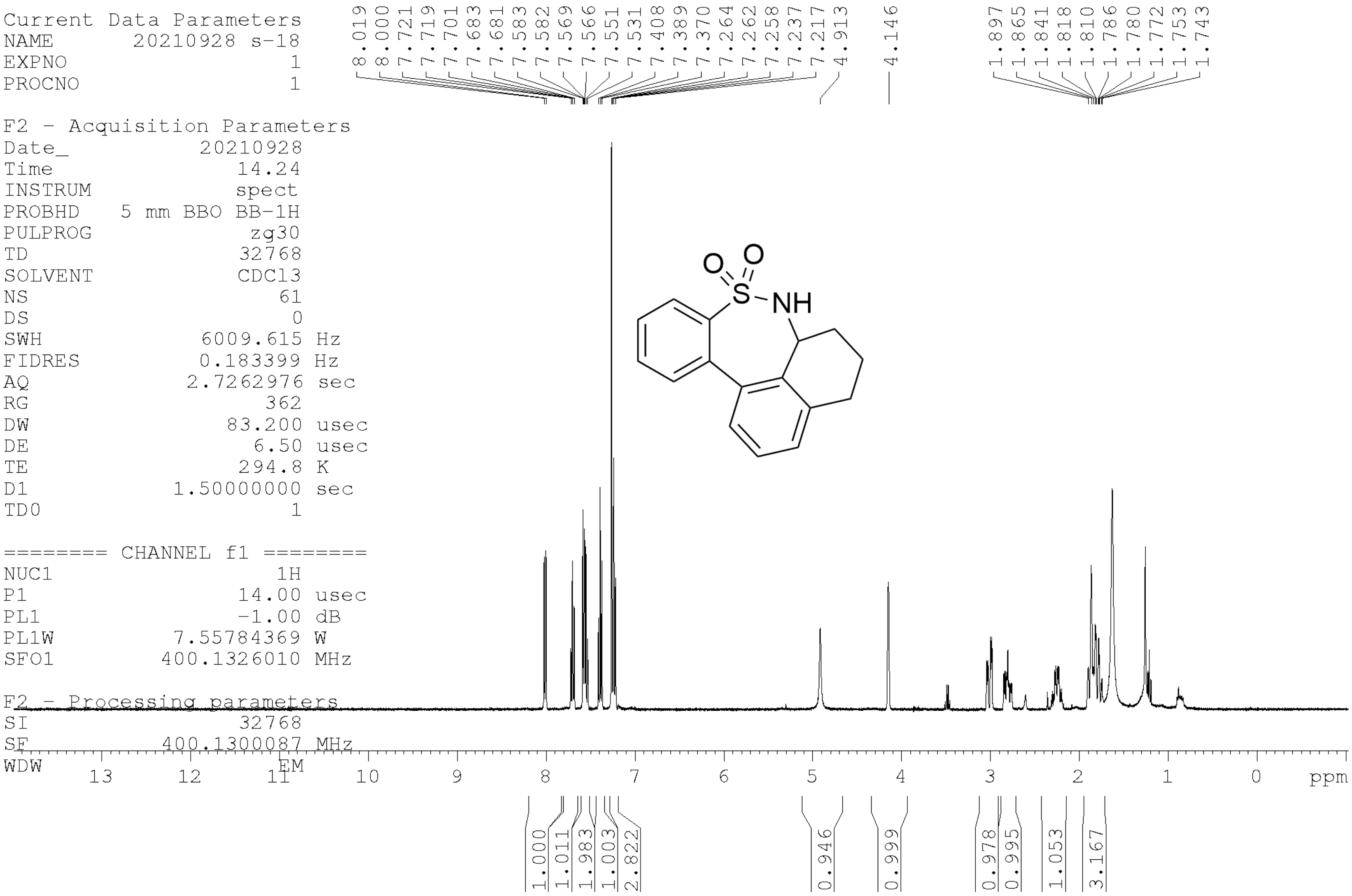

^{13}C NMR of 2w

```
Current Data Parameters
NAME     20210611-S18 cyclohexybe
EXPNO                  13
PROCNO                  1

F2 - Acquisition Parameters
Date_            20210611
Time                16.41
INSTRUM             spect
PROBHD   5 mm BBO BB-1H
PULPROG           zgpg30
TD                 65536
SOLVENT            CDCl3
NS                  1200
DS                     0
SWH            25252.525 Hz
FIDRES          0.385323 Hz
AQ             1.2976128 sec
RG                  1620
DW                19.800 usec
DE                  6.50 usec
TE                 295.3 K
D1            1.50000000 sec
D11           0.03000000 sec
TD0                    1

======== CHANNEL f1 ========
NUC1                 13C
P1                 12.40 usec
PL1       0 dB
PL1W         31.64976883 W
SFO1         100.6243400 MHz

======== CHANNEL f2 ========
CPDPRG[2        waltz16
NUC2                  1H
PCPD2              90.00 usec
PL2                -1.00 dB
```

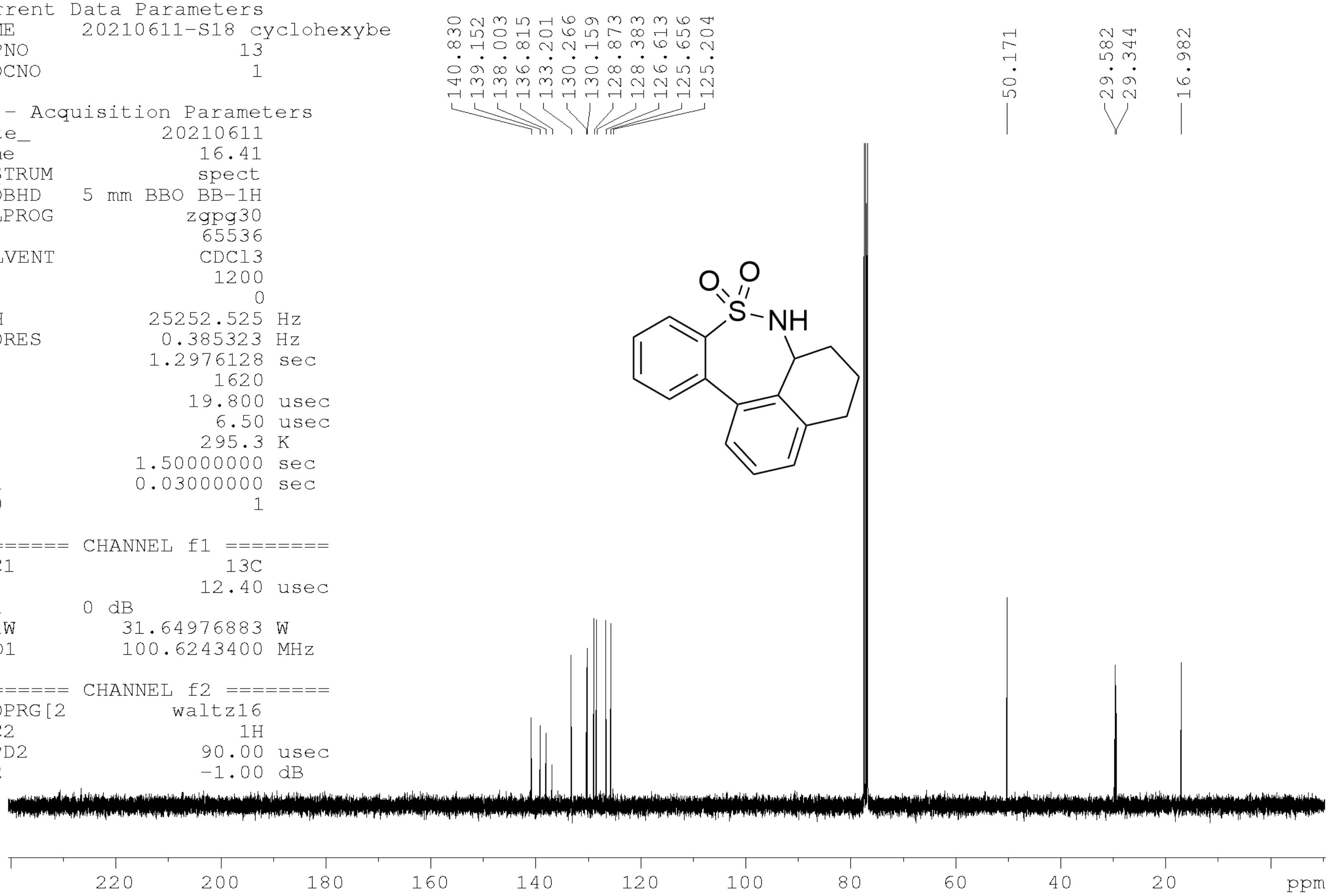

^{1}H NMR of 2x

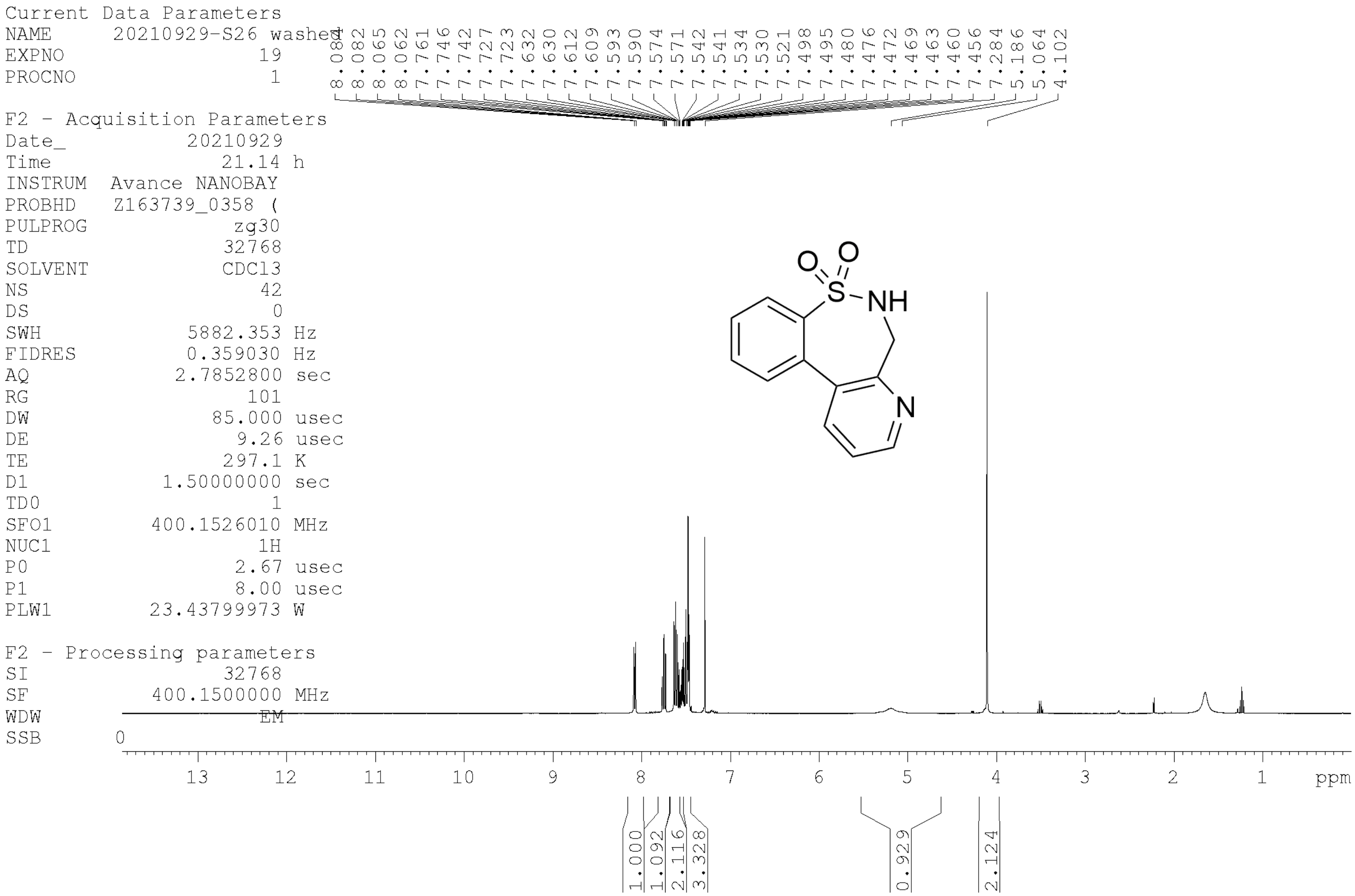
Current Data Parameters
NAME 20210929-S26 washed
EXPNO 19
PROCNO 1
F2 - Acquisition Parameters
Date_ 20210929
Time 21.14 h
INSTRUM Avance NANOBAY
PROBHD Z163739_0358 (
PULPROG zg30
TD 32768
SOLVENT CDCl3
NS 42
DS 0
SWH 5882.353 Hz
FIDRES 0.359030 Hz
AQ 2.7852800 sec
RG 101
DW 85.000 usec
DE 9.26 usec
TE 297.1 K
D1 1.50000000 sec
TD0 1
SFO1 400.1526010 MHz
NUC1 1H
P0 2.67 usec
P1 8.00 usec
PLW1 23.43799973 W
F2 - Processing parameters
SI 32768
SF 400.1500000 MHz
WDW EM
SSB 0
8.084
8.082
8.065
8.062
7.761
7.746
7.742
7.727
7.723
7.632
7.630
7.612
7.609
7.593
7.590
7.574
7.571
7.542
7.541
7.534
7.530
7.521
7.498
7.495
7.480
7.476
7.472
7.469
7.463
7.460
7.456
7.284
5.186
5.064
4.102
O
O
S
NH
N
13
12
11
10
9
8
7
6
5
4
3
2
1
ppm
1.000
1.092
2.116
3.328
0.929
2.124

^{13}C NMR of 2x

Current Data Parameters
NAME 20210928-S26 C13
EXPNO 13
PROCNO 1

F2 - Acquisition Parameters
Date_ 20210928
Time 14.54 h
INSTRUM Avance NANOBAY
PROBHD Z163739_0358 (
PULPROG zgpg30
TD 65536
SOLVENT CDCl3
NS 1651
DS 0
SWH 25000.000 Hz
FIDRES 0.762939 Hz
AQ 1.3107200 sec
RG 101
DW 20.000 usec
DE 6.50 usec
TE 296.8 K
D1 1.50000000 sec
D11 0.03000000 sec
TD0 1
SFO1 100.6293690 MHz
NUC1 13C
P0 2.67 usec
P1 8.00 usec
PLW1 97.02799988 W
SFO2 400.1518007 MHz
NUC2 1H
CPDPRG[2 waltz65
PCPD2 90.00 usec
PLW2 23.43799973 W
PLW12 0.18519001 W

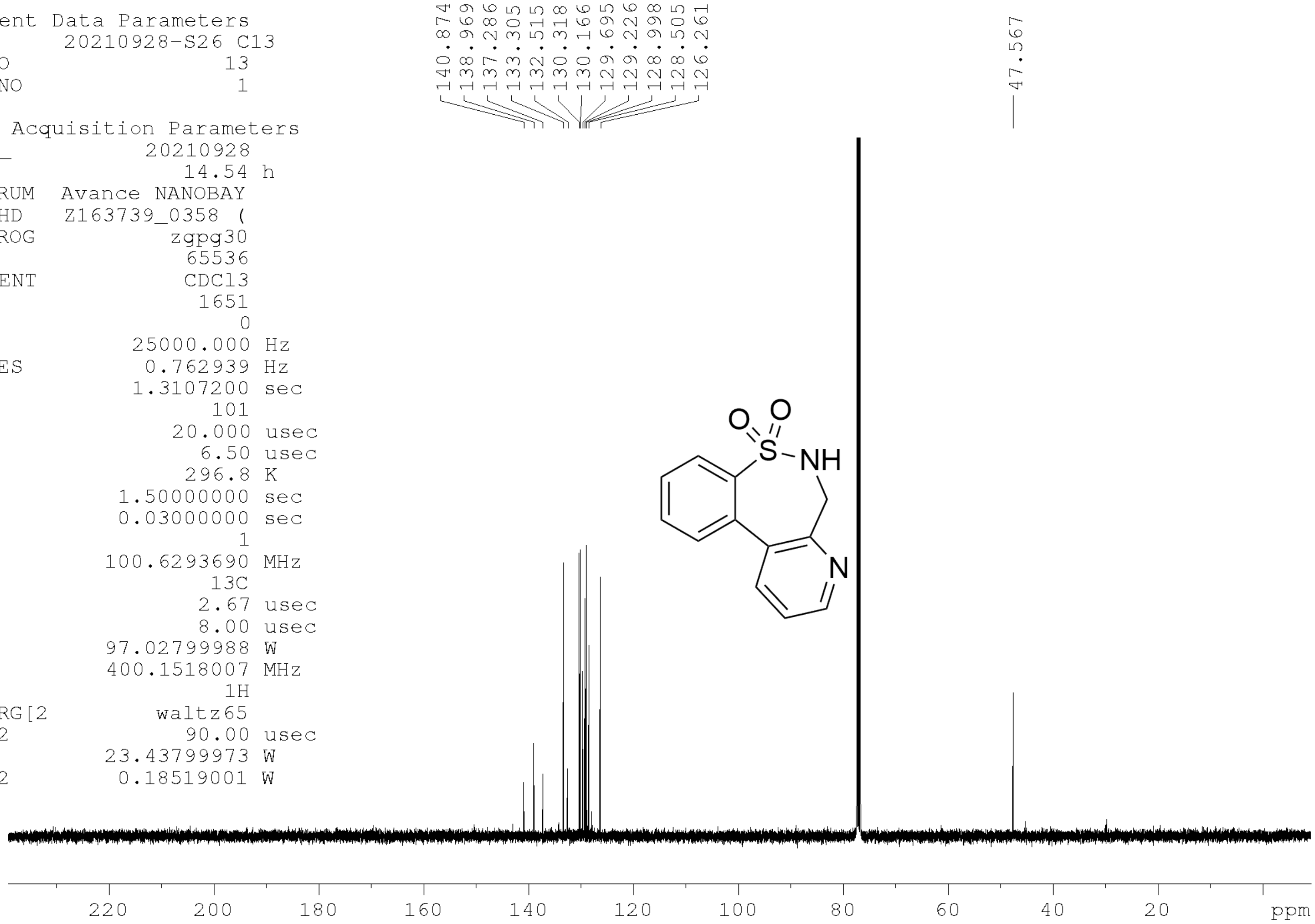

Supplement: Supplementary file 1 — asia70459‐sup‐0001‐Data.pdf [file ASIA-20-e00969-s002.pdf]
